# A Phase 1 Randomized, Open Label, Rectal Safety, Acceptability, Pharmacokinetic, and Pharmacodynamic Study of Three Formulations of Tenofovir 1% Gel (the CHARM-01 Study)

**DOI:** 10.1371/journal.pone.0125363

**Published:** 2015-05-05

**Authors:** Ian Mcgowan, Ross D. Cranston, Kathryn Duffill, Aaron Siegel, Jarret C. Engstrom, Alexyi Nikiforov, Cindy Jacobson, Khaja K. Rehman, Julie Elliott, Elena Khanukhova, Kaleab Abebe, Christine Mauck, Hans M. L. Spiegel, Charlene S. Dezzutti, Lisa C. Rohan, Mark A. Marzinke, Hiwot Hiruy, Craig W. Hendrix, Nicola Richardson-Harman, Peter A. Anton

**Affiliations:** 1 University of Pittsburgh, Pittsburgh, Pennsylvania, United States of America; 2 Magee-Womens Research Institute, Pittsburgh, Pennsylvania, United States of America; 3 David Geffen School of Medicine, University of California at Los Angeles, Los Angeles, California, United States of America; 4 CONRAD, Arlington, Virginia, United States of America; 5 HJF-DAIDS, a Division of The Henry M. Jackson Foundation for the Advancement of Military Medicine, Inc., Contractor to National Institute of Allergy and Infectious Diseases, National Institutes of Health, Department of Health and Human Services, Bethesda, Maryland, United States of America; 6 Department of Medicine, Johns Hopkins University, Baltimore, Maryland, United States of America; 7 Alpha StatConsult LLC, Damascus, Maryland, United States of America; Asociacion Civil Impacta Salud y Educacion, PERU

## Abstract

**Objectives:**

The CHARM-01 study characterized the safety, acceptability, pharmacokinetics (PK), and pharmacodynamics (PD) of three tenofovir (TFV) gels for rectal application. The vaginal formulation (VF) gel was previously used in the CAPRISA 004 and VOICE vaginal microbicide Phase 2B trials and the RMP-02/MTN-006 Phase 1 rectal safety study. The reduced glycerin VF (RGVF) gel was used in the MTN-007 Phase 1 rectal microbicide trial and is currently being evaluated in the MTN-017 Phase 2 rectal microbicide trial. A third rectal specific formulation (RF) gel was also evaluated in the CHARM-01 study.

**Methods:**

Participants received 4 mL of the three TFV gels in a blinded, crossover design: seven daily doses of RGVF, seven daily doses of RF, and six daily doses of placebo followed by one dose of VF, in a randomized sequence. Safety, acceptability, compartmental PK, and explant PD were monitored throughout the trial.

**Results:**

All three gels were found to be safe and acceptable. RF and RGVF PK were not significantly different. Median mucosal mononuclear cell (MMC) TFV-DP trended toward higher values for RF compared to RGVF (1136 and 320 fmol/10^6^ cells respectively). Use of each gel *in vivo* was associated with significant inhibition of *ex vivo* colorectal tissue HIV infection. There was also a significant negative correlation between the tissue levels of TFV, tissue TFV-DP, MMC TFV-DP, rectal fluid TFV, and explant HIV-1 infection.

**Conclusions:**

All three formulations were found to be safe and acceptable. However, the safety profile of the VF gel was only based on exposure to one dose whereas participants received seven doses of the RGVF and RF gels. There was a trend towards higher tissue MMC levels of TFV-DP associated with use of the RF gel. Use of all gels was associated with significant inhibition of *ex vivo* tissue HIV infection.

**Trial Registration:**

ClinicalTrials.gov NCT01575405

## Introduction

Rectal microbicides (RM) are currently being developed to prevent, or at least significantly reduce, the risk of HIV acquisition associated with unprotected receptive anal intercourse (URAI) [1]. Although evidence suggests that rates of new HIV infections in heterosexual populations are slowing, rates of new infection associated with URAI in men who have sex with men (MSM) and transgendered women are stable or increasing [2]. The recent approval of tenofovir disoproxil fumarate/emtricitabine for pre-exposure prophylaxis (PrEP) of HIV infection is a major step forward for HIV prevention; however, suboptimal adherence to oral PrEP can significantly reduce PrEP efficacy [3–5].Consequently, there is an urgent need to develop alternative approaches to PrEP, including a safe and effective RM. An RM that could be used in a pericoital fashion by men or women, especially if it had properties that made the product suitable for use as a sexual lubricant, might be an attractive PrEP option for individuals at risk of HIV infection through URAI.

Attention is currently being focused on the development of tenofovir (TFV) gel as a potential RM. The vaginal formulation of TFV used in the CAPRISA 004 study [6] has been evaluated in a Phase 1 rectal safety study (RMP-02/MTN-006) [7]. Use of the gel was associated with mild to moderate gastrointestinal symptoms including bloating, pain, urgency, and diarrhea. The vaginal formulation (VF) of TFV is hyperosmolar (3111 mOsmol/kg) and these symptoms may have been linked to product osmolality [8]. Consequently, the TFV gel used in a second Phase 1 study (MTN-007) was formulated with a lower glycerin concentration (5% w/w rather than the 20% w/w used in the RMP-02/MTN-006 vaginal formulation) to yield a product osmolality of 836 mOsmol/kg [9]. This reduced glycerin vaginal formulation (RGVF) was better tolerated by participants in the MTN-007 study [10] and is currently being evaluated in a Phase 2 expanded safety study (MTN-017; ClinicalTrials.gov Identifier: NCT01687218). As part of an ongoing program grant from the National Institutes of Health Integrated Preclinical-Clinical Program (IPCP) for HIV Topical Microbicides, we have developed a third rectal-specific formulation (RF) TFV gel. Compared to the RGVF TFV gel, the RF TFV gel contains less glycerin (2.5% w/w) and added carbopol (0.5% w/w), with a neutral pH and is nearly iso-osmolar (479 mOsmol/kg); it was also safe and effective in preclinical evaluation [11].

The purpose of the Combination HIV Antiretroviral Rectal Microbicide (CHARM)-01 study was to directly compare the safety, acceptability, and pharmacokinetic (PK) and pharmacodynamics (PD) profiles of the aforementioned TFV gel formulations in a crossover study. A second study (CHARM-02) was designed to complement the CHARM-01 study by evaluating systemic PK, the luminal distribution and clearance of the three gels, and their impact on mucosal permeability (manuscript in progress). The design of the CHARM-01 study includes multicompartmental PK, detailed assessment of mucosal safety (including histology, flow cytometry, and rectal microflora), and a PD component that utilizes an *ex vivo* colorectal HIV-1 challenge assay to generate preliminary efficacy data [7,12]. The goal of the CHARM-01 study was to provide a comprehensive data set that could guide decisions as to which TFV formulation should be advanced to later stage, larger, and more expensive studies.

## Materials and Methods

The protocol for this trial and supporting CONSORT checklist are available as supporting information; see S1 CONSORT Checklist and S1 Protocol.

### Ethics Statement

The study was designed by the investigators with collaborative input from CONRAD and the NIAID/DAIDS/Prevention Sciences IPCP for HIV Topical Microbicides, as stipulated in the award notice, and reviewed by the U.S. Food and Drug Administration (FDA). The study was approved by the University of Pittsburgh Institutional Review Board (IRB) as well as the University of California at Los Angeles IRB. All subjects provided written informed consent. The trial is registered at ClinicalTrials.gov, number # NCT01575405 and this paper is in compliance with the CONSORT 2010 recommendations for reporting of trial results (www.consort-statement.org) [13,14].

### Study Schema

**Fig 1:**
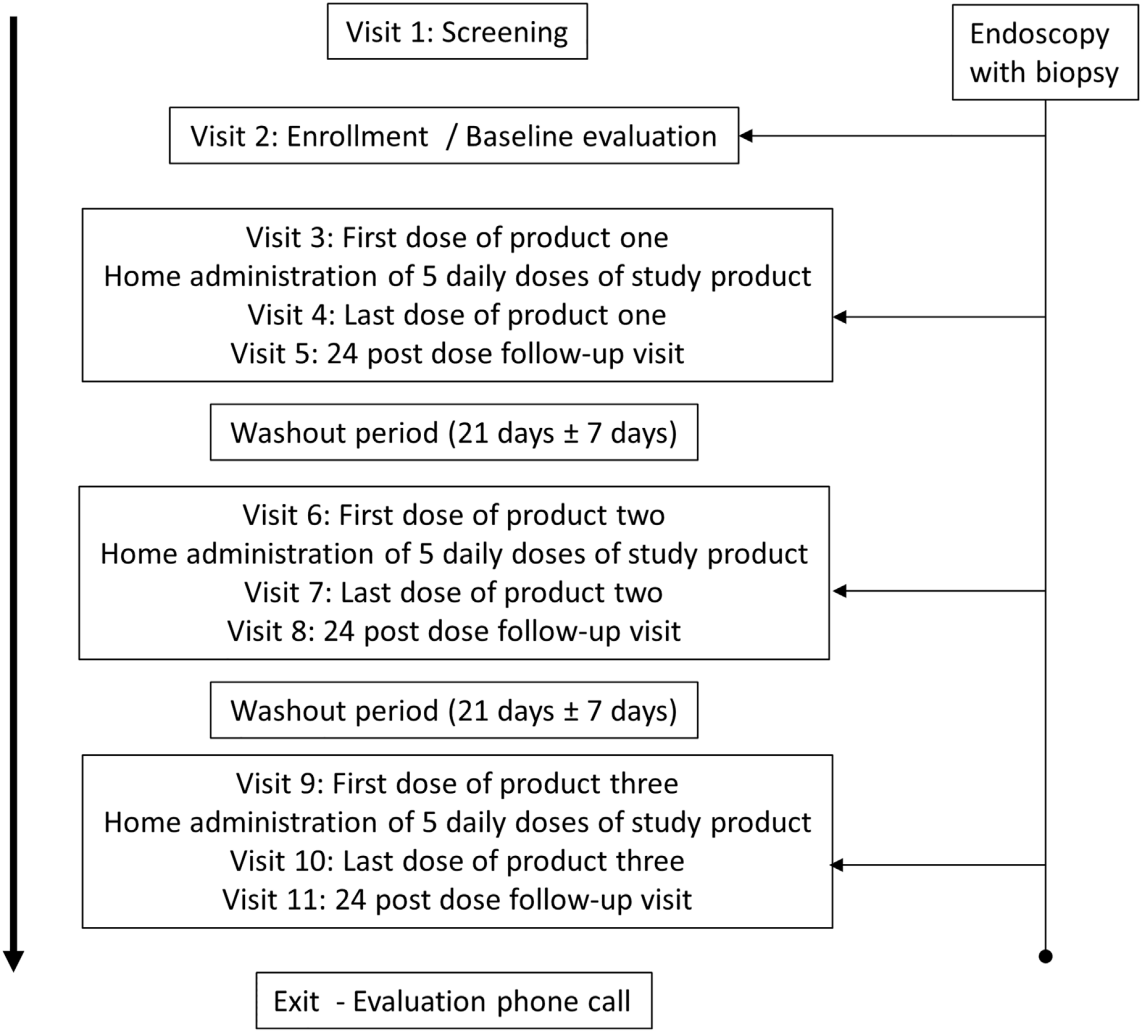
CHARM-01 study design.

The primary objectives of the CHARM-01 study were to evaluate the clinical safety, acceptability, and PK of three formulations of TFV 1% gel when applied rectally. The secondary study objective was to evaluate the mucosal safety of each formulation. The study also had an exploratory objective to assess the preliminary efficacy of each formulation in suppressing *ex vivo* HIV-1 viral replication in colorectal tissue. The CHARM-01 study was a Phase 1, double blind, randomized crossover trial in which participants received the three TFV gel formulations (VF, RGVF, and RF) in a randomized sequence. Each phase of product administration lasted 7 days with a 21 (± 7) day washout period between phases of product administration (Fig 1). The first and seventh doses of study product were administered in the clinic and the remaining five doses were administered by the participant at home, with daily, protocol-defined telephone call reminders to encourage product use.

During the RGVF and RF phase of dosing, participants received seven identical doses of either the RGVF or RF TFV gel. However, during the VF phase of dosing, participants received six doses of a hydroxyethyl cellulose (HEC) placebo gel [15], with only a final dose of VF TFV gel. As the majority of participants in the RMP-02/MTN-006 rectal safety trial who received VF TFV gel experienced gastrointestinal side effects (bloating, abdominal discomfort, and diarrhea) [7], it was considered unethical to ask participants to use more than one dose of VF TFV gel.

The study was conducted at two clinical sites (The University of Pittsburgh, Pittsburgh, Pennsylvania. and the David Geffen School of Medicine, University of California at Los Angeles, Los Angeles, California). Enrollment began in March 2013 and the last participant completed the study in October 2013. The target sample size was 18 (nine participants at each site) and enrolled participants were assigned at random to one of the three study formulation sequences. Randomization was done in blocks of three at each site to ensure balance between formulation groups and the sequence of administration between sites. The randomization scheme was stratified by site and generated by the University of Pittsburgh, Center for Research on Health Care Data Center, using computer-generated random numbers.

The randomization assignments for up to 12 participants (24 total per site) were delivered to the Director of Pharmacy Affairs at the Magee-Womens Research Institute (MWRI) who held primary responsibility for maintaining the blinding and generated the product labels.

### Study population

The study population consisted of healthy, RAI-abstinent, HIV-uninfected, adults (male and female) aged 18 years or older at time of screening who had been successfully vaccinated for hepatitis B virus (HBV) or who had naturally acquired immunity to HBV, as evidenced by HBV antibody titers. An additional inclusion criterion for female participants was the active use of an acceptable form of contraception (e.g., barrier method, intrauterine device, hormonal contraception, surgical sterilization, or vasectomization of the male partner). Individuals with abnormalities of the colorectal mucosa, significant gastrointestinal symptoms (such as a history of rectal bleeding), evidence of rectal *Chlamydia trachomatis* (CT) or *Neisseria gonorrhea* (GC) infection, chronic HBV infection, or a requirement to use drugs that were likely to increase the risk of bleeding following mucosal biopsy were excluded from the study.

### Study products

The VF TFV gel, the RGVF TFV gel, and the Universal HEC placebo gel were manufactured under direction from CONRAD (Arlington, VA) by DPT Laboratories (San Antonio, TX). DPT Laboratories also manufactured the RF TFV gel under direction of Dr. Lisa Rohan’s Group at MWRI. HTI applicators (HTI Plastics, Lincoln, NE) were used to deliver gel in the CHARM-01 study. These applicators had been initially designed for vaginal use and have been used in all of the previous vaginal microbicide trials with TFV gel. They have also been used rectally in the RMP-02/MTN-006 and MTN-007 studies [7,10]. Each opaque pre-filled applicator was packaged with a plunger and labeled with a code to preserve the identity of the formulation. Each pre-filled applicator contained a dose of approximately 4 mL of TFV gel or the HEC placebo. The pre-filled applicators were shipped directly to study site pharmacies and were stored by and dispensed from the site pharmacy.

Each participant was assigned applicators based on the randomization number. At Visits 3, 6, and 9 the participant’s first dose of study product was administered by the clinical staff. During the period of daily administration, study participants were instructed to insert one dose of gel into the rectum once daily for five days. A final dose of study product was administered by clinic staff at Visits 4, 7, and 10.

### Study procedures

There were a total of eleven study visits and one follow-up phone call. After obtaining informed consent all participants were screened with a thorough medical history, a targeted physical examination, a digital rectal examination, and rectal swabs for CT/GC nucleic acid amplification testing (NAAT). Urine was also collected for CT/GC NAAT and for pregnancy testing in the female participants (pregnancy testing was repeated at all subsequent clinical visits). Blood was collected for safety labs (complete blood count, urea nitrogen, creatinine, alanine aminotransferase, and aspartate aminotransferase) and serology (syphilis, HIV-1, hepatitis B, and herpes simplex 1 and 2). Participants who met the aforementioned inclusion criteria during the Screening Visit were enrolled into the study. The Enrollment Visit occurred within 28 days of screening. At the Enrollment Visit, participants were randomized, a web-based behavioral questionnaire was administered, and a rectal examination and focused physical examination were performed. Rectal swabs were collected for CT/GC. Rectal sponges for quantification of cytokines/chemokines in rectal secretions, and PK were also collected. Participants then received a normal saline pH 7.4 enema. A flexible sigmoidoscope was inserted into the rectum and 21 biopsies were collected at approximately 15 cm from the anal verge. Biopsies were used for histology, gene expression, flow cytometry, PK, and *ex-vivo* tissue challenges. At Visits 3, 6, and 9 (Treatment Initiation Visits), all participants had a single applicator of study gel inserted into the rectum. Prior to product insertion, samples were collected for CT/GC, microflora and cytokines. At Visits 4, 7, and 10 (Last Dose Treatment Visits) sponges were collected for quantification of cytokines in rectal fluid. A normal saline enema was then administered followed by a single dose of study product. Approximately 30 minutes later (± 15 minutes) blood, and in females, self-collected vaginal sponges were collected for PK studies. A sigmoidoscope was then inserted and the same rectal tissue biopsy samples were collected as described during the Enrollment Visit. Additional blood and rectal/vaginal sponges were collected at 2 hours (± 30 minutes) and 4 hours (± 30 minutes) after product insertion. At Visits 5, 8, and 11 (conducted 18–30 hours after Visits 4, 7, or 10) blood and rectal/vaginal sponges were collected for PK. Rectal swabs were collected for assessment of microflora after gel use. A web-based questionnaire was also conducted at the end of each period of gel use. At Visit 11 only, blood was collected for HBsAg serology. A brief qualitative interview was conducted after Visit 11 to assess overall product acceptability. A final telephone call follow-up assessment was conducted within 14 days of Visit 11.

### Clinical safety and laboratory assessments

Emergent adverse events (AEs) were graded using the Division of AIDS Table for Grading the Severity of Adult and Pediatric Adverse Events, Version 1.0, December 2004 as well as Addendum 1 and 3 (*Female Genital and Rectal Grading Table for Use in Microbicide Studies* (http://rsc.tech-res.com/safetyandpharmacovigilance/). In cases where an AE was covered in both tables, the *Female Genital or Rectal Grading Table for Use in Microbicide Studies* was the grading scale utilized.

### Product acceptability and adherence

Overall product like (or dislike) and likelihood of gel use in the future were assessed using an internet-based computer-assisted self-interview (CASI). The Wisebag, a lunch bag-style container with an electronic events-monitoring system, is designed as a real-time indirect objective measure of microbicide gel use and was used in the CHARM-01 study to monitor product adherence [16].

### Mucosal safety

#### Histology

A qualitative scoring system developed for inflammatory bowel disease (IBD) research [17] and adapted for use in RM trials [18] was used to characterize potential product-associated injury with a scale of 1 (normal) to 5 (mucosal erosion or ulceration).

#### Rectal microflora

Rectal microflora was characterized using previously described semi-quantitative culture analysis techniques [19,20] that have been used in other RM Phase 1 studies [7,10].

#### Mucosal T cell phenotype

Mucosal mononuclear cells (MMC) were isolated from rectal biopsies using a combination of mechanical and enzyme digestion as previously described [10]. Flow cytometric analysis was performed on a BD LSRFortessa cytometer (BD Biosciences, San Jose, CA). All data were stored in list mode and analyzed with BD FACSDIVA operating system and Flow Jo (Tree Star, Inc., Ashland, OR). All antibodies were purchased from BD Biosciences, San Jose, CA (PerCP-CD45, Clone 2D1; Pacific Blue-CD3, Clone UCHT1; PE-Cy7-CD4, Clone SK3; APC-H7-CD8, Clone SK1; FITC-CD69, Clone FN50; APC-CD184 (CXCR4), Clone 12G5 and PE-CD195 (CCR5)) and titrated under assay conditions to determine an optimum saturating dilution. Cells were stained with LIVE/DEAD Fixable Aqua stain fluorescence (Life Technologies, Eugene, OR) to define viable cells. The gating strategy used in this study can be found in S1 Fig.

### Pharmacokinetic procedures

Blood plasma, peripheral blood mononuclear cells (PBMCs), vaginal and rectal fluid, and rectal tissue were obtained before rectal dosing (Visit 2) and 30 minutes after the seventh dose of the gels (Visits 4, 7, and 10). At 2, 4, and 24h after the final dose, blood plasma, PBMCs, and rectal/vaginal fluid samples were obtained (Visits 5, 8, and 11).

#### Sample Processing

TFV and TFV-DP concentrations were determined via validated liquid chromatographic-tandem mass spectrometric (LC-MS/MS) methods at The Johns Hopkins University Clinical Pharmacology Analytical Laboratory as described previously [21]. All assays were validated following the recommendations of the FDA, Guidance for Industry: Bioanalytical Method Validation guidance document. TFV concentrations were determined in plasma, rectal fluid, and vaginal fluid. TFV-DP concentrations were determined for PBMCs, rectal tissue homogenates, and rectal MMCs. The measured value from each PK assay was used unless the PK value was determined to be between the lower limit of quantification (LLOQ) and the lower limit of detection (LLOD), in which case, a number equal to half that assay’s LLOQ was imputed for that PK value.

### *Ex vivo* tissue biopsy challenge assay

At Enrollment (Visit 2) and post-product exposure timepoints (Visits 4, 7, and 10), endoscopic biopsies were collected in 20 mL RPMI (with 1.125 μg/mL of Fungizone and 0.5 mg/mL of Zosyn) and transported to the laboratory for *ex vivo* infection within 1–2 hours using a common viral stock of HIV-1_BaL_ (10^4^ TCID_50_), as previously described [7,12]. Supernatants for p24 quantification were collected every three days during each 14-day infectibility assay (Days 4, 7, 11 & 14). Results were adjusted for biopsy weight, averaged across quadruplicate assays, and reported as Day 14 cumulative p24 (p24 HIV antigen ELISA; NCI, Bethesda, MD) where the assay’s LLOQ was 10 pg/mL. Non-detectable cumulative p24 measures at Day 14 were converted to 1/2 the LLOQ prior to log transformation.

### Analysis of outcomes

Below, we describe the analyses used for each of the primary and secondary outcomes. SAS version 9.2 was used for the statistical analyses. The primary outcome of safety was assessed at the 5% type I error rate. The target sample size of 18 was chosen to ensure an 85% probability of observing at least one Grade 2 or higher adverse event in an arm when the true event rate is 10%. Each of the secondary outcomes was assessed at the nominal significance level without adjustments for multiplicity.

#### Safety

For safety analysis, the number and frequency of ≥ Grade 2 adverse events (AEs) were tabulated for each of the three study formulations. To determine the extent of AEs, the proportion of subjects that experienced an AE was calculated for each study formulation. The rate of safety events was compared between the RF formulation and either the VF or RGVF formulations using McNemar’s test for each comparison. This was conducted after the final dosing visits (Visits 4, 7, and 10, respectively).

#### Acceptability

We calculated the proportion of participants who reported product characteristics that were considered to be a barrier in use, operationalized as having a rating of lower than 3 on a 5-point Likert scale, in disliking or likelihood of future barrier in use. Acceptability was defined as the converse (rating of ≥ 3). This was calculated for each of the following product characteristics: “consistency”, “color”, “smell”, “taste”, “stickiness”, “feeling inside rectum”, “need for lubrication”, and “sexiness”. McNemar’s test was then used to compare each pair of formulations with respect to acceptability.

#### Pharmacokinetics

TFV-based gel formulations’ PK were evaluated in six compartments (plasma, PBMCs, rectal fluid, rectal tissue, rectal MMCs, and cervicovaginal fluid) after rectal administration of the study product. For matrices other than tissue which were sampled multiple times after the last dose, the 24 hour post-dose concentration and time profiles were examined following the final rectal dose of each TFV-containing study product. This occurred after 7 doses for the RF and RGVF gels and after 1 dose for the VF gel). TFV (or TFV-DP in PBMCs) were characterized in terms of maximum concentration (C_max_), time to maximum concentration (T_max_), and area under the TFV concentration-time curve from 0 to 24h (AUC_0-24_ [log-linear trapezoidal method]) using non-compartmental methods (WinNonlin v. 6.3 software, Pharsight, St. Louis, MO). Rectal biopsies, which were sampled only once with each product, were taken 30 minutes after each final study product dose to determine TFV and TFV-DP concentrations in tissue homogenates and TFV-DP in MMCs. We performed paired comparisons between RF and RGVF using the Wilcoxon rank sum test with exact two-sided significance test (alpha ≤ 0.05). VF PK was not compared to either RF or RGVF due to non-steady state conditions; only one TFV-containing dose was given during the VF phase of the study.

#### Mucosal Immunotoxicity

The association of mucosal parameters with study products was examined. Flow cytometry and histopathology parameters were collected at the enrollment/baseline visit (Visit 2) as well as after the 7^th^ dose of each formulation (Visits 4, 7, and 10). Changes between each of the latter visits and the baseline visit were calculated for the flow cytometry parameters and used as the outcome for the analysis. Microflora anaerobic and facultative parameters were collected at the 1^st^ dose (Visits 3, 6, and 9) and 24hr post dose for each formulation (Visits 5, 8, and 11), and changes between each 1^st^ and 24hr post dose (i.e. Visit 5 minus Visit 3, Visit 8 minus Visit 6 etc.) were calculated and used as the outcome. GEE models were utilized for all outcomes with different link functions to account for the distributional nature (i.e. continuous versus binary). In addition, we assumed an exchangeable within-subject correlation, which assumes the correlation between any two visits is the same. Predictors in the model included drug formulation, visit (or stage), and their interaction. We first tested the interaction to confirm the non-existence of a drug formulation carryover effect. If significant, we based our inference on information at Stage 1. Otherwise, we ran an additive model (w/o interaction term) as our final model. Each participant served as his or her own control and effects were tested using two-sided Wald significance tests with α = 0.05.

#### Pharmacodynamics

Cumulative p24 at Day 14 was used as the primary measure for *ex vivo* tissue infectibility. This was measured at the enrollment/baseline visit (Visit 2) as well as 30 minutes after the 7^th^ dose of each formulation (Visits 4, 7, and 10). Changes between each of the latter visits and the baseline visit were calculated for 14-day cumulative p24. A natural log transformation was then taken and used as the outcome for the analysis. GEE models were utilized with an identity link function and an exchangeable correlation structure to account for the within-participant correlation. Predictors in the model included drug formulation, visit (or stage), and their interaction. Due to the crossover design of the trial, we first tested the interaction to confirm the non-existence of a drug formulation carryover effect. If significant, we based our inference on information at Stage 1. Otherwise, we ran an additive model (w/o interaction term) as our final model.

#### Correlation between Pharmacokinetic and Pharmacodynamic data

TFV measurements from three compartments (rectal fluid, plasma, and rectal tissue) and TFV-DP measurements from three compartments (PBMC, MMCs, and rectal tissue) were log_10_ transformed and paired with the corresponding log_10_ transformed cumulative p24 at Day 14 from the *ex vivo* tissue HIV infections for each subject and sampling time. A linear regression model was fitted to the paired PK:PD data set for each PK compartment, where subject was a covariate [22].

## Results

### Enrollment and retention

**Fig 2:**
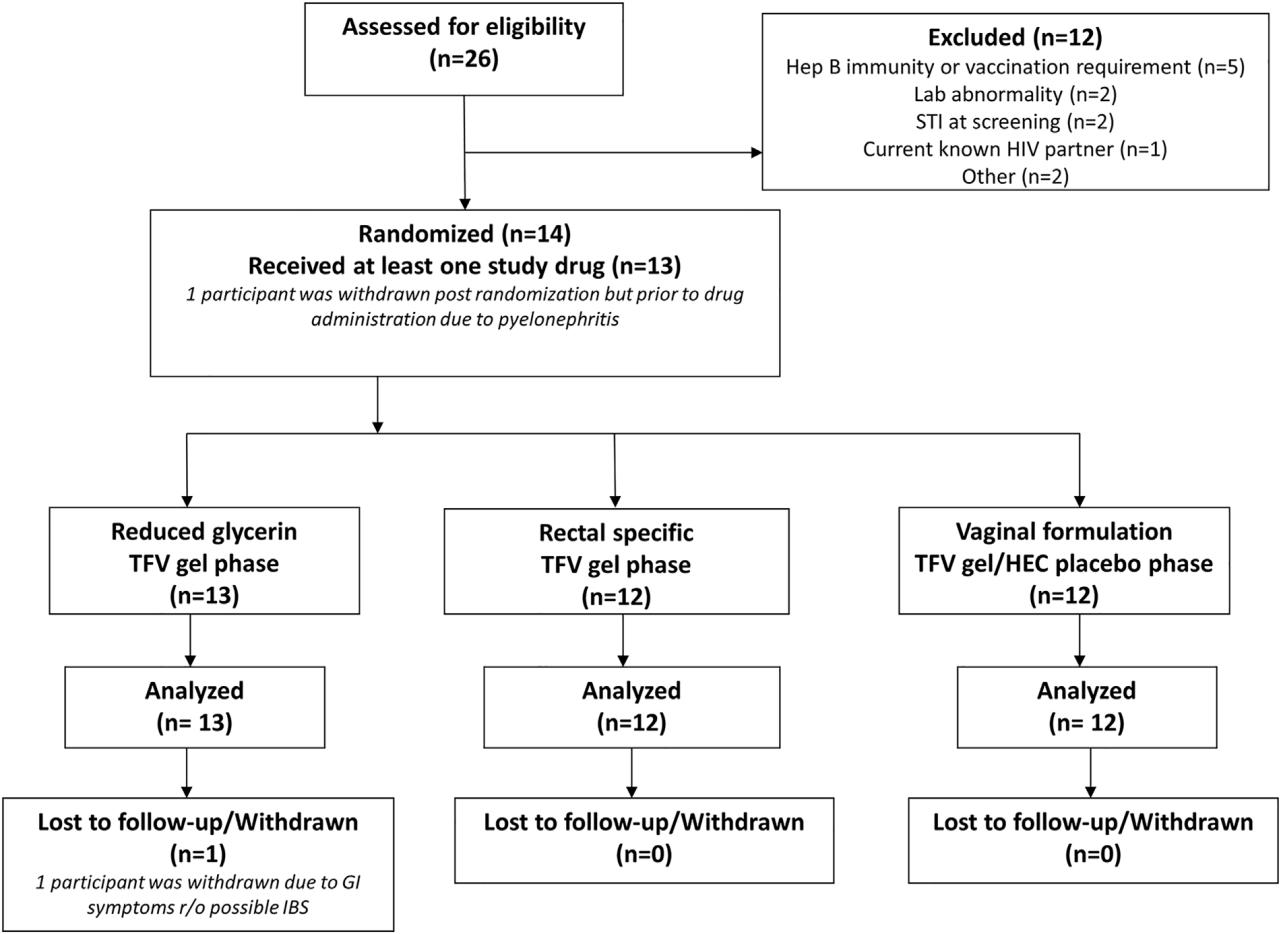
Flow diagram of participant progress through the CHARM-01 study.

A total of 14 participants (11 men and 3 women) were enrolled and randomized in the study (Fig 2), 12 of whom completed the study. The majority of participants were white (57%) with a mean age of 37.7 (± 14.3) years (Table 1). There was no statistical difference between sites in gender composition or the proportion of white participants, although there was a difference with respect to age (41.7 versus 23.0; *P* = 0.04) with UCLA having an older cohort. One female participant was enrolled but developed pyelonephritis prior to product exposure and was removed from the study. A second participant was randomized to receive the RGVF gel as the first study product. The participant completed Visit 5 but was subsequently withdrawn due to gastrointestinal symptoms including bloating and abdominal discomfort suggestive of irritable bowel syndrome. All other participants completed the study. Averaged across all study visits, the proportion of completed administrative procedures, clinical procedures, clinical laboratory sample collection, and research laboratory sample collection was 89%, 87%, 96%, and 86% respectively.

**Table 1:** Baseline demographic data by site.

Variables	UCLA(n = 11)	PITT(n = 3)	Overall(n = 14)
**Age**	41.7 ± 13.6	23.0 ± 1.7	37.7 ± 14.3
**Male**	9(81.82%)	2(66.67%)	11(78.57%)
**Race**			
White	6(54.55%)	2(66.67%)	8(57.14%)
Black or African American	4(36.36%)	1(33.33%)	5(35.71%)
American Indian or Alaska Native	1(9.09%)	0(0.00%)	1(7.14%)
**Hispanic**			
No, not of Hispanic, Latino/a, or Spanish origin	9(81.82%)	3(100.00%)	12(85.71%)
Yes, Mexican, Mexican American, Chicano/a	1(9.09%)	0(0.00%)	1(7.14%)
Yes, Another Hispanic, Latino/a or Spanish origin	1(9.09%)	0(0.00%)	1(7.14%)

### Safety

Adverse events were generally mild (Grade 1, N = 25, 86% of all AEs) or moderate (Grade 2, N = 2, 7% of all AEs). Two Grade 3 events occurred during the VF TFV phase of the study. Both Grade 3 events were in a female participant with pyelonephritis which occurred prior to product administration and were considered unrelated to study product. Gastrointestinal adverse events were common but the majority 17/18 (94%) were mild (Table 2). There were no significant differences in the proportion of participants with ≥ Grade 2 or higher adverse events across the phases of the study (Table 2).

**Table 2:** Adverse events by formulation.

Overall Events	Severity	Overall		RF TFV		RGVF TFV		VF TFV	
		N	%	N	%	N	%	N	%
	Grade 1	25	86.21	7	100.00	7	87.50	11	78.57
	Grade 2	2	6.90	0	0.00	1	12.50	1	7.14
	Grade 3	2	6.90	0	0.00	0	0.00	2	14.29
	Total	29	100	7	100	8	100	14	100
**Overall Events By System**									
**Gastrointestinal**	Grade 1—Mild	17	94.44	5	100	5	83.33	7	100
**Gastrointestinal**	Grade 2—Moderate	1	5.56	0	0.00	1	16.67	0	0.00
**Total**		18	100	5	100	6	100	7	100
**Genitourinary**	Grade 2—Moderate	1	33.33	0	0.00	0	0.00	1	33.33
**Genitourinary**	Grade 3—Severe	2	66.67	0	0.00	0	0.00	2	66.67
**Total**		3	100	0	0.00	0	0.00	3	100
**HEENT**	Grade 1—Mild	2	100	0	0.00	2	100	0	0.00
**Total**		2	100	0	0.00	2	0.00	0	0.00
**Neurological**	Grade 1—Mild	5	100	2	100	0	0.00	3	100
**Total**		5	100	2	100	0	0.00	3	100
**Respiratory**	Grade 1—Mild	1	100	0	0.00	0	0.00	1	100
**Total**		1	100	0	0.00	0	0.00	1	100
**Proportion of participants with≥ Grade 2 adverse events**				0	0.00	1	7.69	1	7.69

### Acceptability

Acceptability was evaluated for the following product characteristics; perception of product consistency, smell, taste, color, stickiness, lubrication, feeling inside the rectum, and sexiness. The ranges for product acceptability characteristics were 75%-100% (RF gel), 82%-100% (RGVF gel), and 82%-100% (VF). There were no significant differences in acceptability for any of the characteristics evaluated across the three study products.

### Adherence

Wisebags were used as a surrogate for product adherence for the five doses that were to be administered at home. Participants at both sites indicated on self-report that they administered the five consecutive doses of gel for each of the three formulations. This was also reported by count of used and unused applicators. Unfortunately, due to a number of equipment and data retrieval problems the self-report adherence data could not be supported or refuted by Wisebag data (manuscript in preparation).

### Mucosal safety

#### Histology

There were no significant increases in histology scores between Visit 2 (Enrollment) and Day 7 of dosing (Visits 4, 7, or 10), or across the three study formulations (data not shown).

#### Mucosal T cell phenotype

Significant changes in T cell phenotype between Visit 2 and Visits 4, 7, or 10 across the three study formulations are summarized in Table 3 (complete data are provided in S1 Table). Briefly, a significant increase between Visit 2 and Day 7 of dosing was seen for CD3+/CD45+ T cells (RF TFV gel (*P* = 0.038) and VF TFV gel(*P* = 0.0005)).

**Table 3:** Flow cytometry data.*

Flow parameter	Enrollment(n = 14)	Mean at 7^th^ Dose	Change at 7^th^ Dose(n = 13)	*P* value**
	Mean (SD), Median (25%, 75%)	N, Mean (SD), Median (25%, 75%)	Mean (SE)	
**% CD3** ^**+**^ **from CD45** ^**+**^	44.4 (17.4), 47.2 (34.6, 58.9)			
Enrollment vs. RF D7		11, 53.8 (16.4), 54.8 (46.9, 66.2)	12.23 (5.89)	0.0380
Enrollment vs. HEC/VF D7		12, 54.6 (18.9), 58.3 (49.8, 63.4)	11.98 (3.46)	0.0005
**% CXCR4** ^**+**^ **from CD4** ^**+**^	71.5 (16.1), 70.3 (57.9, 84.5)			
Enrollment vs. HEC/VF D7		12, 61.1 (23.2), 61.4 (55.0, 79.5)	-10.68 (5.40)	0.0480
Change at 7^th^ Dose (RGVF v HEC/VF)			16.4 (6.70)	0.0142
**% CD69** ^**+**^ **from CD4** ^**+**^	83.4 (6.0), 83.9 (80.4, 87.4)			
Enrollment vs. HEC/VF D7		12, 80.5 (4.9), 82.3 (78.7, 83.5)	-2.27 (0.97)	0.0188
**% CXCR4** ^**+**^ **and CCR5** ^**+**^ **from CD4** ^**+**^	55.6 (11.9), 55.1 (43.8, 66.2)			
Enrollment vs. HEC/VF D7		12, 45.4 (15.9), 48.7 (41.2, 53.9)	-10.16 (4.20)	0.0157
Change at 7^th^ Dose (RGVF v HEC/VF)			15.70 (5.58)	0.0049
**% CXCR4** ^**+**^ **from CD8** ^**+**^	51.2 (17.1), 47.8 (40.2, 68.1)			
Enrollment vs. HEC/VF D7		12, 39.3 (18.8), 38.3 (33.9, 54.2)	-13.38 (4.58)	0.0035
**% CD69** ^**+**^ **from CD8** ^**+**^	85.7 (6.1), 84.7 (82.1, 90.5)			
Enrollment vs. RGVF D7		13, 71.4 (26.6), 80.2 (78.1, 84.3)	-13.54 (6.17)	0.0283
Change at 7^th^ Dose (RF v RGVF)			12.80 (6.02)	0.0336
**% CXCR4** ^**+**^ **and CCR5** ^**+**^ **from CD8** ^**+**^	43.2 (13.1), 42.5 (35.6, 48.0)			
Enrollment vs. HEC/VF D7		12, 31.5 (13.8), 34.0 (29.1, 37.7)	-12.51 (3.66)	0.0006

*Table 3 only lists phenotypes where significant variation was noted between visits of between products. Complete flow cytometry data are provided in S1 Table

**P-value from significance test of relevant contrast from GEE model.

A significant decrease between the time of enrollment and Day 7 of dosing was seen for the following T cell phenotypes: CXCR4+/CD4+ (VF TFV gel; *P* = 0.048), CD69+/CD4+ (VF TFV gel; *P* = 0.0188), CXCR4+/CCR5+/CD4+ (VF TFV gel; *P* = 0.0157), CXCR4+/CD8+ (VF TFV gel; *P* = 0.0035), CD69+/CD8+ (RGVF TFV gel; *P* = 0.0283), and CXCR4+/CCR5+/CD8+ (VF TFV gel; *P* = 0.0006). Significant differences were seen between formulations for the following T cell phenotypes: CXCR4+/CD4+ (RGVF TFV gel versus VF TFV gel; *P* = 0.0142), CXCR4+/CCR5+/CD4+ (RGVF TFV gel versus VF TFV gel; *P* = 0.0049), and CD69+/CD8+ (RF TFV gel versus RGVF TFV gel; *P* = 0.0336).

#### Rectal microflora

*Bacteroides fragilis* was increased in the 24 hr Post Dose sample compared to the 1^st^ Dose sample (RGVF gel; *P* = 0.046) and reduced in the 24 hr Post Dose sample compared to the 1^st^ Dose sample (VF gel; *P* = 0.018). *Lactobacillus* (H_2_O_2_ negative) was reduced in the 24 hr Post Dose sample compared to the 1^st^ Dose sample (RF gel; *P* = 0.0325). Gram positive rods were reduced in the 24 hr Post Dose sample compared to the 1^st^ Dose sample (RF gel; *P* = 0.0422). *Viridans streptococcus* was reduced in the 24 hr Post Dose sample compared to the 1^st^ Dose sample (RF gel; *P* = 0.0110).The majority of changes resulted in a decrease in the prevalence of these organisms (Table 4; complete data are provided in S2 Table).

**Table 4:** Microbiology data.

	1^st^ Dose	24hr Post Dose	Change(n = 13)	*P* Value**
	Descriptive Statistics*	Descriptive Statistics		
	N, Mean (SD), Median (25%, 75%)	N, Mean (SD), Median (25%, 75%)	Diff (SE)	
**Rectal Microflora Cultures (Anaerobic)**				
***Bacteriodes fragilis***				
RGVF: 1^st^ Dose vs. 24hr Post Dose	13, 2.2 (1.6), 2 (1, 4)	13, 3.2 (1.1), 3 (3, 4)	1.05 (0.53)	0.0460
HEC/VF: 1^st^ Dose vs. 24hr Post Dose	12, 3.1 (0.7), 3 (3, 3.5)	12, 2.1 (1.2), 2 (2, 3)	-0.99 (0.32)	0.0018
Change at 24hr Post Dose (RGVF v HEC/VF)			2.04 (0.73)	0.0051
**Rectal Microflora Cultures (Facultative Isolates)**				
***Lactobacillus*, H2O2 negative**				
RF: 1^st^ Dose vs. 24hr Post Dose	12, 0.8 (1.1), 0 (0, 2)	12, 0.3 (0.6), 0 (0, 0)	-0.57 (0.26)	0.0325
Change at 24hr Post Dose (RF v HEC/VF)			-0.74 (0.24)	0.0023
**Gram positive rods, other**				
RF: 1^st^ Dose vs. 24hr Post Dose	12, 2.0 (1.1), 2 (1, 3)	12, 1.4 (1.3), 2 (0, 2.5)	-0.68 (0.34)	0.0422
***Viridans streptococcus*, H2O2-positive**				
HEC/VF: 1^st^ Dose vs. 24hr Post Dose	12, 1.3 (1.4), 1.5 (0, 2)	12, 0.8 (1.2), 0 (0, 1.5)	-0.50 (0.20)	0.0110
***Escherichia coli***				
Change at 24hr Post Dose (RF v HEC/VF)			-0.98 (0.39)	0.0117

*Semi-quantitative score (0 = no growth; 4 = 10^7^ colony forming units/mL)

** *P*-value from significance test of relevant contrast from GEE model.

### Pharmacokinetics

TFV moieties were detected in all compartments sampled, except for PBMC TFV-DP which was below the LLOQ for all products (Table 5). The plasma TFV concentration-time profile (S2 Fig), C_max_, T_max_, and AUC_0-24_ were not significantly different for the RF and RGVF products. There were no differences between RF and RGVF in TFV or TFV-DP in rectal tissue homogenate. Median mucosal mononuclear cell (MMC) TFV-DP trended toward higher values for RF compared to RGVF (1136 (IQR; 473–2200) and 320 (IQR; 170–1151) fmol/10^6^ cells respectively). As mentioned previously, only a single exposure (Day 7) of the vaginally-formulated TFV 1% gel was given to those during their randomization to the VF arm; consequently, the VF product findings for PK are not summarized here.

**Table 5:** Pharmacokinetic data are summarized as median (interquartile range).*

Matrix	Moiety	PK	Units	RF TFV	RGVF TFV	VF TFV
Plasma	TFV	C_max_	ng/mL	7.1 (3.5–11.9)	6.0 (4.3–7.1)	5.1 (3.3–6.2)
		AUC	ng*hr/mL	78 (33–135)	64 (28–97)	36 (23–57)
PBMC	TFV-DP		fmol/M	All BLQ	All BLQ	All BLQ
Colon tissue	TFV	30’	ng/mg	2.9 (0.5–5.8)	1.4 (0.7–3.7)	1.0 (0.1–9.2)
	TFV-DP	30’	ng/mg	10.3 (BLQ-36.8)	5.2 (BLQ-12.8)	BLQ (BLQ-6.4)
Colon tissue MMC	TFV-DP	30’	fmol/M	1136 (473–2200)	320 (170–1151)	91 (19–367)
Rectal Fluid	TFV	C_max_	ng/mL	8.1x10^5^ (1.8 x10^5^-1.6 x10^6^)	9.4 x10^5^ (4.3x10^5^-1.4x10^6^)	3.6x10^5^ (8x10^4^-8.2x10^5^)
		AUC	ng*hr/mL	1.4 x10^6^ (4.5x10^5^-2.9x10^6^)	1.4 x10^6^ (6.6x10^5^-2.5x10^6^)	7.9x10^5^ (5x10^5^-1.4x10^6^)
Vaginal Fluid**	TFV	C_max_	ng/mL	186; 7,526	1,824; 2,460	39; 132
		AUC	ng*hr/mL	263; 4,469	1,381; 2,556	22; 118

BLQ; Below the level of quantification

### Pharmacodynamics

Exposure to all three formulations resulted in a significant reduction in *ex vivo* HIV infection in rectal tissues collected 30 minutes after the last dose of each study product (Visits 4, 7, and 10) as compared to baseline (Visit 2) (Table 6). In addition, the degree of viral suppression associated with one week of daily dosing of the RF TFV gel was significantly greater than that observed 30 minutes after the single (Day 7) dose of the VF TFV gel. There was no difference between the RF and RGVF TFV gels in terms of the degree of viral suppression seen in the explant tissue.

**Table 6:** Tissue pharmacodynamic data.

Tissue Infection and 14-day Log10 Cumulative p24 pg/mL	Enrollment(n = 11)	Mean at 7^th^ Dose	Change at 7^th^ Dose(n = 11)	*P* value*
	Mean (SD), Median (25%, 75%)	N, Mean (SD), Median (25%, 75%)	Mean (SE)	
	2.93 (0.38), 3.01 (2.71, 3.08)			
Enrollment vs. RF D7		10, 1.87 (1.03), 1.99 (0.73, 2.92)	-1.02 (0.26)	<0.0001
Enrollment vs. RGVF D7		11, 2.15 (1.01), 2.58 (0.89, 2.78)	-0.82 (0.24)	0.0008
Enrollment vs. HEC/VF D7		11, 2.41 (0.51), 2.43 (2.20, 2.87)	-0.51 (0.17)	0.0024
Change at 7^th^ Dose (RF v RGVF)			-0.21 (0.23)	0.3610
Change at 7^th^ Dose (RF v HEC/VF)			-0.51 (0.25)	0.0420
Change at 7^th^ Dose (RGVF v HEC/VF)			-0.31 (0.21)	0.1388

* *P*-value from significance test of relevant contrast from GEE model.

### Correlation between pharmacokinetics and pharmacodynamics

**Fig 3:**
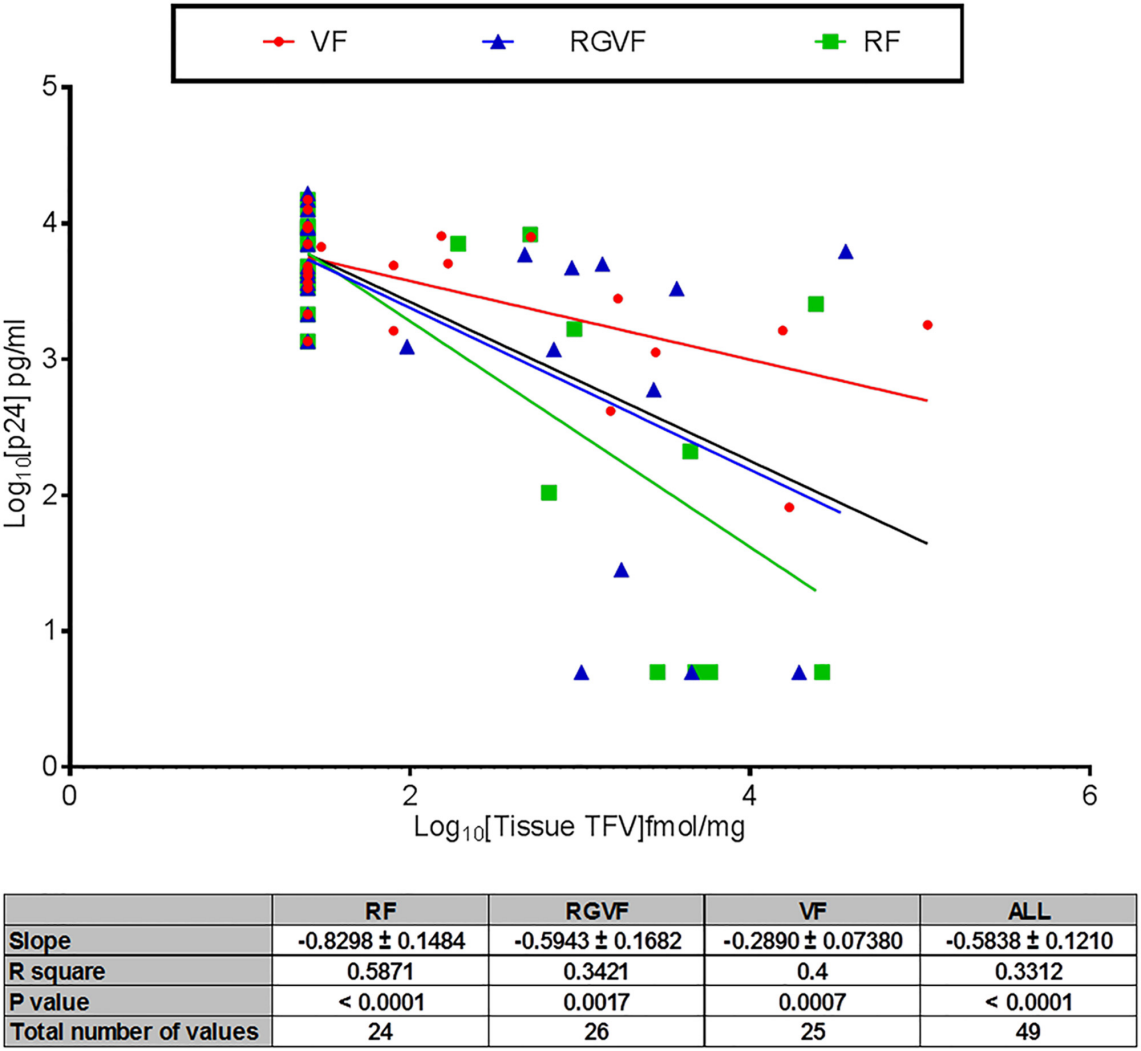
PK/PD relationship between rectal tissue TFV and colorectal tissue supernatant HIV-1 p24 after use of the VF TFV gel, the RGVF TFV gel, and the RF TFV gel. The black line represents PK/PD relationship for the entire data set across all three formulations.

There was a significant negative correlation between tissue concentrations of TFV (*P* < 0.05; Fig 3), tissue TFV-DP, MMC TFV-DP, rectal fluid TFV, and tissue HIV-1 infection (S3 Fig). This observation was observed and consistent for all three formulations. Significant negative correlations between tissue HIV infection and plasma TFV concentrations for the RF and VF TFV gels were observed (*P* < 0.01); however, this trend was not repeated for the RGVF (*P* > 0.05) TFV gel. When all data were pooled, there was an overall significant correlation between plasma TFV concentrations and inhibition of colorectal tissue HIV-1 infection (*P* < 0.05).

## Discussion

With the important caveat that only one dose of the VF TFV gel was administered in this study, the CHARM-01 study demonstrated that all three formulations of TFV gel evaluated in this Phase 1 study were safe and acceptable. Rectal exposure to study products was associated with the detection of TFV in plasma, rectal fluid, and rectal tissue and TFV-DP in rectal tissue and tissue MMC but not in PBMCs. As previously reported, rectal exposure to TFV gels was also associated with detection of TFV in vaginal fluids [7, 23]. Use of all three formulations resulted in significant suppression of HIV-1 viral replication in the *ex vivo* colorectal challenge assay, which correlated with local tissue concentrations of TFV.

We were unable to fully enroll the CHARM-01 study due to a combination of slow participant accrual and product expiration. In the early stage of product development microbicide gel formulations may have limited stability. It is therefore critical that the timing of studies take into consideration product expiration. However, as with our study, unanticipated regulatory delays or slow enrollment may still lead to premature closure of a study.

The majority of reported adverse events in the study were gastrointestinal and of mild severity. There were no differences in rates of adverse events across the three formulations although as mentioned above the VF TFV gel exposure was limited to one exposure which is probably why we did not see more gastrointestinal events in the VF TFV gel phase of dosing. The RGVF was evaluated and found to be safe and acceptable in the MTN-007 rectal safety study [10] and had a similar profile in the CHARM-01 study. Despite the osmolality of the RF gel being half of the RGVF TFV gel (479 versus 846 mOsmol/kg, respectively), there was no difference in their respective safety profiles.

All three formulations were acceptable to the study participants. Of note, the VF TFV gel phase of dosing in CHARM-01 only included exposure to one dose of the VF TFV gel preceded by six daily doses of a HEC placebo gel. This likely explains why exposure to the VF TFV gel was more acceptable than the seven doses of VF TFV gel participants received in the RMP-02/MTN-006 study [7]. In the context of the CHARM-01 protocol, acceptability was based upon the assessment of a series of product characteristics that might be considered as potential barriers to use of these products such as taste, odor, and smell. In previous rectal and vaginal microbicide studies, including the RMP-02/MTN-006 study, acceptability has been assessed only on the basis of intentionality for future use of the product [24].

As with previous RM studies, the CHARM-01 study explored the potential impact of product administration on mucosal safety as assessed by histology, flow cytometry of gastrointestinal derived T cells, and rectal microflora. There were no histological changes noted in the study. There was a significant increase in CD3+/CD45+ T lymphocytes associated with 7 day exposure to the RF TFV gel. However, there were no significant increases in HIV-1 target cells defined by CD4+, CCR5+, or CXCR4+ phenotypes nor were there any significant changes in activation phenotype defined by CD69+, CD38+, or HLA-DR+ phenotypes. In the absence of such changes, it is unclear whether the increase in CD3+/CD45+ T lymphocytes associated with exposure to the RF gel might still be associated with a quantitative increase in HIV-1 target cells such as CD4+/CCR5+ T cells. Another limitation of this study is that we only characterized T cell phenotypes and in future it would be worthwhile broadening the flow panel to include other cells of myeloid lineage such as monocytes and macrophages that might play an important role in the mucosal response to HIV infection and that might be impacted by exposure to microbicide candidates. Flow cytometric analysis of gut-associated lymphoid tissue (GALT) T cell populations may be more useful in evaluating products like the CCR5 antagonist maraviroc that have been associated with changes in activation phenotype and CCR5 expression in GALT [25]. Such studies are currently ongoing in the HPTN-069 study of oral PrEP (ClinicalTrials.gov Identifier: NCT01505114) and will be used in the CHARM-03 study which will evaluate rectal and oral maraviroc products. Modest, but significant changes in rectal microflora were noted for five of the 24 organisms evaluated using semi-quantitative culture techniques. It is uncertain whether these changes could be associated with exposure to the TFV gels. However, it is known that zidovudine has antibacterial activity [26]. The clinical significance of these changes is uncertain and may be better understood after longer term studies and/or the use of newer technologies to characterize and quantify the rectal microbiome in microbicide trials [27]. An alternative approach to the assessment of mucosal safety is to incorporate systems biology to characterize the mucosal transcriptome and proteome before and after microbicide exposure. Using this approach, we have previously documented significant changes in rectal mucosal biology associated with TFV exposure including significant changes in mitochondrial function [10]. Similar evaluations are ongoing with samples from CHARM-01 participants and will be reported separately.

The compartmental PK data from CHARM-01 are similar to PK data generated in the RMP-02/MTN-006 study [7, 28]): rectal exposure to TFV gels is associated with minimal systemic exposure, lack of drug detection in PBMCs, high concentrations in rectal tissue/fluid, and detection in vaginal fluid. MMC TFV-DP trended toward ~2-fold greater concentrations following RF when compared to RGVF. Otherwise, there were no PK differences between these two products.

Single dose VF PK values cannot be fairly compared to the drug accumulation in steady-state RF and RGVF PK values after 7 doses. For example, based on our single dose VF PK data and the long TFV and TFV-DP half-life within most of the matrices tested [29], accumulation of TFV and TFV-DP after 7 daily VF doses would match or exceed the concentrations seen with the RF and RGVF products in this study.

The *ex vivo* HIV-1 tissue biopsy challenge assay has become an important component in the evaluation of candidate microbicides [30] and has been used to generate preliminary efficacy data in several RM studies [28, 31]. In the CHARM-01 study, all three formulations were associated with *ex vivo* viral suppression in colorectal tissue are these data are in keeping with previous Phase 1 RM studies where the use of both VF TFV gel and UC781 was associated with significant inhibition of explant infection [7,12]. These findings are impressive, especially for the VF as only one dose of active product was delivered *in vivo* with tissues acquired within 15–45 minutes following product insertion and it is known that the HEC gel used in the VF gel dosing phase does not have activity in the explant challenge model [12]. These data therefore give significant encouragement to the possibility that RM could be used in a pericoital fashion.

PK/PD modeling has been used to characterize the relationship between systemic and local drug concentrations and the ability to inhibit *ex vivo* viral replication in colorectal tissue [28,31]. PK/PD modeling can also be used to help define therapeutic/pharmacokinetic targets for microbicide development [28]. PK/PD data from the CHARM-01 study demonstrated a significant negative correlation between increasing concentrations of TFV and decreasing levels of *ex vivo* viral replication in colorectal tissue. Strikingly, the relationship was significant for all PK matrices studied (plasma, rectal tissue (TFV and TFV-DP), rectal tissue-derived MMC (TFV-DP), rectal fluid (TFV)).

The primary goal of the CHARM-01 study was to compare three different formulations of TFV gel and to determine whether there were significant differences in safety, acceptability, PK, or PD parameters that might facilitate deciding which product should be advanced into late stage development as a RM. Given the unacceptable rectal safety profile of the VF TFV gel, documented in the RMP-02/MTN-006 study, it is clear that this is not a viable product to develop further as a RM The real decision is whether CHARM-01 allows us to choose between the RF and RGVF formulations of TFV gel. The CHARM-01 PK data do suggest that the RF formulation may deliver higher local concentrations of TFV-DP to the rectal mucosa than the RGVF formulation, although this did not reach significance. This is the only discriminating parameter between the RF and RGVF TFV gels in the CHARM-01 study and may be insufficient to displace the RGVF TFV gel that is currently being evaluated in the MTN-017 study, an International Phase 2 expanded safety study being conducted in the United States, Peru, Thailand, and South Africa. The results of the MTN-017 study (expected in early 2016), with approximately 192 participants, eight week periods of exposure to daily or pericoital RGVF TFV gel, as well as a PK/PD substudy of 36 participants, will have a critical role in defining the future for the RGVF TFV gel as a candidate RM for Phase 3 safety and effectiveness trials. Certainly, with increasing rates of HIV infection in MSM and transgender women [2, 32] there is an urgent need to develop new approaches for the prevention of HIV infection in these highly vulnerable populations.

## Supporting Information

S1 CONSORT Checklist (DOC)

S1 Fig Gating strategy for CHARM-01 flow cytometry panels. (DOCX)

S2 Fig Plasma TFV kinetics. (DOCX)

S3 Fig Complete PK/PD relationships. (DOCX)

S1 Protocol (PDF)

S1 Table Complete flow cytometry data. (DOCX)

S2 Table Complete anaerobic microflora data. (DOCX)

S3 Table Complete facultative microflora data. (DOCX)
