# Supplementary figures and images for: A Phase 1 Randomized, Open Label, Rectal Safety, Acceptability, Pharmacokinetic, and Pharmacodynamic Study of Three Formulations of Tenofovir 1% Gel (the CHARM-01 Study)
Source: PLoS One. 2015 May 5;10(5):e0125363. doi: 10.1371/journal.pone.0125363 (PMC4420274; doi:10.1371/journal.pone.0125363)

Supplementary Figure 1 Plasma TFV kinetics


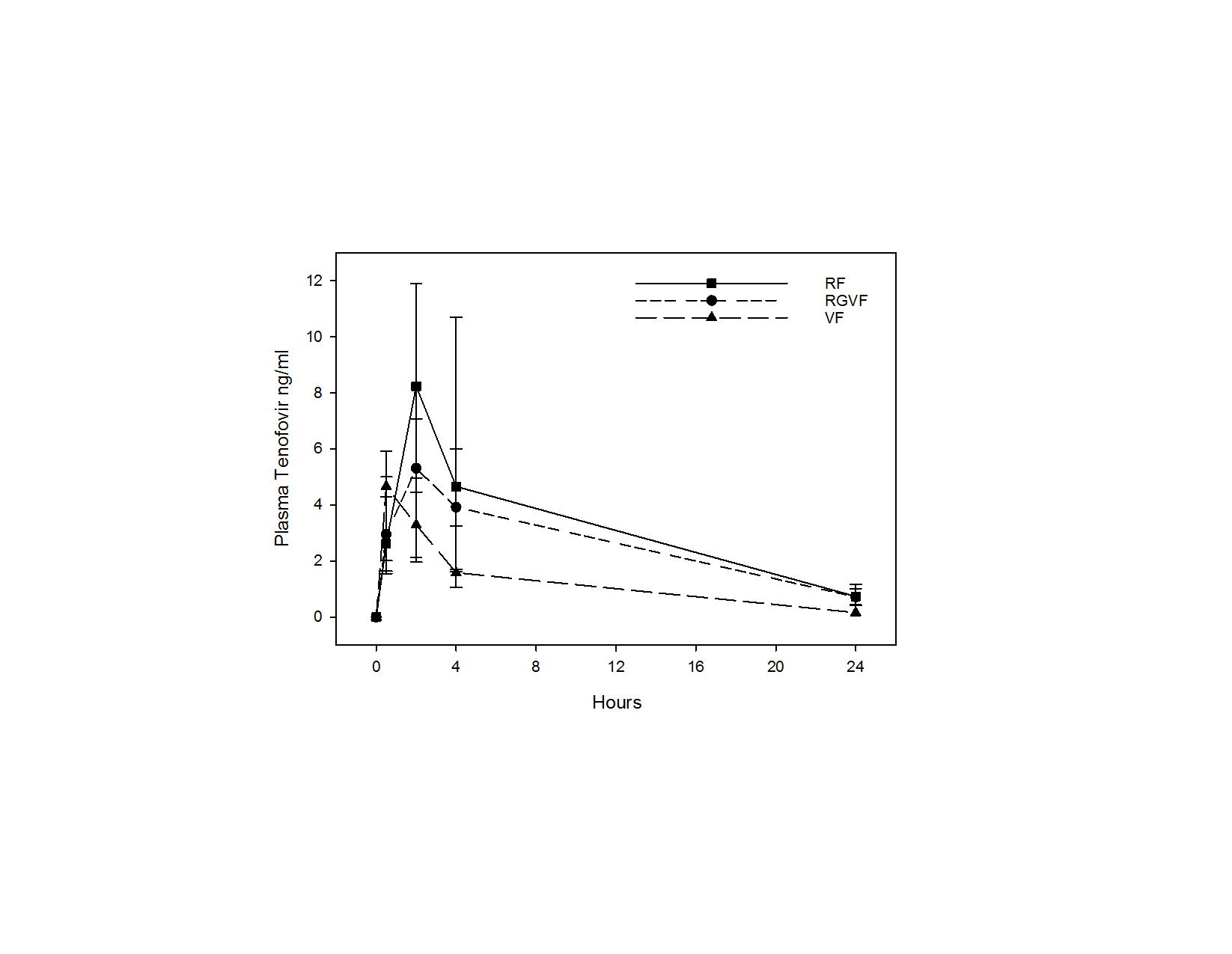

Supplement: S1 Fig — (DOCX) [file pone.0125363.s002.docx]
