# Supplementary material for: A Phase 1 Randomized, Open Label, Rectal Safety, Acceptability, Pharmacokinetic, and Pharmacodynamic Study of Three Formulations of Tenofovir 1% Gel (the CHARM-01 Study)
Source: PLoS One. 2015 May 5;10(5):e0125363. doi: 10.1371/journal.pone.0125363 (PMC4420274; doi:10.1371/journal.pone.0125363)
Supplement: S1 Protocol — (PDF) [file pone.0125363.s005.pdf]

CHARM-01

A Randomized, Double Blind Phase 1 Safety, Acceptability, and Pharmacokinetic Study Comparing Three Formulations of Tenofovir 1% Gel Administered Rectally to HIV-1 Seronegative Adults

Funded by:
Division of AIDS, US National Institute of Allergy and Infectious Diseases
US National Institutes of Health

DAIDS Protocol Number: 11836

IND Holder and Sponsor:
Ian McGowan MD, PhD, FRCP
University of Pittsburgh School of Medicine

This protocol will be performed under the following IND#: ***113,620***

Program Grant #: AI082637

Program Chair: Ian McGowan, MD, PhD, FRCP

Protocol Chair: Peter A. Anton, MD

Version 2.0

12 December 2011

CHARM-01

TABLE OF CONTENTS

LIST OF ABBREVIATIONS AND ACRONYMS vii

PROTOCOL TEAM ROSTER x

INVESTIGATOR SIGNATURE FORM xiii

PROTOCOL SUMMARY xiv

1 KEY ROLES 1

1.1 Protocol Identification 1
1.2 Funding Agency, Sponsor, and Monitor Identification 1
1.3 Medical Officer 1
1.4 Site Investigators 1
1.5 Clinical Laboratories 2
1.6 Research Laboratories 2
1.7 Data Center 2
1.8 Study Operations 2

2 INTRODUCTION 3

2.1 Background of Microbicide Research and Study Rationale 3
2.2 Background and Rationale of Assessments 5
2.2.1 Pharmacokinetics 6
2.2.2 Mucosal Immunotoxicity 8
2.2.3 Ex vivo efficacy in the colorectal explant model 11
2.2.4 Experience and Safety in Colorectal Biopsies for Data Assessments 11
2.2.5 Acceptability Assessments 12
2.3 Description of Study Products 15
2.3.1 Tenofovir 1% Gel 15
2.3.2 Universal HEC Placebo Gel 15
2.3.3 Mechanisms of Action 15
2.3.4 Strength of Study Products to be Evaluated in the Study 15
2.3.5 In vitro and Ex vivo Studies 16
2.4 Animal Studies 19
2.4.1 Vaginal Formulation (VF) of Tenofovir 1% Gel 20
2.4.2 Reduced Glycerin Vaginal Formulation (RGVF) of Tenofovir 1% Gel 24
2.4.3 Rectal Formulation (RF) of Tenofovir 1% Gel 25
2.4.4 Universal HEC Placebo Gel 26
2.4.5 Summary 28
2.5 Human Studies 28
2.5.1 Pharmacokinetics 28
2.5.2 Safety 30
2.5.3 Drug Resistance 35
2.5.4 Other Studies of Tenofovir for HIV Prevention 35
2.6 Justification of Dosing 37
2.7 Justification of Sampling Time Points 37

3 OBJECTIVES 38

3.1 Primary Objectives 38

3.2 Secondary Objectives ... 38
3.3 Exploratory Objectives ... 38

4 STUDY DESIGN ... 38
4.1 Identification of Study Design ... 38
4.2 Summary of Study Endpoints ... 41
4.2.1 Primary Endpoints ... 41
4.2.2 Secondary Endpoints ... 41
4.2.3 Exploratory Endpoints ... 41
4.3 Description of Study Population ... 41
4.4 Time to Complete Accrual ... 42
4.5 Expected Duration of Participation ... 42

5 STUDY POPULATION ... 42
5.1 Selection of Study Population ... 42
5.2 Recruitment ... 42
5.3 Retention ... 42
5.4 Inclusion Criteria ... 43
5.5 Exclusion Criteria ... 44

6 STUDY PRODUCTS ... 46
6.1 Regimen ... 46
6.2 Administration ... 46
6.3 Study Product Formulation ... 46
6.3.1 Vaginal Formulation ... 46
6.3.2 Reduced-Glycerin Vaginal Formulation ... 46
6.3.3 Rectal Specific Formulation ... 47
6.3.4 Universal HEC Placebo Gel Formulation ... 47
6.4 Study Product Supply and Accountability ... 48
6.4.1 Study Product Supply ... 48
6.4.2 Study Product Receipt ... 48
6.4.3 Storage ... 48
6.4.4 Dispensing ... 49
6.4.5 Retrieval of Unused Product ... 49
6.4.6 Accountability ... 49
6.5 Adherence ... 49
6.5.1 Evaluation of Adherence ... 49
6.6 Concomitant Medications and Procedures ... 50
6.7 Prohibited Medications and Procedures ... 50
6.8 Required Medications and Procedures ... 50

7 STUDY PROCEDURES ... 51
7.1 Visit 1: Screening ... 51
7.2 Visit 2: Enrollment/Baseline ... 52
7.3 Visits 3, 6, 9: First Dose of Study Product ... 53
7.4 Take-Home Periods of 5 Doses of Study Product ... 54
7.5 Visit 4, 7, 10: Seventh Dose of Study Product ... 55
7.6 Visit 5, 8, 11: Specimen Sampling 24-hr Post Seventh Dose ... 55
7.7 Exit: Termination Phone Call/Visit ... 56
7.8 Interim Contacts and Visits ... 56
7.9 Participants Who Become Infected with HIV ... 57
7.10 Participants Who Become Pregnant ... 57

7.11 Participants Who Withdraw or Are Withdrawn from the Study 58
7.12 Final Contact 58
7.13 Clinical Evaluations and Procedures 59
7.14 Laboratory Evaluations 60
7.14.1 Clinical Laboratory Testing 60
7.14.2 Research Laboratory Testing 60
7.15 Specimen Collection, Handling, and Processing 61
7.16 Storage of Specimens for Future Use 61
7.17 Biohazard Containment 61
7.18 Behavioral measures 62

8 ASSESSMENT OF SAFETY 63
8.1 Safety Monitoring 63
8.2 Clinical Data Safety Review 63
8.3 Adverse Event Definitions and Reporting Requirements 64
8.3.1 Adverse Events 64
8.3.2 Serious Adverse Events 65
8.3.3 Adverse Event Relationship to Study Product 65
8.4 Expedited Adverse Event (EAE) Reporting Requirements 66
8.5 Reporting of Adverse Reactions to the Responsible IRBs 67
8.6 Pregnancy and Pregnancy Outcomes 67
8.7 Social Harms Reporting 68
8.8 Withdrawal of Subjects Due to Adverse Events 68

9 CLINICAL MANAGEMENT 69
9.1 Grading System 69
9.2 Dose Modification 69
9.3 Discontinuation of Study Product(s) in the Presence of Toxicity 69
9.4 General Criteria for Discontinuation of Study Product(s) 70
9.5 Management of Specific Adverse Events 70
9.5.1 Hemorrhage Following Rectal Mucosal Biopsy 70
9.5.2 Infection Following Rectal Mucosal Biopsy 70
9.5.3 Perforation of Rectum Following Rectal Mucosal Biopsy 70
9.6 Criteria for Early Termination of Study Participation 70

10 STATISTICAL CONSIDERATIONS 71
10.1 Overview and Summary of Design 71
10.2 Study Endpoints 71
10.2.1 Primary Endpoints 71
10.2.2 Secondary Endpoints 72
10.2.3 Exploratory Endpoints 72
10.3 Study Hypotheses 72
10.4 Accrual and Sample Size 73
10.5 Randomization and Blinding 73
10.6 Blinding/Unblinding 73
10.7 Data Analysis 74
10.7.1 Primary Analysis on Safety 74
10.7.2 Primary Analysis on Acceptability 75
10.7.3 Primary Analysis on Pharmacokinetics 76
10.7.4 Secondary Analysis on Mucosal Immunotoxicity 76
10.7.5 Exploratory Analysis on Ex Vivo Efficacy 78
10.7.6 Missing Data 79

11 DATA HANDLING AND RECORDKEEPING 79
11.1 Data Management Responsibilities 79
11.2 Source Documents and Access to Source Data/Documents 79
11.3 Quality Control and Quality Assurance 80
11.4 Study Coordination 80
12 CLINICAL SITE MONITORING 81
13 HUMAN SUBJECTS PROTECTIONS 81
13.1 Institutional Review Boards 82
13.2 Protocol Registration 82
13.3 Risk-Benefit Statement 83
13.3.1 Risks 83
13.3.2 Benefits 87
13.4 Informed Consent Process 87
13.5 Participant Confidentiality 88
13.6 Special Populations 89
13.6.1 Pregnant Women 89
13.6.2 Children 89
13.7 Compensation 90
13.8 Communicable Disease Reporting 90
13.9 Access to HIV-related Care 90
13.9.1 HIV Counseling 90
13.9.2 Care for Participants Identified as HIV-infected 90
13.10 Study Discontinuation 90
14 PUBLICATION POLICY 90
15 LIST OF APPENDICES 91
16 REFERENCES 92
APPENDIX I: SCHEDULE OF STUDY VISITS AND EVALUATIONS 99
APPENDIX II: HIV TESTING ALGORITHM 101
APPENDIX III: TOXICITY TABLES 102
APPENDIX IV: HISTOPATHOLOGY SCORING SYSTEM 103
APPENDIX V: SAMPLE INFORMED CONSENT FORM 104
APPENDIX VI: SAMPLE INFORMED CONSENT FORM (SAMPLE STORAGE) 121

TABLES AND FIGURES

Figure 1: Study Schema xv
Figure 2: Study Regimen xvii
Figure 3: Histological sections of healthy rectosigmoid biopsies from routine colonoscopy. 4
Figure 4: Conceptual Model of a 6-Compartment PK Model Linked to PD Outcomes 7

Table 1: Summary of RAI in Surveys of Sexual Behavior 3
Table 2: Relative Risk of Unprotected Sexual Practice in HIV Transmission 3
Table 3: PK and PD- Advantages/Disadvantages of Sampling by Compartment 8
Table 4: Summary of Biological Specimen Collections 14
Table 5: Rectal Irritation Study in New Zealand White Rabbits 20
Table 6: Other Studies of Tenofovir for HIV Prevention 35
Table 7: PrEP Studies 36
Table 8: Comparison of Tenofovir 1% Gel Formulations 47
Table 9: Visit 1 (Screening) 52
Table 10: Visit 2 (Enrollment/Baseline) 53
Table 11: Visits 3, 6, 9 54
Table 12: Visits 4, 7, 10 55
Table 13: Visits 5, 8, 11 56
Table 14: Exit – Termination Phone Call/ Visit 56
Table 15: Interim Contact and Visits 57
Table 16: Early Termination Visit 58
Table 17: Laboratory Assay Responsibilities 60
Table 18: Probability of events for selected true rates of AEs 74
Table 19: Exact 95% confidence bounds for true rate of AEs (n=18) 75

CHARM-01

LIST OF ABBREVIATIONS AND ACRONYMS

3TC	lamivudine
ABC	abacavir
AE	adverse event
AIDS	Acquired Immunodeficiency Syndrome
ALT	alanine transaminase
ANCOVA	analysis of covariance
ANOVA	analysis of variance
APV	amprenavir
ARV	antiretroviral
ASTM	American Society for Testing and Materials
AST	aspartate aminotransferase
AUC	area under the curve
BV	bacterial vaginosis
BUN	blood urea nitrogen
CAPRISA	Centre for the AIDS Programme of Research in South Africa
CBC	complete blood count
CCR5	chemokine receptor 5
CDC	Centers for Disease Control and Prevention
CFR	Code of Federal Regulations
cGMP	current Good Manufacturing Practices
CHARM	Combination HIV Antiretroviral Rectal Microbicide
C_{max}	maximum serum concentration
C-PMPA	radio labeled tenofovir
CRF	case report form
CRS	clinical research site
CT	*Chlamydia trachomatis*
CVL	cervicovaginal lavage
CXCR4	CXC chemokine receptor 4
d4T	stavudine
DAIDS	Division of AIDS
DC	dendric cells
ddC	zalcitabine
ddI	didanosine
DLV	delavirdine
DMPA	depot medroxyprogesterone acetate
DNA	deoxyribonucleic acid
DP	diphosphate
DSMB	Data and Safety Monitoring Board
EFV	efavirenz
ELISA	Enzyme-Linked Immunosorbent Assay
EAE	Expedited Adverse Event
FDA	Food and Drug Administration

FHI	Family Health International
FTC	emtricitabine
GC	*Gonorrhoeae*
GCP	Good Clinical Practices
GLP	Good Laboratory Practices
GRFT	griffithsin
HEC	hydroxyethylcellulose
HHS	Health and Human Services
HIV	Human Immunodeficiency Virus
HPTN	HIV Prevention Trials Network
HSV	herpes simplex virus
IDV	indinavir
IND	investigational new drug
IoR	Investigator of Record
IPCP	Integrated Pre-Clinical/Clinical Program
iPrEx	Pre-Exposure Prophylaxis Initiative
IRB	Institutional Review Board
IUD	intrauterine device
IV	intravenous
JHU	Johns Hopkins University
MID	median rectal infectious doses
MIP	macrophage inflammatory protein
MMC	mucosal mononuclear cells
mRNA	messenger ribonucleic acid
MO	Medical Officer (Division of AIDS)
MQAP-NICHD	Microbicide Quality Assurance Program-National Institute of Child Health and Human Development
MSM	Men who have sex with men
MTN	Microbicide Trials Network
MTT	[1-(4,5-dimethylthiazol-2-yl)-3,5-diphenylformazan]
MWRIF	Magee-Womens Research Institute and Foundation
N-9	Nonoxynol-9
NAAT	Nucleic acid amplification test
NFV	nelfinavir
NIAID	National Institute of Allergy and Infectious Diseases
NICHD	National Institute of Child Health and Human Development
NIH	(United States) National Institutes of Health
NOAEL	no observed adverse effect level
NNRTI	non-nucleoside reverse transcriptase inhibitor
NRTI	nucleoside reverse transcriptase inhibitor
NVP	nevirapine
OHRP	Office for Human Research Protections
PBMC	peripheral blood mononuclear cells
PCR	polymerase chain reaction
PD	pharmacodynamic(s)
PK	pharmacokinetic(s)

PMPA	9-[(R)-2-(phosphonomethoxy)propyl] adenine monohydrate
PTID	Participant Identification Number
PRN	as needed
PRO	Protocol Registration Office
QD	daily
RANTES	Regulated on activation normal T cell expressed and secreted
RAI	receptive anal intercourse (refers to coitus only, does not include manual stimulation or the use of sex toys or purgatives)
RF	rectal formulation
RGVF	reduced glycerin vaginal formulation
RMP	Rectal Microbicide Program
RPR	rapid plasma reagin
RSC	Regulatory Support Center
RT	reverse transcriptase
RTV	ritonavir
SAE	Serious Adverse Event
SHIV	Simian/Human Immunodeficiency Virus
SIV	Simian Immunodeficiency Virus
SMC	Safety Monitoring Committee
SQV	saquinavir
SSP	study-specific procedures
STI	sexually transmitted infection
SUSAR	Suspected and unexpected serious adverse reactions
TDF	tenofovir disoproxil fumarate (oral tenofovir)
TER	transepithelial resistance
TERIS	Teratogen Information System
ToO	Transfer of Sponsor Obligations
UA	urinalysis
UAI	unprotected anal intercourse
UCLA	University of California, Los Angeles
ULN	upper limit of normal
UTI	urinary tract infection
VF	vaginal formulation
VI	virus isolation
VM	vaginal microbicide
VOICE	Vaginal and Oral Interventions to Control the Epidemic
ZDV	zidovudine

CHARM-01

PROTOCOL TEAM ROSTER

Peter Anton, MD<br>CHARM Project 3 Leader<br>Protocol Chair and University of California at Los Angeles (UCLA), Study Site Investigator<br>Investigator of Record for UCLA CRS<br>UCLA Center for Prevention Research<br>675 Charles E. Young Dr., MRL 2734<br>Los Angeles, CA 90095-7019<br><br>Phone: 310-206-5797<br>Fax: 310-206-8824<br>Email: PAnton@mednet.ucla.edu	Marianne Morse Callahan<br>Deputy Director, Clinical<br>CONRAD<br>1911 North Fort Myer Drive, Suite 900<br>Arlington, VA 22209 USA<br><br>Phone: 703-276-3915<br>Fax: 703-524-4770<br>Email: mcallahan@conrad.org
Ross D. Cranston, MD, FRCP<br>Magee Womens Research Institute (MWRI), Study Site Investigator<br>Investigator of Record for MWRI-University of Pittsburgh CRS<br>Division of Infectious Disease<br>University of Pittsburgh Medical Center<br>3520 Fifth Avenue<br>Keystone Building, Suite 510<br>Pittsburgh, PA 15213 USA<br><br>Phone: 412-383-2054<br>Fax: 412-383-2900<br>Email: rdc27@pitt.edu	Robert Dennis, PhD<br>Data Center, Director<br>UCLA Computing Technologies Research Lab<br>62-073 CHS, 10833 Le Conte Ave<br>Los Angeles, CA 90095 USA<br><br>Phone: 310-206-6556<br>Email: buddy@ucla.edu
Julie Elliot<br>Laboratory Manager<br>UCLA Center for Prevention Research<br>675 Charles E Young Dr South, MRL 1529<br>Los Angeles, CA 90095 USA<br><br>Phone: 310-794-7195<br>Fax: 310-267-0289<br>Email: jelliott@mednet.ucla.edu	Henry L. Gabelnick, PhD<br>CONRAD<br>1911 North Fort Myer Drive, Suite 900<br>Arlington, VA 22209 USA<br><br>Phone: 703-524-4744<br>Fax: 703-524-4770<br>Email: hgabelnick@conrad.org
Craig W. Hendrix, MD<br>Director, Drug Development Unit<br>Division of Clinical Pharmacology<br>The Johns Hopkins Hospital<br>600 North Wolfe Street, Harvey 502<br>Baltimore, MD 21287 USA<br><br>Phone: 410-955-9707<br>Fax: 410-955-9708<br>Email: cwhendrix@jhmi.edu	Cindy Jacobson, PharmD<br>Director of Pharmacy Affairs<br>MWRI<br>204 Craft Avenue<br>Pittsburgh, PA 15213 USA<br><br>Phone: 412-641-8913<br>Fax: 412-641-6170<br>Email: cjacobson@mail.magee.edu

Laura Janocko<br>MWRI McGowan Lab Manager<br>MWRI<br>204 Craft Ave, Room A540<br>Pittsburgh, PA 15213 USA<br><br>Phone: 412-641-7547<br>E-mail: janockole@upmc.edu	Kaleab Z. Abebe, PhD<br>Statistician<br>Center for Research on Health Care Data Center<br>University of Pittsburgh School of Medicine<br>200 Meyran Ave., Suite 300<br>Pittsburgh, PA 15213<br><br>Phone: 412-246-6931<br>Fax: 412-586-9672<br>Email: kza3@pitt.edu
Elena Khanukhova<br>Clinical Research Manager<br>CHARM Regulatory Core<br>UCLA Center for Prevention Research<br>10940 Wilshire Blvd., Suite 900<br>Los Angeles, CA 90024 USA<br><br>Phone: 310-206-3633<br>Fax: 310-794-2829<br>Email: ekhanukhova@mednet.ucla.edu	Cherlynn Mathias, RN, BSN<br>Program Officer/Nurse Consultant<br>Prevention Science Program, Division of AIDS<br>6700 B Rockledge Drive, Room 5110<br>Bethesda, MD 20817 USA<br><br>Telephone: 301-402-0142<br>Email: cmathias@niaid.nih.gov
Christine Mauck, MD, MPH<br>Consultant<br>CKM Consulting, LLC<br>5 Hidden Ponds Ct.<br>Gaithersburg, MD 20878 USA<br><br>Telephone: 301-325-5439<br>Email: cmauck1@gmail.com	Ian McGowan, MD, PhD, FRCP<br>CHARM Program Director<br>MWRI<br>204 Craft Avenue, Room B505<br>Pittsburgh, PA 15213 USA<br><br>Phone: 412-641-4710<br>Fax: 412-641-6170<br>Email: mcgowanim@mail.magee.edu
Lorna Rabe, BS, M(ASCP)<br>Laboratory Manager<br>MWRI<br>204 Craft Avenue, Room A530<br>Pittsburgh, PA 15213 USA<br><br>Phone: 412-641-6042<br>Fax: 412-641-6170<br>Email: rabelk@upmc.edu	Lisa C. Rohan, PhD<br>CHARM Formulation Core Director<br>MWRI<br>204 Craft Avenue<br>Pittsburgh, PA 15213 USA<br><br>Telephone: 412-641-6108<br>Fax: 412-641-6170<br>Email: rsilcr@mwri.magee.edu
Janet Schafer<br>Manager, Clinical Trials/ Regulatory Affairs<br>CONRAD<br>1911 Fort Myer Drive, Suite 900<br>Arlington, VA 22209 USA<br><br>Phone: 703-276-4034<br>Fax: 703-524-4770<br>Email: jschafer@conrad.org	Jill Schwartz, MD<br>Medical Director<br>CONRAD<br>1911 Fort Myer Drive, Suite 900<br>Arlington, VA 22209 USA<br><br>Phone: 703-276-3913<br>Fax: 703-524-4770<br>Email: jschwartz@conrad.org

Hans Spiegel, MD<br>DAIDS Medical Officer<br>NIAID/ Division of AIDS<br>6700A Rockledge Drive, Room 42A 123<br>Bethesda, MD 20817 USA<br><br>Phone: 301-896-4071<br>Fax: 301-896-0315<br>Email: hans.spiegel@nih.gov	Ron Stall, PhD, MPH<br>Behavioral research representative<br>Graduate School of Public Health,<br>University of Pittsburgh<br>130 DeSoto Street, 208 Parran Hall<br>Pittsburgh, PA 15261<br><br>Phone: 412-624-7933<br>Fax: 412-648-5975<br>Email: rstall@pitt.edu
Jim A. Turpin, PhD<br>DAIDS Program Officer<br>Microbicide Research Branch<br>Division of AIDS<br>6700 B Rockledge Drive, Room 4118<br>Bethesda, MD 20892 USA<br><br>Phone: 301-451-2732<br>Fax: 301-496-8530<br>Email: JTurpin@niaid.nih.gov	

CHARM-01

A Randomized, Double Blind Phase 1 Safety, Acceptability, and Pharmacokinetic Study Comparing Three Formulations of Tenofovir 1% Gel Administered Rectally to HIV-1 Seronegative Adults

INVESTIGATOR SIGNATURE FORM

Version 2.0
12 December 2011

Funded by:
Division of AIDS, US National Institute of Allergy and Infectious Diseases
US National Institutes of Health

IND Holder and Sponsor:
Ian McGowan MD PhD, University of Pittsburgh School of Medicine

I, the Investigator of Record, agree to conduct this study in full accordance with the provisions of this protocol. I will comply with all requirements regarding the obligations of investigators as outlined in the Statement of Investigator (Form FDA 1572), which I have also signed. I agree to maintain all study documentation for at least two years following the date of marketing approval for the study product for the indication in which it was studied. If no marketing application is filed, or if the application is not approved, the records will be retained for two years after the investigation is discontinued, the US Food and Drug Administration is notified, and the site's final Financial Status Report is filed with the National Institutes of Health (NIH). Publication of the results of this study will be governed by Combination HIV Antiretroviral Rectal Microbicide (CHARM) Integrated Pre-clinical/Clinical Program (IPCP) and those of NIH. Any presentation, abstract, or manuscript will be made available to CHARM's Scientific Review Committee as well as Division of AIDS (DAIDS) for review prior to submission.

I have read and understand the information in the Investigator's Brochure(s), including the potential risks and side effects of the products under investigation, and will ensure that all associates, colleagues, and employees assisting in the conduct of the study are informed about the obligations incurred by their contribution to the study.

____
Name of Investigator of Record

______________________________ ______________________________
Signature of Investigator of Record Date

CHARM-01

A Randomized, Double Blind Phase 1 Safety, Acceptability, and Pharmacokinetic Study Comparing Three Formulations of Tenofovir 1% Gel Administered Rectally to HIV-1 Seronegative Adults

PROTOCOL SUMMARY

Short Title: CHARM-01

Clinical Phase: Phase 1

IND Holder and Sponsor: Ian McGowan, University of Pittsburgh School of Medicine

Study Site Investigators: Peter A. Anton, MD (UCLA) and Ross Cranston, MD, FRCP (MWRI-UPMC)

Sample Size: Approximately 18 total evaluable participants (~9 per site)

Study Population: HIV-negative men and women

Clinical Research Sites (CRS):

- University of California at Los Angeles (UCLA), Los Angeles, USA
- Magee-Womens Research Institute (MWRI) at University of Pittsburgh, Pittsburgh, USA

Study Design: A double-blinded, randomized, safety, acceptability, pharmacokinetic, and *ex vivo* efficacy study of 3 rectally-applied tenofovir-based microbicide formulations. Each participant will experience seven rectal exposures to the rectal-specific formulation (RF) and seven rectal exposures to the reduced glycerin vaginal formulation (RGVF) of tenofovir 1% gel, but only one exposure to the vaginal formulation (VF), which will be coupled with six preceding exposures to the Universal HEC Placebo Gel to balance out the VF study stage.

Figure 1: Study Schema

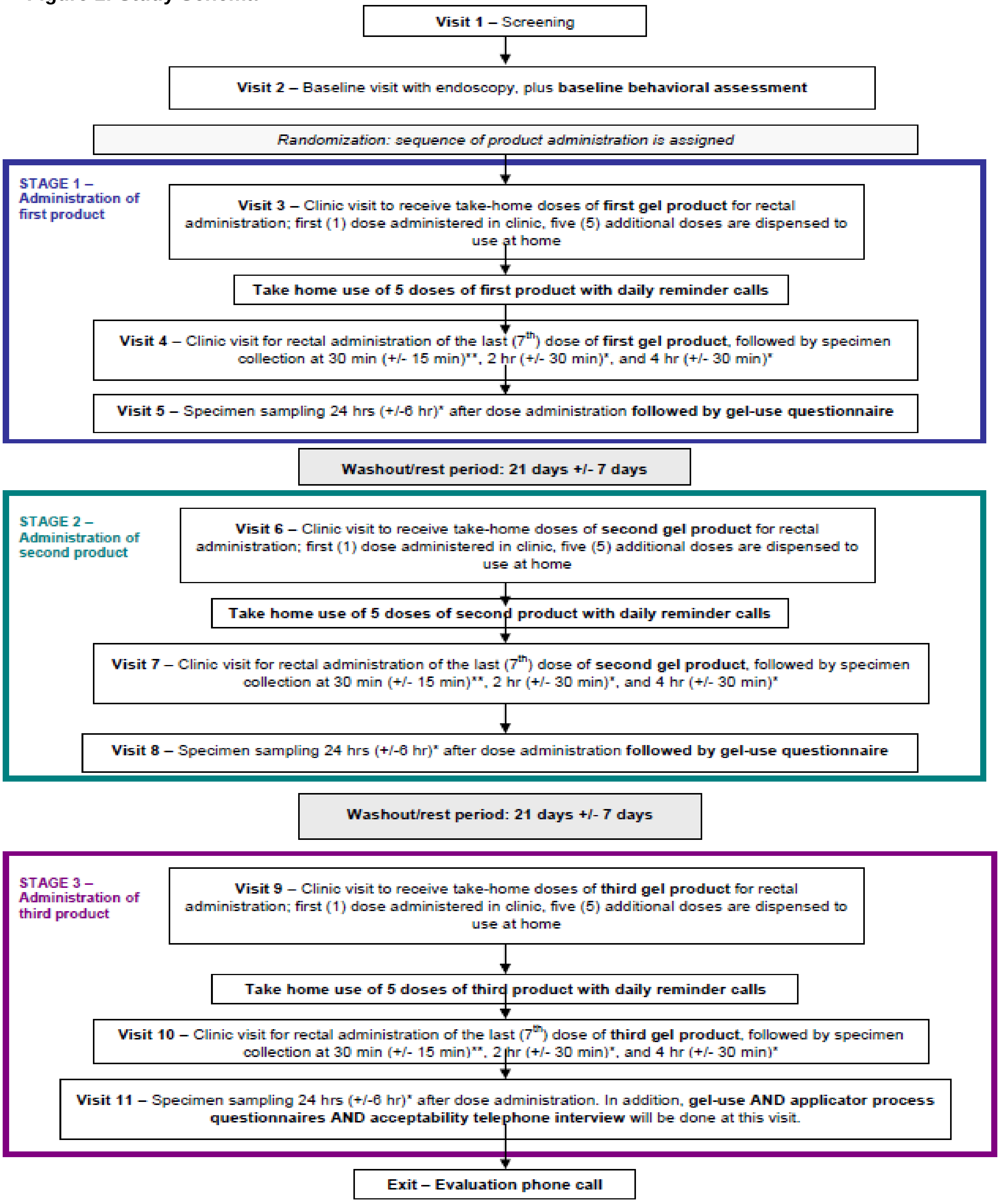

* Only blood and vaginal and rectal fluids are collected

** This time-point includes biopsy collection

Study Duration: Participant accrual will take approximately 6 months and each participant will be on study for approximately 3 months. The total duration of the study will be approximately 1 year.

Study Products: All formulations, except the Universal HEC Placebo Gel, contain tenofovir 1% gel (see Table 8 in section 6.3.3 for formulation details)

- Vaginal formulation (VF)
- Reduced glycerin vaginal formulation (RGVF)
- Rectal specific formulation (RF)

Study Regimen:

Figure 2: Study Regimen

Seven Doses of 1st Study Product	Post-dose Specimen Sampling	Washout/ Rest Period	Seven Doses of 2nd Study Product	Post-dose Specimen Sampling	Washout/ Rest Period	Seven Doses of 3rd Study Product	Post-dose Specimen Sampling
Rectal administration of study gel (first and last dose administered in clinic)	30 min*<br>2-hr<br>4-hr<br>24-hr	21 days +/- 7 days	Rectal administration of study gel (first and last dose administered in clinic)	30 min*<br>2-hr<br>4-hr<br>24-hr	21 days +/- 7 days	Rectal administration of study gel (first and last dose administered in clinic)	30 min*<br>2-hr<br>4-hr<br>24-hr

*Including biopsy collection via flexible sigmoidoscopy

Primary Objectives: Safety, Acceptability, and Pharmacokinetics

- To evaluate the safety of each tenofovir-based microbicide gel formulation when applied rectally
- To evaluate the acceptability of each tenofovir-based microbicide gel formulation when applied rectally
- To compare systemic and compartment pharmacokinetics among the three tenofovir-based microbicide gel formulations when applied rectally

Primary Endpoints:

Safety

- Grade 2 or higher clinical and laboratory adverse events as defined by the *Division of AIDS Table for Grading the Severity of Adult and Pediatric Adverse Events, Version 1.0, Dec 2004* and Addenda 1 and 3 (*Female Genital and Rectal Grading Tables for Use in Microbicide Studies*) to this table.

Acceptability

- Product attributes considered likely to challenge future sustained use by participants.

Pharmacokinetics

- Tenofovir concentrations
    - Plasma
    - Peripheral blood mononuclear cells (PBMC), intracellular
    - Rectal fluid
    - Vaginal fluid, in female participants
    - Rectal mucosal tissue homogenates
    - Rectal mucosal mononuclear cells (MMC)

- Tenofovir diphosphate concentrations

- PBMC
- Rectal mucosal tissue homogenates
- Rectal MMC

Secondary Objective: Mucosal Immunotoxicity

- To evaluate the mucosal immunotoxicity of each tenofovir-based microbicide gel formulation when applied rectally

Secondary Endpoints:

- Rectal microflora
- Rectal cytokines (secreted and messenger ribonucleic acid (mRNA))
- Rectal histology
- Rectal CD4 cell phenotype/activation

Exploratory Objective: *Ex Vivo* Efficacy

- To assess the preliminary (*ex vivo)* efficacy of each tenofovir-based microbicide gel formulation using biopsy explants after each product is applied rectally

Exploratory Endpoint:

- Changes in laboratory-applied HIV-1 p-24 levels in colorectal explant supernatant obtained from biopsies collected after each product is applied rectally

1 KEY ROLES

1.1 Protocol Identification

Protocol Title:	CHARM-01 – A randomized, double blind Phase 1 safety, acceptability, and pharmacokinetic study comparing three formulations of tenofovir 1% gel administered rectally to HIV-1 seronegative adults
Short Title:	CHARM-01
Date:	12 December 2011

1.2 Funding Agency, Sponsor, and Monitor Identification

Funding Agency:	DAIDS/National Institute of Allergy and Infectious Diseases (NIAID)/National Institutes of Health (NIH)<br>6700 B Rockledge Drive<br>Bethesda, MD 20892 USA
IND Holder and Sponsor:	Ian McGowan MD, PhD<br>University of Pittsburgh School of Medicine<br>204 Craft Ave, Room B505<br>Pittsburg, PA 15213 USA
Monitor:	Pharmaceutical Product Development, Inc. (PPD)<br>929 North Front Street<br>Wilmington, NC 28401-3331 USA

1.3 Medical Officer

DAIDS Medical Officer:	Hans Spiegel, MD<br>National Institute of Allergy and Infectious Diseases<br>Division of AIDS<br>6700 A Rockledge Drive, Room 42A 123<br>Bethesda, MD 20817 USA

1.4 Site Investigators

UCLA:	Peter Anton, MD
MWRI:	Ross Cranston, MD, FRCP

1.5 Clinical Laboratories

UCLA:	UCLA Clinical Laboratory<br>200 Medical Plaza, 1st Floor<br>Los Angeles, CA 90095 USA
MWRI:	Quest Diagnostics<br>875 Greentree Road<br>Pittsburgh, PA 15220 USA

1.6 Research Laboratories

UCLA:	UCLA Mucosal Immunology Core Laboratory:<br>675 Charles Young Dr, South<br>MRL Room 1529<br>Los Angeles, CA 90095-7208 USA
MWRI:	Magee-Womens Research Institute<br>McGowan Laboratory (Room A540)<br>Hillier Laboratory (Rooms A520, A530)<br>204 Craft Avenue<br>Pittsburgh, PA 15213 USA
Johns Hopkins:	Johns Hopkins University<br>Clinical Pharmacology Analytical Laboratory<br>600 N. Wolfe St./ Osler 523<br>Baltimore, MD 21287 USA

1.7 Data Center

Data Center:	UCLA Computing Technologies Research Lab<br>62-073 CHS, 10833 Le Conte Ave<br>Los Angeles, CA 90095 USA

1.8 Study Operations

Study Operations:	UCLA Center for Prevention Research<br>10940 Wilshire Blvd., Suite 900<br>Los Angeles, CA 90024 USA

2 INTRODUCTION

2.1 Background of Microbicide Research and Study Rationale

Microbicides are products that are designed to be applied to the vaginal or rectal mucosa with the intent of preventing or at least significantly reducing the acquisition of sexually transmitted infections (STIs) including Human Immunodeficiency Virus (HIV) [1]. The original impetus for vaginal microbicide development was to provide women with options for HIV prevention in settings where their partners were unwilling to use condoms [2]. Years later, the need to also develop a rectal microbicide (RM) became clear, given that a significant proportion of men do not consistently use condoms during anal sex with men or women [3, 4], and that there has consequently been little or no decline in the rates of new HIV infections, particularly in men who have sex with men (MSM) [5].

Furthermore, there is increasing epidemiological evidence that women as well as men in both the developed [4, 6, 7] and developing world [8-10] practice receptive anal intercourse (RAI) (Table 1) , and that a number of men in Sub Saharan Africa practice RAI while also having sexual relationships with women [11, 12].

Table 1: Summary of RAI in Surveys of Sexual Behavior

Population	Gender	N	Prevalence AI	Reference
MSM in EXPLORE study	Men	4295	48-54%	Koblin B et al. 2003 [13]
High risk women	Women	1,268	32%	Gross M et al. 2000 [14]
College students	Men and women	210	20%	Civic D 2000 [15]
US Survey (15-44 year olds)	Men and women	12, 571	35-40%	Mosher WD 2005 [6]
Californian residents	Men and women	3,545	6-8%	Erickson PI et al. 1995 [16]

Unprotected RAI is the sexual behavior with the highest per act risk of HIV acquisition (Table 2), conferring perhaps 10 to 20 times more risk than unprotected vaginal intercourse [17, 18].

Table 2: Relative Risk of Unprotected Sexual Practice in HIV Transmission

Behavior	Risk of HIV Infection	Reference
Oral Sex	0.0% (95% CI: 0,1.5)	Page-Shafer K. et al. 2002 [19]
Vaginal Sex	0.001 – 0.02% (95% CI: NA)	Kalichman S. et al. 2002 [20]
Anal Sex	0.25% (95% CI: 0.06,0.49)	Vittinghoff E. et al. 1999 [18]

Clearly, RMs should be seen as an important HIV prevention technology for all individuals who practice RAI, and not just for MSM.

The rectal columnar epithelium is fragile and extremely vulnerable to HIV-1 infection, in part due to the proximity of sub-epithelial stromal tissues that are densely populated with cells receptive to incident HIV-1 infection such as dendritic cells (DCs), macrophages and T-cells that express both CD4 and both HIV-1 co-receptors CCR5 and CXCR4 [21-23]. Although the mechanisms of viral uptake and infection across rectal mucosa are not fully established, such physiological and anatomical differences may explain why HIV is more readily transmitted across rectal than across the cervicovaginal genital epithelium (Figure 3).

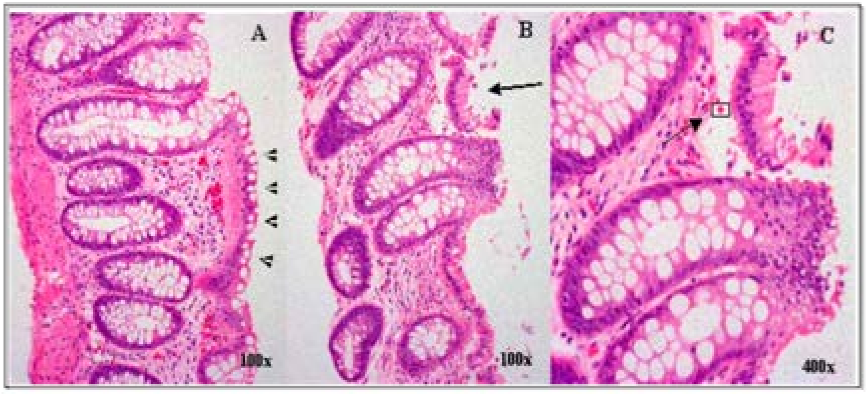

Figure 3: Histological sections of healthy rectosigmoid biopsies from routine colonoscopy. Panel A is uninjured with attached epithelia and prominent goblet cells (arrows). Panel B shows mild injury from colonoscope with epithelial sloughing and mucin depletion. Panel C is a high power view identifying a RBC (box) to emphasize the easy access of an HIV-sized particle.

Given the prevalence of RAI and the vulnerability of the rectal mucosa, it is important to develop microbicide gels that are optimized for rectal administration and to realize that once vaginal microbicides are licensed they are likely to be used in both the vaginal and rectal compartments. Thus establishing the safety of both vaginal and rectal microbicide formulations in the rectal compartment is essential.

There is increasing recognition within the HIV microbicide research communities that there needs to be a more comprehensive approach to the early stage development of candidate products. These intensive, exploratory trials should provide detailed information on pharmacokinetic (PK) and pharmacodynamic (PD) profile, product distribution, systemic and mucosal safety, and, when possible, a surrogate of product efficacy. Together, such data will allow the rational and cost-effective progression of products through the development pipeline. In addition, these data should prevent the premature cessation of late stage effectiveness studies due to lack of efficacy or, more troublingly, product safety issues [24, 25]. The National Institutes of Health (NIH) Division of AIDS (DAIDS) Integrated Pre-clinical/Clinical Program (IPCP) for HIV Topical Microbicides sponsors the Combination HIV Antiretroviral Rectal Microbicide (CHARM) Program.

The CHARM Program represents a careful response to the need for a more comprehensive approach to early stage development of candidate microbicide products. To this end, the CHARM Program has developed the CHARM-01 and CHARM-02 clinical protocols to further define the attributes of three microbicide tenofovir 1% gel

formulations with different osmolality when administered rectally: a vaginal formulation (VF, 3111 mOsmol/kg), a reduced-glycerin vaginal formulation (RGVF, 846 mOsmol/kg), and a rectal-specific formulation (RF, 479 mOsmol/kg). The clinical relevancy of these CHARM protocols derives from other observations and the just-completed RMP-02/MTN-006 trial of rectally-applied VF tenofovir 1% gel which is very hyperosmolar compared to RF and RGVF (see Table 8 and data below in Sections 2.5.1.1.2 and 2.5.2.1.2). In active response to a few gastrointestinal-related Grade 3 AEs (only during 7-day exposure) and less than desired acceptability of the current product used rectally, lower osmolar formulations have been brought to the clinical testing front quickly. The rectal formulation has the lowest osmolality (nearly iso-osmolar) of the three drug-containing products.

This protocol (CHARM-01) will focus on the pharmacokinetics (PK) and immunotoxicity of these gels when administered rectally. The companion protocol (CHARM-02) will focus on the (PK) and product distribution of these same products when administered rectally. In CHARM-01, PK profiles will be obtained in plasma, peripheral blood mononuclear cells (PBMC), rectal fluid, vaginal fluid in women participants, rectal tissue homogenates, and isolated rectal tissue mononuclear cells after three different gel formulations are rectally administered for seven days each. However, to address the Grade 3 AEs mentioned above that were experienced with the 7-day exposures of the VF during a previous study, the VF administration stage will actually consist of a single dose VF product and six days of a placebo product. The RF and RGVF will include 7 full product doses to be administered rectally. In contrast, CHARM-02 will only collect PK profile data from blood after same three gel formulations are rectally administered as single doses.

2.2 Background and Rationale of Assessments

The first rectal safety studies evaluated Nonoxynol-9 (N-9) gel. Tabet et al. described mild rectal histological changes in participants receiving up to 6 weeks of N-9 or placebo gel [26]. In contrast, marked epithelial exfoliation was seen after brief exposure to N-9 in studies by Phillips et al using rectal lavage and histology as endpoints [27, 28]. These contradictory results probably reflect the timing of sample collection. Epithelial reconstitution can occur within 1-8 hours after exposure to N-9 [28, 29]. In the Tabet study samples were collected up to 12 hours after N-9 exposure but only 15 minutes in the Phillips study. The implication of these early studies is that rectal safety should be assessed after acute (within 1 hour) and chronic (at least 7 days) product exposure.

Histology and/or rectal lavage studies can be helpful in documenting severe microbicide associated mucosal changes. However, there is increasing concern that repeated mucosal exposure to VM or RM could induce subtle immunological changes in the vaginal or rectal mucosa that might increase the risk of HIV transmission [30]. Increased expression of mucosal inflammatory cytokines could lead to recruitment of target cells to the local mucosa and these changes would probably not be detected using conventional histological techniques. As a consequence, it will be necessary to develop immunological biomarkers of microbicide safety.

A first step in this process is the characterization of the biological variability of putative mucosal safety biomarkers. Markers that demonstrate extreme variability will be unhelpful as safety biomarkers in microbicide studies. McGowan et al. recently published a study that investigated the biological variability of safety biomarkers in the intestinal mucosa. Intestinal biopsies were collected from 16 participants on three occasions over a 4 week period in the absence of any microbicide exposure [31]. Tissue was collected at 15 and 30 cm from the anal margin and evaluated for biological variability of a broad range of parameters including histology, mucosal cytokine gene expression, rectal immunoglobulins, and mucosal T cell phenotype. The study demonstrated that tissue from both sites was essentially equivalent and that the most stable parameters included mucosal cytokine expression and T cell phenotype. Both of these parameters could therefore have utility in the evaluation of potential microbicide toxicity within phase 1 rectal safety studies.

The first microbicide product to undergo phase 1 rectal safety assessment with this broader range of safety biomarkers was the non nucleoside reverse transcriptase inhibitor UC781. In the study (RMP-01), which was conducted at the University of California at Los Angeles, participants were screened to exclude anorectal STIs and then baseline mucosal samples were collected. After an approximately two week period to allow mucosal healing, the participants received a single dose of UC781. Within 30 minutes of microbicide exposure, the participants underwent mucosal assessment to evaluate acute mucosal responses to UC781. After a second recovery period, seven daily doses of UC781 were administered followed by final mucosal assessment. The range of safety parameters evaluated in the study included intestinal histology, rectal lavage for epithelial exfoliation, intestinal cytokine gene expression, mucosal mononuclear T cell phenotype, rectal immunoglobulins, and fecal calprotectin. A unique feature of this study was the evaluation of intestinal tissue explants, exposed to UC781 *in vivo*, to resist HIV infection *in vitro*. This design feature allowed for preliminary assessment of microbicide efficacy as well as safety before potentially proceeding to much larger clinical effectiveness studies. The data from RMP-01 demonstrated that UC781 was safe, well tolerated, did not induce any mucosal “immunotoxicity” and significantly inhibited explant infection with HIV [32]. CHARM-01 incorporates the same approach to assessment of mucosal immunotoxicity and explant infection as RMP-01.

2.2.1 Pharmacokinetics

For a microbicide to be effective it is critical that the product is present in the appropriate anatomical site, at the correct concentration, throughout the period of exposure to HIV infection. Consequently, the multicompartmental PK assessment included in CHARM-01 (and previously used in RMP-02/MTN-006) will generate important data that, in combination with the explant viral suppression data, will help determine if the test products have the appropriate PK profile to support their use as a rectal microbicide.

The compartmental model of drug migration over time, as conceptualized for CHARM-01, provides a useful framework for PK and PD modeling to understand and predict drug action. We have constructed a conceptual model with 6 anatomic "compartments" within which the microbicide and virus distribute: fluid and cellular subcompartments within the lumen (vaginal and rectal) tissue, and blood compartments (Left Panel, Figure 4). Within the cellular compartment, there are additional transformations of tenofovir to the active diphosphate moiety (TFVpp).

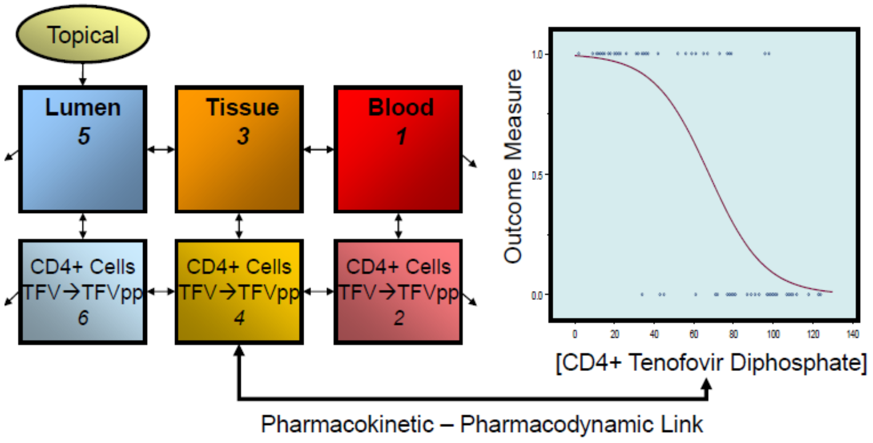

Figure 4: Conceptual Model of a 6-Compartment PK Model Linked to PD Outcomes

The PK model includes the lumen, tissue, and blood compartments further divided into fluid and cellular components (indicated as discrete boxes on the left of Figure 4). Movement of drug among these compartments is a time dependent phenomenon indicated by the bidirectional arrows between compartments and by the unidirectional arrows pointing away from the luminal and blood compartments indicating removal from the body by several clearance mechanisms. The graph on the right indicates any of several PD readouts on the y-axis (such as the risk of infection in a Phase 2B microbicide trial or the ability to infect intestinal explants) with drug exposure (most plausible CD4+ cell intracellular active drug concentration of tenofovir disoproxil fumarate (TDF) on the x-axis. The PK and PD models are linked via active drug concentration at the site of action from the right and left side of the graphic. Large data sets are surely necessary to fill out the complete concentration-response curve shown on the right, but the data from the CHARM studies can begin to flesh out the exposure-response relationship. Success depends upon (1) heterogeneity of drug exposure (distribution of data along the x-axis in the PD graphic), (2) variability in the intercompartmental PK relationship (variability in imputation of drug exposure in a compartment not sampled based on measured concentration in another), and (3) variability in the true PK-PD relationship.

Table 3: PK and PD- Advantages/Disadvantages of Sampling by Compartment

	Colonic Lumen	Colonic Tissue	Blood
**Fluid**	• Reservoir for local colon tissue absorption large impact on tissue kinetics, if absorbed<br>• Minimally invasive sampling	• Reservoir for colon tissue cell uptake at site of action<br>• Difficult to access<br>• Site of RTI prevention action, key for understanding drug exposure-response relation or efficacy	• May represent surrogate for concentration in other sites<br>• Systemic toxicity, if related to tissue levels (non-dosing site)<br>• HIV resistance, if reflects blood intracellular exposure<br>• Most accessible site<br>• Systemic resistance
**Cellular**	• May reflect cellular content of tissue cells<br>• Minimally invasive sampling	• Local GI toxicity<br>• Most difficult to access with sufficient sensitivity	• May represent surrogate for cells in tissue<br>• Systemic toxicity, if reflects non- dosing site tissue cell concentrations<br>• Accessible

The utility of understanding drug concentration over time in these 6 compartments leads to the possibility of building an integrated multi-compartmental PK model where knowledge of dosing regimen and concentration in one compartment allows estimation of concentration in another compartment without sampling. This model has 2 primary uses – one explanatory, the other predictive. First, concentration data from more accessible sampling locations (blood and gut lumen) enable imputation of values in less accessible (tissue and cellular) sites of action. The ability to impute these values becomes critical as one moves from small, intensive exploratory clinical studies where all 6 compartments can be sampled to large confirmatory clinical trials where sparse sampling of a few compartments, at best, is possible. Second, understanding compartmental PK early in microbicide development enables rational decisions about dose selection to achieve desired concentration targets [34].

2.2.2 Mucosal Immunotoxicity

Adequate evaluation of rectal microbicide safety requires a detailed mucosal "immunotoxicity" assessment panel. The data generated from the CHARM-01 study will only provide information about systemic and mucosal safety following exposure to seven doses of various tenofovir-based formulations, and it will be necessary to continue to evaluate the safety of these products in subsequent Phase 2 studies.

There is increasing concern that repeated mucosal exposure to VM or RM could induce subtle immunological changes in the vaginal or rectal mucosa that might increase the risk of HIV transmission, a phenomenon we refer to as "immunotoxicity". A recent study investigated the biological variability of potential intestinal safety biomarkers. Biopsies were collected at 15 and 30 cm from the anal verge from 16 participants on three occasions over a 4 week period in the absence of any

microbicide exposure [31]. Tissue was evaluated for biological variability of a broad range of parameters including histology, mucosal cytokine gene expression, rectal immunoglobulins, and mucosal T cell phenotype. The study demonstrated that tissue from both sites was essentially equivalent and that the most stable parameters included mucosal cytokine gene expression and T cell phenotype.

The first microbicide product to undergo Phase 1 rectal safety assessment with this broader range of safety biomarkers was the vaginally-formulated microbicide UC781, a non-nucleoside reverse transcriptase inhibitor. There were no Grade 3 or 4 nor any procedure-related adverse events (AEs). There were 8 Grade 2 AEs (in 5 participants). All the "immunotoxicity assays" appear to show no change from baseline nor significant differences between study groups [32]. There were also no significant differences in data from tissue samples acquired at 30cm versus those from 10cm, enabling the second Phase 1 RM trial (RMP-02/MTN-007 studying oral/topical tenofovir) to only sample one tissue site.

The current study will use a similar but refined panel of immunotoxicity assays based on data from RMP-02/MTN-006 (tenofovir) and MTN-007 (tenofovir):, rectal and vaginal microflora, rectal fluid cytokine proteins, rectal tissue histology, mucosal tissue mRNA for cytokines, and flow cytometric assessment of mucosal CD4+ and CD8+ T lymphocyte phenotype (CD4, CCR5, CXCR4 CD38 and HLA-DR expression) before and after product exposure.

Intestinal histopathology

Histopathological assessment of intestinal tissue is a routine method of demonstrating mucosal abnormality associated with gastrointestinal diseases such as ulcerative colitis, Crohn's disease, and gluten enteropathy (celiac disease). In general, mucosal change in these diseases can be quite dramatic and microbicide induced changes may be quite subtle. As a consequence we propose to use a qualitative scoring system developed by the inflammatory bowel disease community [36] and adapted for use in HIV Prevention Trials Network (HPTN) 056 study, "Characterization of Baseline Mucosal Indices of Injury and Inflammation in Men for Use in Rectal Microbicide Trials" (See Appendix IV) [31] as well as the completed Phase 1 UC781 rectal application trial (RMP-01) [32] and the Phase 1 oral/topical tenofovir rectal microbicide trial (RMP-02/MTN-006). Prior to the HPTN 056 study, one rectal microbicide study using histological data [26] employed a simple scoring system of normal, slightly abnormal, or abnormal. Using this histological system 69% of the placebo recipients and 89% of the N-9 recipients had slightly abnormal or abnormal rectal biopsies. The scoring system for the HPTN 056 study was developed to provide better discrimination between abnormal and normal histology.

Intestinal mucosal mononuclear cell phenotype

Enzymatic digestion of intestinal biopsies and flow cytometric analysis of T cell populations will be used to determine if product administration is associated in changes in mucosal T cell populations, co-receptor expression, or T cell activation within 24 hours. Co-receptor expression (e.g., CCR5, CXCR4, etc.) on exposed

mucosal immunocytes is required for HIV-1 entry. In healthy HIV-1 seronegative individuals, the expression level of CCR5 is seven-fold higher in mucosal mononuclear cells (MMC) compared to peripheral blood mononuclear cells (PBMC) [22, 23]. It has been shown that MMC are more easily infected with HIV-1 than PBMC [23]. Explanations for the high susceptibility to HIV-1 of MMC may involve the increased expression CCR5, as well as the activation status of the MMC. In addition, the expression of CCR5 has been shown to be up-regulated by pro-inflammatory and T helper (Th)-1 cytokines, while Th-2 cytokines up-regulate CXCR4. CXCR4 is expressed on CD45RO+ T cells in similar levels in MMC and PBMC. This suggests that expression of CCR5 and CXCR4 is partly controlled by Th1/Th2 type of cytokines, which have been shown to be up-regulated in rectal mucosa from HIV-infected patients [37]. It will be important to ascertain whether microbicidal agents trigger similar responses and associated increased vulnerability to HIV-1 infection. The final flow panel will be outlined in the study-specific procedures and determined at the time all of the samples are processed to reflect the most current scientific literature.

Intestinal mucosal cytokine mRNA

Documentation of an increase in mucosal production of pro-inflammatory cytokines such as interleukin (IL)-6 or IL-8 following microbicide exposure may act as a surrogate marker of product-induced toxicity [38]. Recent work has helped define the optimal methodology to measure cytokines in biological samples [39]. In CHARM-01 proinflammatory cytokines that have been associated with increased recruitment of potential HIV-1 target cells and/or replication of HIV-1 infection will be measured.

CCL5, also known as Regulated upon Activation-Normal T Cell Expressed and Secreted (RANTES), macrophage inflammatory protein (MIP)-1α and MIP-1β are the natural ligands for CCR5 while stromal-derived factor (SDF)-1 is the ligand for CXCR4. The physiological function of chemokines and their receptors is to direct migration of recruited lymphocyte subsets to sites of inflammation and immune activation furthering the inflammatory cascade.

In this study quantitative, real-time reverse transcriptase polymerase chain reaction (qRT-PCR) will be used to quantify mucosal messenger RNA (mRNA) expression of relevant proinflammatory cytokines, chemokines, and chemokine receptors in frozen tissue. Frozen tissue (the method used in previous rectal studies related to microbicide development) will be used to allow for comparison to HPTN-056, RMP-01, and RMP-02/MTN-006. The final mucosal mRNA panel will be outlined in the study-specific procedures and determined at the time all of the samples are processed to reflect the most current scientific literature.

Cytokine profile in rectal secretions

As discussed above, measurement of cytokines or chemokines in mucosal tissue or local secretions may provide important information about the potential for a candidate microbicide to induce mucosal toxicity. In addition to the mRNA analysis of intestinal tissue biopsies, cytokine levels in rectal secretions will also be quantified using the

Luminex® technique that can measure multiple cytokine or chemokine proteins in small volumes (< 100 ml) of rectal secretions. Luminex® will be used to measure cytokines, chemokines, and chemokine receptors. The final Luminex® panel will be outlined in the SSP and determined at the time all of the samples are processed to reflect the most current scientific literature.

Microflora

Assessment of pre/post exposure changes in rectal microflora will be conducted. It is currently unknown whether rectal administration of tenofovir 1% gel will prompt a change in the rectal microflora. In humans, transient reductions in vaginal lactobacilli have been noted with vaginal administration of candidate microbicides. Non-human primate studies have not demonstrated significant changes in rectal microflora following rectal administration of vaginal microbicides [45]. In the first human trial assessing rectal application of the UC781 vaginal microbicide formulation, no significant changes were seen within subjects or between study groups in colonization profiles by any of the 23 bacterial categories assessed following cumulative exposure to single and 7 day product use [32].

2.2.3 *Ex vivo* efficacy in the colorectal explant model

Intestinal explants are a novel nonclinical surrogate of efficacy for use in rectal microbicide clinical trials. Colorectal explant techniques have been established and used in several laboratories and have undergone a multi-center validation/standardization process conducted within the NIH sponsored Microbicide Quality Assurance Program [46]. Currently, there are two colorectal explant models; the 'sealed edge' model which provides explant polarization and surrounds the explant with a matrigel cuff to minimize transfer of virus or candidate microbicide around rather than across the explant [47] and the 'exposed edge' model [48] which simply places the explant on a Gelfoam raft. Due to investigators' belief that the act of RAI is traumatic and HIV likely has direct access to sub-epithelial target cells, this second model will be used. Previously in a Phase 1 trial (RMP-01) using UC781, colorectal tissue that was drug-exposed *in vivo* was sampled and studied *ex vivo* for efficacy in suppressing HIV-1 following exposure to laboratory strains of infection. Compared to the participant's biopsy/explant infection at baseline, the 30-minute drug exposure *in vivo* led to a statistically significant *ex vivo* suppression of high-titer HIV infection [33].

2.2.4 Experience and Safety in Colorectal Biopsies for Data Assessments

Endoscopic tissue biopsies from the colorectal area are routinely acquired as part of clinical management as well as a component of mucosal research studies. The rectum is rapidly reparative, with electron micrographs documenting epithelial flattening and stretching to cover the biopsied area in as little as 2 hours. There are limited data on the safety of flexible sigmoidoscopy and rectal biopsy but in a retrospective study from the UK of more than 130,000 procedures, the perforation

rate was quoted as 0.6 per 10,000 procedures and the specific perforation rate for rectal biopsies as 1:8,000 [49]

In healthy individuals, the institutional review boards at UCLA and MRWI currently permit between 20 and 30 biopsies to be collected during a single endoscopic procedure. In the just completed RMP-02/MTN-006 study, 18 subjects experienced 8 flexible sigmoidoscopies each, providing between 13-17 biopsies per procedure over 3-4 months. There were a total of 144 procedures and 2,304 biopsies obtained. There were no cases of significant rectal bleeding or perforation. For context related to CHARM-01, 50% of the individuals in RMP-02/MTN-006 were randomized to a tissue sampling cohort that collected biopsies at 30 minutes and at 24 hours following product exposure.

2.2.5 Acceptability Assessments

Prevention tools are effective only if used. The limited use of condoms by many at-risk individuals illustrates the need for a new HIV prevention product with lower barriers to use. This study will include a behavioral assessment evaluating the experiences participants had with use of three gel formulations. This assessment will not only evaluate experiences with the different gel-formulations, but also evaluate experiences with the design of the vaginal applicator, the use-regimen and the process of application. The acceptability assessment will consist of (1) a baseline behavioral questionnaire, (2) three gel characteristics questionnaires, (3) an application process questionnaire and (4) a qualitative telephone interview. The three quantitative questionnaires have been developed by reviewing literature on acceptability research in vaginal and rectal microbicides [65, 66, 67, 68]. Questions have been selected and adapted to focus on participants' dislikes, as opposed to likes, since these barriers would be reason for product improvements. The qualitative telephone interview will consist of evaluating and clarifying responses given by participants in the quantitative questionnaires. Such a mixed-method design is widely used in acceptability research because of its high yield of information in small sample sizes [68].

A complete table of assessments is included in Appendix 1: Schedule of Study Visits. Table 4, below, includes a summary of the samples collected as they correlate to study endpoints.

Table 4: Summary of Biological Specimen Collections

VISITS		SAFETY AND IMMUNOTOXICITY ENDPOINTS										TENOFOVIR PK CONCENTRATION ENDPOINTS				*EX VIVO* EFFICACY ENDPOINT
		Blood		Urine	Vaginal Swabs	Rectal Swabs		Rectal Sponge	Rectal Biopsies			Blood	Vaginal Sponge	Rectal Sponge	Rectal Biopsies	Rectal Biopsies
		Clinical labs	STDs	STDs, ♀, UA	pH, BV	STDs	Micro-flora	Cytokine fluid	Histo-pathology ~1 bx	MMC (Flow) ~4 bx	mRNA ~1bx	Plasma &PBMC ~24ml/draw	Fluid	Fluid *	Tissue, MMCs ~11 bx	Explants ~4 bx
Visit 1: Screening		✓**	✓†	✓^	✓	✓										
Visit 2: Enrollment/ Baseline		✓	HIV†	✓^		✓		✓	✓	✓	✓	✓	✓	✓	✓	✓
Visit 3: 1st dose of First Formulation *(all specimens collected prior to dose)*			HIV	✓^		✓	✓	✓								
Take-home use of 5 doses of First Formulation																
Visit 4: 7th dose of First Formulation	Pre-dose			♀				✓								
	30 min post-dose								✓	✓	✓	✓ + 2hr & 4hr	✓ + 2hr & 4hr	✓ + 2hr & 4hr	✓	✓
Visit 5:	24 hr post-dose						✓	✓				✓	✓	✓		
Visit 6: 1st dose of Second Formulation *(all specimens collected prior to dose)*			HIV	✓^		✓	✓	✓								
Take-home use of 5 doses of Second Formulation																
Visit 7: 7th dose of Second Formulation	Pre-dose			♀				✓								
	30 min Post-dose								✓	✓	✓	✓ + 2hr & 4hr	✓ + 2hr & 4hr	✓ + 2hr & 4hr	✓	✓
Visit 8:	24 hr post-dose						✓	✓				✓	✓	✓		
Visit 9: 1st dose of Third Formulation *(all specimens collected prior to dose)*			HIV	✓^		✓	✓	✓								
Take-home use of 5 doses of Third Formulation																
Visit 10: 7th dose of Third Formulation	Pre-dose			♀				✓								
	30 min Post-dose								✓	✓	✓	✓ + 2hr & 4hr	✓ + 2hr & 4hr	✓ + 2hr & 4hr	✓	✓
Visit 11:	24 hr post-dose	✓***					✓	✓				✓	✓	✓		

* V2 rectal fluid will be used for intra-visit comparison of cytokine protein levels to tissue cytokine mRNA levels; V4 rectal fluid will be used as baseline for inter-visit comparison of cytokine protein quantification by Luminex®
** Including PT/INR, HBsAb, and HBsAg
*** HBsAg only
† Including plasma archive
^ All urine tests, including ♀

Clinical labs (blood): CBC with diff and platelets, ALT and AST, BUN, creatinine, *(PT/INR and HBsAb – done only at visit 1)*
STD labs (blood): RPR, HSV 1 and 2 serology, HIV *(with plasma archive only at visits 1 and 2)*
STD labs (urine and rectal swab): GC/CT by NAAT
UA (urine): protein, glucose, nitrites, and leukocyte esterase
♀ (urine): hCG

2.3 Description of Study Products

2.3.1 Tenofovir 1% Gel

Tenofovir (sometimes referred to as PMPA,9-[(R)-2-(phosphonomethoxy)propyl] adenine monohydrate) is a novel nucleotide analogue belonging to the class of acyclic phosphonomethylether nucleotides with potent activity against retroviruses. Three formulations of tenofovir 1% gel are to be evaluated in this study: VF, RGVF, and RF. Further information regarding VF and RGVF is available in the current version of CONRAD's investigator's brochure [50] (permission to review IND 73,382 is stated in CONRAD's cross-reference letter). Information regarding the RF tenofovir 1% gel is available in a separate investigator's brochure developed specifically for the rectal formulation (included in the RF IND application).

Each participant will experience seven RF doses and seven RGVF doses of tenofovir 1% gel, but only one dose of the VF, which will be coupled with six preceding exposures to the Universal HEC Placebo Gel to balance out the VF study stage.

2.3.2 Universal HEC Placebo Gel

The placebo gel is the Universal Hydroxyethylcellulose (HEC) Placebo Gel [79], a vaginal product which contains HEC as the gelling agent, purified water, sodium chloride, sorbic acid and sodium hydroxide. HEC is used to approximate the viscosity of microbicide gel candidates.

2.3.3 Mechanisms of Action

Tenofovir is an acyclic nucleotide analogue of adenosine monophosphate. Once inside the cell tenofovir is phosphorylated by cellular enzymes to form tenofovir diphosphate. Tenofovir diphosphate is a competitive inhibitor of HIV-1 reverse transcriptase (RT) that terminates the growing deoxyribonucleic acid (DNA) chain. Tenofovir diphosphate is a weak inhibitor of mammalian DNA polymerases α, β, and mitochondrial DNA polymerase γ.

The Universal HEC Placebo Gel contains the components listed above (Section 2.3.2) [79]. The gel is isotonic and formulated at a pH of 4.4 to avoid disrupting the normal rectal pH (which physiologically has a broad normal range).

2.3.4 Strength of Study Products to be Evaluated in the Study

The strength of the tenofovir gel will be the same concentration (1% w/w) previously tested in HPTN 050 (as stated in DAIDS cross-reference letter for IND 55,690), Centre for the AIDS Programme of Research in South Africa (CAPRISA)-004, CONRAD A04-095 (IND 73,382) and A04-099 (IND 73,382), HPTN 059 (IND 55,690), MTN-001 (IND 55,690), MTN-002 (IND 55,690), and RMP-02/MTN-006 (IND 73,382).

From the current good manufacturing practice (cGMP) formulators, DPT Pharmaceuticals, the density of the VF gel is 1.06 g/mL, of the RGVF gel is 1.02 g/mL, and of the RF gel is 1.04 g/mL. Each gram of gel contains 10 mg of tenofovir, and with the final calculated amount per dose rounded, this results in ~42 mg of tenofovir per dose for the VF and the RF, and ~41 mg for the RGVF. Seven doses of RF and seven doses of RGVF will be used by each participant, but only one dose of VF.

2.3.5 *In vitro* and *Ex vivo* Studies

2.3.5.1 Tenofovir Gel, Various Formulations

Formulation Testing

A new vaginal formulation of tenofovir 1% gel has been developed with a reduced level of glycerin (RGVF). The original formulation, used in all vaginal microbicide tenofovir gel studies, may have been associated with mild gastrointestinal intolerance in a previous rectal microbicide study (RMP-02/MTN-006, see Sections 2.5.1.1.2 and 2.5.2.1.2) [44]. These adverse events were thought to be potentially linked to the high osmolality of the VF (3111 mOsmol/kg) formulation. The RGVF (846 mOsmol/kg) formulation has lower osmolality than the VF formulation, in hopes of improved tolerance.

Additionally, the RF formulation (with osmolality of 479 mOsmol/kg, see below) was developed based on criteria established through an earlier clinical study conducted at Johns Hopkins University by Dr. Craig Hendrix in which placebo products of various product attributes, including various osmolalities, were rectally administered [51]. Distribution, safety and acceptability were assessed. Preliminary results indicated that the aqueous-based candidates studied, both liquid and semi-solid, were acceptable to research participants, were well-retained, and were associated with less damage to colonic epithelium by histology and less small molecule permeability. This is in contrast to the lipid-based candidates, which demonstrated greater permeability and histologic changes in the colonic epithelium. Based on this study, an aqueous-based gel product containing 1% (w/w) tenofovir was formulated for rectal use which has an osmolality of 479 mOsmol/kg, pH of 7.0, increased spreadability, and decreased firmness as evaluated using *in vitro* methods. The RF product has a pH of ~7.0, in the mid-range of the potential fluxes of the rectal compartment. As the VF (pH ~4.5) appeared to only have consequences related to osmolality and pH of the RGVF to be used in this trial is ~4.5, it is not anticipated that the pH of the formulations will impact safety.

The current CHARM-01 study will involve rectal administration of seven doses of the reduced-glycerin vaginal formulation (RGVF) and seven doses of the newly developed rectal specific formulation (RF) of tenofovir 1% gel. The original vaginal formulation (VF) will only be administered as a single dose, however, to balance out the three study stages, it will be preceded by 6 doses of the placebo gel.

Condom Integrity

The compatibility of original tenofovir 1% gel (VF) was tested with three types of lubricated male latex condoms. A matched placebo gel and Universal HEC placebo were used as comparator gels. The condoms tested were representatives of leading brands on the US market (Trojan® and Durex®) with either silicone or aqueous lubricant. The airburst test was used to evaluate changes in film integrity (strength) and test specimens were measured before and after treatment with the gels to assess changes in strength properties following the application of the three gel preparations. All three gels (the original vaginal gel, matched placebo, and the Universal HEC Placebo Gel) were shown to be compatible with the above condoms.

The RF was shown to be compatible with lubricated and non-lubricated latex condoms using the Magee condom compatibility test based on the Texture Analyzer. A second condom compatibility study with the RF was performed by FHI using the ASTM standard testing protocol (ASTM D7661-10). In this testing, 6 styles of condoms were evaluated: 3 non-lubricated latex (Durex®, Lifestyles®, and Trojan®), 2 polyisoprene (Avanti Bare® and Lifestyles SKYN®), and 1 polyurethane (Trojan Supra®). The condoms were treated with the RF gel and tested for tensile (break force and elongation) and airburst (pressure and volume) properties. Untreated condoms subjected to testing procedures served as a control; condoms treated with a known degradant served as a positive control.

The condom testing data for the RF gel was evaluated for statistical significance using Tukey's multiple range test. Overall, in 20 of the 24 sets of results (4 tests and 6 condoms) there were either no significant differences between the treated and control groups or the treated group performed significantly better than the control. In the four sets of results in which the treated condoms performed significantly worse than the controls, observed for two of the non-lubricated latex condoms and for the polyurethane condom, the differences were much smaller (at least 74% smaller) than those seen after exposure of condoms to the known degradant. There were no significant differences between the treated and control groups for the polyisoprene condoms.

The RGVF of tenofovir 1% gel is only slightly modified from the original VF; therefore, it is expected to perform similarly to the original VF. Nevertheless, similar condom testing is planned for the RGVF using the same protocol (ASTM D7661-10). CONRAD's IND 73,382 will include the data for RGVF testing (authorization for review is stated in CONRAD's cross-reference letter).

Safety Testing in Cell Lines

Safety testing of the RGVF in epithelial cell lines has demonstrated retention of transepithelial resistance (TER) by Caco-2 and HEC-1-A cell lines, unlike the original VF, which induced a transient drop in the epithelial resistance. The TER is the resistance that develops once a cell monolayer grows to confluence. A fall in

TER that occurs after product exposure may indicate that the product (e.g. N-9) has innate cellular toxicity. These data suggest that the RGVF may be just as effective as the original VF but less toxic to the rectal epithelium. No detrimental effects have been seen in similar safety testing of the RF [52].

Safety Testing in Colorectal Explant Cultures

Safety testing of colorectal explants shows similar MTT (Formazan [1-(4, 5-dimethylthiazol-2-yl)-3, 5-diphenylformazan]) results with both the RGVF and the original VF. However, histological testing showed retention of the epithelium after application of the RGVF as compared to epithelial stripping with the original VF. Additional testing in colorectal explant cultures also showed that the RGVF did not compromise product efficacy. Collectively, these data suggest that the RGVF is just as effective as the original VF but is less toxic to the epithelium. In addition, no detrimental effects on explant tissue have been seen during testing of the RF using similar *ex vivo* explants.

Anti-HIV-1 Activity, Resistance, and Cross-resistance

The *in vitro* antiviral activity of unformulated tenofovir against laboratory and clinical isolates of HIV-1 was assessed in lymphoblastoid cell lines, primary monocyte/macrophage cells and peripheral blood lymphocytes [53, 54]. The 50% effective concentration (EC_{50}) values for tenofovir were in the range of 0.04 µM - 8.5 µM. In drug combination studies of tenofovir with NRTIs (abacavir [ABC], didanosine [ddI], lamivudine [3TC], stavudine [d4T], zalcitabine [ddC], zidovudine [ZDV]); non-nucleoside reverse transcriptase inhibitors (NNRTI) (delavirdine [DLV], efavirenz [EFV], nevirapine [NVP]); and protease inhibitors (amprenavir [APV], indinavir [IDV], nelfinavir [NFV], ritonavir [RTV], saquinavir [SQV]), additive/synergistic effects were observed. Tenofovir displayed antiviral activity *in vitro* against HIV-1 clades A, B, C, D, E, F, G, and O (EC_{50} values 0.5 µM - 2.2 µM) and showed strain specific activity against HIV-2 (EC_{50} values ranged from 1.6 µM to 5.5 µM).

HIV-1 isolates with reduced susceptibility to unformulated tenofovir have been selected *in vitro* [54, 55]. These viruses expressed a K65R mutation in RT and showed a 2-4 fold reduction in susceptibility to tenofovir. Of note, this mutation also confers increased susceptibility to some other nucleoside reverse transcriptase inhibitors (NRTI), and is associated with approximately 50% reduction in the replicative capacity of HIV-1 (potentially resulting in a “less fit” virus) [56]. Tenofovir-resistant isolates of HIV-1 have been recovered from some patients treated with Viread® in combination with certain antiretroviral (ARV) agents [54]. In treatment-naïve patients, 8/47 (17%) isolates from patients failing Viread® + 3TC + EFV through week 144 showed >1.4 fold (median 3.7) reduced susceptibility in vitro to tenofovir.

Cross-resistance among certain NRTIs has been recognized [53, 54]. The M184V/I and/or K65R substitutions selected *in vitro* by the combination of emtricitabine (FTC) and unformulated tenofovir are also observed in some HIV-1 isolates from

subjects failing treatment with tenofovir in combination with either 3TC or FTC, and either abacavir, didanosine, or zalcitabine. Therefore, cross-resistance among these drugs may occur in patients whose virus harbors either or both of these amino acid substitutions. In treatment-experienced patients, 14/304 (5%) isolates from patients failing Viread® through week 96 showed >1.4 fold (median 2.7) reduced susceptibility to tenofovir. Genotypic analysis of resistant isolates showed a mutation in the HIV-1 RT gene resulting in the K65R amino acid substitution. HIV-1 isolates from patients (n = 20) whose HIV-1 expressed a mean of 3 ZDV-associated RT amino acid substitutions (M41L, D67N, K70R, L210W, T215Y/F, or K219Q/E/N) showed a 3.1-fold decrease in the susceptibility to tenofovir. Multinucleoside resistant HIV-1 with a T69S double insertion mutation in the RT showed reduced susceptibility to tenofovir [55].

2.3.5.2 Universal HEC Placebo Gel

Formulation Testing

Analyses of pH (pH of HEC gel alone is 4.4; mixed with human seminal plasma is 8.03) found that a HEC formulation did not show significant buffering capacity and could not acidify the alkaline pH of seminal plasma, a favorable property for a placebo formulation [72]. *In vitro* assessments of spermicidal activity utilizing human semen from healthy donors showed that HEC gel had little effect on sperm motility during a 60-minute incubation.

Safety Testing in Cell Lines

Dilutions of the HEC gel in culture medium exhibited negligible toxicity to human vaginal epithelial cells (standard MTT assay), even at the lowest dilution tested (1:2) [79]. Exposure of human vaginal epithelial cells to the HEC gel resulted in minimal IL-1α induction, even at the lowest dilutions tested (lowest dilution, 1:2). Additional studies have shown that HEC gel is safe to peripheral blood mononuclear cells, and colorectal epithelial cell lines [47, 73]. Indeed, no changes in the transepithelial resistance were noted after HEC gel was applied [73].

Safety Testing in Colorectal Explant Cultures

The HEC gel was applied to colorectal explant tissues using a polarized system [47]. For safety analysis the MTT assay and histology were performed. No observed reduction in the MTT levels or changes in the tissue architecture were noted.

Anti-HIV-1 Activity

Further analysis showed that this gel has no anti-HIV activity as it did not protect peripheral blood mononuclear cells, macrophage, or colorectal explant cultures from infection [47, 73].

2.4 Animal Studies

2.4.1 Vaginal Formulation (VF) of Tenofovir 1% Gel

2.4.1.1 Vaginal Administration

Please see the Investigator's Brochure for the Vaginal Formulation, which is an appendix of the Investigator's Brochure for the Rectal-specific formulation, for descriptions of animal studies carried out involving vaginal administration of the VF.

2.4.1.2 Rectal Administration – Toxicology

14-Day Rectal Irritation Study of Tenofovir Vaginal Gel in Rabbits

A fourteen-day repeat dose toxicology study of tenofovir vaginal gel in New Zealand White rabbits following rectal administration was carried out. The objective of the study was to determine the potential toxicity and pharmacokinetics of tenofovir vaginal gel formulations following single, daily rectal administration for fourteen days to male and female rabbits.

Forty New Zealand White rabbits (approximately 10-12 weeks of age and weighing in the range of 2.0 to 2.5 kg at initiation of treatment) were assigned to five dose groups (one sham control, one placebo control and three active test article) consisting of four animals per sex per group under Good Laboratory Practices [(GLP) Pacific BioLabs, Hercules, CA] [50]. The placebo control and active test articles consisted of tenofovir matched placebo gel and three different concentrations (1%, 3%, and 10%) of tenofovir gel respectively. The lubricant for the sham control group was K-Y Jelly from a commercial source.

All female animals were dosed for 14 days and all male animals were dosed for 15 days. Animals in Groups 2 to 5 received 1 mL doses of the respective placebo or test articles via rectal administration for 14/15 consecutive days. A short, soft catheter was attached to a syringe and filled with 1 mL of the appropriate test article. Animals in Group 1, (sham control) underwent the same treatment procedure for 14/15 days with the exception that no dose was administered and the catheter was lubricated with a non-irritating lubricant (K-Y Jelly) prior to insertion. The rectal route of administration was selected as it is the intended clinical route of administration.

Table 5: Rectal Irritation Study in New Zealand White Rabbits

Group	Intervention
1	Sham
2	Tenofovir placebo gel
3	Tenofovir 1% gel
4	Tenofovir 3% gel
5	Tenofovir 10% gel

The test article, VF tenofovir 1% gel, was well tolerated at dose concentrations (1 mL dose volume) of 1% (10 mg/dose), 3% (30 mg per dose) and 10% (100 mg per

dose) when administered as a daily rectal dose for 14 days to female rabbits or 15 days to male rabbits. There was no mortality in this study, and there was only one clinical finding that was potentially study-related: redness at the site of administration in one animal on one day of dosing. There was no evidence of a test article effect on body weight, body weight gain or food consumption over the dose period.

The test article, at the concentrations tested, was without significant effect at the rectal site of administration. Gross pathology at necropsy provided no evidence for tissue damage or inflammation of the rectum or surrounding tissues at the concentrations tested; histopathological evaluation of the rectum and parts of the colon immediately adjacent to the rectum also showed no effect at the concentrations tested. Each rectum sample was subsectioned into proximal, mid and distal sections (in relation to the site of test article application) for histopathological analysis. Within each section, at least 5 subsections were evaluated for inflammation and other types of lesions. As mentioned, no differences were seen.

Rectal administration of the test articles produced little evidence of test article related systemic effects, despite measurable systemic exposures to tenofovir. At necropsy, gross pathology provided no *in situ* evidence for tissue damage or target organ effects. Changes in several hematology, coagulation and clinical chemistry parameters that reached statistical significance were not considered test article related because they were typically sporadic, not dose-related, and were present in only one gender of rabbit on each occasion. Organ weight changes also reached statistical significance on occasion, but these were also considered not to be test article related for the same reasons cited above, i.e., sporadic and not dose-related. No tissues or organs other than the rectum and colon were examined for histopathological changes.

Measurement of tenofovir in rabbit plasma utilized a validated LC-MS/MS method, with a lower limit of quantification (LLOQ) of 1 ng/mL and an upper limit of quantification of (ULOQ) of 2500 ng/mL. Rectal application of test articles resulted in measurable systemic concentrations of tenofovir at all dose levels after the first dose on Day 1 and the Day 14 dose. Tenofovir exposures were variable on Day 1. However, by Day 14 plasma concentrations were more consistent among individual animals and there was a clear dose-related increase in tenofovir exposures in both male and female rabbits. Systemic exposures to tenofovir were comparable in female and male rabbits. Absorption of tenofovir was relatively rapid, with the plasma T_{max} occurring at 1 hr on Day 1 (for most dose groups) and at 2 hr (female rabbits) and 4 hr (male rabbits) on Day 14. Mean C_{max} values on Day 1 ranged from 11.7 ng/mL (Group 4 females) to 59.0 ng/mL (Group 3 females), except Group 3 males where the C_{max} was 1182 ng/mL. Mean C_{max} values on Day 14 ranged from a low of 32.3 ng/mL (Group 3 males) to 265 ng/mL (Group 5 males). The mean T_{max} and C_{max} values for Group 3 males on Day 1 were skewed by one male rabbit with a very high tenofovir plasma concentration at 24 hr post dose of 4210

ng/mL. The elimination half-life for tenofovir could not be determined with accuracy due to the variable exposures on Day 1, and a poorly defined terminal elimination phase on Day 14. For those groups where a half-life could be measured on Day 14, the $t_{1/2}$ for tenofovir ranged from 11.3 to 16.2 hours. It is possible that continued absorption of tenofovir from the rectal site of administration contributed to the inability to accurately measure half-life on Day 14. Tenofovir plasma concentrations increased in both female and male rabbits with increasing dose. However, the increase in exposure was somewhat less than dose proportional. On Day 14 when tenofovir plasma concentrations were most consistent across individual animals, the decrease in dose-proportional exposure for C_{max}/Dose between Group 3 (10 mg) and Group 5 (100 mg) was 66% and 18% for female and male rabbits, respectively. The decrease for AUC_{last}/Dose between Group 3 and Group 5 was 52% and 32% for female and male rabbits, respectively. There was a marked increase in tenofovir exposure over the 14 days of rectal administration. Accumulation ratios (AUC_{last} Day 14/AUC_{last} Day 1) varied from 7.2 to 23.7 across dose groups.

The No Observed Adverse Effect Level (NOAEL) for rectal administration of test article in this study was greater than the highest concentration tested, i.e., >10% tenofovir in vaginal gel (a 100 mg dose).

This study is described on pages 16, 17, and 26 of the Investigator's Brochure for the VF.

2.4.1.3 Rectal Administration – Pharmacokinetics

PK data from rectal administration of the VF come from two studies, described below.

Macaque Pharmacokinetic Study – Rectal Administration

This pharmacokinetic study in rhesus macaques [71] involved first intravaginal and then intrarectal administration of 0.2, 1 or 5% tenofovir in the VF. Groups of 6 Rhesus macaques were pretreated with depo-medroxyprogesterone acetate (30 mg i.m.) 30 days prior to tenofovir administration to synchronize menstrual cycles and thin the vaginal mucosa. Gel containing 0.2, 1 or 5% tenofovir was administered intravaginally at a dose volume of 0.6 mL/kg. Plasma and vaginal and rectal fluid samples were collected pre-dose and at 0.25, 1, 4, 8 and 24h after dosing for analysis of tenofovir concentrations by validated LC-UV or LC-MS/MS methods. At 24 hours, biopsies from the vaginal wall, cervix and rectum were collected for analysis of tenofovir and tenofovir diphosphate concentrations. After a 3-week washout period, the same gel doses were applied intrarectally to the same animals, with samples collected as outlined above. Pharmacokinetic parameters were generated using noncompartmental analysis (WinNonlin 5.1).

Following vaginal and rectal administration, tenofovir was detectable by 0.25h in all matrices distal to where the dose was administered (plasma and vagina or rectum). Except for vaginal and rectal dosing with 0.2% tenofovir, at least 5/6 macaques tested in the 1% and 5% groups had detectable tenofovir concentrations in all matrices 24 hours after dosing. At all doses, concentrations at the dosing site were typically 1-2 logs higher than in the opposite compartment, and 4-5 logs higher than in the plasma. Vaginal dosing resulted in local vaginal fluid C_{max} and AUC_{0-24} values that were 58-82% lower than were achieved in rectal fluid with rectal dosing. Conversely, vaginal dosing resulted in plasma C_{max} and AUC_{0-24} values that were 1-2 fold greater than were achieved in plasma after rectal dosing. AUC_{0-24} values in plasma ranged from 0.02-0.04% of those in vaginal fluid after vaginal dosing and from 0.002-0.008% of those in rectal fluid after rectal dosing. This study is described on page 14 of the Investigator's Brochure for the VF.

<u>Macaque Efficacy Studies – Rectal administration</u>

This pilot macaque efficacy trial [58] involved a single rectal dose of the VF, and is described below in section 2.4.1.4. Plasma samples from that study were assayed for tenofovir concentration by the Clinical Pharmacology and Analytical Chemistry Core of the University of North Carolina Center for AIDS Research. Drug concentrations in plasma were determined by a validated high pressure liquid chromatography (HPLC) method with ultraviolet detection. This method utilized a dynamic range of 10 to 10,000 ng/mL, with intra- and inter-day variability of <10% across this range. Total tenofovir concentrations were assayed in tissues using a fully validated HPLC method with mass spectrometry detection.

Analysis of intestinal tissue samples collected at necropsy showed that all tenofovir-dosed animals had measurable concentrations of drug in lysates of colorectal tissue at concentrations between 20.8 and 54.2 µg/g protein but no drug was detected in lysates of homogenates from the small intestine. Tissues from untreated animals acted as negative controls. To indirectly estimate the amount of intracellular phosphorylated tenofovir in tissues, samples were analyzed with (to measure the combination of tenofovir + tenofovir monophosphate + tenofovir diphosphate) and without (to measure tenofovir only) phosphatase hydrolysis. Subtracting the concentration of tenofovir obtained from tissue samples without phosphatase, from the concentration of tenofovir obtained from tissue samples with phosphatase, demonstrated that between 46-75% of total tenofovir in tissues was present as the intracellular monophosphate and diphosphate forms. Based on intracellular data describing tenofovir monophosphate: diphosphate ratios, it was estimated that approximately 30-60% of total tenofovir in tissues was present as the intracellular diphosphate form. The relatively low rectal dose of tenofovir gel applied, an average of 10 µg/kg, resulted in a maximum plasma detection level of 0.19%, which was detected 15-minutes after rectal dosing. This study is described on page 30 of the Investigator's Brochure for the VF.

2.4.1.4 Rectal Administration – Efficacy

The rectal application of tenofovir was evaluated for protective efficacy against rectal challenge with SIV in a well established and standardized pre-clinical macaque model [58]. A total of 20 purpose-bred Indian rhesus macaques were used to evaluate the protective efficacy of topical tenofovir. Six animals received tenofovir 1% gel *per rectum* 15 minutes prior to virus challenge and 3 macaques received tenofovir 1% gel per rectum 2 hours prior to virus challenge, whereas 4 macaques received placebo gel and 4 macaques remained untreated. In addition, 3 macaques were given tenofovir gel 2 hours after virus challenge. Following intrarectal instillation of 20 median rectal infectious doses (MID_{50}) of a non-cloned, virulent stock of $SIV_{mac251/32H}$ all animals were analyzed for virus infection, by virus isolation (VI) from PBMC, quantitative proviral DNA load in PBMC, plasma viral ribonucleic acid (vRNA) load by sensitive quantitative competitive (qc)-RT PCR and presence of SIV-specific serum antibodies by Enzyme-Linked Immunosorbent Assay (ELISA).

A significant protective effect was seen (p=0.003; Fisher's Exact Probability test) wherein 8 of 9 macaques given tenofovir *per rectum* either 15 minutes or 2 hours prior to virus challenge were protected from infection (n=6) or had modified virus outcomes (n=2) while 4 of 4 untreated macaques and 3 of 4 macaques given placebo gel were infected, as were 2 of 3 animals receiving tenofovir gel after challenge. Moreover, analysis of lymphoid tissues *post mortem* failed to reveal sequestration of SIV in the protected animals.

Colorectal explants from non-SIV challenged tenofovir treated macaques were resistant to infection *ex vivo*, whereas no inhibition was seen in explants from the small intestine. Tissue-specific inhibition of infection was associated with the intracellular detection of tenofovir. In colorectal explants from 3 of 4 animals, complete or nearly complete inhibition of virus replication was seen and in the other animals, a high level of variability between replicate samples resulted in lower mean inhibition. In contrast, inhibition of virus replication was not seen in explants from the small intestine suggesting that tenofovir was, at least in part, acting on cells at the virus portal of entry.

PK results from this study are described above, in Section 2.4.1.3. This study is described on page 30 of the Investigator's Brochure for the VF.

2.4.2 Reduced Glycerin Vaginal Formulation (RGVF) of Tenofovir 1% Gel

28-Day Rectal Dose Toxicity Study in Male and Female Rabbits

The formulation changes to the tenofovir 1% gel were minor (i.e. lowering the glycerin content and making slight adjustments in other excipients), and such reductions do not typically require additional safety evaluations (*FDA Guidance for Industry, Nonsterile Semisolid Dosage Forms – Scale-up and Post-Approval*

Changes.) Nevertheless, a 28-day rabbit rectal toxicity study of the RGVF was conducted in August-September 2011. This study was designed to evaluate the potential toxicity of various gels, including rectal tenofovir (TFV), griffithsin (GRFT), TFV and GRFT in combination, and reduced glycerin vaginal formulation of TFV (RGVF). Four treatment groups of four male and four female New Zealand White Hra:(NZW)SPF albino rabbits received one of four test articles, 1% TFV, 0.1% GRFT, 1% TFV/0.1% GRFT, and RGVF of TFV 1% Gel. One additional group of four animals/sex served as the control and received the rectal placebo gel. The study product (placebo or test article) was administered to all groups rectally, once a day for 28 consecutive days, at a dose volume of 1 mL, with standard observations of morbidity, mortality, food consumption, etc. Blood and urine samples for clinical pathology evaluations were collected from all animals pretest and at the terminal necropsy; blood samples for evaluations of plasma concentrations of the test article were collected from all animals on Days 1 and 28.

All animals survived to the scheduled necropsy on Day 29. No test article-related effects were evident in the following parameters evaluated: clinical observations, body weights, food consumption, hematology, coagulation, clinical chemistry, urinalysis, organ weights, and macroscopic pathology. There were no test article-related microscopic findings with the exception of males administered 1.0% TFV/ 0.1% GRFT rectal gel and RGVF of TFV 1% gel. Microscopic findings in these groups consisted of minimal to mild depletion of secretory material from the mucosal cells and goblet cells of the rectum. There was no apparent atrophy of the epithelium and the apparent decrease in goblet cells was attributed to the loss of secretory contents and thus decreased prominence of these cells. This finding was not considered to be adverse, given the low severity and lack of further epithelial changes.

In conclusion, 28 days of once daily rectal administration of 1% rectal tenofovir (TFV), 0.1% griffithsin (GRFT), 1% TFV and 0.1% GRFT in combination, and reduced glycerin vaginal formulation of 1% TFV (RGVF) in male and female rabbits did not result in local or systemic toxicity.

2.4.3 Rectal Formulation (RF) of Tenofovir 1% Gel

2.4.3.1 Rectal Administration – Toxicology

14-Day Rectal Irritation Study of Rectal Specific Formulation (RF) of Tenofovir Gel in Rabbits

The potential toxicity and toxicokinetics of GLP tenofovir rectal specific gel formulation (RF) was evaluated following once daily rectal administration for 14 consecutive days in male and female rabbits. Three treatment groups of four male and four female New Zealand White rabbits were administered the RF gel at respective dose levels of 1, 3, or 10%. Two additional groups of four animals/ sex served as control groups and received the saline control or placebo gel.

The saline control, placebo, or test article was administered at a fixed dose volume of 1 mL.

Observations for morbidity, mortality, injury, and the availability of food and water were conducted twice daily for all animals. Detailed clinical observations were conducted daily at 1 to 2 hours post dose. Body weights were measured and recorded weekly. Food consumption was measured and recorded daily and reported weekly. Physical examinations were conducted pretest. Blood and urine samples for clinical pathology evaluations were collected from all animals pretest and prior to the terminal necropsy. Blood samples for determination of the plasma concentrations of the test article were collected from all animals at designated time points on Days 1 and 14. At study termination, necropsy examinations were performed, organ weights were recorded, and tissues were microscopically examined.

All animals survived to the scheduled necropsy on Day 15. No test article-related effects were evident in the following parameters evaluated: clinical observations, body weights, food consumption, hematology, coagulation, clinical chemistry, urinalysis, organ weights, and macroscopic and microscopic pathology.

In summary, 14 days of once daily rectal administration of RF gel at concentrations of 1, 3, or 10% to male and female rabbits did not cause local or systemic toxicity. The NOAEL for the RF was also considered to be the highest concentration of 10% tenofovir.

2.4.4 Universal HEC Placebo Gel

HEC is the gelling agent in the placebo gel. The results of multiple animal studies have been consistent with the safety of this ingredient. A recently completed rectal study in a macaque model also appears to be consistent with the safety of this ingredient.

Toxicology

Up to 55 intravenous injections of HEC were given to dogs (dose and number not specified) without causing injury other than that typical of the other water-soluble cellulose ethers [74]. Only transitory changes in the blood picture and the deposition of the material on the intima of the blood vessels were noted. Groups of rats maintained for two years on diets containing HEC (n not specified, up to 5%) did not exhibit any adverse effects. HEC has also been administered to rats in single oral doses as high as 23,000 mg/kg without observed toxic effects (n not specified).

Intraperitoneal administration of unformulated HEC to pregnant mice in a 1% and 4% concentration caused an increase in resorptions, but no detectable increase in birth defects [75]. While no epidemiological studies of congenital anomalies in infants born to women exposed to HEC during pregnancy have been reported, the Teratogen

Information System (TERIS) considers the magnitude of teratogenic risk to a child born after exposure during gestation to be none [76].

CF-1 mice (n not specified) pretreated with medroxyprogesterone acetate were administered 0.02 mL of HEC gel vaginally, followed by a 0.01 mL inoculum of 10 intravaginal $dose_{50}$ units of HSV-2 0.3 minutes later [72]. On day 3, vaginal lavage was cultured on human foreskin fibroblasts, and mice were considered infected if a cytopathic effect was observed after 3 days of incubation. Control animals were treated similarly but were not administered the test article. Infection rate following pretreatment with HEC gel (90%) was not significantly different from pretreatment with PBS (80%) or from mice given no treatment (% not specified). HEC gel did not enhance susceptibility of mice to HSV-2 when administered 12 hours before vaginal challenge [79].

A 10-day rabbit vaginal irritation study (10/arm, 2 arms, HEC gel vs. 0.9% saline control) found that the HEC gel was not irritating to the vaginal mucosa of rabbits when dosed daily for 10 days. One animal in the HEC gel group had an instance of vaginal redness (compared to four animals in the saline group), which did not persist and was not evident at the end of the study. Diarrhea, few feces, and soiling of the anogenital area were noted in that animal. Body weight changes were noted to be normal. In 9 of 10 animals, necropsy results were normal. Anogenital soiling was observed in the animal that exhibited erythema during the in-life phase of the study. Histopathological changes observed were similar to those seen in the saline control group and likely attributable to those that occur as a result of the repeated insertion of a catheter, rather than due to any effect of the test samples.

HEC gel was used as the placebo comparator in a recent rectal safety study of a combination microbicide in a macaque model [35]. A third study arm received no product and served as a negative control. Rectal safety of the active product and HEC gel was evaluated following four daily applications of study products. Rectal flora, pH, and rectal lavage samples were assessed pre-and post-dosing and showed no evidence of toxicity in the macaques that received HEC gel. The infrequent evidence of epithelial sloughing and rare incidence of associated blood cells in rectal lavage samples was similar in the HEC placebo and no product arms of this study.

Effectiveness

The effect of the placebo gel on vaginal transmission of $SHIV_{162p3}$ (10^3 $TCID_{50}$) to rhesus monkeys was determined in two separate studies (n = 5, n = 3, respectively) [79]. Macaques pretreated with medroxyprogesterone acetate were vaginally administered 1 mL of the HEC gel formulation 15 minutes prior to challenge with 0.5 mL $SHIV_{162p3}$. Investigators monitored total RNA load in the animal plasma for a total of 8 weeks by means of a standard quantitative RT-PCR. The first study utilized the HEC gel formulation at pH 6.5; the second study utilized a formulation at pH 4.4. In both studies, all monkeys were infected, as determined by the presence of viral RNA in circulating blood, regardless of the pH of the formulation.

2.4.5 Summary

All of the animal studies conducted to date provide support that the three formulations to be used in CHARM-01 have been deemed acceptable vis a vis animal toxicity, pharmacokinetics, and efficacy, with the highest NOAEL tested being a concentration of 10%.

2.5 Human Studies

This study, along with a companion CHARM-02 trial, will be the first rectal safety study of the new rectal specific formulation (RF) of tenofovir 1% gel. Two Phase 1 rectal studies are currently evaluating the RGVF (MTN-007 and Project GEL). However, a broad range of studies have been conducted with the original VF. Most of the studies involve vaginal or penile application of the gel and there is one study of rectal application; data are summarized below.

2.5.1 Pharmacokinetics

2.5.1.1 Vaginal Formulation (VF) of Tenofovir 1% Gel

CONRAD holds the IND for the vaginal formulation of tenofovir 1% gel (IND 73,382) and provided a cross-reference letter. The RGVF gel is covered under the same IND and is referenced in the same letter.

2.5.1.1.1 Vaginal Administration

Data from “Phase 1 Safety and Acceptability Study of the Vaginal Microbicide Agent PMPA Gel”, also known as HPTN 050 has been published [59]. Eighty-four (60 HIV-negative and 24 HIV-positive) women applied either 0.3% or tenofovir 1% gel once or twice daily for 14 days. Systemic absorption was limited (maximum serum levels 3.1-25.8 ng/mL).

In MTN-002, the first microbicide trial to be conducted during pregnancy, 16 women received a single vaginal dose of tenofovir 1% gel prior to elective cesarean section. Tenofovir levels were measured in blood, amniotic fluid, cord blood, endometrial tissue, and placental tissue. Plasma tenofovir levels were compared to historical controls. Study results demonstrated that the PK levels of a single vaginal dose of tenofovir 1% gel in pregnant women was similar to those found in non-pregnant women and that serum tenofovir levels were up to 50 – 100 times less as compared to standard oral dosing. Additionally, tenofovir was shown to get to the fetal compartment with low overall cord levels (~40 times less than oral dosing), but with a similar cord blood: maternal ratio. Overall findings suggest that tenofovir is safe for use in term pregnancy and warrants additional investigation during pregnancy.

In MTN-001, the first head-to-head comparison of VF tenofovir 1% gel dosing and oral tenofovir disoproxil fumarate (TDF) oral dosing, at 7 clinical sites, 144 women received 6 weeks of daily dosing with oral TDF, vaginal VF 1% gel, or both in a 3 period cross-over study. TFV and TFV-DP concentrations were assessed in blood, tissue, and vaginal lumen. Serum TFV concentrations were 50-times greater following oral dosing when compared to vaginal dosing. By contrast, cervicovaginal lavage TFV concentrations were 1000 times greater following vaginal dosing. TFV-DP was greater after vaginal dosing when compared to oral dosing in the following anatomic sites - vaginal tissue (100-fold) and endocervical cytobrush cells (20-times). In contrast PBMC TFV-DP concentrations were 20-fold greater after oral TDF dosing compared to vaginal dosing. TFV-DP concentrations in PBMC after steady-state vaginal dosing were below the limits of quantitation (median and up to the 75% percentile) [60].

2.5.1.1.2 Rectal Administration

In RMP-02/MTN-006, 12 male and female research participants received sequentially: single dose oral TDF, single dose VF 1% per rectum, 7 daily doses of VF 1% gel per rectum. Blood, tissue, and luminal sampling followed each of these 3 dosing periods. In general, findings comparing oral and rectal dosing were similar to findings in MTN-001 comparing oral and vaginal dosing. At 30 minutes following single rectal, topical dosing, colon tissue concentrations were 100-times greater than 30 minutes after single oral dosing. Multiple rectal doses resulted in five times greater concentrations in tissue when compared to a single rectal dose with no significant increase in plasma concentrations. PBMC TFV-DP concentrations were below limits of quantitation in the vast majority of specimens collected in the 24 hours following a single rectal dose. For this reason and given the vaginal steady-state dosing PBMC results noted above, PBMC assessments are not likely to be fruitful in this study [44].

2.5.1.2 Reduced Glycerin Vaginal Formulation (RGVF) of Tenofovir 1% Gel

CONRAD holds the IND for the RGVF of tenofovir 1% gel (IND 73,382) and provided a cross-reference letter. The VF gel is covered under the same IND and is referenced in the same letter.

2.5.1.2.1 Vaginal Administration

As noted in section 2.4.1.2, the formulation changes to the original tenofovir 1% gel were minor. The glycerin reduction change was made without the need to perform another safety study, since it was a reduction in an excipient. Such reductions do not typically require additional safety evaluations (*FDA Guidance for Industry, Nonsterile Semisolid Dosage Forms – Scale-up and Post-Approval Changes.*)

2.5.1.2.2 Rectal Administration

No vaginal administration studies have been planned for evaluation of RGVF. Two Phase 1 rectal-administration studies will evaluate the RGVF tenofovir 1% gel. MTN-007 is currently exploring this formulation, and a second study called Project GEL is planned for initiation.

2.5.1.3 Rectal Formulation (RF) of Tenofovir 1% Gel

2.5.1.3.1 Vaginal and Rectal Administration

Since the RF formulation is strictly for rectal for use, no vaginal administration studies will be conducted. As previously mentioned, this study, along with a companion CHARM-02 trial, will be the first rectal administration study of this formulation.

2.5.1.4 Summary

HPTN 050, MTN-002, MTN-001, and RMP-02/MTN-006 studies have been conducted with tenofovir 1% gels, three of which used VF administered vaginally and one used VF administered rectally. The glycerin reduction change in the original formulation for the RGVF gel was made without the need to perform another safety study, since it was a reduction in an excipient. Such reductions do not typically require additional safety evaluations (*FDA Guidance for Industry, Nonsterile Semisolid Dosage Forms – Scale-up and Post-Approval Changes.*) Overall, these previous studies provide support for moving forward with testing the new RF formulation in this CHARM-01 trial.

2.5.2 Safety

2.5.2.1 Vaginal Formulation (VF) of Tenofovir 1% Gel

CONRAD holds the IND for the vaginal formulation of tenofovir 1% gel (IND 73,382) and provided a cross-reference letter. The RGVF gel is covered under the same IND and is referenced in the same letter.

2.5.2.1.1 Vaginal and Penile Administration

In HPTN 050, the tenofovir 1% gel formulation was well tolerated in both HIV-uninfected and -infected women [59]. Further, 94% of female participants and 81% of male participants indicated they would definitely or probably use tenofovir vaginal gel in the future. While a number of participants (92%) reported some type of AE, the majority of them were mild (87%) and limited to pruritus (n = 18), erythema (n=14), petechiae/ecchymosis (n = 14), vaginal discharge (n = 13), and burning (n = 10). Only four severe AEs were reported, but of these, only one (lower abdominal pain) was thought to be product-related. Product concentration, sexual activity and HIV status were not

associated with a specific AE pattern. No clinically significant systemic toxicity was observed. No serious adverse events (SAEs) were reported.

In a male tolerance study (CONRAD A04-099/IND 73,382) [61], tenofovir 1% gel was well tolerated in men following seven days of once daily exposure, for 6 to 10 hours, to the penis. There were few reported and observed genital findings after product use including mild pain (burning, irritation, discomfort) and pruritus. All observed findings were classified as mild, small in size and requiring no treatment. Reported symptoms were mild, of short duration and resolved by the final visit. There were no noticeable differences between signs and symptoms of genital irritation in the circumcised compared to uncircumcised group.

A Phase 2 study of tenofovir 1% gel (HPTN 059) has completed follow up [50]. This study assessed safety and acceptability of, and adherence to a regimen of tenofovir gel for vaginal use in HIV-uninfected women versus a placebo gel. Exploratory objectives included measurement of vaginal flora characteristics, assessment of the effects of gel on genital cytokine and chemokine expression, and the evaluation of cytokine and chemokine expression to correlate expression with evidence of inflammation, epithelial disruption and genital symptoms. The study was a four-arm, three-site, randomized, controlled trial comparing gel used once daily and gel used prior to intercourse, to placebo gel, with 6 months gel exposure and follow-up. The study was conducted among 200 women in Pune, India; Birmingham, Alabama, USA; and New York, New York, USA. Participants were sexually active, HIV-uninfected women between ages 18 and 50, but not menopausal or post-menopausal. Participants had six months of study gel exposure and follow-up. They were randomized to either the once daily or coitally dependent group, and received either tenofovir or placebo gel. Participants received single use unit dose tubes and single-use applicators.

No statistically significant differences were seen between those receiving active and placebo gels in complete blood count (CBC), liver function tests, or renal function tests. Among those using a study gel daily, no participants had pelvic exam findings involving generalized erythema or severe edema or deep epithelial disruption at any follow-up visit during the study. At the Week-24 Visit, no participants had exam findings suggestive of vaginitis, cervicitis, superficial disruption, disrupted blood vessels, or intermenstrual bleeding. Adherence to study gel was high, and was supported by PK data. 79% of women reporting gel use in past 12 hrs had low but detectable plasma tenofovir supporting self-reported adherence data. Daily and coital use was highly acceptable to women. These data suggest a favorable safety and acceptability profile of tenofovir gel, and support routine monitoring for genital findings among women without genital symptoms at six month intervals.

A Phase 2b study of vaginally-administered tenofovir 1% gel use (CAPRISA 004) has recently completed follow-up and data analysis [62]. This study, conducted among sexually active, HIV-uninfected women at an urban and rural site in South Africa, compared the safety and effectiveness of tenofovir 1% gel when used within 12-hours before and after intercourse, versus placebo gel (HEC). Safety assessments as well as HIV and urine pregnancy tests were performed at monthly follow-up visits. Pelvic exams were also performed at quarterly visits.

Study results suggest that vaginally-administered, coitally-dependent use of tenofovir 1% gel is safe. No increases in renal, hepatic, pregnancy-related, or genital AEs were observed. Additionally, tenofovir 1% gel was shown to reduce HIV infection by approximately 39% regardless of sexual behavior, condom use, herpes simplex virus (HSV)-2 infection, or urban/rural location. It is important to note, however, that the high acceptability rate (~97%) did not correspond to the average adherence rate (~61%).

In September 2009, the Microbicide Trials Network initiated VOICE – Vaginal and Oral Interventions to Control the Epidemic – conducted across 15 study sites in Uganda, South Africa and Zimbabwe. VOICE was designed as a Phase 2b HIV prevention trial to evaluate two antiretroviral-based approaches: the daily use of an ARV tablet as oral PrEP and the daily use of a microbicide vaginal gel. As originally designed, VOICE had five study groups, with about 1,000 participants to be enrolled in each: tenofovir vaginal gel, inactive vaginal placebo gel, tenofovir tablet, Truvada tablet, and a placebo tablet. The tenofovir tablet arm was discontinued in October 2011 after it was determined that although the tenofovir tablets were safe, they were no better in preventing HIV than the placebo tablet.

A routine DSMB review of the study data of 5,029 participants enrolled between the start of the study and September 2011 was completed in November 2011. Although the DSMB identified no safety concerns with the tenofovir vaginal gel, it recommended that VOICE discontinue the tenofovir gel arm and the placebo gel arm of the study, because there was no difference in effect between them in preventing HIV infection in the women enrolled in the trial. Because the DSMB had no major concerns about the safety of tenofovir gel, the participants randomized to the gel arms will be discontinued from the study at their next scheduled clinic visit.

No safety concerns were identified in the oral Truvada® tablet, and VOICE will continue to test Truvada's safety and effectiveness [80].

2.5.2.1.2 Rectal Administration

RMP-02/MTN-006 is a just-completed, Phase 1 trial of single and 7-day rectal exposure of vaginally-formulated tenofovir 1% gel, with comparisons made with

single dose oral tenofovir tablet in 18 HIV-seronegative participants. Single-dose topical exposure of the tenofovir 1% gel showed no increase in AEs compared with placebo. The 7-day exposure of the %1 tenofovir gel identified no difference in Grade 1 or 2 AEs compared to placebo. However, there were 6 of 8 Grade 3 AEs reported during the 7-day exposure. These were in 2 of the 12 drug-exposed subjects, and were mainly lower gastrointestinal complaints that resolved. This was not statistically significant in this small study but was thought to be related to the hyperosmolar gel. Response to this finding further supported the production the reduced osmolar vaginal formulation (reduced-glycerin VF) and now use in ongoing clinical trials (MTN-007). Systemic absorption of the topical tenofovir was slower and peaked lower than the single oral dosing earlier (in the same subjects). Tissue concentration of tenofovir and tenofovir diphosphate were higher than the oral dosing-related tissue concentrations (as seen in the vaginal dosed PK reports below). These studies are completed, but complete analysis is pending [44].

2.5.2.2 Reduced Glycerin Vaginal Formulation (RGVF) of Tenofovir 1% Gel

CONRAD holds the IND for the RGVF of tenofovir 1% gel (IND 73,382) and provided a cross-reference letter. The VF gel is covered under the same IND and is referenced in the same letter.

2.5.2.2.1 Vaginal <u>and</u> Rectal Administration

No vaginal administration studies have been planned for evaluation of RGVF. Two Phase 1 rectal-administration studies will evaluate the RGVF tenofovir 1% gel. MTN-007 is currently exploring this formulation, and a second study called Project GEL is planned for initiation.

2.5.2.3 Rectal Formulation (RF) of Tenofovir 1% Gel

2.5.2.3.1 Vaginal <u>and</u> Rectal Administration

Since the RF formulation is strictly for rectal for use, no vaginal administration studies will be conducted. CHARM-01 and a companion study CHARM-02 will be the first rectal safety studies of this new formulation.

2.5.2.4 Universal HEC Placebo Gel

Unformulated hydroxyethylcellulose is known to be a non-irritating substance in humans (skin sensitization is unusual), with doses less than 2 g/kg by ingestion not expected to be toxic [77]. No inhalation studies have been conducted, but exposure of humans to the dust in manufacturing operations over many years has not led to any known adverse effects.

<u>Safety – Vaginal Administration</u>

The hydroxyethylcellulose placebo formulation was developed and adopted for use in the HPTN 035 microbicide study, the Phase II/IIb Safety and Effectiveness Study of the Vaginal Microbicides Buffer Gel and 0.5% PRO2000/5 Gel (P) for the Prevention of HIV Infection in Women.

A randomized, closed label, Phase I study of daily vaginal Universal HEC Placebo Gel exposure was conducted in 2003 [78]. In this trial, 30 women were randomized to twice-daily vaginal applications of 3.5 mL of the Universal HEC Placebo Gel or polystyrene sulfonate (PSS) vehicle. The primary objective of this study was to assess and compare the effects of the test articles on symptoms and signs of irritation of the external genitalia, cervix, and vagina as seen on naked eye exam after 7 and 14 days of use including disruption of the epithelium and blood vessels as seen on colposcopy after 14 days of use. Secondary objectives included: an assessment and comparison of differences in vaginal health by evaluating the results of wet mounts, pH, and Gram-stained vaginal smears (Nugent score and neutrophil counts) after 7 and 14 days of use and vaginal cultures after 14 days of use; and an assessment of acceptability of the study products after 14 days of use among participants.

Results of this trial indicated that both gels appear safe for vaginal use twice a day for 14 days in sexually abstinent women. Two out of 14 women (14.3%) randomized to the HEC group reported at least one symptom of mild severity of genital irritation, which included genital burning, soreness and pelvic pain. Three out of 14 women in the HEC group (21.4%) had colposcopic findings that included erythema, petechiae and peeling [78]. No deep genital disruption was observed in either product group. Minimal changes in wet mounts, pH, Nugent scores, neutrophils, and vaginal flora were observed in both product groups.

<u>Safety – Rectal Administration</u>

Two Phase 1 rectal microbicide trials completed to date have used the Universal HEC Placebo Gel in the placebo arm. RMP-01 using topical UC781 enrolled 36 subjects with 12 randomized to the HEC placebo arm. Following single topical rectal HEC exposure, four Grade 2 AEs were reported in 1 individual, shown to be in the placebo group. As this individual had no complaints during the subsequent 7-day exposure, the symptoms of fever, cramps, flatulence, and diarrhea were likely not related to the HEC gel. There were no other Grade 2 or Grade 3 AE associated with HEC gel [32]. In the second rectal microbicide Phase 1 safety trial, RMP-02/MTN-006, 18 subjects were studied with 6 randomized to the HEC placebo arm in the topical-exposed portion of the trial. With regards to gastrointestinal-related AEs following single-dose topical HEC exposure, no Grade 2 or Grade 3 AEs were reported. Following 7-day exposure, one Grade 2 event was reported in one subject receiving HEC gel (abdominal pain); three events were reported in 2 subjects in the tenofovir-treated group. None of the five gastrointestinal Grade 3

AEs reported in 2 subjects (all during 7-day exposure) were related to HEC placebo; all were in the tenofovir-product group. No SAEs were reported.

2.5.2.5 Summary

While the safety data suggest an overall favorable safety and effectiveness profile for tenofovir 1% gel, further studies must be done to assess whether RF formulation of tenofovir 1% gel is safe, well-tolerated, and efficacious when administered rectally. As previously mentioned, CHARM-01 and a companion study CHARM-02 will be the first rectal safety studies of the new rectal specific formulation (RF) of tenofovir 1% gel.

2.5.3 Drug Resistance

In HPTN 050, no new resistance mutations evolved in plasma or cervicovaginal lavage (CVL) after 14 days of tenofovir gel use, but 3 women had plasma mutations associated with low level tenofovir resistance identified at both Day 0 and Day 14 (M41L, L210M, ±T215I/Y) [59].

2.5.4 Other Studies of Tenofovir for HIV Prevention

Several other studies of the safety and/or effectiveness of topical tenofovir 1% gel as an HIV prevention strategy are summarized below in Table 6.

Table 6: Other Studies of Tenofovir for HIV Prevention

Location	Sponsor	Population	Design	Formulation and Route of Administration
USA, Dominican Republic	CONRAD A04-095/IND 73,382	Sexually abstinent women	PK study; single dose and 14-day once or twice-daily.	VF, vaginal administration
South Africa, Uganda, USA	DAIDS/MTN-001/IND 55,690	Sexually active women	Phase 2 Adherence and Pharmacokinetics Study of Oral and Vaginal Preparations of Tenofovir	VF, vaginal administration;<br><br>Oral preparation, by mouth
USA	DAIDS IPCP/ RMP-02/MTN-006/ CONRAD IND 73,382	Sexually abstinent (for active phases of study and for 5 days following biopsy collection) women and men	Phase 1 Rectal PK and Acceptability	VF, rectal administration
Malawi, South Africa, Uganda, Zimbabwe,	(MTN-003/VOICE Study) NIH	5029 high-risk women	TDF topical<br>TDF oral<br>FDC/TDF oral	VF, vaginal administration;<br><br>TDF oral, by mouth

Studies examining the safety and/or effectiveness of oral formulations of tenofovir as a prevention strategy are summarized in Table 7 below.

Table 7: PrEP Studies

Location	Sponsor	Population	PrEP Strategy
**Phase 2**			
West Africa (Ghana, Nigeria, Cameroon)	Family Health International	936 high-risk women	TDF
United States	Centers for Disease Control and Prevention (CDC)	400 MSM	TDF
Malawi, South Africa, Uganda, Zimbabwe,	(MTN-003/VOICE Study) NIH	5029 high-risk women	TDF topical<br>TDF oral<br>FDC/TDF oral
**Phase 3**			
Thailand	CDC	2,000 injection drug users (~20% women)	TDF
Botswana	CDC	1,200 men and women	FTC/TDF
Peru, Ecuador, Brazil, Thailand, South Africa, United States	(iPrEx Study) NIH	1,400 MSM (potential expanded sample size of 3,500)	FTC/TDF
Africa	FHI	3,800 high-risk women	FTC/TDF
Africa	University of Washington, Gates Foundation	3,900 HIV-1 seronegative partners within HIV-1 discordant couples	FTC/TDF<br>TDF

The recently published results of the Pre-Exposure Prophylaxis Initiative (iPrEx) study were extremely encouraging that orally administered tenofovir in combination with emtricitabine prevents HIV infection [63]. In this randomized double-blind, placebo-controlled clinical study of a daily oral dose of Truvada (tenofovir disoproxil fumarate/emtricitabine fixed dose combination) in 2,499 highly sexually active MSM, the Truvada arm had a 44% reduction (95% confidence interval 15% to 63%). This is highly relevant for development of rectal microbicides as rectal tissue concentrations are anticipated to be higher after topical than oral dosing as is the case for vaginal compared to oral dosing.

In a recently completed unpublished study (MTN-001, unpublished, Hendrix, data on file) in which women received daily tenofovir by oral, vaginal, and both routes in 3 distinct 6 week periods, vaginal dosing resulted in tissue TFV diphosphate (TFV-DP) concentrations more than one hundred times greater than oral dosing [60]. This gradient is anticipated to be even greater with rectal dosing (with respect to rectal tissue TFV-DP) due to histologic differences between vaginal and rectal tissue, which favor drug penetration of rectal tissue. These data will shortly be available from the just completed RMP-2/MTN-006 trial [44].

In another unpublished study using a single oral dose of TDF given to sexually-active women, rectal concentrations exceeded vaginal concentrations of TFV-DP by 200-fold, suggesting differences intrinsic to the tissue, apart from mucosal permeability

differences may also favor rectal tissue concentration of drug (^{14}C-Tenofovir Study, unpublished, Hendrix, data on file).

2.6 Justification of Dosing

The choice of the tenofovir 1% gel concentration is based on both animal and clinical evidence described above, suggesting an appropriate safety profile and potency. Animal and human studies have demonstrated minimal vaginal irritation at this concentration. Rectal administration has demonstrated efficacy in a macaque model [58]. Finally, limited vaginal PK tenofovir data in nonhuman primates demonstrate that tenofovir gel is broadly distributed in vaginal tissues following vaginal application and can penetrate to epithelial tissues [50]. There are no published studies of drug penetration into human colonic tissue after either rectal or oral administration of tenofovir, though these studies have recently been completed and samples are currently undergoing processing and analyses. Preliminary data from RMP-02/MTN-006 indicates that tenofovir and tenofovir diphosphate were detected in the tissue and intracellularly with the current validated assays [44], supporting the feasibility and utility of this planned study.

With regard to timing, in a single dose study of tenofovir disoproxil fumarate tablets or TDF (VIREAD®, currently-marketed oral dosage form) in healthy volunteers, both median plasma TFV and PBMC TFV-DP concentrations are very near, but slightly above using liquid chromatography-tandem mass spectrometry (LC/MS/MS) detection limits on the 8th day (192 hours) after dosing, but not thereafter. At 8 days, values are 1/100th and 1/5th of peak concentrations for TFV and TFV-DP, respectively. By the 11th day, no subjects had TFV or TFV-DP concentrations above detection limits.

2.7 Justification of Sampling Time Points

First dose of each gel formulation will be administered in clinic by the study site investigator or the designated study staff, and five doses will be dispensed to the participants for take-home use. The take-home use period will be followed by another clinic visit for administration of the seventh dose of the study product and various post-dose specimen collections. Compartmental tenofovir PK during the 24 hours post administration of the seventh dose of each formulation will be characterized using a sampling strategy that includes blood (plasma and intracellular PBMC), rectal secretions, cervicovaginal secretions for women, and tissue (whole and MMC). Blood samples for plasma and intracellular PBMC PK will be drawn at pre-dose (Visit 2) and then at 0.5-hr (+/-15min), 2-hr (+/-30min), 4-hr (+/-30min), and 24-hr (-18/+30hr) after the seventh dose of each product. To complement blood PKs, while minimizing repeated mucosal sampling, rectal fluid and vaginal fluid (for female participants) will also be collected at pre-dose (Visit 2), 0.5 hr, 2hr, 4hr, and 24-hr.

One flexible sigmoidoscopy with biopsies will be performed at approximately 30 minutes post administration of the seventh dose of each study product (at Visits 4, 7, and 10). At each time point incorporating a flexible sigmoidoscopy (Visit 2/Baseline, Visit 4, Visit

7, and Visit 10), samples will be obtained for PK, as described above, as well as for histology, cytokine mRNA, explant infection assays, and flow cytometry, assessing changes in mucosal CD4+ surface phenotypes.

After the seventh dose exposures of each gel formulation, a window of approximately 2-4 weeks will be incorporated for mucosal healing before exposure to another formulation. As TFV has ~17-hour plasma half-life and TFV diphosphate has a half life of 4-5 days, no detectable levels are anticipated after 1 week. Given the 2 week window, confounding TFV or TFV diphsophate from the prior exposure is highly unlikely.

As rectal microflora and rectal fluid cytokine protein levels can change rapidly, specimen for these indices will be collected at baseline of each formulation (Visits 3, 6, and 9) immediately prior to insertion of the first dose of each product and at 24 hr post seventh dose exposure of each product.

3 OBJECTIVES

3.1 Primary Objectives

- To evaluate the safety of each tenofovir-based microbicide gel formulation when applied rectally
- To evaluate the acceptability of each tenofovir-based microbicide gel formulation when applied rectally
- To compare systemic and compartment pharmacokinetics among the three tenofovir-based microbicide gel formulations when applied rectally

3.2 Secondary Objectives

- To evaluate the mucosal immunotoxicity of each tenofovir-based microbicide gel formulation when applied rectally

3.3 Exploratory Objectives

- To assess the preliminary (*ex vivo)* efficacy of each tenofovir-based microbicide gel formulation using biopsy explants after each product is applied rectally

4 STUDY DESIGN

4.1 Identification of Study Design

A double-blinded, randomized, safety, acceptability, pharmacokinetic, and ex vivo efficacy study of 3 rectally applied tenofovir-based microbicide formulations: a RF, a VF, and a reduced glycerin vaginal formulation (RGVF). Each participant will experience seven rectal exposures to the rectal-specific formulation (RF) and seven rectal

exposures to the reduced glycerin vaginal formulation (RGVF) of tenofovir 1% gel, but only one exposure to the vaginal formulation (VF), which will be coupled with six preceding exposures to the Universal HEC Placebo Gel to balance out the VF study stage. After completing a screening evaluation (Visit 1), participants will return to clinic for a baseline evaluation (Visit 2), including flexible sigmoidoscopy, baseline behavioral questionnaire, and sample collection, and then be randomized to study product sequence. With 21 days +/- 7 days washout/rest period between each stage, participants will return to the clinic for three study stages, called *Stage 1-Administration of First Product*, *Stage 2 – Administration of Second Product*, and *Stage 3 – Administration of Third Product*. Each stage will begin with the first dose administered in clinic (Visits 3, 6, and 9) during which the participant will receive a single rectally-applied dose of one of the study products in a double-blinded, randomized sequence and will then have 5 doses of the same product dispensed for take-home use. After completing the take-home use period, participant will return to clinic for the administration of the seventh dose of the same formulation, with various specimen collections at the following time points: pre dose, 0.5-hr post-dose, 2-hr post-dose, 4-hr post-dose, and 24-hr post-dose; acceptability assessments will be conducted after the take-home use period as well. The sequence of visits will be repeated for each gel formulation. A more detailed summary of sample collection can be found in Appendix I and Table 4.

Figure 1: Study Schema

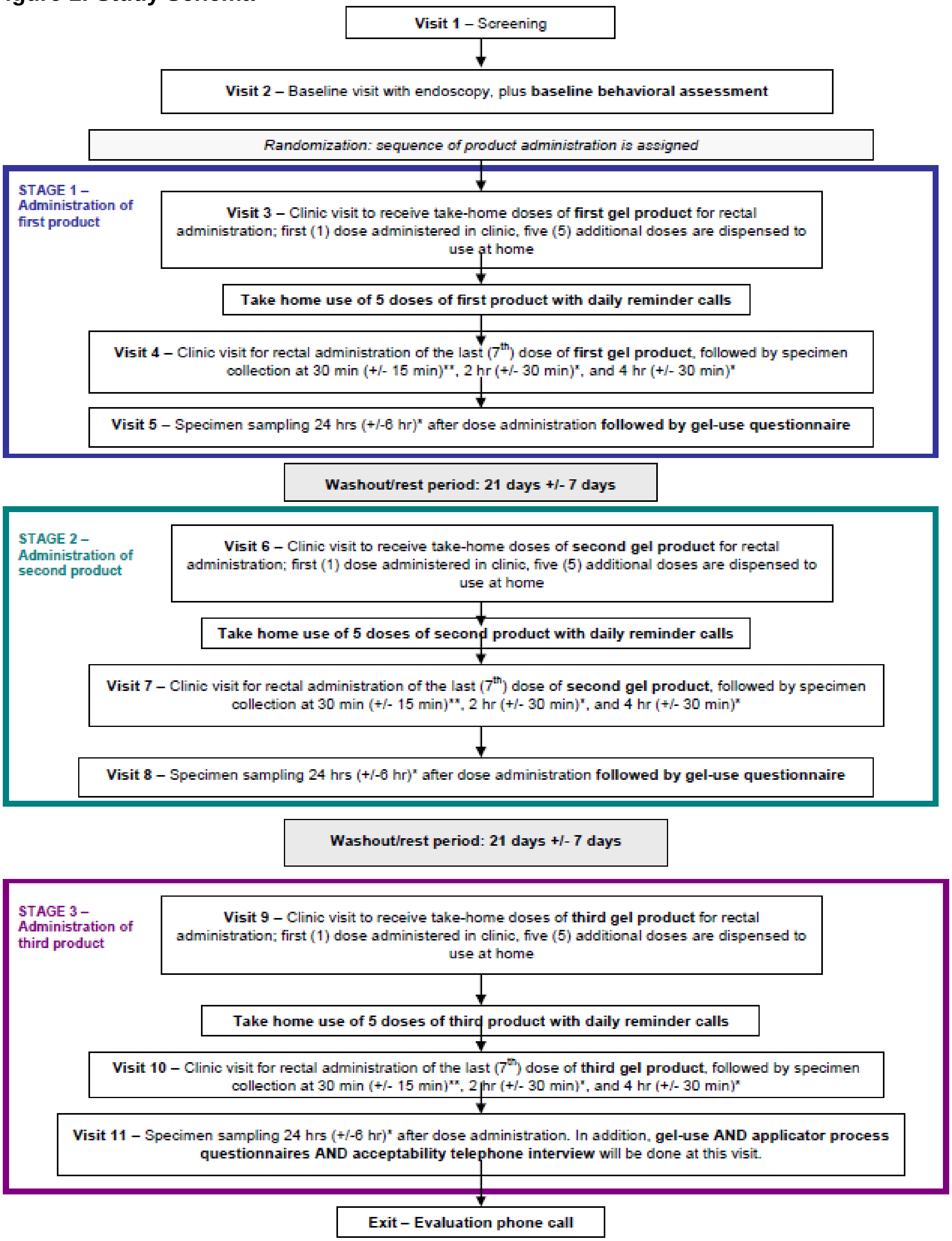

* Only blood and vaginal and rectal fluids are collected

** This time-point includes biopsy collection

4.2 Summary of Study Endpoints

4.2.1 Primary Endpoints

Safety

- Grade 2 or higher clinical and laboratory adverse events as defined by the *Division of AIDS Table for Grading the Severity of Adult and Pediatric Adverse Events, Version 1.0, Dec 2004* and *Addenda 1 and 3 (Female Genital and Rectal Grading Tables for Use in Microbicide Studies)* to this table.

Acceptability

- Product attributes considered likely to challenge future sustained use by participants.

Pharmacokinetics

- Tenofovir concentrations
    - Plasma
    - PBMC (intracellular)
    - Rectal fluid
    - Vaginal fluid, in female participants
    - Rectal mucosal tissue homogenates
    - Rectal MMC

- Tenofovir diphosphate concentrations
    - PBMC
    - Rectal mucosal tissue homogenates
    - Rectal MMC

4.2.2 Secondary Endpoints

- Rectal microflora
- Rectal cytokines (secreted and mRNA)
- Rectal histology
- Rectal CD4 cell phenotype/activation

4.2.3 Exploratory Endpoints

- Changes in HIV-1 p-24 levels in colorectal explant supernatant

4.3 Description of Study Population

This study will include HIV-negative men and women who meet the criteria outlined in Sections 5.4 and 5.5.

4.4 Time to Complete Accrual

Accrual is expected to be completed within approximately 6 months.

4.5 Expected Duration of Participation

Participant accrual will take approximately 6 months. Each participant will be on study for approximately 3 months. The total duration of the study will be approximately 1 year.

5 STUDY POPULATION

5.1 Selection of Study Population

The inclusion and exclusion criteria outlined in Sections 5.4 and 5.5 will be utilized to ensure the appropriate selection of study participants.

5.2 Recruitment

Participants will be recruited from a variety of sources, using the following key strategies:

- Clinician-patient referrals
- Use of existing "study registries" that contain the names and phone numbers of individuals who have given informed consent to be reached for future studies for which they may be eligible
- Participant referrals (participants refer their friends or partners who may meet eligibility criteria)
- Passive self-referral: interested individuals see a study poster or brochure advertising the study and call the study site directly

Study staff will meet as needed to discuss current recruitment status, targets, and strategies. Staff also will follow-up with all persons who express an interest in the study to ensure that screening appointments are scheduled and carried out in a timely manner.

5.3 Retention

Once participants enroll in this study, the study site will make every effort to retain them for the duration of follow-up in order to minimize possible bias associated with loss-to-follow-up. The study staff is responsible for developing and implementing local standard operating procedures to target this goal. Components of such procedures include:

- Thorough explanation of the study visit schedule and procedural requirements during the informed consent process, and re-emphasis at each study visit
- Thorough explanation of the importance of all three dosing and sampling phases to the overall success of the study
- Use of appropriate and timely visit reminder mechanisms (via email and/or telephone)
- Immediate and multifaceted follow-up on missed visits

5.4 Inclusion Criteria

Individuals who meet the following criteria are eligible for inclusion in the study:

1. ≥ Age of 18 at screening
2. Willing and able to communicate in English
3. Willing and able to provide written informed consent to take part in the study
4. Willing and able to provide adequate locator information
5. Understands and agrees to local sexually transmitted infection (STI) reporting requirements
6. HIV-1 uninfected at screening according to the standard DAIDS algorithm in Appendix II
7. Must have been vaccinated for or have natural immunity to Hepatitis B, which will be verified by a positive Hepatitis B surface antibody (HBsAb) test at screening

   Note: One re-screen will be allowed for individuals who are non immune to Hepatitis B but undergo vaccination.

8. Availability to return for all study visits, barring unforeseen circumstances
9. Willing to abstain from RAI and practices involving insertion of anything in rectum (drug, enema, penis, or sex toy) for 72 hours before and 72 hours after each flexible sigmoidoscopy and study product exposure.
10. Must agree to use study provided condoms for the duration of the study
11. Must be in general good health
12. Must agree not to participate in other concurrent interventional and/or drug trials
13. Per participant report at screening, a history of consensual RAI at least once in the last three months.

In addition to the criteria listed above, female participants must meet the following criteria:

14. Willing to abstain from insertion of anything into vagina (drug, douche, penis, or sex toy) other than the swabs/sponges for study related specimen collection for 24 hours before and after each study product exposure

15. Post-menopausal or using (or willing to use) an acceptable form of contraception (e.g., intrauterine device (IUD), hormonal contraception, surgical sterilization, or vasectomization of male partner). If the female participant has female partners only, the method of contraception will be noted as a barrier method in the study documentation.

5.5 Exclusion Criteria

Individuals who meet any of the following criteria at screening will be excluded from the study:

1. Abnormalities of the colorectal mucosa, or significant colorectal symptom(s), which in the opinion of the clinician represents a contraindication to biopsy (including but not limited to presence of any unresolved injury, infectious or inflammatory condition of the local mucosa, and presence of symptomatic external hemorrhoids).

2. At screening: participant-reported symptoms and/or clinical or laboratory diagnosis of active rectal or reproductive tract infection requiring treatment per current CDC guidelines or symptomatic urinary tract infection (UTI). Infections requiring treatment include symptomatic bacterial vaginosis, symptomatic vaginal candidiasis, other vaginitis, trichomoniasis, Chlamydia (CT), gonorrhea (GC), syphilis, active HSV lesions, chancroid, pelvic inflammatory disease, genital sores or ulcers, cervicitis, or symptomatic genital warts requiring treatment. Note that an HSV-1 or HSV-2 seropositive diagnosis with no active lesions is allowed, since treatment is not required.

   Note: In cases of non-anorectal GC/CT identified at screening, one re-screening 2 months after screening visit will be allowed

3. Per participant report and/or clinical or laboratory diagnosis, anorectal STI within six months prior to the Screening Visit

4. At screening:
   a. Hemoglobin < 10.0 g/dL
   b. Platelet count less than 100,000/mm^3
   c. White blood cell count < 2,000 cells/mm^3 or > 15,000 cells/mm^3
   d. *For females:* calculated creatinine clearance less than 60 mL/min by the Cockcroft-Gault formula where creatinine clearance in mL/min (140- age in years) x (weight in kg) x (0.85 for female)/72 x (serum creatinine in mg/dL)
   e. *For males:* calculated creatinine clearance less than 60 mL/min by the Cockcroft-Gault formula where creatinine clearance in mL/min = (140 - age in years) x (weight in kg) x (1 for male)/72 x (serum creatinine in mg/dL)
   f. Serum creatinine > 1.3× the site laboratory upper limit of normal (ULN)

    g. Alanine transaminase (ALT) and/or aspartate aminotransferase (AST) > 2.5× the site laboratory ULN
    h. +1 glucose or +1 protein on urinalysis (UA)
    i. History of bleeding problems (verified via prothrombin time (PT)/ International Normalized Ratio (INR) test)
    j. Positive for Hepatitis B surface antigen (HBsAg)

5. History of significant gastrointestinal bleeding in the opinion of the investigator

6. Known allergic reaction to methylparaben, propylparaben, sorbic acid, glycerin, glycerol, or tenofovir

7. Current known HIV-infected partner(s)

8. By participant report at enrollment, history of excessive daily alcohol use (as defined by the CDC as heavy drinking consisting of an average consumption of more than 2 drinks per day for men, and more than 1 drink per day for women), frequent binge drinking or illicit drug use that includes any injection drugs, methamphetamines (crystal meth), heroin, or cocaine including crack cocaine, within the past 12 months

9. Per participant report at screening, anticipated use and/or unwillingness to abstain from the following medications during the period of study participation:
    a. Heparin, including Lovenox®
    b. Warfarin
    c. Plavix® (clopidogrel bisulfate)
    d. Rectally administered medications (including over-the-counter products)
    e. Acyclovir, valacyclovir, famciclovir, and TDF
    f. Aspirin
    g. Non-steroidal anti-inflammatory drugs (NSAIDS)
    h. Any other drugs that are associated with increased likelihood of bleeding following mucosal biopsy

10. By participant report at screening, use of systemic immunomodulatory medications, rectally administered medications, rectally administered products (including condoms) containing N-9, or any investigational products within the 4 weeks prior to the Enrollment/Baseline Evaluation Visit and throughout study participation

11. History of recurrent urticaria

12. Any other condition or prior therapy that, in the opinion of the investigator, would preclude informed consent, make study participation unsafe, make the individual unsuitable for the study or unable to comply with the study requirements. Such conditions may include, but are not limited to, current or recent history of severe, progressive, or uncontrolled substance abuse, or renal, hepatic, hematological, gastrointestinal, endocrine, pulmonary, neurological, or cerebral disease

In addition to the criteria listed above, female participants will be excluded if they meet any of the following criteria:

13. Pregnant at the Enrollment/Baseline Visit

14. Breastfeeding at screening or intend to breastfeed during study participation per participant report.

6 STUDY PRODUCTS

6.1 Regimen

Each participant will receive seven rectally-administered doses of the rectal-specific formulation (RF) and seven rectal exposures to the reduced glycerin vaginal formulation (RGVF) of tenofovir 1% gel, but only one dose of the vaginal formulation (VF), which will be coupled with six preceding exposures to the Universal HEC Placebo Gel to balance out the VF study stage. The products will be administered in a double-blinded randomized sequence with approximately 2-4 weeks between each study stage.

6.2 Administration

During each study stage, two doses – the first and the seventh – will be administered by a study site investigator or a designated sub-investigator and 5 doses will be dispensed for take-home use. Any deviations or occurrence of note during product administration will be noted in the participant study record and *Product Use Log*, if applicable.

6.3 Study Product Formulation

6.3.1 Vaginal Formulation

The original VF is a transparent, viscous gel formulation containing 1% (weight/weight or w/w) of tenofovir (PMPA, 9-[(R)-2-(phosphonomethoxy) propyl]adenine monohydrate), formulated in purified water with edetate disodium, citric acid, glycerin, methylparaben, propylparaben, hydroxyethylcellulose, and pH adjusted to 4-5. This formulation has been used in all clinical trials (vaginal, penile, and rectal) of tenofovir 1% gel to date. One dose of this formulation will be used, with 6 doses of the HEC placebo gel preceding it to balance out this study stage.

6.3.2 Reduced-Glycerin Vaginal Formulation

The RGVF is also a transparent, viscous gel formulation containing 1% (w/w) of tenofovir (PMPA), formulated in purified water with edetate disodium, citric acid, glycerin, methylparaben, propylparaben, hydroxyethylcellulose, and pH adjusted to 4-5. However, the RGVF has lower glycerin content than the VF and a significantly reduced osmolality (836 or 846 versus 3111 mOsmol/kg). Lowering the glycerin

content lowered the viscosity, so the HEC concentration was increased by 10% (a change considered to be insignificant). The amount of parabens was increased by 10% each to improve the antimicrobical effectiveness. The RGVF formulation with 2.75% HEC was used in MTN-007 (CONRAD IND 73,382; currently enrolling), which is the only clinical study of this formulation. The RGVF formulation has since been modified to increase the viscosity. The revised RGVF is the only formulation that will be made going forward, including the clinical supplies that are being made for CHARM-01. Differences between the original VF and the RGVF gels are available in Table 8 below.

Table 8: Comparison of Tenofovir 1% Gel Formulations (HEC placebo gel included as well)

Chemical Name	Original VF % w/w	RGVF % w/w (used in MTN-007)	RGVF % w/w (to be used in CHARM-01)	RF % w/w	Universal HEC Placebo gel
Glycerin	20.00	**5.00***	**5.00**	**2.50**	**0**
Hydroxyethyl cellulose	2.50	**2.75**	**3.00**	**0**	**2.7**
Carbopol 974	0	0	0	**0.50**	0
Sodium carboxymethylcellulose	0	0	0	**1.00**	0
Methylparaben	0.18	**0.22**	**0.22**	0.18	**0**
Propylparaben	0.02	**0.024**	**0.05**	0.02	**0**
Purified water	75.23	**89.936**	**89.66**	**94.78**	**96.34**
Disodium edetate (EDTA)	0.05	0.05	0.05	**0.01**	**0**
Citric acid	1.00	1.00	1.00	**0**	**0**
PMPA	1.00	1.00	1.00	1.00	**0**
Sodium hydroxide	As needed	As needed	As needed	As needed	As needed
Diluted hydrochloric Acid	As needed	As needed	As needed	As needed	As needed
pH	4.5	**4.6**	4.5	**7**	**4.4**
Osmolality (mOsmol/kg)	3111	**836**	**846**	**479**	**304**

***Differences between original VF and other formulations are in bold**

6.3.3 Rectal Specific Formulation

The RF is a translucent colorless viscous gel formulation containing 1% (w/w) of tenofovir (PMPA) formulated in purified water with EDTA, glycerin, methylparaben, propylparaben, carbopol, sodium carboxy methyl cellulose, and pH adjusted to 7. The RF is close to isoosmolar with an osmolality of 479 mOsmol/kg. Formulation details are shown in Table 8. Seven doses of this formulation will be used.

6.3.4 Universal HEC Placebo Gel Formulation

The Universal HEC Placebo Gel contains hydroxyethylcellulose as the gelling agent, purified water, sodium chloride, sorbic acid and sodium hydroxide [79]. The gel is isotonic and formulated at a pH of 4.4 to avoid disrupting the normal vaginal pH and has minimal buffering capacity to avoid the inactivation of sexually transmitted pathogens. Hydroxyethylcellulose is used to approximate the viscosity of microbicide

gel candidates. Each pre-filled applicator will deliver approximately 4 mL of HEC placebo gel.

6.4 Study Product Supply and Accountability

6.4.1 Study Product Supply

The vaginal formulation (VF) of the tenofovir 1% gel, the reduced glycerin vaginal formulation (RGVF) of the tenofovir 1% gel, and the Universal HEC placebo gel will be manufactured under direction from CONRAD (Arlington, VA, USA) by DPT Laboratories, San Antonio, TX USA, which is a contract manufacturing facility (CONRAD IND 73,382: complete information is available in Chemistry, Manufacturing and Controls Section and authorization of IND review is stated CONRAD's cross-reference letter.) DPT Laboratories will conduct on-going stability and microbiologic testing for the finished products post fill. DPT Laboratories will fill the applicators with the reduced glycerin vaginial formulation of tenofovir 1% gel to create pre-filled applicators and package each applicator and plunger in a wrapper.

DPT Laboratories will also manufacture the tenofovir 1% rectal-specific gel formulation (RF) under direction from Dr. Lisa Rohan's Group at MWRI. DPT Laboratories will manufacture each tenofovir 1% gel formulation, analyze/release the gels under cGMP, and fill the applicators to create pre-filled opaque applicators. The manufacturing information related to the RF formulation of tenofovir 1% gel is available in Chemistry, Manufacturing and Controls Section of the RF IND application. DPT Laboratories will conduct on-going stability and microbiologic testing for the finished products post fill. DPT Laboratories will fill the applicators with the tenofovir 1% rectal-specific gel formulation to create pre-filled applicators and package each applicator and plunger in a wrapper.

The applicators being used in CHARM-01 were initially designed for vaginal use and have been used in all of the previous vaginal microbicide trials with tenofovir 1% gel. They have also been used rectally in the RMP-02/MTN-006 rectal Phase 1 study of tenofovir 1% gel. Each opaque pre-filled applicator will be packaged with a plunger in a wrapper and labeled with a code to preserve the identity of the formulation. Each pre-filled applicator will contain and deliver a dose of approximately 4 mL of tenofovir 1% gel. The pre-filled applicators will be shipped directly to the pharmacy at each study site and will be stored and dispensed from the site pharmacy.

6.4.2 Study Product Receipt

The study site pharmacist is required to maintain complete records of all study products received.

6.4.3 Storage

Study gel must be stored at controlled room temperature, 25°C (77°F), at all times. Excursions are permitted between 15°C and 30°C (59°F and 86°F).

Storage conditions for protocol-provided study products will include segregation, security, temperature monitoring, and sanitation. Study products should be stored in a limited access area that is locked when not in use. The study products should be accessible only to authorized personnel.

6.4.4 Dispensing

Study products will be dispensed from the pharmacy to study staff for an enrolled participant only upon receipt of a written prescription from an authorized prescriber. Study gel will be administered by the study site investigator or the designated study staff using product dispensed by the pharmacy. Participants will receive a vaginal formulation (VF), a reduced-glycerin vaginal formulation (RGVF), and a rectal-specific formulation RF in a double-blinded sequence. Each prefilled applicator will be dispensed at the designated study visit.

6.4.5 Retrieval of Unused Product

Any dispensed but unused product will be handled according to the appropriate site and pharmacy procedures and quarantined within 24 hours.

6.4.6 Accountability

The pharmacist at each study site is required to maintain a complete record of all study products received from the manufacturer and subsequently dispensed. At the end of the study, specific instructions will be provided for the return or destruction of the study products.

6.5 Adherence

Study product will be administered during inpatient clinic visits by the study site investigator or a designated sub-investigator. Anything of note during this process will be recorded in the source documents.

6.5.1 Evaluation of Adherence

Wisebag™

Wisebag™ (Wisepill Technologies Adherence Management Solutions, South Africa) is a portable, lunch-bag size container, and has a self-contained battery-operated electronic device that can send a real-time signal to a central management system through a wireless system. Through this latter modality, it is possible to monitor adherence on an ongoing, 'real time' basis. This study will include the use of a Wisebag for all home administrations. Wisebag will provide an alternative measure of

adherence to the study products compared to self-reported adherence, and it will provide the opportunity to evaluate the usability of Wisebag-technology in future rectal microbicide trials [69, 70].

6.6 Concomitant Medications and Procedures

With the exception of medications listed as prohibited, enrolled study participants may use concomitant medications during study participation. All concomitant medications, over-the-counter preparations, vitamins and nutritional supplements, recreational drugs, and herbal preparations reported throughout the course of the study will be recorded in the source documents and on case report forms designated for that purpose.

6.7 Prohibited Medications and Procedures

Study participants will be prohibited from using the following medications throughout the study period: TDF, acyclovir, valacyclovir, famciclovir, heparin (including Lovenox®), warfarin, Plavix® (clopidogrel bisulfate), rectally administered medications (including over-the-counter products), aspirin or NSAIDS, and other drugs that are associated with increased likelihood of bleeding following mucosal biopsy.

Participants will be advised to refrain from RAI or any practices which include rectal insertion of anything (drug, enema, penis, or sex toy) for 72 hours before and 72 hours after each flexible sigmoidoscopy and study product exposure. Female participants will be advised to refrain from any practices, which include vaginal insertion of anything (drug, douche, penis, or sex toy) for 24 hours before and after each study product exposure. Furthermore, study participants will be advised not to use the following products within 2 weeks or 6 biological half-lives of the drug, whichever is longer, of enrollment and throughout the study period: systemic immunomodulatory medications, rectally administered medications, rectally administered products containing N-9, or any other investigational products.

If participants report using any of these medications or products while on study, their study product administration will be put on hold, pending discussions with the study site investigators, Sponsor, and/or DAIDS MO.

6.8 Required Medications and Procedures

Latex male condoms are recommended for use by participants enrolled in this study. Study sites will provide latex male condoms to participants in quantities expected to be sufficient for the study period. These male condoms will not be impregnated or coated with any type of spermicide. Male condoms will be recommended for all sexual encounters during the study period. In the event that a participant needs additional male condoms between visits, he or she may request these from study sites at any time. Participants will also be provided with a list of approved brands that can be used in place of condoms provided by study to help encourage condom use.

7 STUDY PROCEDURES

This section includes information on visit-specific study procedures. An overview of the study visits and evaluations is available in Appendix I. Detailed instructions to guide and standardize sample collection are provided in Section 7.15 and the study-specific procedures manual. Unless otherwise specified, the laboratory procedures listed in this section are performed at the local study site laboratories. All visits for female participants should be scheduled to best avoid menses.

In addition to any Interim Visits that may occur in accordance with guidance outlined in Section 7.9, the following visits should take place for study participants:

**Visit 1:**	Screening Visit
**Visit 2:**	Enrollment/Baseline Visit
**Visit 3:**	First Dose of 1st Formulation
**Take Home Use:**	Five Doses of 1st Formulation Used at Home
**Visit 4:**	Seventh Dose of 1st Formulation
**Visit 5:**	24 hr Post Seventh Dose of 1st Formulation
**Visit 6:**	First Dose of 2nd Formulation
**Take Home Use:**	Five Doses of 2nd Formulation Used at Home
**Visit 7:**	Seventh Dose of 2nd Formulation
**Visit 8:**	24 hr Post Seventh Dose of 2nd Formulation
**Visit 9:**	First Dose of 3rd Formulation
**Take Home Use:**	Five Doses of 3rd Formulation Used at Home
**Visit 10:**	Seventh Dose of 3rd Formulation
**Visit 11:**	24 hr Post Seventh Dose of 3rd Formulation
**Exit:**	Termination Phone Call

Participants will have 21 days +/- 7 days washout/rest period between Visits 5 and 6, and between Visits 8 and 9.

Prescreening via a phone call will take place with potential participants, and those interested in the study can request that the informed consent documents be mailed out to them prior to the Screening Visit.

7.1 Visit 1: Screening

Written informed consent will be obtained before any procedures are initiated. For participants who do not meet the eligibility criteria, screening will be discontinued once ineligibility is determined. Participants not meeting the eligibility criteria may be rescreened at a later date, if appropriate, as in the case of a medication washout period.

Table 9: Visit 1 (Screening)

Component	Procedure/Analysis
**Administrative**	• Obtain written informed consent<br>• Assign participant ID (PTID)<br>• Assess eligibility<br>• Obtain locator information
**Clinical**	• Record medical history<br>• Record menstrual history of female participants<br>• Record concomitant medications<br>• Perform physical exam, including digital rectal exam<br>• Provide counseling and condoms<br>o Contraception<br>o Condom use, HIV/STI risk reduction<br>o HIV pre-and post-test
**Blood Specimens (~30 mL)**	• Collect blood specimens for<br>o CBC with differential and platelets<br>o PT/INR<br>o Blood urea nitrogen (BUN), creatinine, alanine transaminase (ALT), aspartate aminotransferase (AST)<br>o Hepatitis B Surface Antibody (HBsAb)<br>o Hepatitis B Surface Antigen (HBsAg)<br>o Syphilis rapid plasma reagin (RPR) (confirmatory tests as needed)<br>o HSV serologies (HSV 1 and 2 antibody testing)<br>o HIV serostatus (confirmatory tests as needed)<br>o Plasma archive
**Urine Specimens**	• Collect urine sample for<br>o Dipstick Urinalysis (UA) for protein, glucose, nitrites, and leukocyte esterase<br>o GC/CT by Nucleic acid amplification test (NAAT)<br>o Qualitative hCG for females of childbearing potential
**Vaginal Specimens♀**	• Self-collected vaginal swabs for<br>o Vaginal pH for BV assessment<br>o Vaginal swab for Gram stain for BV assessment
**Rectal Specimens**	• Collect rectal swabs for GC/CT (NAAT)

♀ For female participants

7.2 Visit 2: Enrollment/Baseline

Visit 2 must take place no more than 28 days following Visit 1; after this period, participants will need to be rescreened.

Table 10: Visit 2 (Enrollment/Baseline)

Component	Procedure/Analysis
**Administrative**	• Confirm informed consent<br>• Confirm eligibility<br>• Confirm/update locator information<br>• Confirm and complete randomization†<br>• Provide available test results<br>• Provide reimbursement for study visit
**Clinical**	• Review/update medical history<br>• Review/update menstrual history of female participants<br>• Record concomitant medications<br>• Perform physical exam, including digital rectal exam<br>• Provide counseling and condoms<br>o Contraception<br>o Condom use, HIV/STI risk reduction<br>o HIV pre-and post-test
**Behavioral Questionnaire**	• Administer baseline behavioral questionnaire
**Blood Specimens (~45 mL)**	• Collect blood specimens for<br>o CBC with differential and platelets<br>o BUN, creatinine, ALT, AST<br>o HIV serostatus (confirmatory tests as needed)<br>o Plasma archive<br>o PK (plasma and PBMC)
**Urine Specimens**	• Collect urine sample for<br>o Dipstick UA for protein, glucose, nitrites, and leukocyte esterase<br>o GC/CT (NAAT)<br>o Qualitative hCG for females of childbearing potential
**Vaginal Specimens**♀	• Collect vaginal sponges for PK (vaginal fluid)
**Rectal Specimens**	• Collect rectal swabs for<br>o GC/CT (NAAT)<br>• Collect rectal sponges for<br>o Cytokine profile by Luminex®<br>o PK (rectal fluid)<br>• Administer Normosol enema<br>• Collect rectal biopsies (~21) via flexible sigmoidoscopy procedure for:<br>o Histology<br>o Mononuclear cell phenotype<br>o Cytokine mRNA profile<br>o Colorectal explants<br>o PK (whole tissue and MMC)

† Randomization is completed after the V2 assessments and sample collections have been completed and lab results reviewed (BUN, CBC, creatinine, ALT, AST and STI screen)
♀ For female participants

7.3 Visits 3, 6, 9: First Dose of Study Product

Visit 3: First dose of 1st formulation in double-blinded, randomized sequence
Visit 6: First dose of 2nd formulation in double-blinded, randomized sequence
Visit 9: First dose of 3rd formulation in double-blinded, randomized sequence

Participants will have 21 days +/- 7 days washout/rest period between Visits 5 and 6, and between Visits 8 and 9.

Table 11: Visits 3, 6, 9

Component	Procedure/Analysis
**Administrative**	• Confirm/update locator information<br>• Provide available test results<br>• Provide reimbursement for study visit
**Pre-dose Procedures**	
**Clinical**	• Review/update medical history<br>• Review/update menstrual history of female participants<br>• Record any AEs and concomitant medications<br>• Perform physical exam, including digital rectal exam<br>• Provide counseling and condoms<br>o Contraception<br>o Condom use, HIV/STI risk reduction<br>o HIV pre-and post-test
**Blood Specimens (~5 mL)**	• Collect blood specimens for<br>o HIV serostatus (confirmatory tests as needed)
**Urine Specimens**	• Collect urine sample for<br>o Dipstick UA for protein, glucose, nitrites, and leukocyte esterase<br>o GC/CT (NAAT)<br>o Qualitative hCG for females of childbearing potential
**Rectal Specimens**	• Collect rectal swabs for<br>o Microflora<br>o GC/CT (NAAT)<br>• Collect rectal sponges for cytokine profile by Luminex®
**Study Product Dose**	• Single dose of study gel administered rectally<br>• Five doses of study are dispensed to be administered at home (plus 1 extra for emergency purposes) together with Wisebag<br>• Remind subjects that dosing should be done each morning and recorded in the *Product Use Log*<br>• Arrange for daily product administration verification call and explain that prior to the daily call, study staff will check the wireless Wisebag system (that tracks Wisebag use online) to verify adherence

7.4 Take-Home Periods of 5 Doses of Study Product

After the first dose of the randomized product is administered in clinic at Visits 3, 6, and 9, five (5) applicators will be dispensed to study participants to be administered at home (plus 1 extra applicator). Instructions will be reviewed and proper procedures for rectal administration will be explained. Participants will be instructed to rectally insert the product at approximately the same time every day for five days and note the time of application on the *Product Use Log*. This log can also be used to note various adverse events participants may experience.

In addition, a daily product administration verification call will take place during the take-home stages of the trial. Specific time of the call will be arraged between participants and study staff upon dispensation of the take-home applicators. Prior to each daily call, study staff will check the wireless Wisebag system (that tracks Wisebag use) and discuss any adherence issues during the calls.

7.5 Visit 4, 7, 10: Seventh Dose of Study Product

Visit 4: Seventh dose of 1st formulation in double-blinded, randomized sequence
Visit 7: Seventh dose of 2nd formulation in double-blinded, randomized sequence
Visit 10: Seventh dose of 3rd formulation in double-blinded, randomized sequence

Table 12: Visits 4, 7, 10

Component		Procedure/Analysis
**Administrative**		• Confirm/update locator information<br>• Provide available test results<br>• Provide reimbursement for study visit
**Pre-dose Procedures**		
**Clinical**		• Review/update medical history<br>• Review/update menstrual history of female participants<br>• Record any AEs and concomitant medications<br>• Perform physical exam, including digital rectal exam<br>• Provide counseling and condoms<br>o Contraception<br>o Condom use, HIV/STI risk reduction
**Urine Specimens**		• Collect urine sample for<br>o Qualitative hCG for females of childbearing potential
**Rectal Specimens**		• Collect rectal sponges for cytokine profile by Luminex®<br>• Administer Normosol enema
**Study Product Dose**		• Single dose of study gel administered rectally
**Post-dose Procedures**		
**30 min** (+/- 15 min)	**Blood Specimen (~25 mL)**	• Collect blood specimens for PK (plasma and PBMC)
	**Vaginal♀ Specimen**	• Self-collected vaginal sponges for PK (vaginal fluid)
	**Rectal Specimens**	• Collect rectal sponges for<br>o PK (rectal fluid)<br>• Collect rectal biopsies (~21) via flexible sigmoidoscopy procedure for:<br>o Histology<br>o Mononuclear cell phenotype<br>o Cytokine mRNA profile<br>o Colorectal explants<br>o PK (whole tissue and MMC)
**2 hr** (+/- 30 min)	**Blood (~25 mL), Rectal, and Vaginal♀ Specimens**	• Collect blood specimens for PK (plasma and PBMC)<br>• Self-collected vaginal sponges for PK (vaginal fluid)<br>• Collect rectal sponges for<br>o PK (rectal fluid)
**~4 hr** (+/- 30 min)	**Blood (~25 mL), Rectal, and Vaginal♀ Specimens**	• Collect blood specimens for PK (plasma and PBMC)<br>• Self-collected vaginal sponges for PK (vaginal fluid)<br>• Collect rectal sponges for<br>o PK (rectal fluid)

♀ For female participants

7.6 Visit 5, 8, 11: Specimen Sampling 24-hr Post Seventh Dose

The 24-hr post-dose sampling visit must occur within 18 to 30 hours post seventh dose.

Table 13: Visits 5, 8, 11

Component	Procedure/Analysis
**Administrative**	• Confirm/update locator information<br>• Provide available test results<br>• Provide reimbursement for study visit
**Clinical**	• Record any AEs and concomitant medications<br>• Provide condoms
**Acceptability Questionnaires**	• Administer:<br>o Web-based Gel-Use Questionnaire<br>o Web-based Application Process Questionnaire **(at Visit 11 only)**
**Acceptability phone interview (at Visit 11 only)**	• Brief qualitative interview to clarify web based responses generated during the study on acceptability questionairres
**Blood Specimens (~25 mL; ~30 mL at Visit 11)**	• Collect blood specimens for PK (plasma and PBMC)<br>• **At Visit 11 only:** Collect additional blood specimen for Hepatitis B Surface Antigen (HBsAg) test
**Vaginal Specimens**♀	• Self-collected vaginal sponges for PK (vaginal fluid)
**Rectal Specimens**	• Collect rectal swabs for<br>o Microflora<br>• Collect rectal sponges for<br>o Cytokine profile by Luminex®<br>o PK (rectal fluid)

♀ For female participants

Participants will have 21 days +/- 7 days washout/rest period between Visits 5 and 6, and between Visits 8 and 9.

7.7 Exit: Termination Phone Call/Visit

The Termination Phone Call/Visit should take place within 7-14 days of Visit 11. Study staff will conduct a phone call with participants to inquire about AEs and to review any available test results. If indicated, a visit may be scheduled instead or in addition to the termination phone call. If all clinical laboratory test results are not available at the time of the follow-up call, another call will be made once the results are available.

Table 14: Exit – Termination Phone Call/ Visit

Component	Procedure/Analysis
**Clinical**	• Provide available test results<br>• Review/update medical history<br>• Record any AEs and concomitant medications<br>• Schedule a clinic visit if indicated

7.8 Interim Contacts and Visits

Interim visits may be performed at any time during the study. All interim contacts and visits will be documented in the source documentation and on applicable case report forms.

Some Interim Visits may occur for administrative reasons. For example, the participant may have questions for study staff. Other interim contacts and visits may occur in response to AEs experienced by study participants. When interim contacts or visits are completed in response to participant reports of AEs, study staff will assess the reported event clinically and provide or refer the participant to appropriate medical care.

Table 15: Interim Contact and Visits

Component	Procedure/Analysis
**Administrative**	• Review/update demographic information<br>• Review/update locator information<br>• Provide test results, if applicable
**Clinical**	• Review/update medical history<br>• Review/update menstrual history for female participants<br>• Record any AEs and concomitant medications<br>• Perform symptom directed physical exam, if clinically indicated<br>• Perform digital rectal exam and standard anoscopy, if clinically indicated<br>• Reinforce counseling<br>o Contraception<br>o Condom use, HIV/STI risk reduction<br>o HIV pre-and post-test counseling
**Specimens**	• Collect appropriate specimens and perform testing as clinically indicated

7.9 Participants Who Become Infected with HIV

If a participant self-reports seroconversion while on study, study staff will direct the participant to the appropriate HIV care. Study staff will also request documentation of lab results or, failing availability of test results, will perform an HIV-1 test. Protocol-specified procedures will continue until the current stage is completed except:

- Product use
- HIV serology
- STI screening
- Counseling for HIV/STI risk reduction. Counseling will be modified to address primary and secondary HIV/STI prevention for infected individuals.

7.10 Participants Who Become Pregnant

Study staff will capture pregnancies on study CRFs. Protocol-specified procedures will continue until the current stage is completed except:

- Product use
- Contraceptive counseling
- Qualitative hCG
- Flexible sigmoidoscopy and biopsies
- Collection of anorectal swabs and sponges
- Collection of vaginal swabs and sponges

7.11 Participants Who Withdraw or Are Withdrawn from the Study

If the participant withdraws or is withdrawn from the study after receiving study product an Early Termination Visit will be conducted, if possible.

Table 16: Early Termination Visit

Component	Procedure/Analysis
**Administrative**	• Review/update demographic information<br>• Review/update locator information<br>• Provide test results, if applicable
**Clinical**	• Review/update medical history<br>• Record any AEs and concomitant medications<br>• Perform symptom directed physical exam<br>• Perform digital rectal exam and standard anoscopy<br>• Reinforce counseling<br>o Contraception<br>o Condom use, HIV/STI risk reduction<br>o HIV pre-and post-test counseling
**Gel use query**	• Participants will be asked which gel they thought they were receiving (VF, RF, or RGFV)
**Specimens^**	• Collect appropriate specimens and perform testing as clinically indicated<br>• If appropriate and the participant is willing, the following research specimens will also be collected
**Blood Specimens^**	• Collect blood specimens for PK (plasma and PBMC)<br>• Collect blood specimens for<br>o CBC with differential and platelets<br>o Blood urea nitrogen (BUN), creatinine, alanine transaminase (ALT), aspartate aminotransferase (AST)<br>o Hepatitis B Surface Antigen
**Vaginal Specimens♀^**	• Self-collected vaginal sponges for PK (vaginal fluid)
**Rectal Specimens^**	• Collect rectal swabs for<br>o Microflora<br>• Collect rectal sponges for<br>o Cytokine profile by Luminex®<br>o PK (rectal fluid)<br>• Administer Normosol enema<br>• Collect rectal biopsies (~21) for<br>o Histology<br>o Mononuclear cell phenotype<br>o Cytokine mRNA profile<br>o Colorectal explants<br>o PK (whole tissue and MMC)

♀ For female participants
^ Only to be performed if appropriate and the participant is willing to have specimens collected

7.12 Final Contact

After the Exit phone call or Early Termination Visit, a final contact may be required to provide laboratory test results and post-test counseling. In addition, for participants who become pregnant during study participation, an additional contact may be required to ascertain the participant's pregnancy outcome. All final contacts will be documented in participant study records.

7.13 Clinical Evaluations and Procedures

The following history and physical exam components will be conducted at select visits.

Medical History

- Each participant will be asked about any symptoms or AEs experienced since their previous visit
- When appropriate a complete Review of Systems will be completed

Physical Exam

- Height (may be omitted after the Screening Visit)
- Weight (may be omitted after the Screening Visit)
- Vital signs
    - Temperature
    - Pulse
    - Blood pressure
- General appearance
- Abdomen
- Other components as indicated by participant symptoms

Rectal Exam and Rectal Specimen Collection

The participant will be positioned in the left lateral decubitus position for the following procedures:

- Visual and digital rectal exam: The examiner will conduct a visual examination of the anus and surrounding area and note any abnormality. The examiner will then insert a lubricated gloved finger into the anal canal and sweep around the internal anal circumference.
- Rectal swab and sponge collection:
    - A lubricated plastic anoscope will be gently and fully inserted (until the lateral 'wings' touch the anal margin) and the obturator removed.
    - Swabs for GC/CT and microflora will be sequentially inserted through the anoscope and placed in contact with the rectal wall, turned through 360 degrees and removed.
    - Sponges will be inserted through the anoscope after swab collection, placed in contact with rectum, and allowed to remain there for 5 minutes.
    - After the sponges are removed the anoscope will be slowly removed.
- Enema: A 125 mL Normosol enema will be inserted through the anus and the contents gently squeezed into the rectum. The participant will hold the fluid in the rectum for 5 minutes then expel it.
- Flexible sigmoidoscopy and biopsy: A flexible sigmoidoscope will be inserted to approximately 10-20 cm, where approximately 17-21 biopsies will be taken using large-cup biopsy forceps.

Vaginal Specimen Collection

Female participants will be instructed in the self-collection of vaginal swabs and sponges.

- Swabs: The participant will gently insert 2 cotton swabs into her vagina, turn the swabs 360 degrees and remove them. One swab will be used to test the pH on site with a pH indicator strip ranging from 3.6 to 6.1. The second swab will be rolled onto a clean microscope slide, air-dried, labeled, and placed in a protective slide holder.
- Sponges: The participant will insert the sponge(s) into her vagina and allow them to remain in contact with the vaginal wall for 1 minute. After 1 minute, she will remove the sponges and place them in the collection media.

7.14 Laboratory Evaluations

7.14.1 Clinical Laboratory Testing

The local clinical laboratory or designee will run the following, as indicated:

- Blood specimens:
  - CBC with platelets and differential
  - PT/INR
  - BUN, creatinine, AST, ALT
  - HBsAb
  - HBsAg
  - HSV serology (HSV 1 and 2)
  - Syphilis testing by RPR with confirmatory testing as needed
  - HIV-1 serology, with confirmatory testing as needed

- Urine specimens:
  - Dipstick UA
  - Urine GC/CT (NAAT)
  - Qualitative hCG

7.14.2 Research Laboratory Testing

Table 17: Laboratory Assay Responsibilities

Assay		JHU Clinical Pharmacology Analytical Laboratory	MWRI	UCLA
**HIV**			Local, as needed	Local, as needed
**BV**	**pH**		Local bedside	Local bedside
	**Gram Stain**		Overnight	
**Rectal GC/CT (NAAT)**			Overnight	
**PK**		Batch		
**Rectal Microflora**			Overnight	
**Histology**			Batch	
**MMC (Flow)**			Processed and analyzed	Processed and shipped to MWRI for analysis
**Cytokine Profile**	**Luminex®**			Batch
	**mRNA**		Batch	

**Explants**	**Set-up**		Local	Local
	**p24**			Batch

Local= Samples collected and run at local site
Overnight= Samples collected at local site and will be shipped overnight to the laboratory indicated
Batch= Samples collected at local site and will be batch shipped to the laboratory indicated
See SSPs for instructions on processing and shipping samples.
HIV PCR using plasma archive samples will only be run if needed.

7.15 Specimen Collection, Handling, and Processing

Study sites will adhere to the standards of good clinical laboratory practice, DAIDS Laboratory Requirements and site standard operating procedures for proper collection, labeling, transport, and storage of specimens at the local laboratories.

(http://www.niaid.nih.gov/LabsAndResources/resources/DAIDSClinRsrch/Documents/gclp.pdf)

Specimen collection, testing, and storage at the site laboratories will be documented per standard site practice. In cases where laboratory results are not available due to administrative or laboratory error, sites are permitted to re-draw specimens that are intended for use in the screening as well as ongoing safety assessments process.

Specimens will be handled in accordance with Requirements for DAIDS Sponsored and/or Funded Laboratories in Clinical Trials.

(https://phacs.nichdclinicalstudies.org/publicDocs/DAIDS_SourceDocPolicy.pdf)

7.16 Storage of Specimens for Future Use

Study staff will store all specimens collected in this study on site at least through the end of the study. Specimens will not be labeled with any personal identifiers. Storage of all study samples will follow local standard operating procedure to ensure the anonymity and confidentiality of the trial research participants. Specimens remaining at the end of the study will be transferred to a designated bio-repository with appropriate participants' permission and after all protocol-required and quality assurance testing has been completed. If such permission is not obtained, those participant's samples will be destroyed.

7.17 Biohazard Containment

As the transmission of blood-borne pathogens can occur through contact with contaminated needles, blood, and blood products, appropriate blood and secretion precautions will be employed by all personnel in the drawing of blood and shipping and handling of all specimens for this study, as currently recommended by the United States Centers for Disease Control and Prevention. All infectious specimens will be transported in accordance with US regulations, 42 Code of Federal Regulations (CFR) 72.

7.18 Behavioral measures

Baseline behavioral questionnaire

This will be a Web-based self interview that the participants will complete at the Visit 2 at a computer terminal located in the research offices. In addition to demographics, this questionnaire will assess participants' history of anal sex in the last three months, habits of condom- and lubricant-use and experience with use of enema's/rectal douches. It will also include questions about history of alcohol use and other psycho-active substances.

Gel-use questionnaires

These web-based self-interviews will be completed by the participants at Visits 5, 8 and 11. These questionnaires will include structured and semi-structured questions about experiences the participant had using the three different gel formulations rectally. This evaluation will include an assessment of several specific characteristics, evaluating if participants liked or disliked these characteristics and if they could pose a barrier in future sustained use. The questionnaire will furthermore ask for side-effects participants experienced while using the gel, and how much participants were bothered by them. In addition, during each gel-use questionnaire, participants will be asked what study product they thought they were receiving during each completed study stage.

Application process questionnaire

This web-based self-interview will be completed by the participants at Visit 11, after administration of the last gel characteristics questionnaire. This questionnaire will evaluate the process of applying the gel, the participants' opinion on the applicator's design and will evaluate the feasibility of the daily use-regimen. This will be done by asking participants about their likes and dislikes, and their opinions on possible future use-barriers. In this questionnaire participants will furthermore be asked to compare the three different gel-formulations used over the course of the trial.

Acceptability telephone interview

At the end of the study a trial staff member will conduct an acceptability telephone interview with the study participants. The answers participants provided on the questionnaires will guide this telephone interview by inquiring about product characteristics participants ranked as 1 or 2 on the 5-level Likert-scales, indicating that participants disliked them or foresaw them as barriers in future sustained use. The interview will take about 20-30 minutes and will be audiotaped. All tapes of the interviews will be kept in a locked cabinet and after the interviews have been transcribed and checked for accuracy, the tapes will be destroyed.

Wisebag

Wisebag™ (Wisepill Technologies Adherence Management Solutions, South Africa) is a portable, lunch-bag size container, and has a self-contained battery-operated electronic device that will send a real-time signal to a central management system through a wireless system. Through this latter modality, it is possible to monitor adherence on an ongoing, 'real time' basis. This study will include the use of a Wisebag for all home administrations, and the study staff will check the Wisebag data via the online tracking system prior to the daily product administration verification calls taking place during the home administration period. Wisebag will provide an alternative measure of adherence to the study products next to self-reported adherence, and it will provide the opportunity to evaluate the usability of Wisebag-technology in future rectal microbicide trials [69,70].

8 ASSESSMENT OF SAFETY

8.1 Safety Monitoring

The study site investigators are responsible for continuous close safety monitoring of all study participants and for promptly alerting the CHARM Program Chair (who is the IND Sponsor) and the protocol team if unexpected concerns arise. A sub-group of the protocol team, including the CHARM Program Chair, Protocol Chair, CONRAD Medical Officer (MO), DAIDS MO, and the study site investigators serves as the Protocol Safety Review Team (PSRT).

The CHARM Regulatory Compliance and Informatics Core (Core B) will review monthly safety and enrollment data reports prepared by the site team. Monthly status reports will be submitted to the DAIDS MO, CONRAD MO, CHARM Protocol Chair, and CHARM Program Chair (who is the IND Sponsor) at the University of Pittsburgh for review. These reports will include all adverse events reported for the study, determined to be related or unrelated to the study products. The study team will meet, as needed, throughout the period of study implementation to review the safety data (blinded to treatment assignment), discuss product use management, and address any potential safety concerns

8.2 Clinical Data Safety Review

A multi-tiered safety review process will be followed for the duration of this study. The study site investigators are the first layer and are responsible for the initial evaluation and reporting of safety information at the participant level, and for promptly alerting the PSRT if unexpected concerns arise. In the event of two or more study participants' experiencing an AE ≥ Grade 3, study site investigator, CHARM Protocol Chair, and CHARM Program Chair will review and discuss the appropriate study management with the DAIDS and CONRAD MOs.

During the trial, the PSRT will review monthly status reports described above and conduct calls to clarify the data as appropriate. The content, format and frequency of

the safety reports will be agreed upon in advance of study implementation. If necessary, external experts representing expertise in the fields of microbicides, biostatistics, HIV transmission, and medical ethics may be invited to review the events.

If at any time, a decision is made to discontinue study gel in all participants, the CHARM Program Chair and IND Sponsor Dr. Ian McGowan/University of Pittsburgh, after consultation with the CONRAD MO, the DAIDS MO, and the protocol team will inform the US Food and Drug Administration (FDA) and the study site PIs. The site PIs will notify the responsible IRBs expeditiously.

8.3 Adverse Event Definitions and Reporting Requirements

8.3.1 Adverse Events

An adverse event is defined as any untoward medical occurrence in a clinical research participant administered an investigational product and which does not necessarily have a causal relationship with the investigational product. As such, an AE can be an unfavorable or unintended sign (including an abnormal laboratory finding, for example), symptom or disease temporally associated with the use of an investigational product, whether or not considered related to the product. This definition is applied to all the study groups, and is applied to all groups beginning from the time of randomization. The term “investigational product” for this study refers to all study products listed in Section 6 plus the gel applicator.

Study participants will be instructed to contact the study site staff to report any AEs they may experience at any time between enrollment and completion of their participation. In the case of a life-threatening event, they will be instructed to seek immediate emergency care. Where feasible and medically appropriate, participants will be encouraged to seek evaluation where the study clinician is based, and to request that the clinician be contacted upon their arrival. Sites will obtain written permission from the participant to obtain and use records from non-study medical providers to complete any missing data element on a CRF related to an adverse event. All participants reporting an untoward medical occurrence will be followed clinically, until the occurrence resolves (returns to baseline) or stabilizes.

The study site investigators will determine AE resolution or stabilization in their best clinical judgment, but may seek medical consultation regarding follow-up or additional evaluations of an AE from the DAIDS MO and/or PSRT. Study site staff will document in source documents and case report forms all AEs reported by or observed in enrolled study participants regardless of severity and presumed relationship to study product. The DAIDS AE Grading Table Version 1.0, December 2004 (Clarification dated August 2009), Addenda 1 and 3 (Female Genital and Rectal Grading Tables for Use in Microbicide Studies) will be the primary tools for grading adverse events for this protocol. Adverse events not included in that table will be graded by the DAIDS AE Grading Table, Version 1.0 December 2004 (Clarification dated August 2009). In cases where an AE is covered in multiple tables, Addendum

3 (Rectal Grading Table for Use in Microbicide Studies) will be the grading scale utilized. Please note that the grading scale for proteinuria should also be used for grading glycosuria.

8.3.2 Serious Adverse Events

Serious adverse events (SAEs) will be defined by the Manual for Expedited Reporting of Adverse Events to DAIDS (Version 2.0, dated January 2010) as AEs occurring at any dose that:

- Results in death
- Is life-threatening
- Results in persistent or significant disability/incapacity
- Is a congenital anomaly/birth defect
- Requires inpatient hospitalization or prolongation of existing hospitalization

  Note: Per ICH SAE definition, hospitalization itself is not an adverse event, but is an outcome of the event. Thus, hospitalization in the absence of an adverse event is not regarded as an AE, and is not subject to expedited reporting. The following are examples of hospitalization that are not considered to be AEs:

  - o Protocol-specified admission (e.g. for procedure required by study protocol)
  - o Admission for treatment of target disease of the study, or for pre-existing condition (unless it is a worsening or increase in frequency of hospital admissions as judged by the clinical investigator)
  - o Diagnostic admission (e.g. for a work-up of an existing condition such as persistent pretreatment lab abnormality)
  - o Administrative admission (e.g. for annual physical)
  - o Social admission (e.g. placement for lack of place to sleep)
  - o Elective admission (e.g. for elective surgery)

Important medical events that may not result in death, be life-threatening, or require hospitalization may be considered a serious adverse drug experience when, based upon appropriate medical judgment, they may jeopardize the patient or subject and may require medical or surgical intervention to prevent one of the outcomes listed above.

8.3.3 Adverse Event Relationship to Study Product

The relationship of all AEs to study product will be assessed per the *Manual for Expedited Reporting of Adverse Events to DAIDS (Version 2.0, dated January 2010)*, the Investigator’s Brochures, and clinical judgment. Per the *Manual for Expedited Reporting of Adverse Events to DAIDS (Version 2.0, dated January 2010*), the relationship categories that will be used for this study are:

- ***Related***: There is a reasonable possibility that the AE may be related to the

study agent(s)

- ***Not related***: There is not a reasonable possibility that the AE is related to the study agent(s)

8.4 Expedited Adverse Event (EAE) Reporting Requirements

Expedited Adverse Event Reporting

Requirements, definitions and methods for expedited reporting of Adverse Events (AEs) are outlined in Version 2.0 of the DAIDS EAE Manual, which is available on the RSC website at http://rsc.tech-res.com/safetyandpharmacovigilance/.

The DAIDS Adverse Experience Reporting System (DAERS), an internet-based reporting system, must be used for expedited AE reporting to DAIDS. In the event of system outages or technical difficulties, expedited AEs may be submitted via the DAIDS EAE Form. For questions about DAERS, please contact DAIDS-ES at DAIDS-ESSupport@niaid.gov. Site queries may also be sent from within the DAERS application itself. Where DAERS has not been implemented, sites will submit expedited AEs by documenting the information on the current DAIDS EAE Form. This form is available on the RSC website: http://rsc.tech-res.com/safetyandpharmacovigilance/. For questions about EAE reporting, please contact the RSC (DAIDSRSCSafetyOffice@tech-res.com).

EAE reporting procedures specific to this protocol: Once the sites have submitted EAEs via DAERS (as above), the RSC Safety Office will prepare and forward a draft safety report to the CHARM Program Chair (who is the IND Sponsor), CONRAD MO, and DAIDS MO. The CHARM Program Chair will review the draft safety report with the appropriate institutional representatives and safety physicians at the University of Pittsburgh and MWRI, as well as CONRAD and DAIDS MOs.

Study sites will be contacted if any further information or clarification is needed. The RSC Office will then prepare the final safety report and submit it to the CHARM Program Chair/University of Pittsburgh for submission to the FDA. The CHARM Program Chari (IND Sponsor) will assume responsibility for the reporting of Serious Adverse Events to the FDA and regulatory agencies outside the U.S., as appropriate and per 21 CFR 312.32 and 21 CFR 312.33. Copies of this final report will be filed with CONRAD and RSC. Additionally, the RSC Safety Office will distribute safety reports to all DAIDS sites that use products under investigation in this study.

For all EAEs submitted, sites must file an RSC update with the final or stable outcome unless the initial EAE submitted had a final or stable outcome noted already.

In case of a fatal or life-threatening suspected adverse reaction, the CHARM Program Chair (IND Sponsor) will notify the responsible review division of the FDA by telephone or facsimile transmission as soon as possible but in no event later than 7 calendar days after the CHARM Protocol Chair's receipt of the respective adverse event information and determination that it meets the respective criteria for reporting.

EAE Reporting Requirements for this Study

The Serious Adverse Event (SAE) EAE Reporting Category, as defined in Version 2.0 of the DAIDS EAE Manual, will be used for this study. The study agents for which expedited reporting is required are: rectal-specific formulation (RF) of tenofovir 1% gel, the reduced glycerin vaginal formulation (RGVF) of tenofovir 1% gel, the vaginal formulation (VF) of tenofovir 1% gel, Universal HEC Placebo Gel, and the gel applicator.

Grading Severity of Events

The Division of AIDS Table for Grading the Severity of Adult and Pediatric Adverse Events, Version 1.0, December 2004 (Clarification dated August 2009), Addenda 1 and 3 (Female Genital and Rectal Grading Tables for Use in Microbicide Studies) will be the primary tools for grading adverse events for this protocol. Adverse events not included in those tables will be graded by the *DAIDS AE Grading Table, Version 1.0 December 2004 (Clarification dated August 2009)*. In cases where an AE is covered in multiple tables, *Addendum 3 (Rectal Grading Table for Use in Microbicide Studies)* will be the grading scale utilized.

The *DAIDS AE Grading Table, Version 1.0, December 2004 (Clarification dated August 2009)* and *Addenda 1 and 3* are available on the RSC website at http://rsc.tech-res.com/safetyandpharmacovigilance/.

EAE Reporting Period

- The expedited AE reporting period for this study is defined as the entire study duration for an individual participant (from randomization until the participant's final study contact).
- After the protocol-defined AE reporting period, unless otherwise noted, only suspected unexpected serious adverse reactions (SUSARs as defined in Version 2.0 of the EAE Manual) will be reported to DAIDS if the study staff become aware of the events on a passive basis (from publicly available information).

8.5 Reporting of Adverse Reactions to the Responsible IRBs

The study site investigators will report adverse reactions to the responsible IRB for that study site in accordance with respective IRB policies and procedures.

Follow-up information to a reported adverse event will be submitted to the IRB as soon as the relevant information is available. If the results of the study site investigator's follow-up investigation show that an adverse event that was initially determined to not require reporting to the IRB does, in fact, meet the requirements for reporting, the study site investigator will report it as soon as possible in accordance with respective IRB policies and procedures.

8.6 Pregnancy and Pregnancy Outcomes

Pregnant participants are excluded from this study. Routine urine testing is performed at study visits 1, 2, 3, 4, 6, 7, 9, and 10 for female participants of childbearing potential. If participants become pregnant at any time during the course of the study, study agents are discontinued, but participants will remain in the study and will continue with the assessments outlined in Section 7.11, if deemed reasonable by both the site investigator and the participant.

Pregnancy-related data will be collected using CRFs for all pregnancies detected during the study. Pregnancy outcomes will not be expeditiously reported to CHARM Program Chair (IND Sponsor), CONRAD, and the DAIDS MO, unless there is an associated adverse event in the pregnant participant that meets expedited reporting criteria or the pregnancy results in a congenital anomaly meeting the Manual for Expedited Reported of Adverse Events to DAIDS (Version 2.0, January 2010) guidelines for expedited reporting. Fetal losses without congenital anomalies or maternal complications that require expedited reporting will not be expeditiously reported, but data will be captured via the pregnancy CRFs.

After the participant's final study contact, pregnancy outcomes that meet criteria for EAE reporting as described above (e.g., maternal complications, congenital anomalies) occurring among participants known to be pregnant at the last study visit will continue to be expeditiously reported. CHARM Regulatory and Informatics Core will prepare and provide a quarterly report of all pregnancies and their outcomes occurring during the course of the study to the CHARM Program Chair and IND Sponsor Dr. Ian McGowan/University of Pittsburgh, CONRAD, and DAIDS. The CHARM Regulatory and Informatics Core will also prepare an annual summary report of all AEs for the annual IND reports (submitted by the CHARM Program Chair and IND Sponsor Dr. Ian McGowan/University of Pittsburgh).

8.7 Social Harms Reporting

Although study sites make every effort to protect participant privacy and confidentiality, it is possible that participants' involvement in the study could become known to others, and that social harms may result (i.e., because participants could become known as HIV-infected or at "high risk" for HIV infection). For example, participants could be treated unfairly or discriminated against, or could have problems being accepted by their families and/or communities.

Social harms that are judged by the study site investigator to be serious or unexpected will be reported to responsible site IRB at least annually or according to their individual requirements. In the event that a participant reports social harm, every effort will be made by study staff to provide appropriate care and counseling to the participant, and/or referral to appropriate resources for the safety of the participant as needed.

8.8 Withdrawal of Subjects Due to Adverse Events

Clinical Management Section 9 below includes guidelines for withdrawal of research participants from this study.

9 CLINICAL MANAGEMENT

Guidelines for clinical management and product hold/discontinuation are outlined in this section. In general, the study site investigator has the discretion to hold study product at any time if he or she feels that continued product use would be harmful to the participant, or interfere with participant's acute medical condition (such as e.g. viral gastroenteritis) or with treatment deemed clinically necessary according to the judgment of the investigator.

9.1 Grading System

The primary grading system for this study is *Rectal Grading Table for Use in Microbicide Studies*; it is Addendum 3 to the *DAIDS AE Grading Table, Version 1.0, December 2004 (Clarification dated August 2009)* and can be found at the Regulatory Support Center (DAIDS RSC) website http://rsc.tech-res.com/safetyandpharmacovigilance/.

9.2 Dose Modification

No dose modifications will be undertaken in this study.

9.3 Discontinuation of Study Product(s) in the Presence of Toxicity

Each participant will receive seven doses of RF and RGVF, and a single dose of VF which will be preceded by six doses of the Universal HEC Placebo Gel. Unless otherwise specified adverse events will be managed as follows:

Grade 1 or 2
In general, participants who develop a Grade 1 or 2 AE regardless of relatedness to study product that is not specifically addressed further in the protocol, may continue use of study products per protocol.

Grade 3
Participants who develop a Grade 3 AE or toxicity that is not specifically addressed further in the protocol and is judged to be related to study product should have that study product permanently discontinued.

Grade 4
Participants who develop a Grade 4 AE or toxicity that is not specifically addressed further in the protocol, regardless of relationship to study product, should have the current study product permanently discontinued.

9.4 General Criteria for Discontinuation of Study Product(s)

Study participants will be permanently discontinued from product use by the study site investigator or a designated sub-investigator in the event of the following:

- Pregnancy
- HIV seroconversion
- Clinical reasons determined by the investigator

9.5 Management of Specific Adverse Events

9.5.1 Hemorrhage Following Rectal Mucosal Biopsy

If bleeding continues after the flexible sigmoidoscopy procedure that is uncontrolled and results in the passage of blood clots per rectum, the participant will be referred for assessment in the emergency department of the nearest hospital.

9.5.2 Infection Following Rectal Mucosal Biopsy

The rate of local or systemic infection following colorectal biopsy is very low. Any participant presenting with local or systemic features compatible with infection (fever, localized anorectal pain, anal discharge) will be referred to the emergency department of the nearest hospital.

9.5.3 Perforation of Rectum Following Rectal Mucosal Biopsy

Intestinal perforation associated with flexible sigmoidoscopy and biopsy is estimates at 1: 8,000 [49]. Any participant presenting with local or systemic clinical features suggestive of this condition (abdominal pain, swelling, fever) will be referred to the emergency department of the nearest hospital. The research teams at UCLA and MWRI have extensive experience conducting multiple endoscopic procedures with roughly the same number of biopsies within 24 hours (e.g., RMP-02/MTN-006) with no procedure-related AEs.

9.6 Criteria for Early Termination of Study Participation

Participants may voluntarily withdraw from the study for any reason at any time. The site investigator also may withdraw participants from the study to protect their safety and/or if they are unwilling or unable to comply with required study procedures. Participants also may be withdrawn if the study sponsor, government or regulatory authorities (including the Office of Human Research Protections), or site IRBs terminate the study prior to its planned end date. Every reasonable effort is made to complete a final evaluation of participants who withdraw or are withdrawn from the study prior to completing follow-up. Study staff members will record the reason(s) for all withdrawals in participants' study records.

10 STATISTICAL CONSIDERATIONS

10.1 Overview and Summary of Design

This will be a double-blinded, randomized, safety, pharmacokinetic, and *ex vivo* efficacy study of three rectally applied tenofovir-based microbicide formulations: a rectal formulation (RF), a vaginal formulation (VF), and a reduced glycerin vaginal formulation (RGVF). Each participant will experience seven rectal exposures to RF and seven rectal exposures to RGVF, but only one exposure to the vaginal formulation (VF), which will be coupled with six preceding exposures to the Universal HEC Placebo Gel to balance out the study stages.

After completing a screening evaluation (Visit 1), participants will return to clinic for a baseline evaluation (Visit 2), including flexible sigmoidoscopy and sample collection, and then be randomized to study product sequence. With 21 days +/- 7 days washout/rest period between each stage, participants will return to the clinic for three study stages, called *Stage 1-Administration of First Product*, *Stage 2 – Administration of Second Product*, and *Stage 3 – Administration of Third Product*. Each stage will begin with the first dose administered in clinic (Visits 3, 6, and 9) during which the participant will receive a single rectally-applied dose of one of the study products in a double-blinded, randomized sequence and will then have 5 doses of the same product dispensed for take-home use. After completing the take-home use period, participant will return to clinic for the administration of the seventh dose of the same formulation, with various specimen collections at the following time points: pre-dose, 0.5-hr post-dose, 2-hr post-dose, 4-hr post-dose, and 24-hr post-dose. The sequence of visits will be repeated for each gel formulation. A more detailed summary of specimen collection can be found in Table 4 and Appendix I.

10.2 Study Endpoints

10.2.1 Primary Endpoints

Consistent with the primary study objectives to evaluate the safety and to compare systemic and compartment pharmacokinetics of each tenofovir-based microbicide gel formulation when applied rectally, the following endpoints will be assessed:

Safety

- Grade 2 or higher clinical and laboratory adverse events as defined by the *Division of AIDS Table for Grading the Severity of Adult and Pediatric Adverse Events, Version 1.0, Dec 2004* and Addenda 1 and 3 (*Female Genital and Rectal Grading Tables for Use in Microbicide Studies*) to this table

Acceptability

- Product attributes considered likely to challenge future sustained use by participants.

Pharmacokinetics

- Tenofovir concentrations
  - Plasma
  - PBMC (intracellular)
  - Rectal fluid
  - Vaginal fluid, in female participants
  - Rectal mucosal tissue homogenates
  - Rectal MMC

- Tenofovir diphosphate concentrations
  - PBMC
  - Rectal mucosal tissue homogenates
  - Rectal MMC

10.2.2 Secondary Endpoints

Consistent with the secondary study objective to evaluate mucosal immunotoxicity of each tenofovir-based microbicide gel formulation when applied rectally, the following endpoints will be assessed:

- Rectal microflora
- Rectal cytokines (secreted and tissue mRNA)
- Rectal histology
- Rectal tissue CD4 cell phenotype/activation

10.2.3 Exploratory Endpoints

Consistent with the exploratory study objective to evaluate preliminary (*ex vivo*) efficacy of each tenofovir-based microbicide gel formulation when applied rectally, the following endpoint will be assessed:

- Changes in vitro applied HIV-1 p-24 levels in colorectal explant supernatant after each product is applied rectally

10.3 Study Hypotheses

This protocol and derived data will provide information that will address, even if not completely answer, the following study hypotheses:

- All three formulations of tenofovir 1% gel will be safe when applied rectally
- The RF of tenofovir 1% gel will be at least as safe as the VF and RGVF formulations when applied rectally

- All three formulations of tenofovir 1% gel will be equally acceptable when applied rectally
- All three formulations will have measurable drug concentrations in all compartments studied following a declining concentration gradient away from the dosing site in the rectal lumen
- The RF of tenofovir 1% gel will be equal to or less immunotoxic to the mucosa when applied rectally than the VF and RGVF formulations
- The RF of tenofovir 1% gel will be at least as effective in preventing infection by *ex vivo* HIV challenge testing as the VF and RGVF formulations

10.4 Accrual and Sample Size

There will be a total of 18 participants, men and women, enrolled at two clinical sites (UCLA, MWRI). While the intent will be to enroll equally at both sites, recruitment may take place at either site to complete the study. Participants will be HIV-1 seronegative, generally healthy and at least 18 years of age.

Based on the two prior rectal microbicide trials conducted by this group (RMP-01 and RMP-02/MTN-006), it is anticipated that approximately one fifth of enrolled participants will be women. Based on the prior studies with similar eligibility requirements, each site is expected to enroll approximately 1-2 participants per month. Therefore accrual is expected to take approximately 6 months. The target for retention will be 95% of enrolled participants over the study period. In the case of discontinuation/non-adherence, additional participants may enroll at the discretion of the protocol team, to replace participants who withdraw or are withdrawn. Therefore the total sample size may be slightly exceeding 18 at the end of the study.

10.5 Randomization and Blinding

Enrolled participants will be assigned at random to one of the three study formulation sequences. Randomization will be done in blocks of size 3 at each site. This will ensure balance between formulation groups and the sequence of administration between sites. The randomization scheme will be stratified by site and be generated by the University of Pittsburgh, Center for Research on Health Care Data Center, using computer-generated random numbers.

The randomization assignments will be delivered to the Director of Pharmacy Affairs at the MWRI who holds primary responsibility for maintaining the blind and who will generate the product labels. Because of the uncertainty of the actual number of subjects that will be enrolled at each site, randomization for 12 participants (24 total) will be created for each site.

10.6 Blinding/Unblinding

Blinding will be maintained until all data are entered into the study database, all study endpoint data and other data included in the final analysis have been cleaned and

verified, and the data are ready for final analysis. This will be explained to participants as part of the informed consent process.

There are no circumstances under which it is expected that unblinding will be necessary for provision of medical treatment or to otherwise protect the safety of study participants. As described in Section 9, in the event that a study site investigator is concerned that a participant might be put at undue risk by continuing product use, the product use may be discontinued; however knowledge of the specific sequence to which the participant was assigned should not be necessary to guide further follow-up and/or treatment.

10.7 Data Analysis

Descriptive statistics and graphics will be used to summarize the characteristics of endpoints among the three study products. For categorical variables, the numbers and the proportions will be tabulated; for continuous variables, the mean, median, standard deviation, and quartiles will be reported. Unless described elsewhere, to assess the change of the primary endpoints between study products at post-dose visits, McNemar's test (for categorical variables) or paired t-test (for continuous variables) will be used. In the event that certain assumptions are violated (i.e. sample size, distribution of variable), the nonparametric or exact version of the test will be used instead. Generalized linear models will be used to compare correlated observations between study formulations.

10.7.1 Primary Analysis on Safety

For the safety analysis, the number and the frequency of ≥ Grade 2 adverse events will be tabulated for each of the three study formulations. To determine whether AEs are occurring excessively, the proportion of subjects that experience an AE will be calculated for each study formulation. A single summary outcome of this type (yes/no) can be reasonable assumed to follow a Bernoulli distribution. Table 18 shows for selected true underlying rates between .01 and .20 the probability of zero, one or more, two or more, and three or more subjects experiencing AEs in a sample of 18.

Table 18: Probability of events for selected true rates of AEs

True rate	Zero subjects	One or more subjects	Two or more subjects	Three or more subjects
**.01**	.835	.165	.014	.001
**.02**	.695	.305	.050	.005
**.03**	.578	.422	.100	.016
**.04**	.480	.520	.161	.033
**.05**	.397	.603	.226	.058
**.06**	.328	.672	.294	.090
**.07**	.271	.729	.362	.127
**.08**	.223	.777	.428	.170
**.09**	.183	.817	.491	.217
**.10**	.150	.850	.550	.266

**.11**	.123	.877	.604	.317
**.12**	.100	.900	.654	.369
**.13**	.082	.918	.699	.421
**.14**	.066	.934	.740	.471
**.15**	.054	.946	.776	.520
**.16**	.043	.957	.808	.567
**.17**	.035	.965	.836	.612
**.18**	.028	.972	.861	.654
**.19**	.023	.977	.882	.693
**.20**	.018	.982	.901	.729

Table 19: Exact 95% confidence bounds for true rate of AEs (n=18)

Number of events	Estimated rate	Lower bound	Upper bound
**0**	.000	.000	.185
**1**	.056	.016	.273
**2**	.111	.053	.347
**3**	.167	.097	.414
**4**	.222	.143	.476
**5**	.278	.191	.535

The rate of safety events will be compared between the RF formulation and each of the VF and RGVF formulations using McNemar's test for each comparison. This will be conducted after the final dosing visits (4, 7, and 10, respectively).

Additional safety analyses will also tabulate the number and type of AEs observed overall, and by severity, site, and study product. AEs that lead to discontinuation of study participation will be tabulated separately.

10.7.2 Primary Analysis on Acceptability

The baseline behavioral data will be primarily descriptive of demographic variables (age, gender-identity, sexual identity, racial background, education, and income), anal sex in the prior three months, lubricant-use, condom-use, enema-/rectal douche-use and psycho-active substance-use. The gel and application-process data will provide descriptive statistics on participants' opinions on the gel's characteristics, application process, the applicator design and the use-regimen, as well as the degree to which participants believe these characteristics and side-effects could pose barriers in future sustained use. Consistent with the primary study objective to evaluate what product characteristics participants consider to be barriers in use, the primary endpoint is to determine product characteristics disliked or considered likely to challenge future sustained use by participants. We will calculate the proportion of participants who report product characteristics to be considered a barrier in use, operationalized as having a rating of lower than 3 on a 5-point Likert scale, in disliking or likelihood of future barrier in use. We will examine the distributions of scores on all product characteristics and determine product characteristics that pose or could pose significant barriers in current or future product use. The phone interviews will be audio-taped, transcribed, and analyzed for content. In this process, we will be able to integrate the qualitative data to gain insights about the quantitative responses.

10.7.3 Primary Analysis on Pharmacokinetics

Pharmacokinetics in 5-6 compartments of each tenofovir-based gel formulation will be evaluated after rectal administration. The primary pharmacokinetic parameter to be calculated after the seventh rectal dose of each of the three tenofovir-containing product exposures will be the area under the matrix concentration-time curve from 0 to infinity ($0 \rightarrow \infty$). This will be estimated using the log-linear trapezoidal method. Tenofovir $AUC_{0\rightarrow\infty}$ will be estimated in each subject using 2 samples obtained over a 24-hr period after a tenofovir dose in the following 3 matrices: cervicovaginal fluid, rectal fluid, and rectal tissue. Tenofovir $AUC_{0\rightarrow\infty}$ will be estimated in each subject using 4 samples obtained over a 24-hr period after a tenofovir dose in the blood plasma. Tenofovir-diphosphate $AUC_{0\rightarrow\infty}$ will be estimated in each subject using 2 samples obtained over a 24-hr period after a tenofovir dose in rectal tissue, and mononuclear cells isolated from rectal tissue. Tenofovir-diphosphate $AUC_{0\rightarrow\infty}$ will be estimated in each subject in the PBMCs, using 4 samples obtained over a 24-hr period after a tenofovir dose.

To perform an extracellular and intracellular concentration comparison between blood plasma and vaginal fluid, rectal fluid, and rectal tissue, a composite approach will be used: a composite concentration-time profile over 24 hours will be generated for all matrices. This time profile will be used to calculate a composite $AUC_{0\rightarrow\infty}$, in addition to a CL/F, and a $t_{1/2}$. To compare tissue pharmacokinetics to fluid biological matrices, an estimated tissue density of 1.05 g/mL will be used to convert ng/gm to ng/mL.

Intra-individual comparisons for intracellular (PBMCs, rectal tissue, isolated rectal mononuclear cells) and extracellular (plasma, rectal fluid, vaginal fluid, rectal tissue) tenofovir $AUC_{0\rightarrow\infty}$ will be performed to determine differences in exposure between each tenofovir-based gel formulation.

Inter-individual comparisons will be performed on composite AUCs within each rectal dosing phase to determine the relative extracellular and intracellular penetration of tenofovir in systemic and peripheral compartments.

10.7.4 Secondary Analysis on Mucosal Immunotoxicity

The association of four sets of mucosal parameters with study products will be examined. Among them, histopathology is a categorical measure, whereas the mucosal mononuclear cell phenotype, cytokine mRNA, secreted cytokines are continuous measures. All four parameters are measured at baseline (Visit 2) and then either immediately pre-dosing (secreted rectal cytokines at Visit 3, Visit 6, and Visit 9) with 1-2 assessments post each product or as above with no pre-dosing measurement (histology, cytokine mRNA, flow cytometry). For the index where samples are acquired immediately pre-dosing of each product (Visit 3, Visit 6, and Visit 9) this value will serve as baseline for statistical comparisons. The sample value

from Visit 2 for secreted rectal cytokines will only serve as non-exclusionary screening information should higher-than-expected baseline inflammation be present. Each participant will serve as his or her own control.

Because of the small number of participants planned, assessments to look for changes will be done using two-sided tests with α=0.05. Hence, any significant results will need to be followed with confirmatory studies and discussion of their clinical relevance. Assuming normally distributed differences, and a paired t-test with a sample size of 18 participants, there will be 80% power to detect an effect size (Cohen's d) of 0.66 and 99% power to detect an effect size of 1 between any two product formulations. To convert the effect size d, into measured units, the derived standard deviations for each assay change is multiplied by d. Thus, for example, based on the results from the RMP-01 UC781 trial, there was a 98% power to detect an average decrease of 6.34% in CD 4-lymphocytes, and higher power for larger differences. This approach will provide sufficient power to detect any changes that we currently believe will be scientifically important.

The associations of four sets of mucosal parameters with the use of study product will be examined as a secondary objective. All four of these parameter sets (histopathology, cell phenotype, cytokine mRNA, secreted cytokines) are collected multiple times per participant. For all four parameter panels, the main question of interest is whether the study products (RF, VF, RGVF) have systematically different mucosal damage parameters post exposure to seven doses.

Paired t-tests will be conducted within each study formulation to compare continuous mucosal outcomes measured at baseline (Visit 2 or 3, depending on endpoint) with each subsequent dosing visit. For categorical mucosal outcomes, McNemar's test will be conducted.

In order to make comparisons between study formulations, a generalized linear mixed model will be utilized with each of the six mucosal parameters as the outcome variable (either 30-min post-dose or 24-hr post dose). There will be fixed predictor variables for the baseline mucosal variable, the study formulation (RF, VF, and RGVF), stage (1, 2, or 3), and the interaction between stage and formulation. In addition, there will be a random effect for subject in order to account for the correlated observations. Testing for the interaction term will give us information about whether the 21 (+/- 7) day washout period is adequate for removing any carryover effects between study formulations. If we can safely assume the absence of carryover effects, the model will be able to give us information on the variability of study formulations with respect to mucosal variables.

- **Microflora:** Microflora measures will be graded on a 0 to 4 ordinal scale and recorded at baseline and post-exposure. Depending on the empirical distributions across the points in this scale, statistical procedures will either involve analysis of the actual pre-post differences (ordinal) or dichotomized versions of the pre and post scores (binary). In the ordinal case, one sample and multi-sample signed rank tests

will be used to examine whether (i) there is an overall change in microflora levels before/after (24 hours) each product exposure and (ii) whether the study products differ significantly from each other in pre-post change. Similarly, if dichotomization is more appropriate, then the binary baseline and post-exposure data will be analyzed using exact McNemar tests (to examine if levels change significantly pre to post).

- **Histology:** Slides will be reviewed and scored by a qualified pathologist using a qualitative scoring system (Appendix IV) used in previous studies [31]. Examination of two point (normal versus abnormal) or three point (normal, slightly abnormal, abnormal) scales will be undertaken. As above, comparisons will be used to examine whether (i) there is an overall change in histology scores before/after (24 hours) each product exposure and (ii) whether the study products differ significantly from each other in pre-post change.

- **Flow cytometry:** Many of the flow cytometry measurements from the HPTN 056 study are quite stable across time. Parameters with high intrasubject correlations (RFI measures are log-transformed) include %CD3, %CD4, %CD8, CD38RFI on CD4+, CD38RFI on CD8+, %CD38||CD4, and %CD38||CD8 which have intrasubject correlations of between 0.7 and 0.9 [31]. As above, comparisons will be used to examine whether (i) there is an overall change in cell populations before/after (24 hours) each product exposure and (ii) whether the study products differ significantly from each other in pre-post change. It is expected that clinically important differences in these parameters between treatment groups will be found to be statistically significant by these analyses.

- **Mucosal Cytokine levels:** The cytokine data, in HPTN 056 and earlier work, for RANTES, IFN-γ, and IL-10 mRNA all showed strong stability (intrasubject correlations between 0.7 or 0.8; analysis was on log-transformed scale for all three of these) [64]. Again, this suggests that any clinically meaningful differences between treatment groups are likely to result in small p-values. For secreted cytokines (collected by rectal sponge), similar stability data does not yet exist but baseline data from RMP-02/MTN-006 [44] demonstrated quantifiable levels with low inter-subject variability at baseline. As above, comparisons will be used to examine whether (i) there is an overall change in cytokine levels before/after (24 hours) each product exposure and (ii) whether the study products differ significantly from each other in pre-post change.

10.7.5 Exploratory Analysis on *Ex Vivo* Efficacy

The main question of interest for the explant studies is whether the tenofovir-based gel formulations have reduced explant infectivity post-treatment. As virus growth varies according to the day of observation, comparisons will be made once exponential virus growth is being observed, using an improved statistical method ("soft endpoint"), developed for the Microbicide Quality Assurance Program (MQAP-NICHD) [46]. Optical Density (O.D.) data from all p24 assays will be compared to a universal standard curve for O.D. values within a + 95% confidence interval of the plate standards. Differences between study products will be determined using

Repeated Measures ANOVA and ANCOVA (adjusting for baseline differences).

Changes in cumulative p24 antigen will be the primary outcome in the explant studies. Unlike the situation in the immunotoxicity portion of this study, the design here is for efficacy. Consequently, a more conservative two-sided paired t-test with α=.05 will be used. Three (1%) formulations will be used and only one site (10cm) will be studied, using one viral titer (10^4 $TCID_{50}$ HIV-1 BaL; all from the same viral production lot). The previous UC781 study (RMP-01) at 10cm showed effect sizes of 1.4 for 0.25% at 10^4 $TCID_{50}$ (corresponding to an average difference of 5240) and an effect size of .8 for 0.1% at 10^4 $TCID_{50}$ (corresponding to an average difference of 1110). However, while nearly all 36 participants' tissues were infectible at baseline with the higher 10^4 titer, only 2/3 of those same 36 participants' tissues were infectible with the 10^2 viral titer at baseline [33]. Given the 18 participants, only the high viral titer will be used in this trial. Using a two-sided paired t-test with α=.05, the study will have 70% power for an effect size of .80 and 99% power for an effect size of 1.4. Comparisons between baseline and each study product will be done using multi-level models, but given the extremely small sample sizes, this will only be done for powering further studies, as there will not be sufficient power to detect a difference in changes, except for very extreme outcomes.

10.7.6 Missing Data

All reasonable efforts will be made to obtain complete data for all participants. However, missing observations will occur due to missed visits, participants lost to follow-up, or noncompliance. Research participants who fail to complete dosing and evaluation relevant to all study formulations for any reason may be replaced at the discretion of the study site investigators after consultation with the Sponsor.

As previously noted, all available data will be used for safety purposes. All available data will also be used for descriptive purposes. Formal statistical comparisons between formulations, however, must necessarily be limited to subjects who complete use of at least 2 formulations.

11 DATA HANDLING AND RECORDKEEPING

11.1 Data Management Responsibilities

Study case report forms will be developed by the CHARM Regulatory Compliance and Informatics Core (Core B). Quality control reports and queries routinely will be generated and distributed to the study sites for verification and resolution prior to reporting data to DAIDS.

11.2 Source Documents and Access to Source Data/Documents

All study sites will maintain source documents and data in accordance with *Requirements for Source Documentation in DAIDS Funded and/or Sponsored Clinical Trials*

(http://www.niaid.nih.gov/LabsAndResources/resources/DAIDSClinRsrch/Documents/sourcedocpolicy.pdf).

Each investigator will maintain and securely store complete, accurate, and current study records throughout the study. Per US regulations, for each of the three investigational products tested, the investigators will retain all study records on site for at least two years following the date of marketing approval for the study product for the indication in which it was studied. If no marketing application is to be filed or if the application is not approved, the records must be retained until two years after the investigation is discontinued and the US FDA is notified.

Study records must be maintained on site for the entire period of study implementation. Thereafter, instructions for record storage will be provided by Sponsor. No study records may be moved to an off-site location or destroyed prior to receiving approval from both DAIDS and Sponsor.

11.3 Quality Control and Quality Assurance

Quality control and quality assurance procedures will be performed by all study sites as specified in *Requirements for Clinical Quality Management Plans at DAIDS Funded and/or Supported Clinical Research Sites*:

http://www.niaid.nih.gov/LabsAndResources/resources/DAIDSClinRsrch/Documents/qmppolicy.pdf

11.4 Study Coordination

Ian McGowan of the University of Pittsburgh holds the IND for this study. Copies of all regulatory documents submitted to this IND will be forwarded to DAIDS, for cross-referencing with other INDs for the study products.

Study implementation will follow this protocol, which may not be amended without prior written approval from the Sponsor and DAIDS Medical Officer. Study implementation will also be guided by a common study-specific procedures manual that provides further instructions and operational guidance on conducting study visits; data and forms processing; specimen collection, processing, and shipping; AE assessment, management and reporting; dispensing study products and documenting product accountability; and other study operations.

Close coordination between protocol team members is necessary to track study progress, respond to queries about proper study implementation, and address other

issues in a timely manner. Rates of accrual, retention, follow-up, and AE incidence will be monitored closely by the team.

12 CLINICAL SITE MONITORING

Non-network study monitoring will be carried out by Pharmaceutical Product Development Inc., (PPD, Wilmington, NC). Site monitoring visits will be conducted to assess overall study compliance, as required per Requirements for On-Site Monitoring of DAIDS Funded and/or Sponsored Clinical Trials, GCP, and FDA regulations 21 CFR Part 312:

http://www.niaid.nih.gov/LabsAndResources/resources/DAIDSClinRsrch/Documents/onsitemonitor_reqs.pdf

Study monitors will visit the site to complete the following:

- Assess compliance with the study protocol, Good Clinical Practices (GCP) guidelines, and applicable regulatory requirements, including US CFR Title 45 Part 46 and Title 21 Parts 50, 56, and 312
- Review informed consent forms, procedures, and documentation
- Perform source document verification to ensure the accuracy and completeness of study data
- Verify proper collection and storage of biological specimens
- Verify proper storage, dispensing, and accountability for investigational study products
- Assess implementation and documentation of internal site quality management procedures
- Assess site staff training needs

Site investigators will allow study monitors to inspect study facilities and documentation (e.g., informed consent forms, clinic and laboratory records, other source documents, case report forms), as well as observe the performance of study procedures. Investigators also will allow inspection of all study-related documentation by authorized representatives of the DAIDS, Sponsor and US regulatory authorities. A site visit log will be maintained at the study sites to document all visits. The outcomes of the monitoring visits and the subsequent reports of resolutions of any identified problems will be provided to the Sponsor of the IND application.

13 HUMAN SUBJECTS PROTECTIONS

The investigators will make efforts to minimize risks to participants. Volunteers and study staff members will take part in a thorough informed consent process. Before beginning the study, the investigators will have obtained IRB approval and the protocol will have been submitted to the appropriate regulatory agencies involved in this trial.

The investigators will permit audits by the NIH, Sponsor, Office for Human Research Protections (OHRP), the FDA, or any of their appointed agents.

13.1 Institutional Review Boards

Each participating study site is responsible for assuring that this protocol and the associated site-specific informed consent documents and study-related materials are reviewed by an IRB responsible for oversight of research conducted at the study sites. Any amendments to the protocol, informed consent documents, and other study-related materials must be approved by the responsible IRB prior to implementation.

Subsequent to the initial review and approval, the responsible IRBs must review the study at least annually. Each study site investigator will submit safety and progress reports to the IRBs at least annually and within three months after study termination or completion. These reports will include the total number of participants enrolled in the study, the number of participants who completed the study, all changes in the research activity, and all unanticipated problems involving risks to human subjects or others. Study sites will submit documentation of continuing review to the DAIDS Protocol Registration Office (PRO) in accordance with the DAIDS Protocol Registration Policy and Procedures Manual.

13.2 Protocol Registration

Prior to implementation of this protocol, and any subsequent full version amendments, each site must have the protocol and the protocol consent form(s) approved, as appropriate, by their local IRB/ethics committee (EC) and any other applicable regulatory entity (RE). Upon receiving final approval, sites will submit all required protocol registration documents to the DAIDS PRO at the Regulatory Support Center (RSC). The DAIDS PRO will review the submitted protocol registration packet to ensure that all of the required documents have been received.

Site-specific informed consent forms (ICFs) *WILL NOT* be reviewed or approved by the DAIDS PRO, and sites will receive an Initial Registration Notification when the DAIDS PRO receives a complete registration packet. Receipt of an Initial Registration Notification indicates successful completion of the protocol registration process. Sites will not receive any additional notifications from the DAIDS PRO for the initial protocol registration. A copy of the Initial Registration Notification should be retained in the site's regulatory files.

Upon receiving final IRB/EC and any other applicable approval(s) for an amendment, study sites should implement the amendment immediately. Sites are required to submit an amendment registration packet to the DAIDS PRO at the RSC. The DAIDS PRO will review the submitted protocol registration packet to ensure that all the required documents have been received. A copy of the Amendment Registration Notification should be retained in the site's regulatory files.

For additional information on the protocol registration process and specific documents required for initial and amendment registrations, refer to the current version of the *DAIDS Protocol Registration Manual*, available at http://rsc.tech-res.com/protocolregistration.

13.3 Risk-Benefit Statement

13.3.1 Risks

General

Phlebotomy may lead to discomfort, feelings of dizziness or faintness, and/or bruising, swelling and/or infection. Disclosure of STI status may cause sadness or depression in volunteers. Participation in clinical research includes the risks of loss of confidentiality and discomfort with the personal nature of questions.

Although all efforts will be made to protect participant privacy and confidentiality, it is possible that participant's involvement in the study could become known to others, and that social harms may result (i.e. become known as HIV-infected or at "high risk" for HIV infection). For example, participants could be treated unfairly or discriminated against, or could have problems being accepted by their families and/or communities.

Participants in sites requiring partner notification in response to diagnosed STIs or HIV infection could have problems in their relationships with their sexual partners. Participants also could have problems in their partner relationships associated with use or attempted use of study products. In addition, participants could misunderstand the current experimental status of the study gels (i.e., their unknown safety and unproven efficacy) and as a result increase their HIV risk behaviors while in the study.

Anoscopy

Anoscopy is a commonly practiced medical procedure and the procedures done in this trial will not involve any unusual risks or discomforts. The risk associated with these procedures is a minor discomfort during insertion of the anoscope, in addition to a small amount of bleeding.

Flexible Sigmoidoscopy

Flexible sigmoidoscopy is a commonly practiced medical procedure and the endoscopic procedures done in this trial will not involve any unusual risks or discomforts. The risks associated with these procedures include mild discomfort and the feeling of having a "bloated stomach". Mild rectal irritation and urgency to move bowels may also occur.

Endoscopic biopsies are painless, begin to heal within 2 hours, and are completely healed within 3-5 days. On extremely rare occasions, the endoscopic procedure or biopsies may lead to pain, infection (sepsis), bleeding or perforation of the gastrointestinal tract. Perforation secondary to mucosal biopsy occurs approximately

once out of every 8,000 procedures. If this extremely rare complication occurs, antibiotics and surgery to repair the tear may be necessary.

Vaginal Swabs and Sponges
There is the risk of mild discomfort and/or pressure in the vagina and/or pelvis from both these procedures in addition to a slight risk of bleeding.

Rectal Swabs and Sponges
There is the risk of mild discomfort from both these procedures in addition to a slight risk of bleeding.

Enemas
The main risk from having an enema is temporary discomfort. A hollow soft plastic tube about the thickness of a pencil will be used to put approximately 120 mL of enema fluid into the rectum to stimulate a bowel movement with stool evacuation and flush it out (a larger volume may be required if the initial volume does not produce results). This may cause a “bloated” or “cramping” feeling. Some air may be pumped into the rectum as well, causing flatulence. The tube is small, but it might cause some anal or rectal discomfort if the subject has any hemorrhoids or other painful conditions.

Applicator
Use of a plastic-tipped, blunt-end vaginal applicator to deliver a vaginal microbicide into the rectal compartment may be associated with minor anorectal trauma including mild epithelial tears and bruising in the anorectal area. To minimize the risk of trauma during applicator insertion, a small amount of a commercially available iso-osmolar lubricant will be used to aid insertion by the clinical investigator.

Risk of Answering the Web-Based Questionnaires
There may be discomfort or embarrassment related to questions dealing with sexual behaviors, personal habits and experiences with rectal use of the study products.

Risk of Participating in Telephone Interview
Participants may feel embarrassed or uncomfortable when answering questions about experiences with rectal use of the study products. While not anticipated, there is also the potential risk of a violation of the participants’ privacy and confidentiality, in the event that someone overhears the telephone conversation. The telephone interviews will be recorded and transcribed.

The primary risk from participating in the telephone interviews concerns loss of confidentiality. To protect participants’ confidentiality during these interviews, all tapes of the interviews will be kept in a locked cabinet, and after the interviews have been transcribed and checked for accuracy, the tapes will be destroyed. Participants are free to suggest a name that can be used during the interviews.

Tenofovir 1% Gel

Vaginal Formulation (VF), Vaginal, Penile, and Rectal Applications (CONRAD IND 73,382 – authorization for review is stated in CONRAD's IND cross-reference letter): Administration of VF tenofovir gel intravaginally at 0.3% and 1% concentrations in the HPTN 050 Phase 1 study resulted in minimal local irritation and little or no systemic adverse effects were identified [59]. Although 92% of participants reported at least 1 AE, 87% of those reported AEs were mild, and 70% of the AEs were limited to the genitourinary tract. Four severe AEs were reported, with only one, lower abdominal pain, thought to be product-related. The risks associated with tenofovir gel are believed to be less than those identified for systemic use. In the HPTN 050 Phase 1 study of tenofovir gel, serum PK analysis the cohort of HIV-seronegative women demonstrated 14 of 24 women (56%) had detectable levels (just above lower limit of quantification) of plasma tenofovir at any time point during the 14 day exposure [59]. There were no correlations between low but detectable level of plasma tenofovir and reported AEs. The relative bioavailability of vaginal compared to oral dosing of TFV (vs. TDF) is approximately 2%. In several subsequent studies, these results have been reproduced with less than half of women having measurable TFV in the blood after vaginal dosing with median TFV concentrations around 3 ng/mL. For reference, oral TDF dosing results in steady-state concentrations of TFV more than one hundred times higher.

Given that Phase 1 data for the topical application of TFV 1% gel (VF) demonstrate measurable plasma concentrations of tenofovir in some participants, participants with hepatitis B infection might be at risk for development of tenofovir resistant hepatitis B. However, participants with known chronic hepatitis B infection will not be eligible for enrollment.

It is not known what effect tenofovir gel could have on the HIV quasispecies or HIV disease progression in HIV infected participants or their partners. There is a theoretical risk that tenofovir absorbed systemically from the rectally-applied tenofovir gel could result in mutations of the HIV virus in participants who become infected with HIV during the study, or their partner, if the partner is infected with HIV. Limited resistance data from HPTN 050 show no new resistance mutations in plasma or CVL specimens after 14 days of tenofovir gel use. No participant had high level tenofovir resistance mutations (e.g., K65R). In addition, the recently completed CAPRISA 004 study did not demonstrate the development of tenofovir related resistance mutations in women exposed to tenofovir gel (VF, vaginal administration) who subsequently became infected with HIV [62].

Some of the possible side effects of the VF study gel are dryness, itching, burning, pain in the rectal area and a temporary increase in rectal urgency or leakage.

In the male tolerance study of penile application of VF tenofovir 1% gel, there were few urogenital findings observed after product use in a limited number of participants,

all findings were classified as mild, and required no treatment. The most common symptoms included mild penile pain (burning, irritation, discomfort) and pruritus [61].

There is currently no published safety data regarding rectal application of VF tenofovir 1% gel. The recently completed rectal safety study (RMP-02/MTN-006) has not yet been published, but the preliminary findings have been presented at CROI 2011 [44]. For detailed comments, see Sections 2.5.1.1.2 and 2.5.2.1.2. As is relevant here, the rectal exposure of VF tenofovir 1% gel was safe, as defined per protocol by the number of ≥ Grade 2 AE reported. No differences in Grade 2 AEs were seen between those receiving HEC placebo or the hyperosmolar VF tenofovir gel. Eight Grade 3 AEs occurred; two of these were in 2 subjects not receiving topical tenofovir gel. The other 6 of 8 Grade 3 AEs were seen in a total of 2 subjects and only during the 7-day exposure period. These were mostly gastrointestinal-related and thought to be due to the hyperosmolar nature of the VF. Both were in the tenofovir arm (not statistically significant) and resolved. The applied tenofovir-containing gel was associated with only a few, statistically significant mucosal changes (primarily in altered cytokine levels) compared to the HEC placebo group. Topically-applied tenofovir was detectable in plasma and tissue for a short period following single dose exposure and also following seven-day exposure.

Reduced Glycerin Vaginal Formulation (RGVF), Vaginal and Rectal Administration (CONRAD IND 73,382 – authorization for review is stated in CONRAD's IND cross-reference letter): As noted in Section 2, the formulation changes to the tenofovir 1% gel were minor. The glycerin reduction change was made without the need to perform another safety study, since it was a reduction in an excipient. Such reductions do not typically require additional safety evaluations (*FDA Guidance for Industry, Nonsterile Semisolid Dosage Forms – Scale-up and Post-Approval Changes*.) For rectal administration, two Phase 1 studies, MTN-007 and Project GEL, will evaluate the RGVF.

Rectal Formulation (RF), Rectal Administration: As previously mentioned, this CHARM-01 study, along with a companion CHARM-02 trial, will be the first rectal safety study of the new rectal specific formulation (RF) of tenofovir 1% gel.

Universal HEC Placebo Gel: Twice daily intravaginal administration of HEC gel over the course of two weeks resulted in mild genital irritation, including genital burning, soreness, and pelvic pain, in 2 out of 14 women (14.3%) [78, 79]. Three out of the 14 women (12.4%) had colposcopic findings which included erythema, petechiae and peeling, although no findings with deep disruption were observed during follow-up. HEC gel did not appear to alter vaginal health or shift vaginal flora and no SAEs were reported.

Two Phase 1 rectal microbicide trials completed to date have used HEC gel in the placebo arm. RMP-01 using topical UC781 enrolled 36 subjects with 12 randomized to the HEC placebo arm. Following single topical rectal HEC exposure, four Grade 2 AEs were reported in 1 individual, shown to be in the placebo group. As this

individual had no complaints during the subsequent 7-day exposure, the symptoms of fever, cramps, flatulence, and diarrhea were likely not related to the HEC gel. There were no other Grade 2 or Grade 3 AE associated with HEC gel [32].

In the second rectal microbicide Phase 1 safety trial, RMP-02/MTN-006, 18 subjects were studied with 6 randomized to the HEC placebo arm in the topical-exposed portion of the trial. With regards to gastrointestinal-related AEs following single-dose topical HEC exposure, no Grade 2 or Grade 3 AEs were reported. Following 7-day exposure, one Grade 2 event was reported in one subject receiving HEC gel (abdominal pain); three events were reported in 2 subjects in the tenofovir-treated group. None of the five gastrointestinal Grade 3 AEs reported in 2 subjects (all during 7-day exposure) were related to HEC placebo; all were in the tenofovir-product group. No SAEs were reported.

13.3.2 Benefits

Participants and others may benefit in the future from information learned from this study. Specifically, information learned in this study may lead to the development of safe and effective interventions to prevent HIV transmission. Participants also may appreciate the benefit of earlier diagnosis of STIs; additionally, participants will be referred for treatment for any incidental findings detected during screening and other study-related examinations.

13.4 Informed Consent Process

It is the responsibility of the study site investigator to ensure that the Elements of Informed Consent (21 CFR 50.25 and ICH GCP 4.8.10) and Health Insurance Portability Accountability Act (HIPAA) guidelines are followed and documented in the source document file. The process for obtaining informed consent from potential research participants should be clearly documented and appropriately filed within the site's standard operating procedures.

Written informed consent will be obtained from each study participant prior to both screening and enrollment. Written informed consent also will be obtained for long-term specimen storage and possible future testing, but consent for specimen storage and future testing is not required for study participation. In obtaining and documenting informed consent, the investigators and their sub-investigators will comply with applicable local and US regulatory requirements and will adhere to GCP and to the ethical principles that have their origin in the Declaration of Helsinki. Study staff must document the informed consent process in accordance with the Requirements for Source Documentation in DAIDS Funded and/or Sponsored Clinical Trials available at:

(http://www.niaid.nih.gov/LabsAndResources/resources/DAIDSClinRsrch/Documents/sourcedocpolicy.pdf).

Participants will be provided with copies of the informed consent forms if they are willing to receive them. Each study site is responsible for developing study informed consent forms for local use, based on the templates in the Appendices that describe the purpose of screening and of the study, the procedures to be followed, and the risks and benefits of participation, in accordance with all applicable regulations.

The informed consent process will cover all elements of informed consent required by research regulations. In addition, the process will specifically address the following topics of importance to this study:

- The unknown safety and unproven efficacy of the study products
- The need to practice safer sex behaviors regardless of study treatment group
- The importance of adherence to the study visit and procedures schedule
- The potential medical risks of study participation (and what to do if such risks are experienced)
- The potential social harms associated with study participation (and what to do if such harms are experienced)
- The real yet limited benefits of study participation
- The distinction between research and clinical care
- The right to withdraw from the study at any time

The informed consent process will include an assessment of each potential participant's understanding prior to enrollment and randomization of concepts identified by the protocol team as essential to the informed consent decision. Participants who are not able to demonstrate adequate understanding of key concepts after exhaustive educational efforts will not be enrolled in the study.

If during the trial a consent revision where new information that might affect the research participant's willingness to participate is presented, participants will be informed of the revisions. If a research participant terminates the study and consent form revision occurs after their participation has ended, they do not need to sign the revised consent form.

13.5 Participant Confidentiality

All study procedures will be conducted in private and every effort will be made to protect participant privacy and confidentiality to the extent possible. Each study site will implement confidentiality protections that reflect the local study implementation plan and the input of study staff and community representatives to identify potential confidentiality issues and strategies to address them. In addition to local considerations, the protections described below will be implemented at all sites.

All study-related information will be stored securely at the study site. All participant information will be stored in locked file cabinets in areas with access limited to study staff. Data collection, process and administrative forms, laboratory specimens and other reports will be identified by a coded number only to maintain participant

confidentiality. Forms, lists, logbooks, appointment books and any other listings that link participant ID numbers to other identifying information will be stored in a separate, locked file in an area with limited access. All local databases will be secured with password-protected access systems. Participants' study information will not be released without their written permission, except as necessary for review, monitoring, and/or auditing by:

- Representatives of the US Federal Government, including the US FDA, the US OHRP, NIH, and/or contractors of the NIH
- Representatives of the Sponsor
- CHARM Program Cores
- CONRAD
- Site IRBs

In addition, a Certificate of Confidentiality from the US Department of Health and Human Services will be obtained for this study. This Certificate protects study staff from being compelled to disclose study-related information by any US Federal, State or local civil, criminal, administrative, legislative or other proceedings. It thus serves to protect the identity and privacy of study participants.

13.6 Special Populations

This section outlines considerations made for the inclusion or exclusion of special populations in this study.

13.6.1 Pregnant Women

Women who test positive for pregnancy at screening or enrollment visits will not be eligible to participate in this study.

A urine pregnancy test will be performed on all women of childbearing potential at at visits 1, 2, 3, 4, 6, 7, 9, and 10 and additionally if clinically indicated. Investigators will discontinue study product among participants who test positive for pregnancy. During the informed consent process, women will be informed that none of the study products are methods of contraception and that the effects of these products on a developing human fetus are unknown. All potential participants will be required to be currently using a reliable method of contraception, such as hormonal contraception, intrauterine device, or sterilization.

13.6.2 Children

The NIH has mandated that children be included in research trials when appropriate. This study meets *Justifications for Exclusion* criteria for younger children as set forth by the NIH. Specifically, "insufficient data are available in adults to judge potential risk in children" and "children should not be the initial group to be involved in research

studies." This study does not plan to enroll children or adolescents under 18 years of age.

13.7 Compensation

Pending IRB approval, participants will be compensated for their time and effort in this study, and/or be reimbursed for travel to study visits, child care, and time away from work according to sites' practices. Reimbursement amounts will be specified in each study site's informed consent form.

13.8 Communicable Disease Reporting

Study staff will comply with all applicable local requirements to report communicable diseases including HIV identified among study participants to local health authorities. Participants will be made aware of all reporting requirements during the study informed consent process.

13.9 Access to HIV-related Care

13.9.1 HIV Counseling

HIV test-related counseling will be provided to all potential study participants who consent to undergo HIV screening to determine their eligibility for this study and to all enrolled participants at each follow-up HIV testing time point. Testing will be performed in accordance with the algorithm in Appendix II. Counseling will be provided in accordance with standard HIV counseling policies and methods at each site and additionally will emphasize the unknown efficacy of the study products in preventing HIV infection. In accordance with NIH, participants must receive their HIV test results to take part in this study. Referral for additional counseling related to testing or diagnosis will occur if needed or requested by the participant.

13.9.2 Care for Participants Identified as HIV-infected

Participants will be provided with their HIV test results in the context of post-test counseling. Per site SOPs, participants found to be HIV-infected will be referred to available sources of medical and psychosocial care and support, and local research studies for HIV-infected adults.

13.10 Study Discontinuation

This study may be discontinued at any time by NIAID, Sponsor, CONRAD, the US FDA, the OHRP, or site IRBs.

14 PUBLICATION POLICY

Publication of the results of this study will be governed by the policies of the CHARM IPCP and those of NIH. Any presentation, abstract, or manuscript will be made available for prior review to the CHARM's Scientific Review Committee as well as DAIDS, with the final decision remaining with the CHARM PI.

15 LIST OF APPENDICES

APPENDIX I: SCHEDULE OF STUDY VISITS AND EVALUATIONS
APPENDIX II: HIV TESTING ALGORITHM
APPENDIX III: TOXICITY TABLES
APPENDIX IV: HISTOPATHOLOGY SCORING SYSTEM
APPENDIX V: SAMPLE INFORMED CONSENT FORM
APPENDIX VI: SAMPLE INFORMED CONSENT FORM (SAMPLE STORAGE)

16 REFERENCES

1. McGowan I. Microbicides for HIV prevention: reality or hope? *Curr Opin Infect Dis* 2010; **23(1)**:26-31.

2. Stein ZA. HIV prevention: the need for methods women can use. *Am J Public Health* 1990; **80(4)**:460-462.

3. Carlos JA, Bingham TA, Stueve A, Lauby J, Ayala G, Millett GA*, et al.* The role of peer support on condom use among Black and Latino MSM in three urban areas. *AIDS Educ Prev* 2010; **22(5)**:430-444.

4. Gorbach PM, Manhart LE, Hess KL, Stoner BP, Martin DH, Holmes KK. Anal intercourse among young heterosexuals in three sexually transmitted disease clinics in the United States. *Sex Transm Dis* 2009; **36(4)**:193-198.

5. Beyrer C. Global prevention of HIV infection for neglected populations: men who have sex with men. *Clin Infect Dis* 2010; **50 Suppl 3**:S108-S113.

6. Mosher WD, Chandra A, Jones J. Sexual behavior and selected health measures: men and women 15-44 years of age, United States, 2002. In. Hyattsville, MD: National Center for Health Statistics; 2005.

7. Misegades L, Page-Shafer K, Halperin D, McFarland W. Anal intercourse among young low-income women in California: an overlooked risk factor for HIV? *AIDS* 2001; **15(4)**:534-535.

8. Karim SS, Ramjee G. Anal sex and HIV transmission in women. *Am J Public Health* 1998; **88(8)**:1265-1266.

9. Lane T, Pettifor A, Pascoe S, Fiamma A, Rees H. Heterosexual anal intercourse increases risk of HIV infection among young South African men. *AIDS* 2006; **20(1)**:123-125.

10. Kalichman SC, Simbayi LC, Cain D, Jooste S. Heterosexual anal intercourse among community and clinical settings in Cape Town, South Africa. *Sex Transm Infect* 2009; **85(6)**:411-415.

11. Baral S, Trapence G, Motimedi F, Umar E, Iipinge S, Dausab F*, et al.* HIV prevalence, risks for HIV infection, and human rights among men who have sex with men (MSM) in Malawi, Namibia, and Botswana. *PLoS ONE* 2009; **4(3)**:e4997.

12. Baral S, Sifakis F, Cleghorn F, Beyrer C. Elevated risk for HIV infection among men who have sex with men in low- and middle-income countries 2000-2006: a systematic review. *PLoS Med* 2007; **4(12)**:e339.

13. Koblin BA, Chesney MA, Husnik MJ, Bozeman S, Celum CL, Buchbinder S*, et al.* High-risk behaviors among men who have sex with men in 6 US cities: baseline data from the EXPLORE Study. *Am J Public Health* 2003; **93(6)**:926-932.

14. Gross M, Holte SE, Marmor M, Mwatha A, Koblin BA, Mayer KH. Anal sex among HIV-seronegative women at high risk of HIV exposure. The HIVNET Vaccine Preparedness Study 2 Protocol Team. *J Acquir Immune Defic Syndr* 2000; **24(4)**:393-398.

15. Civic D. College students' reasons for nonuse of condoms within dating relationships. *J Sex Marital Ther* 2000; **26(1)**:95-105.

16. Erickson PI, Bastani R, Maxwell AE, Marcus AC, Capell FJ, Yan KX. Prevalence of anal sex among heterosexuals in California and its relationship to other AIDS risk behaviors. *AIDS Educ Prev* 1995; **7(6)**:477-493.

17. Leynaert B, Downs AM, De V, I. Heterosexual transmission of human immunodeficiency virus: variability of infectivity throughout the course of infection. European Study Group on Heterosexual Transmission of HIV. *Am J Epidemiol* 1998; **148(1)**:88-96.

18. Vittinghoff E, Douglas J, Judson F, McKirnan D, MacQueen K, Buchbinder SP. Per-contact risk of human immunodeficiency virus transmission between male sexual partners. *Am J Epidemiol* 1999; **150(3)**:306-311.

19. Page-Shafer K, Shiboski CH, Osmond DH, Dilley J, McFarland W, Shiboski SC*, et al.* Risk of HIV infection attributable to oral sex among men who have sex with men and in the population of men who have sex with men. *AIDS* 2002; **16(17)**:2350-2352.

20. Kalichman SC, Rompa D, Luke W, Austin J. HIV transmission risk behaviours among HIV-positive persons in serodiscordant relationships. *Int J STD AIDS* 2002; **13(10)**:677-682.

21. Lapenta C, Boirivant M, Marini M, Santini SM, Logozzi M, Viora M*, et al.* Human intestinal lamina propria lymphocytes are naturally permissive to HIV-1 infection. *Eur J Immunol* 1999; **29(4)**:1202-1208.

22. Anton PA, Elliott J, Poles MA, McGowan IM, Matud J, Hultin LE*, et al.* Enhanced levels of functional HIV-1 co-receptors on human mucosal T cells demonstrated using intestinal biopsy tissue. *AIDS* 2000; **14(12)**:1761-1765.

23. Poles M, Elliot J, Shi D-P, Giorgi J., Hultin L., Anton P. Mucosal mononuclear cells (MMCs) are more infectable with HIV in vitro than peripheral blood mononuclear cells (PBMCs). *6th Conference on Retroviruses and Opportunistic Infections Chicago, IL,* 2001.

24. McGowan I. Microbicides: a new frontier in HIV prevention. *Biologicals* 2006; **34(4)**:241-255.

25. McGowan I. Rectal microbicides: a new focus for HIV prevention. *Sex Transm Infect* 2008; **84(6)**:413-417.

26. Tabet SR, Surawicz C, Horton S, Paradise M, Coletti AS, Gross M*, et al.* Safety and toxicity of nonoxynol-9 gel as a rectal microbicide. *Sex Transm Infect* 1999; **26(10)**:564-571.

27. Phillips DM, Taylor CL, Zacharopoulos VR, Maguire RA. Nonoxynol-9 causes rapid exfoliation of sheets of rectal epithelium. *Contraception* 2000; **62(3)**:149-154.

28. Phillips DM, Sudol KM, Taylor CL, Guichard L, Elsen R, Maguire RA. Lubricants containing N-9 may enhance rectal transmission of HIV and other STIs. *Contraception* 2004; **70(2)**:107-110.

29. Phillips DM, Zacharopoulos VR. Nonoxynol-9 enhances rectal infection by herpes simplex virus in mice. *Contraception* 1998; **57(5)**:341-348.

30. van de Wijgert JH, Shattock RJ. Vaginal microbicides: moving ahead after an unexpected setback. *AIDS* 2007; **21(18)**:2369-2376.

31. McGowan I, Elliott J, Cortina G, Tanner K, Siboliban C, Adler A*, et al.* Characterization of baseline intestinal mucosal indices of injury and inflammation in men for use in rectal microbicide trials (HIV Prevention Trials Network-056). *J Acquir Immune Defic Syndr* 2007; **46(4)**:417-425.

32. Anton PA, Saunders T, Elliott J, Khanukhova E, Dennis R, Adler A, Cortina G, Tanner K, Boscardin J, Cumberland W, Zhou Y, Ventuneac A, Carballo-Diéguez A, Rabe L, McCormick T, Gabelnick H, Mauck C, McGowan I. First phase 1 double-blind, placebo-controlled, randomized rectal microbicide trial using UC781 gel with a novel index of ex vivo efficacy, *PLoS One*, Accepted, July 2011

33. Elliott J, McGowan I, Adler A, Tanner K, Johnson EJ, Saunders T*, et al.* Strong suppression of HIV-1 infection of colorectal explants following *in vivo* rectal application of UC781 gel: A novel endpoint in a Phase I trial [Abstract]. *16th Conference on Retroviruses and Opportunistic Infections Montreal, Canada* 2009.

34. Veronese F, Anton P, Fletcher CV, DeGruttola V, McGowan I, Becker S*, et al.* Implications of HIV PrEP trials results. *AIDS Res Hum Retroviruses* 2011; **27(1)**:81-90.

35. Patton DL, Sweeney YT, Paul KJ. A summary of preclinical topical microbicide rectal safety and efficacy evaluations in a pigtailed macaque model. *Sex Transm Dis* 2009; **36(6)**:350-356.

36. Geboes K, Riddell R, Ost A, Jensfelt B, Persson T, Lofberg R. A reproducible grading scale for histological assessment of inflammation in ulcerative colitis. *Gut* 2000; **47(3)**:404-409.

37. McGowan I, Elliott J, Fuerst M, Taing P, Boscardin J, Poles M*, et al.* Increased HIV-1 mucosal replication is associated with generalized mucosal cytokine activation. *J Acquir Immune Defic Syndr* 2004; **37(2)**:1228-1236.

38. Doncel GF, Chandra N, Fichorova RN. Preclinical assessment of the proinflammatory potential of microbicide candidates. *J Acquir Immune Defic Syndr* 2004; **37 Suppl 3**:S174-S180.

39. Fichorova RN, Richardson-Harman N, Alfano M, Belec L, Carbonneil C, Chen S*, et al.* Biological and technical variables affecting immunoassay recovery of cytokines from human serum and simulated vaginal fluid: a multicenter study. *Anal Chem* 2008; **80(12)**:4741-4751.

40. Poullis A, Foster R, Mendall MA, Fagerhol MK. Emerging role of calprotectin in gastroenterology. *J Gastroenterol Hepatol* 2003; **18(7)**:756-762.

41. Roseth AG, Aadland E, Jahnsen J, Raknerud N. Assessment of disease activity in ulcerative colitis by faecal calprotectin, a novel granulocyte marker protein. *Digestion* 1997; **58(2)**:176-180.

42. Gaya DR, Lyon TD, Duncan A, Neilly JB, Han S, Howell J*, et al.* Faecal calprotectin in the assessment of Crohn's disease activity. *QJM* 2005; **98(6)**:435-441.

43. Tamboli CP, Richard F, Colombel JF. Fecal calprotectin in Crohn's disease: new family ties. *Gastroenterology* 2003; **124(7)**:1972-1974.

44. Anton P, Cranston R, Carballo-Dieguez AKA, Khanukhova E, Elliott J, Janocko LE*, et al.* RMP-02/MTN-006: A phase 1 placebo-controlled trial of rectally applied 1% vaginal TFV gel with comparison to oral TDF [Abstract]. *18th Conference on Retroviruses and Opportunistic Infections Boston* 2011.

45. Patton DL, Cosgrove-Sweeney YT, Rabe LK, Hillier SL. The pig-tailed macaque rectal model: microflora and chlamydial infection. *Sex Transm Dis* 2001; **28(7)**:363-366.

46. Richardson-Harman N, Lackman-Smith C, Fletcher PS, Anton PA, Bremer JW, Dezzutti CS*, et al.* Multisite comparison of anti-human immunodeficiency virus microbicide activity in explant assays using a novel endpoint analysis. *J Clin Microbiol* 2009; **47(11)**:3530-3539.

47. Abner SR, Guenthner PC, Guarner J, Hancock KA, Cummins JE, Jr., Fink A*, et al.* A Human Colorectal Explant Culture to Evaluate Topical Microbicides for the Prevention of HIV Infection. *J Infect Dis* 2005; **192(9)**:1545-1556.

48. Fletcher PS, Elliott J, Grivel JC, Margolis L, Anton P, McGowan I*, et al.* Ex vivo culture of human colorectal tissue for the evaluation of candidate microbicides. *AIDS* 2006; **20(9)**:1237-1245.

49. Robinson RJ, Stone M, Mayberry JF. Sigmoidoscopy and rectal biopsy: a survey of current UK practice. *Eur J Gastroenterol Hepatol* 1996; **8(2)**:149-151.

50. CONRAD. Tenofovir Gel (GS-1278) Investigator's Brochure. Fourth Edition, November 12, 2010.

51. Fuchs EJ, Lee LA, Torbenson MS, Parsons TL, Bakshi RP, Guidos AM*, et al.* Hyperosmolar sexual lubricant causes epithelial damage in the distal colon: potential implication for HIV transmission. *J Infect Dis* 2007; **195(5)**:703-710.

52. Dezzutti C, Rohan LC, Lynam J, Wang L, Friend D. TFV reformulation results in improved product safety for rectal application. [Abstract]. *18th Conference on Retroviruses and Opportunistic Infections Boston* 2011.

53. Gilead Sciences. Viread Investigator's Brochure. September 2006.

54. Gilead Sciences. Viread (tenofovir disoproxil fumarate) tablets. Package Insert. November 2008.

55. Gilead Sciences. Viread company core safety information. September 2006.

56. Weber J, Chakraborty B, Weberova J, Miller MD, Quinones-Mateu ME. Diminished replicative fitness of primary human immunodeficiency virus type 1 isolates harboring the K65R mutation. *J Clin Microbiol* 2005; **43(3)**:1395-1400.

57. Parikh UM, Dobard C, Sharma S, Cong ME, Jia H, Martin A*, et al.* Complete protection from repeated vaginal simian-human immunodeficiency virus exposures in macaques by a topical gel containing tenofovir alone or with emtricitabine. *J Virol* 2009; **83(20)**:10358-10365.

58. Cranage M, Sharpe S, Herrera C, Cope A, Dennis M, Berry N*, et al.* Prevention of SIV rectal transmission and priming of T cell responses in macaques after local pre-exposure application of tenofovir gel. *PLoS Med* 2008; **5(8)**:e157.

59. Mayer KH, Maslankowski LA, Gai F, El-Sadr WM, Justman J, Kwiecien A*, et al.* Safety and tolerability of tenofovir vaginal gel in abstinent and sexually active HIV-infected and uninfected women. *AIDS* 2006; **20(4)**:543-551.

60. Hendrix C, Minnis A, Guddera V, Riddler S, Salata R, Nakabiito C*, et al.* MTN-001: A phase 2 cross-over study of daily oral and vaginal TFV in healthy, sexually active women results in significantly different product acceptability and vaginal tissue drug concentrations. [Abstract]. *18th Conference on Retroviruses and Opportunistic Infections Boston* 2011.

61. Schwartz JL, Poindexter A, Wheeless A, Mauck CK, Callahan MM. Safety evaluation of 1% tenofovir gel in healthy men. *Int J STD AIDS* 2009; **20(6)**:384-386.

62. Abdool KQ, Abdool Karim SS, Frohlich JA, Grobler AC, Baxter C, Mansoor LE*, et al.* Effectiveness and safety of tenofovir gel, an antiretroviral microbicide, for the prevention of HIV infection in women. *Science* 2010; **329(5996)**:1168-1174.

63. Grant RM, Lama JR, Anderson PL, McMahan V, Liu AY, Vargas L*, et al.* Preexposure chemoprophylaxis for HIV prevention in men who have sex with men. *N Engl J Med* 2010; **363(27)**:2587-2599.

64. Grivel JC, Elliott J, Lisco A, Biancotto A, Condack C, Shattock RJ*, et al.* HIV-1 pathogenesis differs in rectosigmoid and tonsillar tissues infected ex vivo with CCR5- and CXCR4-tropic HIV-1. *AIDS* 2007; **21(10)**:1263-1272.

65. Morrow KM, Ruiz MS, Assessing microbicide acceptability: a comprehensive and integrated approach, *AIDS Behav* 2008; 12:272–283

66. Coly A, Gorbach PM, Microbicide acceptability research: recent findings and evolution across phases of product development, *Current Opinion in HIV and AIDS* 2008, 3:581–586

67. Ventuneac A, Carballo-Diéguez A, Acceptability of UC781 gel as a rectal microbicide among HIV-uninfected women and men, *AIDS Behav* 2010; 14:618–628

68. Mantella JE, Myerc L, Microbicide acceptability research: current approaches and future directions, *Social Science & Medicine* 60 (2005) 319–330

69. Haberer JE, Kahane J, Kigozi I, Emenyonu N, Hunt P, Martin J, Bangsberg DR Real-time adherence monitoring for HIV antiretroviral therapy, *AIDS Behav* (2010) Dec;14(6):1340-6.

70. Gengiah T, Mansoor L, Naidoo A, The “wisebag:” an innovative strategy for enhancing measurement of microbicide gel use in clinical trials, *Microbicide conference 2010*, Session 16, Sunday, May 23, 2010 Abstract 57 (abstract and oral presentation)

71. Nuttall J, Kashuba A, Wang R, White N, Allen P, Roberts J, Romano J. The pharmacokinetics of tenofovir following intravaginal and intrarectal

administration of tenofovir gel to rhesus macaques in a microbicide trial [Abstract #40]. *Microbicides 2010: Building Bridges in HIV Prevention*, Pittsburgh, PA, May 22-25, 2010

72. Tien D, Schnaare RL, Kang F, et al. In vitro and in vivo characterization of a potential universal placebo designed for use in vaginal microbicide clinical trials. AIDS Research & Human Retroviruses 2005; 21(10):845-53

73. Dezzutti CS, James VN, Ramos A, et al. In vitro comparison of topical microbicides for prevention of human immunodeficiency virus type 1 transmission. Antimicrobial Agents & Chemotherapy 2004; 48(10):3834-44

74. Patty's Industrial Hygiene and Toxicology Online. http://www3intersciencewileycom/cgi-bin/mrwhome/104554795/HOME; accessed 4/25/08: Section 43.4

75. Guttner J, Klaus S, Heinecke H. Embryotoxizitat intraperitoneal applizierter Hydroxyethylcellulose bei der Maus. Anatomischer Anzeiger 1981; 149(3):282-5

76. http://depts.washington.edu/terisweb/teris/index.html. In; accessed January 8, 2008

77. Dreisbach R. Handbook of Poisoning. 9 ed. Los Altos, CA: Lang Medical Publications; 1977

78. Schwartz JL, Ballagh SA, Kwok C, et al. Fourteen-day safety and acceptability study of the universal placebo gel. Contraception 2007; 75(2):136-41

79. CONRAD. Investigator's Brochure: Universal HEC Placebo Gel. September 2011; Edition 4.0.

80. Press release: MTN Statement on Decision to Discontinue Use of Tenofovir Gel in VOICE, a Major HIV Prevention Study in Women http://www.mtnstopshiv.org/sites/default/files/attachments/MTNStatementNov17DSMB_final.pdf

APPENDIX I: SCHEDULE OF STUDY VISITS AND EVALUATIONS

	Screening	Baseline	Dosing												Exit
			First Formulation				Second Formulation				Third Formulation				
			1st Dose	5 Take-home Doses	7th Dose	24-hr Post	1st Dose	5 Take-home Doses	7th Dose	24-hr Post	1st Dose	5 Take-home Doses	7th Dose	24-hr Post	Phone Call
**Visit**	**1**	**2**	**3**	**n/a**	**4**	**5**	**6**	**n/a**	**7**	**8**	**9**	**n/a**	**10**	**11**	**Exit**
**Administrative**															
Informed consent	X	confirm													
Eligibility determination	X	confirm													
Randomization†		X													
Locator Info	X	X	X		X	X	X		X	X	X		X	X	
Counseling	X	X	X		X		X		X		X		X		
**Clinical Evaluation**															
Med history	X	X	X		X		X		X		X		X		X
Menstrual history♀	X	X	X		X		X		X		X		X		
AE/ concomitant med assessment	Con meds only	Con meds only	X		X	X	X		X	X	X		X	X	X
Physical exam with DRE*	X	X	X		X		X		X		X		X		
Flexible sigmoidoscopy with biopsies		X			X				X				X		
Enema		X			X				X				X		
**Safety labs**															
CBC with differential & PLT	X	X													
BUN, creatinine, ALT, AST	X	X													
Hepatitis BsAb	X														
Hepatitis BsAg	X													X	
PT/INR	X														
UA-dipstick	X	X	X				X				X				
Urine hCG♀	X	X	X		Pre Dose		X		Pre Dose		X		Pre Dose		
**BV and pH**															
BV and vaginal pH ♀	X														
**STI screen**															
HIV	X	X	X				X				X				
Plasma archive	X	X													
GC/CT (urine and rectal)	X	X	X				X				X				
RPR	X														
HSV serology	X														
**PK Assays**															
Plasma & PBMC		X			X + 2hr, 4hr	X			X + 2hr, 4hr	X			X + 2hr, 4hr	X	
Vaginal fluid♀		X			X + 2hr, 4hr	X			X + 2hr, 4hr	X			X + 2hr, 4hr	X	
Rectal fluid		X			X + 2hr, 4hr	X			X + 2hr, 4hr	X			X + 2hr, 4hr	X	
Whole tissue & MMC		X			X				X				X		
**Rectal Assays**															
Microflora			X			X	X			X	X			X	
Cytokine expression (by Luminex)		X	X		Pre Dose	X	X		Pre Dose	X	X		Pre Dose	X	
Cytokine mRNA profile		X			X				X				X		
Histopathology		X			X				X				X		

MMC phenotype		X			X				X				X		
Explants (p24)		X			X				X				X		
**Study Product**			**X**	**X**	**X**		**X**	**X**	**X**		**X**	**X**	**X**		
**Behavioral Evaluation**															
Baseline behavioral questionnaire		X													
Gel-use questionnaire						X				X				X	
Application process questionnaire														X	
Acceptability phone interview															X

*DRE Digital rectal exam
♀ Female participants
† Randomization is completed between Visit 2 and Visit 3

APPENDIX II: HIV TESTING ALGORITHM

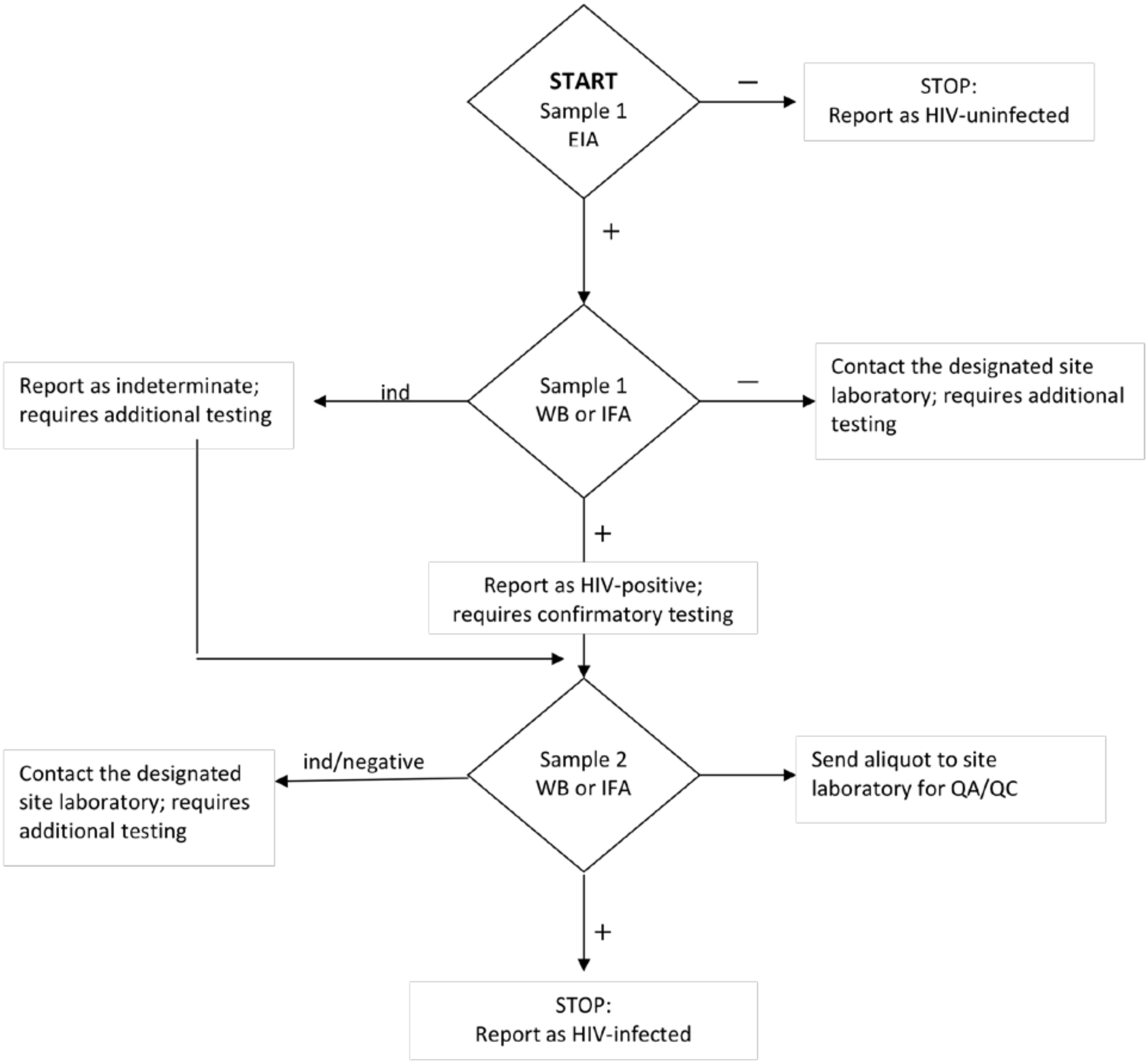

APPENDIX III: TOXICITY TABLES

The *Division of AIDS Rectal Grading Table for Use in Microbicide Studies (DAIDS AE Grading Table Addendum 3)* will be the primary tool for grading adverse events for this protocol.

Adverse events not included in the *Rectal Grading Table* will be graded by the most current *Division of AIDS Table for Grading the Severity of Adult and Pediatric Adverse Events (DAIDS AE Grading Table, Version 1.0 dated December 2004, Clarification dated August 2009).*

In cases where an AE is covered in both tables, *DAIDS AE Grading Table Addendum 3* will be the grading scale utilized.

All Division of AIDS grading tables are available online at: http://rcc.tech-res.com/safetyandpharmacovigilance/.

APPENDIX IV: HISTOPATHOLOGY SCORING SYSTEM

Participant ID: _________ Visit No.: _______ Visit Date: ______

**Please Circle the Grade**
**Grade 0**<br>**No abnormality**
**Grade 1**<br>**Mononuclear cell infiltrate**
**Grade 2**<br>**Neutrophilic infiltrate-lamina propria**
**Grade 3**<br>**Neutrophilic infiltrate-epithelium**
**Grade 4**<br>**Crypt destruction**
**Grade 5**<br>**Erosion or ulceration**

APPENDIX V: SAMPLE INFORMED CONSENT FORM

This is a sample consent form document only. Each participating site is responsible for developing its own informed consent documents. Institutions may require specific informed consent language (e.g., HIPAA language) and have different policies on compensation.

Full Title: CHARM-01 – A Randomized, Double Blind Phase 1 Safety, Accceptability, and Pharmacokinetic Study Comparing Three Formulations of Tenofovir 1% Gel Administered Rectally to HIV-1 Seronegative Adults

Short Title: CHARM-01

Sponsor(s): The study is being paid for by the Division of AIDS, US National Institute of Allergy and Infectious Diseases of the National Institutes of Health and is part of the Combination HIV Antiretroviral Rectal Microbicide (CHARM) Program.

Study Products: Under direction from CONRAD, DPT Laboratories will provide the vaginal formulation (VF) of the tenofovir 1% gel, the reduced glycerin vaginal formulation (RGVF) of the tenofovir 1% gel, and the Univeral HEC placebo gel,. DPT Laboratories will also provide the rectal formulation (RF) of the tenofovir 1% gel.

Principal Investigator: *[INSERT NAME]*
Key Study Staff: *[INSERT NAME(S)]*
Phone: *[INSERT NUMBER(S)]*

INTRODUCTION

You are being asked to participate in a research study investigating the safety and acceptability of various gel formulations (mixtures) containing the same amount of the anti-HIV drug tenofovir when applied rectally. Investigators are hopeful that the tenofovir gel will be developed into something called a microbicide that will help prevent Human Immunodeficiency Virus (HIV) infection. A microbicide is a product that kills bacteria and viruses.

Tenofovir gel is *experimental* for HIV prevention. This means we do not know if it works to protect against HIV. You will use three different types (called "formulations") of the gel in this study. In future studies, we would like to see if tenofovir gel, when inserted into the rectum, could prevent HIV. In order to do this, first we need to make sure that tenofovir is safe for use in the rectum and where tenofovir goes in the body. Tenofovir gel is not approved by the US Food and Drug Administration (FDA) for use in the rectum.

A total of eighteen male and female volunteers will be enrolled in this study from two universities – University of California, Los Angeles (UCLA) and Magee Womens Research Institute in Pittsburgh. If you agree to take part in this study, study

investigators will need to see if you are healthy enough to participate. Participating in this study requires 11 clinic visits (including the visit today) and a final phone call over approximately 3-4 months.

If you join the study, you will be asked to not put anything in your rectum for 72 hours before and after having tissue samples taken, because this may alter the study results and you may be at higher risk for bleeding or getting or spreading an infection until the biopsy sites have fully healed.

To decrease the chance of bleeding after tissue samples are taken, you will be asked to avoid taking any of the following medications:

- Heparin, including Lovenox®
- Coumadin® (warfarin)
- Plavix® (clopidogrel bisulfate)
- More than 81mg of aspirin per day
- Non-steroidal anti-inflammatory drugs such as Motrin, Advil or Aleve. These medications increase the risk of bleeding. Tylenol is allowed.
- Any other drugs that are associated with increased likelihood of bleeding following rectal biopsy

You will also be asked to avoid using the following products or medications, which are likely to alter the study results:

- Tenofovir, acyclovir, valacyclovir, or famciclovir
- Any products rectally that contain the chemical nonoxyl-9 (N-9). A list of some products containing N-9 is attached to this consent form. This list is not complete. You should check the label of any products you use rectally to be sure that they do not contain N-9. N-9 has been shown to change the rectal lining, which could result in injury to you.

Prior to joining the study, you will have an HIV test done, and we will also ask you questions about the HIV status of your current partner(s). If you have HIV, you will not be able to join the study, and you will learn from the study staff where you can get care or treatment.

YOUR PARTICIPATION IN THIS STUDY IS VOLUNTARY

This consent form gives you information about this research study and all study procedures. The study staff will discuss this information with you. You are free to ask questions about this study at any time. Once you understand the study procedures and if you agree to take part, you will be asked to sign your name on this consent form. A copy of this form will be offered for you to keep.

Before you learn about this study, it is important that you know the following:

- You do not have to be in this study if you do not want to;

- You may decide not to take part in the study procedures and may withdraw from the study at any time;
- Some people may not be able to join the research study because of information found during the screening process.
- You will receive results of your lab tests even if you are not eligible to join the study.

DISCLOSURE STATEMENT

[INSERT THE FOLLOWING SECTION AS REQUIRED PER INSTITUTIONAL SITE STANDARD]

Your health care provider may be an investigator of this research protocol, and as an investigator, is interested in both your clinical welfare and in the conduct of this study. Before entering this study or at any time during the research, you may ask for a second opinion about your care from another doctor who is in no way associated with this project. You are not under any obligation to participate in any research project offered by your physician.

This study is sponsored by the U.S. National Institutes of Health (NIH) and is part of the Combination HIV Antiretroviral Rectal Microbicide (CHARM) Program. Under direction from CONRAD, DPT Laboratories will provide the vaginal formulation (VF) of the tenofovir 1% gel, the reduced glycerin vaginal formulation (RGVF) of the tenofovir 1% gel, and the Universal HEC placebo gel. DPT Laboratories will also provide the rectal formulation (RF) of the tenofovir 1% gel. The person in charge of this study at this site is *[INSERT NAME OF PRINCIPAL INVESTIGATOR]*.

PURPOSE OF THE STUDY

This study has three main purposes. The first one is to find out whether the study gels cause any side effects when inserted into the rectum. The second is to find out how the study gels affect human cells and tissues. And the third is to find out which of the study gels is best for rectal use.

Three different formulations tenofovir gel are being tested in this study, and they should only be used rectally (not vaginally): the vaginal formulation (VF), reduced glycerin vaginal formulation (RGFV), and rectal formulation (RF). These gels are not approved for rectal use. This study will be the first study in humans using the RF formulation, and only preliminary findings of the rectal application of the VF formulation are available (no currently published data are available).

This study, taking place at University of California, Los Angeles (UCLA) and Magee Womens Research Institute in Pittsburgh, and a companion study, taking place at the Johns Hopkins University in Baltimore, are the first studies in humans that use the rectal formulation (RF) of tenofovir gel.

STUDY GELS AND STUDY STAGES

This study has three study stages, and you will receive seven doses of different formulations in each stage. The order in which these study stages occur will be chosen at random, which means by chance, like flipping a coin or throwing dice. You will not know and cannot choose the order in which you receive your gels doses and the study staff cannot choose it for you. The study staff and study doctor will not know the order of your gel doses either.

In two of the study stages you will receive full seven doses of the reduced glycerin vaginal formulation (RGFV) and the rectal formulation (RF), but in the third stage you will receive six doses of placebo formulation gel which does not have any medicine in it, followed by only one dose of the the vaginal formulation (VF). You won't know which stages these will be, since their order will be chosen at random.

During each stage, you will receive your first gel dose in clinic, inserted rectally by a study clinician, using an applicator. You will then take five applicators home with you (plus one extra for emergency use) and insert them at home, one per day for five days. Finally, you will come back to clinic to receive your seventh dose and have different biological specimens collected, including blood, urine, and samples from your rectum which will be collected at different time points, including at 24 hours after application of the seventh dose. *Procedures* section below explains in detail what will happen at each visit, including behavioral questionnaires which you will be asked to complete when you first begin the study and then as you complete each study stage.

PROCEDURES

If you decide to take part in the study, your visit will continue today after you read, discuss, understand, and sign this form.

All study visits will take approximately 1-5 hours. You will be in the study for about 3-4 months from the time of today's visit until your follow-up phone call at the end of the study. Please, review all the visits described below and ask questions about anything you do not understand.

Visit 1 – Screening Visit

The Screening Visit will take about 2 hours. You will be asked to complete the following procedures:

- Sign this form after you have reviewed it carefully and had the chance to ask questions about the study
- Answer questions about yourself, such as your medical history, any medicines that you are taking and how we can contact you.
    - If you are a female participant, you will be asked about your menstrual history

- Have a physical exam, including an examination of your anus and rectum
- Learn from study staff how to: avoid infections passed during sex, use condoms, and prevent pregnancy
- Receive condoms from the study staff.
- Provide urine sample for urinalysis, gonorrhea, and Chlamydia testing
    - If you are a female participant, your urine may also be tested for pregnancy
- Have rectal specimens collected by a study clinician to be tested for gonorrhea and Chlamydia
- If you are a female participant, you will be asked to collect samples of fluid from your vagina to test the pH levels of your vaginal fluid and for Bacterial Vaginosis (or BV, which is a condition in women where the normal balance of bacteria in the vagina is disrupted and replaced by an overgrowth of certain bacteria). You will be given instructions and some privacy to collect these samples.
- Have a blood sample (about 30 mL or about 6 teaspoons) taken to check the following:
    - If you have a chronic Hepatitis B infection
    - the health of your blood, liver, kidneys, and clotting factors
    - Hepatitis B vaccination status (this is to see if you've been vaccinated for Hepatitis B or have natural immunity to Hepatitis B)
    - Syphilis status
    - Herpes status
    - HIV status
    - Double-check your HIV status, if necessary (this blood sample is saved in case further testing is needed)

[INSERT THE FOLLOWING SECTION IF REQUIRED BY SITE IRB]:

The HIV test is a test for the antibody to HIV. An antibody is a substance that blood cells make to fight infection. A positive HIV test means that your blood sample tested positive for HIV and that repeat testing will be performed to confirm (prove) this finding. If your sample is proved to be positive for HIV, it means that you are a carrier of HIV. It also means that you can pass the virus to others through sexual intercourse, by sharing needles, and through donating blood and organs. A negative HIV test means that at this time, no antibody to HIV was found in your sample based on the result of the initial screening test, repeat screening tests, or a confirmatory test.

There can be individuals who have HIV test results that are called "false positive." That means that, for some reason, the test shows that HIV antibodies are present in the blood when they are not. There can also be "false negative" results. That means that the test is negative when HIV is actually already present in the blood. This happens when the person has been infected with HIV, but that person's body has not yet made antibodies to the virus, or HIV antibody is present in the person's blood, but for some reason the test failed to detect it.

You will also be counseled about the risks for transmitting HIV to others, risks for developing AIDS, and the available treatments for HIV infection. You will return to the

clinic to receive results from this repeat test, but will no longer be tested in the clinic for HIV antibody. If you have HIV, you will not be able to join the study, and you will learn from the study staff where you can get care or treatment.

It may take about 2 weeks to get the results of the rest of your tests. We will give you the test results when they are available. If you have any infections, you will learn from the study staff where you can get care or treatment. If the results of your screening tests show that you are able to continue your participation in this study, the study staff will contact you to schedule Visit 2.

Visit 2 – Baseline Visit

About 1-2 weeks after Visit 1, you will return for a clinic visit. At this visit, you will:

- Confirm that you want to continue to participate in this study
- Let us know if there are any changes in where you live or how we may contact you
- Receive a copy of your test results, if appropriate, and review these results with the study staff
- Tell us about any changes in your medical history
    - If you are a female participant, you will be asked about your menstrual history
- Tell us if there have been changes to any medicines you are taking now
- Have a physical exam, including an examination of your anus and rectum
- Learn from study staff how to: avoid infections passed during sex, use condoms, and prevent pregnancy
- Receive condoms from the study staff.
- Have blood samples (about 45 mL or about 9 teaspoons) taken to check the following:
    - the health of your blood, liver, and kidneys
    - HIV status
    - Double-check your HIV status, if necessary (this blood sample is saved in case further testing is needed)
    - Test for levels of tenofovir in the blood
- Provide a urine sample for urinalysis, gonorrhea, and Chlamydia testing
    - If you are a female participant, your urine may also be tested for pregnancy
- If you are a female participant, you will be asked to self-collect samples of fluid from your vagina. This sample will be used to compare against vaginal fluid that will be collected from you after you receive the study gels. You will be given instructions and some privacy to collect these samples.
- Have rectal specimens collected by a study clinician to be tested for gonorrhea, Chlamydia, signs of inflammation, and for tenofovir levels.
- Have an enema administered to prepare your rectum for a flexible sigmoidoscopy procedure (see below).
- Have a flexible sigmoidoscopy procedure performed, so that tissue samples (also known as "biopsies"; approximately 21 total) can be collected from your rectum. Flexible sigmoidoscopy is a procedure that lets your doctor examine the inside of your large intestine from the rectum and through the lower part of your colon. It is

important that you do not put anything in your rectum for the 72 hours before and after having tissue samples taken, because this may alter the study results and you may be at higher risk for bleeding or getting or spreading an infection until the biopsy sites have healed

- Complete a computerized questionnaire about your sexual behavior and sexual history. This should take about 20-25 minutes to complete.

Visits 3, 6, and 9

Visits 3, 6, and 9 will be approximately four weeks apart. At these visits, you will:

- Let us know if there are any changes in where you live or how we may contact you
- Receive a copy of your test results, if appropriate, and review these results with the study staff
- Tell us about any changes in your medical history
    - o If you are a female participant, you will be asked about your menstrual history
- Tell us if there have been changes to any medicines you are taking now
- Have a physical exam, including an examination of your anus and rectum
- Learn from study staff how to: avoid infections passed during sex, use condoms, and prevent pregnancy
- Receive condoms from the study staff.
- Have about 1 teaspoon of blood collected to check your HIV status
- Provide a urine sample for urinalysis, gonorrhea, and Chlamydia testing.
    - o If you are a female participant, your urine may also be tested for pregnancy
- Have rectal specimens collected by a study clinician to be tested for gonorrhea, Chlamydia
- Have fluid specimens taken from your rectum to be tested for bacteria and signs of inflammation
- **Study gel administration:** You will have the first dose of one of the study gels inserted into your rectum by a study clinician.

Take-home use of study gel

You will receive 6 applicators for a 5-day supply of study gel to take home. Study staff will remind you to keep a record of your gel use, provide you with a study diary (called *Product Use Log*), and schedule a daily phone call from study staff for the 5 days you'll be using the gel at home. The 5-day applicator supply of applicators will be given to you in something called a *Wisebag*™. This is a container the size of a lunch-bag, which will register when it is opened and can send this information to study staff through build-in wireless technology. This information will be used to monitor (via a secure online system) when an applicator is taken out of the bag and will be checked prior to each daily phone call.

Visits 4, 7, and 10

Visits 4, 7, and 10 will take place after you finish taking your five doses at home, which will be five days after Visits 3, 6, and 9, respectively. At these visits, you will:

- Let us know if there are any changes in where you live or how we may contact you
- Receive a copy of your test results, if appropriate, and review these results with the study staff
- Tell us about any changes in your medical history
  - If you are a female participant, you will be asked about your menstrual history
- Tell us if there have been changes to any medicines you are taking now
- Have a physical exam, including an examination of your anus and rectum
- Receive condoms from the study staff.
- Provide a urine sample for a pregnancy test, if you are a female participant
- Have fluid specimens taken from your rectum to be tested for bacteria and signs of inflammation
- Have an enema administered
- **Study gel administration:** You will have the seventh dose of one of the study gels inserted into your rectum by a study clinician.
- About 30 minutes after you receive the gel, you will:
  - Have a blood sample (about 25 mL or about 5 teaspoons) collected to check for tenofovir levels in the blood
  - Fluid specimens taken from your rectum to be tested for bacteria, signs of inflammation, and levels of tenofovir
  - If you are a female participant, you will be asked to self-collect samples of fluid from your vagina to test for tenofovir. You will be given instructions and some privacy to collect these samples.
  - Have a flexible sigmoidoscopy procedure performed, so that tissue samples (approximately 21) can be collected. It is important that you do not put anything in your rectum for the 72 hours before and after having tissue samples taken, because this may alter the study results and you may be at higher risk for bleeding or getting or spreading an infection until the biopsy sites have healed
- Approximately 2 hours and 4 hours after you receive the study gel, you will have:
  - Additional blood samples (about 25 mL or about 5 teaspoons at each time point) collected to check for tenofovir levels in the blood
  - Fluid specimens taken from your rectum to be tested for bacteria, signs of inflammation, and levels of tenofovir
  - If you are a female participant, you will be asked to self-collect samples of fluid from your vagina to test for tenofovir. You will be given instructions and some privacy to collect these samples

<u>Visits 5, 8, and 11</u>

Visits 5, 8, and 11 will occur the day after Visits 4, 7, and 10, respectively. At these visits, you will:

- Let us know if there are any changes in where you live or how we may contact you

- Receive a copy of your test results, if appropriate, and review these results with the study staff
- Tell us about any changes in your medical history and tell us if there have been changes to any medicines you are taking now
- Receive condoms from the study staff
- Have a blood sample (about 25 mL or about 5 teaspoons) collected to check for tenofovir levels in the blood. In addition, at **Visit 11** ony, you will also have about 4mL or a little less than a teaspoon collected to test for Hepatitis B infection.
- Have fluid specimens taken from your rectum to be tested for bacteria, signs of inflammation, and levels of tenofovir
- If you are a female participant, you will be asked to self-collect samples of fluid from your vagina to test for tenofovir. You will be given instructions and some privacy to collect these samples.
- Complete a computerized questionnaire about your use of the study gel. You will also be asked which product you thought you were receiving during each study stage. This questionnaire should take about 20-25 minutes to complete.
- At **Visit 11 only**, in addition to the questionnaire about the use of study gel, we will ask you to complete one more questionnaire about using the applicator. This should also take about 20-25 minutes to complete.
- Also at **Visit 11 only,** we will ask you to take part in an in-depth interview (called Acceptability Phone Interview) with one of our researchers to discuss what your experiences were like in using the gel. The interview should take about 20-30 minutes and will be audiotaped. You can use a pseudonym (a made-up name) during the interview to protect your identity.

Final Phone Call/Visit

Study staff will call you within 7-14 days of your last clinic visit (Visit 11). During this phone call, we will ask you to:

- Tell us about any side effects you might have had from the last product administration in clinic and any medications you have taken
- If there are any final test results available, we will review them with you as well

Please, tell the study staff about any medical problems you have during the study. You can contact the study staff between your regular visits to report these problems. At each study visit, the study staff will review any side effects you might be experiencing from study products and/or study procedures.

Interim Visits

In some cases, an extra visit or visits (called Interim Visits) might be necessary in between your scheduled study appointments. Sometimes these visits may be necessary to address any questions you might have. At other times, the Interim Visits may occur if you experience side effects that need to be evaluated by study staff. In such cases, study staff may refer you to appropriate medical care.

SAMPLES COLLECTED IN THIS STUDY

Samples collected from you in this study may be used to develop and/or improve new laboratory tests. These samples will NOT be identified as coming from you. Specimens and data collected from you during the study will be stored at the designated laboratory at least until the end of the study or early study termination. For any samples left over after the study is completed, we will ask your permission to keep them for future research (please, see *Sample Storage Consent Form*).

If you do not enroll in the study after the first screening visit (Visit 1), any left-over samples collected from you will be destroyed.

POTENTIAL RISKS AND DISCOMFORTS

Risk of Blood Draws
You may feel discomfort when your blood is drawn. You may also feel dizzy, faint, and lightheaded and/or may have bruising, swelling, or infection at the site of injection.

Risk of Rectal Exams
You may feel discomfort or pressure when your rectum is examined.

Risks of Anoscopy
You may experience minor discomfort during insertion of the anoscope (i.e., small plastic tube inserted approximately 2 inches into your rectum to view the inside of your anus and rectum), in addition to a small amount of bleeding.

Risks from Rectal Swabs and Sponges
You may experience some mild discomfort and pressure in your rectum. In some cases, a small amount of bleeding may occur.

Risks of Vaginal Swabs and Sponges
You may experience some mild discomfort and/or pressure in your genital and pelvic area. Slight vaginal bleeding may also occur.

Risks from Enemas
You may experience some mild discomfort and a bloated or “crampy” feeling. If you have any hemorrhoids or other painful conditions, you might feel anal or rectal discomfort.

Risks from the Applicator
You may experience some discomfort, including small tears and/or bruising in the anorectal area when the clinician rectally inserts the applicator, since it has been designed for vaginal, not rectal use. To minimize the discomfort, the clinician will use a commercially available lubricant to ease insertion of the applicator.

Risks from Flexible Sigmoidoscopy with Biopsies
Flexible sigmoidoscopy is a commonly practiced medical procedure and the endoscopic procedures done in this trial will not involve any unusual risks or discomforts.

- You may experience some mild discomfort and feel bloated, as if you have a bloated stomach.
- You may experience limited rectal bleeding for 1 to 2 days after the procedure
- You may experience low blood pressure after the biopsies
- Even though the risk is low, after the biopsies you may experience infection, mild rectal irritation and may feel a sudden urge to have a bowel movement
- Even though the risk is very rare a perforation (hole or tear) in the intestine may occur. The risk of this complication is estimated to be about 1 in 1,000 procedures. If this happens, antibiotics and surgery to repair the tear may be necessary.

It is not known if the study product will increase the chances of these problems occurring.

Risks from Tenofovir Gel (all formulations) and from Placebo Gel
We do not yet know all the effects of the gels, including how they might affect pregnant women and their unborn babies. Therefore, it is especially important that you do not become pregnant or father a child while on the study.

In addition, we do not yet fully know what effects gels will have on the rectum. In previous studies, the following side effects have been reported:

- Dryness, itching, burning, or pain in the rectal area
- Mild burning, irritation, and discomfort and pruritus (itch)
- A temporary increase in rectal leakage or sense of urgency when having a bowel movement
- When using gel for seven days, more participants experienced gastrointestinal problems
- In a previous study with male volunteers, a few mild side effects on urinary and genital organs occurred; they happended to only a few volunteers and did not need any treatment. The most common symptoms were penile burning, irritation, discomfort, and itching.

Since we do not yet know how tenofovir gel might influence the HIV virus, it might be possible that if you become infected with HIV while the tenofovir is still in your system, the virus could change and become resistant to tenofovir, which means that tenofovir may not work as well if you need to take it for HIV infection.

Risks from Web-based Questionnaires
There may be discomfort or embarrassment related to questions dealing with sexual behaviors and personal habits. If some of the questions upset you or make you uncomfortable, you may choose not to answer them.

Risks from Phone Interview

The phone interview will involve discussions on personal matters, such as sexual behavior. Talking about these issues may make you feel uncomfortable. Remember that the interviewers are professionals trained in sexual research who will keep all your information confidential, and that you can choose not to answer specific questions or stop the interview at any time. These interviews will be recorded and transcribed.

The main risk from participating in the telephone interviews has to do with the loss of confidentiality. To protect your confidentiality, we will keep all tapes of the interviews in a locked cabinet and after the interviews have been transcribed and checked for accuracy, the tapes will be destroyed. If you would like, you are free to suggest a name that we can use during the interviews that may help protect your confidentiality even further. If we chose to use one of your quotes in any documents that describe the interviews, we will describe your comments in ways that will ensure that these quotes cannot be linked to you.

Other Possible Risks
You may become embarrassed, worried, or nervous when discussing ways to protect yourself against HIV and other infections passed during sex and/or when reviewing your test results. You may also become worried or nervous while waiting for your test results and/or sad or depressed upon disclosure of you STI status.

If you have HIV or other infections, knowing this could make you worried or nervous. A trained counselor will help you deal with any feelings or questions you may have. If a sexually transmitted disease has been identified you will be referred immediately for appropriate treatment to your primary care physician. In the event that you do not have a primary care physician, a list of local STI clinics and their phone numbers will be provided. If any sexually transmitted disease, with exception of herpes, is identified, we are required to report this to *[INDICATE LOCAL HEALTH DEPARTMENT AUTHORITIES]*. The reporting of sexually transmitted diseases is done confidentially. Someone from the public health department may contact you to be sure that you and your partners have been treated.

We will make every effort to protect your privacy while you are having the study exams and tests. Your visits here will take place in private. However, it is possible that others may learn that you are taking part in the study here. Because of this, they may treat you unfairly or discriminate against you. For example, you could have problems getting or keeping a job, or being accepted by your family or community. Finding out your HIV status could also cause problems between you and your partner. You may also have problems with your partner associated with use or attempted use of study products.

This research study may involve risks that are currently unforeseeable.

ANTICIPATED BENEFITS TO RESEARCH PARTICIPANTS

You may get no direct benefit from being in this study.

We do not know if tenofovir gel (in any formulation) works to protect against HIV. Because of this, study staff will remind you of the importance of using condoms to protect against HIV.

You or others may benefit in the future from information learned in this study. You may also get some personal satisfaction from being part of research on HIV prevention.

ANTICIPATED BENEFITS TO SOCIETY

Information gathered from this study will help investigators decide whether the tenofovir gel is safe enough to use rectally to move onto the next phase of studies. This knowledge may in the future lead to a microbicide product that could help prevent the spread of HIV.

NEW FINDINGS

During the course of the study, you will be informed of any significant new findings (either good or bad), such as changes in the risks or benefits resulting from participation in the research or new alternatives to participation, that might cause you to change your mind about continuing in the study. If new information is provided to you, your consent to continue participating in this study will be re-obtained.

PARTICIPATION AND WITHDRAWAL

Your participation in this research is voluntary, and if you choose not to participate, that will not affect your relationship with *[INSERT SITE NAME]* or your right to health care or other services at this institution. You are free to withdraw your consent and discontinue participation at any time without prejudice to your future care at *[INSERT SITE NAME].*

The investigators may need to take you off the study early if:

- You become pregnant – in this case, you will not receive any more study products, but you will be asked to continue study visits and to complete any additional follow-ups.
- You do not want to learn your HIV test results.
- The decision is made either to protect your health and safety or because you have developed a certain medical condition and according to a research plan people who develop certain conditions may not proceed with the study
- You are not able to attend the study visits or follow the procedures required by the study
- If you become infected with HIV
- The study is cancelled by the Food and Drug Administration (FDA), the National Institutes of Health (NIH), CONRAD, Office of Human Research Protection (OHPR), or the Institutional Review Board (IRB)

ALTERNATIVES TO PARTICIPATION

You do not have to be in this study. The decision to not be in this study will not affect your care in any way.

COSTS AND FINANCIAL OBLIGATIONS

There will be no cost to you or your insurance carrier for study-related visits, study products, physical examinations, laboratory tests, or other research procedures. If you are found to have a sexually transmitted infection, you will be referred for treatment but any costs associated with this treatment will be your responsibility.

REIMBURSEMENT

You will be paid for your time and effort for all regularly scheduled study visits. You will receive *[INSERT SITE-SPECIFIC REIMBURSEMENT AMOUNT]* for each visit. If required, your transportation costs, such as parking, will also be reimbursed.

PRIVACY AND CONFIDENTIALITY

Efforts will be made to keep your personal information confidential. However, it is not possible to guarantee confidentiality. Your personal information may be disclosed if required by law. The study staff will use your personal information, if needed, to verify that you are not taking part in any other research studies.

Your research samples will be de-identified. The term “de-identified” refers to samples that are labeled with a code that only the research team can link to identifying information. There may be some limited information that is important to this research, such as sex, age, ethnicity, or health, but this information is not enough to identify you without this code. Because samples are de-identified, employers or insurance companies will not be able to discriminate against individual participants.

The only people who will know that you are a research subject are members of the research team and, if appropriate, your physicians and nurses. No information about you, or provided by you during the research will be disclosed to others without your written permission, except:

- if necessary to protect your rights or welfare (for example, if you are injured and need emergency care); or
- if required by law.

Authorized representatives of the *[INSERT NAME SITE IRB OFFICE],* Office for Human Research Protections (OHRP), the Food and Drug Administration (FDA), the National Institutes of Health, study monitors, CONRAD, CHARM Program Cores, and the organizations that provide the gels used in this study may need to review records of individual subjects. As a result, they may see your name; however, they are bound by rules of confidentiality not to reveal your identity to anyone.

Any publication of this study will not use your name or identify you personally. When the results of the research are published or discussed in conferences, no information will be included that would reveal your identity.

[INSERT THE FOLLOWING AS REQUIRED BY SITE IRB]: A description of this clinical trial will be available on www.clinicaltrials.gov, as required by US Law. This website will not include information that can identify you. At most, the website will include a summary of the results. You can search this website at any time.

In addition to the efforts made by the study staff to keep your personal information confidential, a Certificate of Confidentiality will be requested from the U.S. Federal Government for this study. Once obtained, this Certificate will protect study staff from being forced to tell people who are not connected with this study (such as the court system) about your participation or information you give for study purposes. Even with the Certificate of Confidentiality, however, if the study staff learns of possible child abuse and/or neglect or a risk of harm to you or others, they will be required to tell the proper authorities. Having a Certificate of Confidentiality does not prevent you from releasing information about yourself and your participation in the study.

RESEARCH-RELATED INJURY

The U.S. National Institutes of Health (NIH) does not have a mechanism to provide direct compensation for research-related injury, per DAIDS policy (DWD-POL-CL-02.00).

[SITES TO SPECIFY INSTITUTIONAL POLICY]

POSSIBLE COMMERCIAL PRODUCTS

All tissue and/or fluid samples are important to this research study. *[INSERT SITE-SPECIFIC LANGUAGE, AS REQUIRED BY IRB.]*

IDENTIFICATION OF INVESTIGATORS

In the event of a research related injury, or if you experience an adverse reaction, or if you have any questions about this research project, please contact one of the following investigators and/or study staff: *[INSERT NAMES AND PHONE NUMBERS OF SITE INVESTIGATORS AND STUDY STAFF.]*

RIGHTS OF RESEARCH PARTICIPANTS

You may withdraw your consent at any time and discontinue participation at any time without penalty. You are not waiving any legal claims, rights or remedies because of your participation in this research study. *[INSERT SITE-SPECIFIC LANGUAGE, AS REQUIRED BY SITE IRB.]*

If you have questions about your rights as a research participant, please contact *[INSERT CONTACT INFORMATION FOR IRB OR OTHER ORGANIZATION APPROPRIATE FOR THE SITE]*.

SIGNATURE PAGE
[INSERT SIGNATURE BLOCKS AS REQUIRED BY SITE IRB]

If you have read the informed consent, or had it read and explained to you, and all your questions have been answered, please print you name and sign below.

____
Name of Participant (print)

______________________________________ ______________
Signature of Subject Date

____
Name of Investigator/ Staff conducting consent discussion (print)

____ __________
Signature of Investigator/ Staff conducting consent discussion Date

____
Name of Investigator/ Physician performing flexible sigmoidoscopy procedure (print)
(If applicable per site standard)

____ __________
Signature of Investigator/ Date
Physician performing flexible sigmoidoscopy procedure

___________________________ _____________________ __________
Witness' Name (print) Witness's Signature Date
(If applicable per site standard)

[INSERT ATTACHMENTS AS NEEDED]

APPENDIX VI: SAMPLE INFORMED CONSENT FORM (SAMPLE STORAGE)

This is a sample consent form document only. Each participating site is responsible for developing its own informed consent documents. Institutions may require specific informed consent language (e.g., HIPAA language) and have different policies on compensation.

Full Title: CHARM-01 – A Randomized, Double Blind Phase 1 Safety, Acceptability, and Pharmacokinetic Study Comparing Three Formulations of Tenofovir 1% Gel Administered Rectally to HIV-1 Seronegative Adults

Short Title: CHARM-01

Sponsor(s): The study is being paid for by the Division of AIDS, US National Institute of Allergy and Infectious Diseases of the National Institutes of Health and is part of the Combination HIV Antiretroviral Rectal Microbicide (CHARM) Program.

Study Products: Under direction from CONRAD, DPT Laboratories will provide the vaginal formulation (VF) of the tenofovir 1% gel, the reduced glycerin vaginal formulation (RGVF) of the tenofovir 1% gel, and the Universal HEC placebo gel. DPT Laboratories will also provide the rectal formulation (RF) of the tenofovir 1% gel.

Principal Investigator: *[INSERT NAME]*
Key Study Staff: *[INSERT NAME(S)]*
Phone: *[INSERT NUMBER(S)]*

INTRODUCTION

You have decided to participate in a research study investigating the safety of various formulations (mixtures) of the topical gel tenofovir when applied rectally. While you are in this research study, there may be some samples of tissue, blood, and/or fluid collected from you that might be useful for future research. You are being asked to agree to the storage of these samples. This consent form gives you information about the collection, storage and use of your samples. The study staff will discuss this information with you; please ask any questions you might have. If you agree to the storage of your samples, you will be asked to sign this consent form, and copy will be offered for you to keep.

COLLECTION AND USE OF SAMPLES

The research investigators want to save any extra tissue, blood, and/or fluid leftover from your tests during the study. These leftover samples will be kept and used for future research. No additional medical examination or testing is required of you.

The researchers do not plan to contact you or your regular doctor with any results from tests done on your stored samples. This is because research tests are often done with experimental procedures, so the results from one research study are generally not useful for your medical care. If a rare situation came up where the researchers decided that a test result would provide important information for your health, the researchers would tell your study clinician and your study clinician would try to contact you. If you wish to be contacted with this type of test result, you must give the study clinician or nurse any change to your address and/or phone number. If you want your regular doctor to be told about this type of test result, you must provide the study clinician or nurse with your regular doctor's name, address and phone number. Your samples will not be sold or used directly to produce products that can be sold for profit.

You can still be in this study even if you do not want your samples to be stored for future studies.

STORAGE OF SAMPLES

Some of your samples will be stored at laboratory facilities at UCLA's Mucosal Immunology Core Laboratory in Los Angeles, CA and/or at the McGowan Laboratory at the Magee Womens Research Institute in Pittsburgh, PA. These facilities are designed to store samples securely, with only approved researchers permitted to access to the samples. Each university's Institutional Review Board will oversee the storage facilities.

Your samples will be labeled with a unique identifier (such as specimen and test type, date, your subject identification number, and study visit number). The investigators will have sole control over these samples, and only approved researchers will have access to your samples. If your samples are provided to secondary investigators, all subject identifiers will be removed from your samples, and your samples will be made anonymous. Your samples may be stored indefinitely, and the exact time at which your samples will be analyzed has not been determined.

There is no time limit on how long your samples will be stored.

SHARING OF SAMPLES

On the checklist below, we ask for you to indicate if you would permit your samples to be shared with other researchers. If you agree to have your sample shared with other researchers and later decide to withdraw, we may not be able to retrieve any or all of your sample from other researchers. The researcher is not required to store your sample(s) indefinitely.

- ❑ ______ I agree to have my tissue/blood/fluid sample shared with other researchers.
- ❑ ______ I do not want my tissue/blood/fluid sample shared with other researchers.

POTENTIAL RISKS AND DISCOMFORTS

There are few risks related to storing your samples. When tests are done on the stored samples, there is a small risk to your privacy. Although your tissue/blood/fluid samples will be labeled only with your study identification number and your name will not be on any of the samples nor will it be used, researchers who test your samples may be given some information received from you during the study (for example, your age, gender, or medical history). They will never be given your name or information, that when combined could lead to your identification.

This research study may involve risks that are currently unforeseeable.

ANTICIPATED BENEFITS

You will not receive any direct benefit from participating.

ANTICIPATED BENEFITS TO SOCIETY

Information gathered from this study will help investigators decide whether the tenofovir gel is safe enough to use rectally to move onto the next phase of studies. This knowledge may in the future lead to a microbicide product that could help prevent the spread of HIV.

PRIVACY AND CONFIDENTIALITY

Your research samples will be de-identified. The term "de-identified" refers to samples that are labeled with a code that only the research team can link to identifying information. There may be some limited information that is important to this research, such as sex, age, ethnicity, or health, but this information is not enough to identify you without this code. Because samples are de-identified, employers or insurance companies will not be able to discriminate against individual participants. However, sometimes genetic research, even on de-identified samples, may reveal information about an identifiable group.

Because your sample is de-identified, no personal genetic information can be provided to you. You are entitled, however, to any general information developed from this study which may be helpful to the medical care of you or your family members. It is your responsibility to contact the researcher if you want this information.

Authorized representatives of the *[INSERT NAME SITE IRB OFFICE],* Office for Human Research Protections (OHRP), the Food and Drug Administration (FDA), the National Institutes of Health, study monitors, and the organization that provides the gels used in this study may need to review records of individual subjects. As a result, they may see your name; however, they are bound by rules of confidentiality not to reveal your identity to anyone.

IDENTIFICATION OF INVESTIGATORS

If you have any questions about the storage of samples, please contact one of the following investigators and/or study staff: *[INSERT NAMES AND PHONE NUMBERS OF SITE INVESTIGATORS AND STUDY STAFF.]*

RIGHTS OF RESEARCH PARTICIPANTS

Allowing your samples to be stored is completely voluntary. You may decide not to have any samples stored other than what is needed to complete this study and still be in this research study or any future study. If you decide now that your samples can be stored for future research, you may change your mind at any time. You must contact study staff to let them know that you do not want your samples used for future research. Your samples will then not be used and will be destroyed.

You are not waiving any legal claims, rights or remedies because of your participation in this research study. [INSERT SITE-SPECIFIC LANGUAGE, AS REQUIRED BY SITE IRB.]

If you have questions about your rights as a research participant, please contact *[INSERT CONTACT INFORMATION FOR IRB OR OTHER ORGANIZATION APPROPRIATE FOR THE SITE]*.

SIGNATURE PAGE
[INSERT SIGNATURE BLOCKS AS REQUIRED BY SITE IRB]

If you have read this informed consent document (or had it read and explained to you), all your questions have been answered, and you voluntarily agree to have your tissue/blood/fluid samples stored and tested in the future, please print you name and sign below. We will not ask you for your consent again if your samples are tested in the future.

____
Name of Participant (print)

______________________________________ ______________
Signature of Subject Date

____
Name of Investigator/ Staff conducting consent discussion (print)

____ __________
Signature of Investigator/ Staff conducting consent discussion Date

___________________________ _____________________ __________
Witness' Name (print) Witness' Signature Date
(If applicable per site standard)
