# Supplementary material for: A Phase 1 Randomized, Open Label, Rectal Safety, Acceptability, Pharmacokinetic, and Pharmacodynamic Study of Three Formulations of Tenofovir 1% Gel (the CHARM-01 Study)
Source: PLoS One. 2015 May 5;10(5):e0125363. doi: 10.1371/journal.pone.0125363 (PMC4420274; doi:10.1371/journal.pone.0125363)
Supplement: S1 Table — (DOCX) [file pone.0125363.s006.docx]

| **Flow parameter** |  | **Enrollment**  **(n = 14)** | **Mean at 7^th^ Dose** | **Change at 7^th^ Dose**  **(n = 13)** | ***P* value^[[1]](#endnote-1)^*** |
| --- | --- | --- | --- | --- | --- |
| **% CD45^+^** | Mean (SD), Median (25^th^, 75^th^) | 21.0 (8.7), 20.4 (13.2, 26.5) | N, Mean (SD), Median (25^th^, 75^th^) |  |  |
| Enrollment vs. RF D7 at visit 4 | Diff (SE) *P* value |  | 11, 22.1 (11.2), 20.2 (12, 29.8) | -4.41 (2.91) | 0.1293 |
| Enrollment vs. RGVF D7 at visit 4 | Diff (SE) *P* value |  | 13, 20.1 (13.4), 15.4 (11.9, 24.1) | 1.40 (2.06) | 0.4963 |
| Enrollment vs. HEC/VF D7 at visit 4 | Diff (SE) *P* value |  | 12, 20.4 (10.9), 19.4 (12.6, 24.8) | -2.59 (5.58) | 0.6426 |
|  |  |  |  |  |  |
| Change at 7^th^ Dose at visit 4 (RF v RGVF) |  |  |  | -5.81 (3.06) | 0.0575 |
| Change at 7^th^ Dose at visit 4 (RF v HEC/VF) |  |  |  | -1.82 (6.85) | 0.7905 |
| Change at 7^th^ Dose at visit 4 (RGVF v HEC/VF) |  |  |  | 3.99 (5.76) | 0.4894 |
|  |  |  |  |  |  |
| **% CD3^+^ from CD45^+^** | Mean (SD), Median (25^th^, 75^th^) | 44.4 (17.4), 47.2 (34.6, 58.9) |  |  |  |
| Enrollment vs. RF D7 | Diff (SE) *P* value |  | 11, 53.8 (16.4), 54.8 (46.9, 66.2) | 12.23 (5.89) | 0.0380 |
| Enrollment vs. RGVF D7 | Diff (SE) *P* value |  | 13, 50.3 (16.5), 52.5 (46.2, 60.9) | 5.93 (4.73) | 0.2095 |
| Enrollment vs. HEC/VF D7 | Diff (SE) *P* value |  | 12, 54.6 (18.9), 58.3 (49.9, 63.4) | 11.98 (3.46) | 0.0005 |
|  |  |  |  |  |  |
| Change at 7^th^ Dose (RF v RGVF) |  |  |  | 6.29 (4.96) | 0.2045 |
| Change at 7^th^ Dose (RF v HEC/VF) |  |  |  | 0.25 (5.50) | 0.9642 |
| Change at 7^th^ Dose (RGVF v HEC/VF) |  |  |  | -6.04 (6.01) | 0.3145 |
|  |  |  |  |  |  |
| **% CD4^+^ from CD45^+^** | Mean (SD), Median (25^th^, 75^th^) | 62.2 (9.1), 62.4 (54, 68.2) | N, Mean (SD), Median (25^th^, 75^th^) |  |  |
| Enrollment vs. RF D7 | Diff (SE) *P* value |  | 11, 63.5 (11.0), 65.1 (51.1, 71.9) | 0.34 (2.28) | 0.8828 |
| Enrollment vs. RGVF D7 | Diff (SE) *P* value |  | 13, 58.2 (17.3), 60.7 (47.8, 66.9) | -4.92 (4.11) | 0.2304 |
| Enrollment vs. HEC/VF D7 | Diff (SE) *P* value |  | 12, 60.5 (11.1), 61.2 (51.2, 70.3) | -2.60 (2.73) | 0.3404 |
|  |  |  |  |  |  |
| Change at 7^th^ Dose (RF v RGVF) |  |  |  | 5.26 (4.15) | 0.2055 |
| Change at 7^th^ Dose (RF v HEC/VF) |  |  |  | 2.94 (3.11) | 0.3451 |
| Change at 7^th^ Dose (RGVF v HEC/VF) |  |  |  | -2.32 (5.09) | 0.6488 |
|  |  |  |  |  |  |
| **% CCR5^+^ from CD4^+^** | Mean (SD), Median (25^th^, 75^th^) | 79.5 (8.0), 79.8 (76, 85.5) |  |  |  |
| Enrollment vs. RF D7 | Diff (SE) *P* value |  | 11, 78.7 (14.1), 84.0 (66.7, 89) | -0.42 (2.67) | 0.8753 |
| Enrollment vs. RGVF D7 | Diff (SE) *P* value |  | 13, 78.4 (17.3), 84.0 (72.8, 88) | -0.39 (3.64) | 0.9137 |
| Enrollment vs. HEC/VF D7 | Diff (SE) *P* value |  | 12, 78.6 (9.9), 81.3 (73, 85.7) | -0.55 (2.04) | 0.7890 |
|  |  |  |  |  |  |
| Change at 7^th^ Dose (RF v RGVF) |  |  |  | -0.02 (3.16) | 0.9937 |
| Change at 7^th^ Dose (RF v HEC/VF) |  |  |  | 0.13 (2.30) | 0.9563 |
| Change at 7^th^ Dose (RGVF v HEC/VF) |  |  |  | 0.15 (2.71) | 0.9556 |
|  |  |  |  |  |  |
| **% CD38^+^ from CD4^+^** | Mean (SD), Median (25^th^, 75^th^) | 75.1 (11.4), 73.8 (67.3, 86.5) | N, Mean (SD), Median (25^th^, 75^th^) |  |  |
| Enrollment vs. RF D7 | Diff (SE) *P* value |  | 11, 75.3 (11.4), 80.8 (61.8, 83.5) | -0.70 (2.11) | 0.7410 |
| Enrollment vs. RGVF D7 | Diff (SE) *P* value |  | 13, 77.7 (10.9), 77.0 (70.7, 85.3) | 2.74 (2.72) | 0.3151 |
| Enrollment vs. HEC/VF D7 | Diff (SE) *P* value |  | 12, 78.0 (8.1), 78.7 (70.5, 85.3) | 0.86 (1.52) | 0.5738 |
|  |  |  |  |  |  |
| Change at 7^th^ Dose (RF v RGVF) |  |  |  | -3.44 (3.71) | 0.3548 |
| Change at 7^th^ Dose (RF v HEC/VF) |  |  |  | -1.55 (2.80) | 0.5788 |
| Change at 7^th^ Dose (RGVF v HEC/VF) |  |  |  | 1.88 (2.85) | 0.5090 |
|  |  |  |  |  |  |
| **% CXCR4^+^ from CD4^+^** | Mean (SD), Median (25^th^, 75^th^) | 71.5 (16.1), 70.3 (57.9, 84.5) |  |  |  |
| Enrollment vs. RF D7 | Diff (SE) *P* value |  | 11, 69.5 (12.9), 69.8 (63.5, 79.4) | -0.82 (5.62) | 0.8845 |
| Enrollment vs. RGVF D7 | Diff (SE) *P* value |  | 13, 75.6 (16.2), 78.6 (71.1, 85.7) | 5.72 (5.20) | 0.2712 |
| Enrollment vs. HEC/VF D7 | Diff (SE) *P* value |  | 12, 61.1 (23.2), 61.4 (55, 79.5) | -10.68 (5.40) | 0.0480 |
|  |  |  |  |  |  |
| Change at 7^th^ Dose (RF v RGVF) |  |  |  | -6.53 (6.10) | 0.2843 |
| Change at 7^th^ Dose (RF v HEC/VF) |  |  |  | 9.87 (6.63) | 0.1366 |
| Change at 7^th^ Dose (RGVF v HEC/VF) |  |  |  | 16.4 (6.70) | 0.0142 |
|  |  |  |  |  |  |
| **% HLADR^+^ from CD4^+^** | Mean (SD), Median (25^th^, 75^th^) | 43.4 (11.8), 43.3 (35.5, 48.3) | N, Mean (SD), Median (25^th^, 75^th^) |  |  |
| Enrollment vs. RF D7 | Diff (SE) *P* value |  | 11, 40.2 (12.6), 36.6 (28.1, 47.4) | -2.92 (5.05) | 0.5633 |
| Enrollment vs. RGVF D7 | Diff (SE) *P* value |  | 13, 42.7 (20.8), 38.4 (33.7, 41.7) | -1.38 (5.99) | 0.8179 |
| Enrollment vs. HEC/VF D7 | Diff (SE) *P* value |  | 12, 43.3 (13.2), 43.8 (33.7, 50.1) | -0.37 (5.07) | 0.9425 |
|  |  |  |  |  |  |
| Change at 7^th^ Dose (RF v RGVF) |  |  |  | -1.54 (4.90) | 0.7538 |
| Change at 7^th^ Dose (RF v HEC/VF) |  |  |  | -2.55 (3.94) | 0.5176 |
| Change at 7^th^ Dose (RGVF v HEC/VF) |  |  |  | -1.01 (3.86) | 0.7928 |
|  |  |  |  |  |  |
| **% CD69^+^ from CD4^+^** | Mean (SD), Median (25^th^, 75^th^) | 83.4 (6.0), 83.9 (80.4, 87.4) |  |  |  |
| Enrollment vs. RF D7 | Diff (SE) *P* value |  | 11, 83.0 (5.6), 84.2 (79.7, 88.2) | 0.47 (2.29) | 0.8383 |
| Enrollment vs. RGVF D7 | Diff (SE) *P* value |  | 13, 78.0 (13.2), 79.5 (76.8, 84.2) | -4.40 (3.92) | 0.2606 |
| Enrollment vs. HEC/VF D7 | Diff (SE) *P* value |  | 12, 80.5 (4.9), 82.3 (78.7, 83.5) | -2.27 (0.97) | 0.0188 |
|  |  |  |  |  |  |
| Change at 7^th^ Dose (RF v RGVF) |  |  |  | 4.87 (3.77) | 0.1968 |
| Change at 7^th^ Dose (RF v HEC/VF) |  |  |  | 2.74 (1.87) | 0.1432 |
| Change at 7^th^ Dose (RGVF v HEC/VF) |  |  |  | -2.13 (3.55) | 0.5485 |
|  |  |  |  |  |  |
| **% CXCR4^+^ and CCR5^+^ from CD4^+^** | Mean (SD), Median (25^th^, 75^th^) | 55.6 (11.9), 55.1 (43.8, 66.2) | N, Mean (SD), Median (25^th^, 75^th^) |  |  |
| Enrollment vs. RF D7 | Diff (SE) *P* value |  | 11, 53.6 (15.1), 55.7 (48.6, 61.2) | -0.83 (5.53) | 0.8809 |
| Enrollment vs. RGVF D7 | Diff (SE) *P* value |  | 13, 59.1 (18.7), 54.5 (43, 68.9) | 5.55 (5.46) | 0.3094 |
| Enrollment vs. HEC/VF D7 | Diff (SE) *P* value |  | 12, 45.4 (15.9), 48.7 (41.2, 53.9) | -10.16 (4.20) | 0.0157 |
|  |  |  |  |  |  |
| Change at 7^th^ Dose (RF v RGVF) |  |  |  | -6.37 (6.54) | 0.3299 |
| Change at 7^th^ Dose (RF v HEC/VF) |  |  |  | 9.33 (6.38) | 0.1434 |
| Change at 7^th^ Dose (RGVF v HEC/VF) |  |  |  | 15.70 (5.58) | 0.0049 |
|  |  |  |  |  |  |
| **% CD8^+^ from CD45^+^** | Mean (SD), Median (25^th^, 75^th^) | 32.8 (8.4), 31.3 (28, 41) |  |  |  |
| Enrollment vs. RF D7 | Diff (SE) *P* value |  | 11, 31.7 (10.5), 27.7 (25, 42.3) | -0.24 (2.13) | 0.9114 |
| Enrollment vs. RGVF D7 | Diff (SE) *P* value |  | 13, 34.8 (14.4), 32.7 (29.2, 34.5) | 3.12 (3.32) | 0.3473 |
| Enrollment vs. HEC/VF D7 | Diff (SE) *P* value |  | 12, 32.4 (9.6), 29.6 (25.3, 37.8) | 0.64 (1.92) | 0.7411 |
|  |  |  |  |  |  |
| Change at 7^th^ Dose (RF v RGVF) |  |  |  | -3.36 (3.70) | 0.3638 |
| Change at 7^th^ Dose (RF v HEC/VF) |  |  |  | -0.87 (2.17) | 0.6878 |
| Change at 7^th^ Dose (RGVF v HEC/VF) |  |  |  | 2.48 (4.06) | 0.5406 |
|  |  |  |  |  |  |
| **% CCR5^+^ from CD8^+^** | Mean (SD), Median (25^th^, 75^th^) | 82.4 (7.5), 84.6 (81.7, 86.4) | N, Mean (SD), Median (25^th^, 75^th^) |  |  |
| Enrollment vs. RF D7 | Diff (SE) *P* value |  | 11, 82.5 (10.2), 86.3 (76.2, 89.6) | -1.06 (2.13) | 0.6202 |
| Enrollment vs. RGVF D7 | Diff (SE) *P* value |  | 13, 74.3 (23.9), 88.0 (60.8, 89) | -8.62 (5.97) | 0.1491 |
| Enrollment vs. HEC/VF D7 | Diff (SE) *P* value |  | 12, 82.0 (7.8), 81.7 (79.5, 88.8) | -1.11 (1.95) | 0.5691 |
|  |  |  |  |  |  |
| Change at 7^th^ Dose (RF v RGVF) |  |  |  | 7.56 (6.29) | 0.2289 |
| Change at 7^th^ Dose (RF v HEC/VF) |  |  |  | 0.05 (2.20) | 0.9805 |
| Change at 7^th^ Dose (RGVF v HEC/VF) |  |  |  | -7.51 (6.36) | 0.2374 |
|  |  |  |  |  |  |
| **% CD38^+^ from CD8^+^** | Mean (SD), Median (25^th^, 75^th^) | 81.8 (9.5), 84.3 (76.9, 90.8) |  |  |  |
| Enrollment vs. RF D7 | Diff (SE) *P* value |  | 11, 82.5 (9.0), 82.9 (77.2, 89.7) | 0.54 (1.57) | 0.7329 |
| Enrollment vs. RGVF D7 | Diff (SE) *P* value |  | 13, 79.3 (9.6), 81.5 (74.1, 87.2) | -1.19 (2.70) | 0.6600 |
| Enrollment vs. HEC/VF D7 | Diff (SE) *P* value |  | 12, 84.3 (7.0), 85.1 (79.6, 90.4) | 2.01 (1.86) | 0.2812 |
|  |  |  |  |  |  |
| Change at 7^th^ Dose (RF v RGVF) |  |  |  | 1.72 (2.60) | 0.5084 |
| Change at 7^th^ Dose (RF v HEC/VF) |  |  |  | -1.47 (2.34) | 0.5283 |
| Change at 7^th^ Dose (RGVF v HEC/VF) |  |  |  | -3.20 (2.66) | 0.2293 |
|  |  |  |  |  |  |
| **% CXCR4^+^ from CD8^+^** | Mean (SD), Median (25^th^, 75^th^) | 51.2 (17.1), 47.8 (40.2, 68.1) | N, Mean (SD), Median (25^th^, 75^th^) |  |  |
| Enrollment vs. RF D7 | Diff (SE) *P* value |  | 11, 43.7 (20.9), 49.6 (37.2, 57.6) | -6.87 (6.91) | 0.3200 |
| Enrollment vs. RGVF D7 | Diff (SE) *P* value |  | 13, 47.4 (20.3), 53.5 (34.3, 60.5) | -4.65 (5.78) | 0.4216 |
| Enrollment vs. HEC/VF D7 | Diff (SE) *P* value |  | 12, 39.3 (18.8), 38.3 (33.9, 54.2) | -13.38 (4.58) | 0.0035 |
|  |  |  |  |  |  |
| Change at 7^th^ Dose (RF v RGVF) |  |  |  | -2.23 (5.91) | 0.7062 |
| Change at 7^th^ Dose (RF v HEC/VF) |  |  |  | 6.50 (7.42) | 0.3809 |
| Change at 7^th^ Dose (RGVF v HEC/VF) |  |  |  | 8.73 (7.82) | 0.2645 |
|  |  |  |  |  |  |
| **% HLADR^+^ from CD8^+^** | Mean (SD), Median (25^th^, 75^th^) | 53.9 (17.6), 56.7 (43.9, 68.2) |  |  |  |
| Enrollment vs. RF D7 | Diff (SE) *P* value |  | 11, 50.7 (12.4), 50.6 (46.1, 62.8) | -6.05 (4.15) | 0.1445 |
| Enrollment vs. RGVF D7 | Diff (SE) *P* value |  | 13, 49.5 (12.0), 51.0 (40.1, 56.3) | -6.10 (4.64) | 0.1880 |
| Enrollment vs. HEC/VF D7 | Diff (SE) *P* value |  | 12, 50.4 (15.8), 49.4 (40.4, 57.4) | -5.74 (4.67) | 0.2193 |
|  |  |  |  |  |  |
| Change at 7^th^ Dose (RF v RGVF) |  |  |  | 0.05 (3.50) | 0.9886 |
| Change at 7^th^ Dose (RF v HEC/VF) |  |  |  | -0.31 (4.23) | 0.9412 |
| Change at 7^th^ Dose (RGVF v HEC/VF) |  |  |  | -0.36 (3.29) | 0.9124 |
|  |  |  |  |  |  |
| **% CD69^+^ from CD8^+^** | Mean (SD), Median (25^th^, 75^th^) | 85.7 (6.1), 84.6 (82.1, 90.5) | N, Mean (SD), Median (25^th^, 75^th^) |  |  |
| Enrollment vs. RF D7 | Diff (SE) *P* value |  | 11, 84.3 (6.0), 85.2 (78.4, 89.6) | -0.74 (1.43) | 0.6051 |
| Enrollment vs. RGVF D7 | Diff (SE) *P* value |  | 13, 71.4 (26.6), 80.2 (78.1, 84.3) | -13.54 (6.17) | 0.0283 |
| Enrollment vs. HEC/VF D7 | Diff (SE) *P* value |  | 12, 83.4 (3.7), 84.0 (81.3, 85.5) | -1.04 (2.12) | 0.6231 |
|  |  |  |  |  |  |
| Change at 7^th^ Dose (RF v RGVF) |  |  |  | 12.80 (6.02) | 0.0336 |
| Change at 7^th^ Dose (RF v HEC/VF) |  |  |  | 0.30 (2.18) | 0.8906 |
| Change at 7^th^ Dose (RGVF v HEC/VF) |  |  |  | -12.50 (6.79) | 0.0655 |
|  |  |  |  |  |  |
| **% CXCR4^+^ and CCR5^+^ from CD8^+^** | Mean (SD), Median (25^th^, 75^th^) | 43.2 (13.1), 42.5 (35.6, 48) |  |  |  |
| Enrollment vs. RF D7 | Diff (SE) *P* value |  | 11, 37.0 (19.8), 41.1 (18.5, 48.2) | -5.25 (5.99) | 0.3809 |
| Enrollment vs. RGVF D7 | Diff (SE) *P* value |  | 13, 37.8 (17.4), 35.4 (28.4, 50.9) | -5.52 (4.96) | 0.2651 |
| Enrollment vs. HEC/VF D7 | Diff (SE) *P* value |  | 12, 31.5 (13.8), 34.0 (29.1, 37.7) | -12.51 (3.66) | 0.0006 |
|  |  |  |  |  |  |
| Change at 7^th^ Dose (RF v RGVF) |  |  |  | 0.28 (6.37) | 0.9654 |
| Change at 7^th^ Dose (RF v HEC/VF) |  |  |  | 7.26 (6.88) | 0.2910 |
| Change at 7^th^ Dose (RGVF v HEC/VF) |  |  |  | 6.98 (7.25) | 0.3353 |

1. * *P*-value from significance test of relevant contrast from GEE model [↑](#endnote-ref-1)
