# Supplementary material for: A Phase 1 Randomized, Open Label, Rectal Safety, Acceptability, Pharmacokinetic, and Pharmacodynamic Study of Three Formulations of Tenofovir 1% Gel (the CHARM-01 Study)
Source: PLoS One. 2015 May 5;10(5):e0125363. doi: 10.1371/journal.pone.0125363 (PMC4420274; doi:10.1371/journal.pone.0125363)
Supplement: S2 Table — (DOCX) [file pone.0125363.s007.docx]

| **Rectal Microflora Cultures (Anaerobic)** | **1^st^ Dose** | **24hr Post Dose** | **Change**  **(n = 13)** | ***P* Value^[[1]](#endnote-1)^*** |
| --- | --- | --- | --- | --- |
|  | **Descriptive Statistics** | **Descriptive Statistics** |  |  |
|  | N, Mean (SD), Median (25^th^, 75^th^) | N, Mean (SD), Median (25^th^, 75^th^) | Diff (SE) |  |
|  |  |  |  |  |
| **Bacteriodes Fragilis** |  |  |  |  |
| RF: 1^st^ Dose vs. 24hr Post Dose | 12, 2.1 (1.4), 2.5 (1, 3) | 12, 2.1 (1.6), 2.0 (0.5, 4) | -0.08 (0.54) | 0.8870 |
| RGVF: 1^st^ Dose vs. 24hr Post Dose | 13, 2.2 (1.6), 2.0 (1, 4) | 13, 3.2 (1.1), 3.0 (3, 4) | 1.05 (0.53) | 0.0460 |
| HEC/VF: 1^st^ Dose vs. 24hr Post Dose | 12, 3.1 (0.7), 3.0 (3, 3.5) | 12, 2.1 (1.2), 2.0 (2, 3) | -0.99 (0.32) | 0.0018 |
|  |  |  |  |  |
| Change at 24hr Post Dose (RF v RGVF) |  |  | -1.13 (0.61) | 0.0630 |
| Change at 24hr Post Dose (RF v HEC/VF) |  |  | 0.91 (0.75) | 0.2269 |
| Change at 24hr Post Dose (RGVF v HEC/VF) |  |  | 2.04 (0.73) | 0.0051 |
|  |  |  |  |  |
| **Gram negative rods, other non-pigmented** |  |  |  |  |
| RF: 1^st^ Dose vs. 24hr Post Dose | 12, 2.3 (1.3), 3.0 (1.5, 3) | 12, 2.3 (1.4), 2.0 (2, 3.5) | -0.05 (0.52) | 0.9265 |
| RGVF: 1^st^ Dose vs. 24hr Post Dose | 13, 2.6 (1.0), 3.0 (2, 3) | 13, 2.6 (1.1), 3.0 (2, 3) | 0.05 (0.37) | 0.8910 |
| HEC/VF: 1^st^ Dose vs. 24hr Post Dose | 12, 2.6 (1.2), 2.5 (2, 3.5) | 12, 2.7 (1.1), 2.0 (2, 4) | 0.07 (0.34) | 0.8430 |
|  |  |  |  |  |
| Change at 24hr Post Dose (RF v RGVF) |  |  | -0.10 (0.49) | 0.8401 |
| Change at 24hr Post Dose (RF v HEC/VF) |  |  | -0.12 (0.52) | 0.8222 |
| Change at 24hr Post Dose (RGVF v HEC/VF) |  |  | -0.02 (0.50) | 0.9725 |
|  |  |  |  |  |
| **Gram negative pigmented** |  |  |  |  |
| RF: 1^st^ Dose vs. 24hr Post Dose | 12, 1.8 (1.1), 2.0 (1, 3) | 12, 1.7 (1.2), 2.0 (1, 2) | -0.11 (0.12) | 0.3598 |
| RGVF: 1^st^ Dose vs. 24hr Post Dose | 13, 1.8 (1.0), 2.0 (1, 2) | 13, 1.6 (1.3), 2.0 (1, 2) | -0.23 (0.30) | 0.4463 |
| HEC/VF: 1^st^ Dose vs. 24hr Post Dose | 12, 1.7 (1.2), 2.0 (0.5, 3) | 12, 1.3 (1.0), 1.5 (0.5, 2) | -0.33 (0.37) | 0.3642 |
|  |  |  |  |  |
| Change at 24hr Post Dose (RF v RGVF) |  |  | 0.12 (0.34) | 0.7290 |
| Change at 24hr Post Dose (RF v HEC/VF) |  |  | 0.22 (0.31) | 0.4697 |
| Change at 24hr Post Dose (RGVF v HEC/VF) |  |  | 0.10 (0.45) | 0.8186 |
| **Rectal Microflora Cultures (Anaerobic)** | **1^st^ Dose** | **24hr Post Dose** | **Change**  **(n = 13)** | ***P* Value^*^** |
|  | **Descriptive Statistics** | **Descriptive Statistics** |  |  |
|  | N, Mean (SD), Median (25^th^, 75^th^) | N, Mean (SD), Median (25^th^, 75^th^) | Diff (SE) |  |
| **Gram positive cocci** |  |  |  |  |
| RF: 1^st^ Dose vs. 24hr Post Dose | 12, 2.2 (1.2), 3.0 (1.5, 3) | 12, 2.6 (1.4), 3.0 (2, 4) | 0.42 (0.47) | 0.3709 |
| RGVF: 1^st^ Dose vs. 24hr Post Dose | 13, 3.0 (0.8), 3.0 (2, 4) | 13, 2.7 (1.3), 3.0 (2, 4) | -0.30 (0.38) | 0.4335 |
| HEC/VF: 1^st^ Dose vs. 24hr Post Dose | 12, 2.6 (1.0), 2.0 (2, 3.5) | 12, 2.6 (1.0), 3.0 (2, 3) | -0.01 (0.35) | 0.9802 |
|  |  |  |  |  |
| Change at 24hr Post Dose (RF v RGVF) |  |  | 0.72 (0.76) | 0.3431 |
| Change at 24hr Post Dose (RF v HEC/VF) |  |  | 0.43 (0.56) | 0.4405 |
| Change at 24hr Post Dose (RGVF v HEC/VF) |  |  | -0.29 (0.52) | 0.5709 |
|  |  |  |  |  |
| **Gram positive rods, Clostridium-like** |  |  |  |  |
| RF: 1^st^ Dose vs. 24hr Post Dose | 12, 0.7 (1.2), 0.0 (0, 1) | 12, 0.6 (1.2), 0.0 (0, 0.5) | -0.09 (0.52) | 0.8659 |
| RGVF: 1^st^ Dose vs. 24hr Post Dose | 13, 0.6 (0.8), 0.0 (0, 1) | 13, 0.6 (1.0), 0.0 (0, 2) | 0.01 (0.29) | 0.9599 |
| HEC/VF: 1^st^ Dose vs. 24hr Post Dose | 12, 0.8 (1.0), 0.5 (0, 1.5) | 12, 0.7 (1.2), 0.0 (0, 1) | -0.16 (0.47) | 0.7265 |
|  |  |  |  |  |
| Change at 24hr Post Dose (RF v RGVF) |  |  | -0.10 (0.54) | 0.8501 |
| Change at 24hr Post Dose (RF v HEC/VF) |  |  | 0.08 (0.38) | 0.8433 |
| Change at 24hr Post Dose (RGVF v HEC/VF) |  |  | 0.18 (0.42) | 0.6687 |
|  |  |  |  |  |
| **Gram positive rods, other** |  |  |  |  |
| RF: 1^st^ Dose vs. 24hr Post Dose | 12, 2.5 (1.4), 3.0 (2, 3.5) | 12, 2.2 (1.5), 3.0 (0.5, 3) | -0.23 (0.53) | 0.6697 |
| RGVF: 1^st^ Dose vs. 24hr Post Dose | 13, 1.9 (1.6), 2.0 (0, 3) | 13, 1.8 (1.5), 2.0 (0, 3) | -0.22 (0.47) | 0.6468 |
| HEC/VF: 1^st^ Dose vs. 24hr Post Dose | 12, 2.2 (1.5), 2.0 (1, 3.5) | 12, 2.1 (1.4), 2.0 (1, 3) | -0.04 (0.57) | 0.9393 |
|  |  |  |  |  |
| Change at 24hr Post Dose (RF v RGVF) |  |  | -0.01 (0.80) | 0.9907 |
| Change at 24hr Post Dose (RF v HEC/VF) |  |  | -0.18 (0.94) | 0.8456 |
| Change at 24hr Post Dose (RGVF v HEC/VF) |  |  | -0.17 (0.70) | 0.8039 |
|  |  |  |  |  |

1. * *P*-value from significance test of relevant contrast from GEE model [↑](#endnote-ref-1)
