# Supplementary material for: A Phase 1 Randomized, Open Label, Rectal Safety, Acceptability, Pharmacokinetic, and Pharmacodynamic Study of Three Formulations of Tenofovir 1% Gel (the CHARM-01 Study)
Source: PLoS One. 2015 May 5;10(5):e0125363. doi: 10.1371/journal.pone.0125363 (PMC4420274; doi:10.1371/journal.pone.0125363)
Supplement: S3 Table — (DOCX) [file pone.0125363.s008.docx]

| **Rectal Microflora Cultures (Facultative Isolates)** | **1^st^ Dose** | **24hr Post Dose** | **Change**  **(n = 13)** | | ***P* Value^[[1]](#endnote-1)^*** |
| --- | --- | --- | --- | --- | --- |
|  | **Descriptive Statistics** | **Descriptive Statistics** |  |  |  |
|  | N, Mean (SD), Median (25^th^, 75^th^) | N, Mean (SD), Median (25^th^, 75^th^) | Diff (SE) | |  |
| **Lactobacillus, H2O2 positive** |  |  |  | |  |
| RF: 1^st^ Dose vs. 24hr Post Dose | 12, 0.5 (0.9), 0.0 (0, 1) | 12, 0.8 (0.9), 0.5 (0, 2) | 0.34 (0.25) | | 0.1725 |
| RGVF: 1^st^ Dose vs. 24hr Post Dose | 13, 0.5 (0.9), 0.0 (0, 1) | 13, 0.4 (0.7), 0.0 (0, 1) | -0.17 (0.13) | | 0.1951 |
| HEC/VF: 1^st^ Dose vs. 24hr Post Dose | 12, 0.4 (0.8), 0.0 (0, 0.5) | 12, 0.3 (0.7), 0.0 (0, 0.5) | -0.08 (0.22) | | 0.7000 |
|  |  |  |  | |  |
| Change at 24hr Post Dose (RF v RGVF) |  |  | 0.51 (0.29) | | 0.0785 |
| Change at 24hr Post Dose (RF v HEC/VF) |  |  | 0.42 (0.39) | | 0.2795 |
| Change at 24hr Post Dose (RGVF v HEC/VF) |  |  | -0.08 (0.29) | | 0.7705 |
|  |  |  |  | |  |
| **Lactobacillus, H2O2 negative** |  |  |  | |  |
| RF: 1^st^ Dose vs. 24hr Post Dose | 12, 0.8 (1.1), 0.0 (0, 2) | 12, 0.3 (0.6), 0.0 (0, 0) | -0.57 (0.26) | | 0.0325 |
| RGVF: 1^st^ Dose vs. 24hr Post Dose | 13, 0.5 (1.0), 0.0 (0, 0) | 13, 0.4 (0.8), 0.0 (0, 0) | 0.02 (0.34) | | 0.9574 |
| HEC/VF: 1^st^ Dose vs. 24hr Post Dose | 12, 0.3 (0.9), 0.0 (0, 0) | 12, 0.4 (1.0), 0.0 (0, 0) | 0.18 (0.23) | | 0.4415 |
|  |  |  |  | |  |
| Change at 24hr Post Dose (RF v RGVF) |  |  | -0.58 (0.50) | | 0.2412 |
| Change at 24hr Post Dose (RF v HEC/VF) |  |  | -0.74 (0.24) | | 0.0023 |
| Change at 24hr Post Dose (RGVF v HEC/VF) |  |  | -0.16 (0.41) | | 0.7000 |
|  |  |  |  | |  |
| **Gardnerella vaginalis** |  |  |  | |  |
| RF: 1^st^ Dose vs. 24hr Post Dose | 12, 0.4 (0.8), 0.0 (0, 0.5) | 12, 0.3 (0.9), 0.0 (0, 0) | -0.19 (0.22) | | 0.3883 |
| RGVF: 1^st^ Dose vs. 24hr Post Dose | 13, 0.4 (1.0), 0.0 (0, 0) | 13, 0.4 (1.0), 0.0 (0, 0) | 0.04 (0.27) | | 0.8722 |
| HEC/VF: 1^st^ Dose vs. 24hr Post Dose | 12, 0.2 (0.6), 0.0 (0, 0) | 12, 0.4 (1.0), 0.0 (0, 0) | 0.26 (0.33) | | 0.4448 |
|  |  |  |  | |  |
| Change at 24hr Post Dose (RF v RGVF) |  |  | -0.24 (0.21) | | 0.2633 |
| Change at 24hr Post Dose (RF v HEC/VF) |  |  | -0.45 (0.43) | | 0.2935 |
| Change at 24hr Post Dose (RGVF v HEC/VF) |  |  | -0.21 (0.29) | | 0.4644 |
|  |  |  |  | |  |
| **Rectal Microflora Cultures (Facultative Isolates)** | **1^st^ Dose** | **24hr Post Dose** | **Change**  **(n = 13)** | | ***P* Value^*^** |
|  | **Descriptive Statistics** | **Descriptive Statistics** |  |  |  |
|  | N, Mean (SD), Median (25^th^, 75^th^) | N, Mean (SD), Median (25^th^, 75^th^) | Diff (SE) | |  |
| **Diphtheroids** |  |  |  | |  |
| RF: 1^st^ Dose vs. 24hr Post Dose | 12, 1.7 (1.2), 1.0 (1, 2.5) | 12, 1.7 (1.2), 2.0 (0.5, 3) | 0.05 (0.45) | | 0.9172 |
| RGVF: 1^st^ Dose vs. 24hr Post Dose | 13, 1.5 (1.2), 1.0 (1, 2) | 13, 1.6 (1.4), 2.0 (0, 2) | 0.09 (0.55) | | 0.8688 |
| HEC/VF: 1^st^ Dose vs. 24hr Post Dose | 12, 1.4 (1.2), 1.0 (0.5, 2) | 12, 1.8 (0.9), 2.0 (1, 2) | 0.34 (0.37) | | 0.3513 |
|  |  |  |  | |  |
| Change at 24hr Post Dose (RF v RGVF) |  |  | -0.04 (0.81) | | 0.9568 |
| Change at 24hr Post Dose (RF v HEC/VF) |  |  | -0.30 (0.72) | | 0.6821 |
| Change at 24hr Post Dose (RGVF v HEC/VF) |  |  | -0.25 (0.62) | | 0.6847 |
|  |  |  |  | |  |
| **Bacillus** |  |  |  | |  |
| RF: 1^st^ Dose vs. 24hr Post Dose | 12, 0.0 (0.0), 0.0 (0, 0) | 12, 0.0 (0.0), 0.0 (0, 0) | 0.00 (0.00) | | >0.9999 |
| RGVF: 1^st^ Dose vs. 24hr Post Dose | 13, 0.0 (0.0), 0.0 (0, 0) | 13, 0.0 (0.0), 0.0 (0, 0) | 0.00 (0.00) | | >0.9999 |
| HEC/VF: 1^st^ Dose vs. 24hr Post Dose | 12, 0.1 (0.3), 0.0 (0, 0) | 12, 0.1 (0.3), 0.0 (0, 0) | 0.00 (0.12) | | >0.9999 |
|  |  |  |  | |  |
| Change at 24hr Post Dose (RF v RGVF) |  |  | 0.00 (0.00) | | >0.9999 |
| Change at 24hr Post Dose (RF v HEC/VF) |  |  | 0.00 (0.12) | | >0.9999 |
| Change at 24hr Post Dose (RGVF v HEC/VF) |  |  | 0.00 (0.12) | | >0.9999 |
|  |  |  |  | |  |
| **Gram positive rods, other** |  |  |  | |  |
| RF: 1^st^ Dose vs. 24hr Post Dose | 12, 2.0 (1.1), 2.0 (1, 3) | 12, 1.4 (1.3), 2.0 (0, 2.5) | -0.68 (0.34) | | 0.0422 |
| RGVF: 1^st^ Dose vs. 24hr Post Dose | 13, 1.2 (1.0), 2.0 (0, 2) | 13, 1.1 (1.3), 1.0 (0, 2) | 0.10 (0.51) | | 0.8495 |
| HEC/VF: 1^st^ Dose vs. 24hr Post Dose | 12, 2.0 (1.3), 2.0 (1.5, 2.5) | 12, 1.6 (1.6), 1.5 (0, 3) | -0.40 (0.53) | | 0.4520 |
|  |  |  |  | |  |
| Change at 24hr Post Dose (RF v RGVF) |  |  | -0.78 (0.41) | | 0.0556 |
| Change at 24hr Post Dose (RF v HEC/VF) |  |  | -0.29 (0.60) | | 0.6341 |
| Change at 24hr Post Dose (RGVF v HEC/VF) |  |  | 0.49 (0.62) | | 0.4274 |
|  |  |  |  | |  |
| **Rectal Microflora Cultures (Facultative Isolates)** | **1^st^ Dose** | **24hr Post Dose** | **Change**  **(n = 13)** | | ***P* Value^*^** |
|  | **Descriptive Statistics** | **Descriptive Statistics** |  |  |  |
|  | N, Mean (SD), Median (25^th^, 75^th^) | N, Mean (SD), Median (25^th^, 75^th^) | Diff (SE) | |  |
| **Group B Streptococcus** |  |  |  |  | |
| RF: 1^st^ Dose vs. 24hr Post Dose | 12, 0.3 (0.9), 0.0 (0, 0) | 12, 0.7 (1.2), 0.0 (0, 1) | 0.32 (0.41) | | 0.4344 |
| RGVF: 1^st^ Dose vs. 24hr Post Dose | 13, 0.6 (1.5), 0.0 (0, 0) | 13, 0.6 (1.2), 0.0 (0, 1) | 0.04 (0.25) | | 0.8891 |
| HEC/VF: 1^st^ Dose vs. 24hr Post Dose | 12, 0.4 (0.8), 0.0 (0, 0.5) | 12, 0.8 (1.1), 0.0 (0, 1.5) | 0.34 (0.34) | | 0.3227 |
|  |  |  |  | |  |
| Change at 24hr Post Dose (RF v RGVF) |  |  | 0.29 (0.41) | | 0.4891 |
| Change at 24hr Post Dose (RF v HEC/VF) |  |  | -0.02 (0.42) | | 0.9705 |
| Change at 24hr Post Dose (RGVF v HEC/VF) |  |  | -0.30 (0.41) | | 0.4610 |
|  |  |  |  | |  |
| **Enterococcus** |  |  |  | |  |
| RF: 1^st^ Dose vs. 24hr Post Dose | 12, 0.4 (0.9), 0.0 (0, 0.5) | 12, 0.7 (1.4), 0.0 (0, 0.5) | 0.27 (0.47) | | 0.5711 |
| RGVF: 1^st^ Dose vs. 24hr Post Dose | 13, 0.4 (1.0), 0.0 (0, 0) | 13, 0.5 (1.0), 0.0 (0, 0) | 0.04 (0.41) | | 0.9174 |
| HEC/VF: 1^st^ Dose vs. 24hr Post Dose | 12, 0.2 (0.4), 0.0 (0, 0) | 12, 0.0 (0.0), 0.0 (0, 0) | -0.16 (0.14) | | 0.2519 |
|  |  |  |  | |  |
| Change at 24hr Post Dose (RF v RGVF) |  |  | 0.23 (0.68) | | 0.7392 |
| Change at 24hr Post Dose (RF v HEC/VF) |  |  | 0.43 (0.55) | | 0.4321 |
| Change at 24hr Post Dose (RGVF v HEC/VF) |  |  | 0.21 (0.42) | | 0.6196 |
|  |  |  |  | |  |
| **Staphylococcus Aureus** |  |  |  | |  |
| RF: 1^st^ Dose vs. 24hr Post Dose | 12, 0.0 (0.0), 0.0 (0, 0) | 12, 0.0 (0.0), 0.0 (0, 0) | -0.01 (0.01) | | 0.5887 |
| RGVF: 1^st^ Dose vs. 24hr Post Dose | 13, 0.0 (0.0), 0.0 (0, 0) | 13, 0.0 (0.0), 0.0 (0, 0) | 0.01 (0.01) | | 0.4517 |
| HEC/VF: 1^st^ Dose vs. 24hr Post Dose | 12, 0.1 (0.3), 0.0 (0, 0) | 12, 0.0 (0.0), 0.0 (0, 0) | -0.08 (0.08) | | 0.2710 |
|  |  |  |  | |  |
| Change at 24hr Post Dose (RF v RGVF) |  |  | -0.02 (0.02) | | 0.4740 |
| Change at 24hr Post Dose (RF v HEC/VF) |  |  | 0.08 (0.07) | | 0.2699 |
| Change at 24hr Post Dose (RGVF v HEC/VF) |  |  | 0.09 (0.08) | | 0.2625 |
|  |  |  |  | |  |
| **Rectal Microflora Cultures (Facultative Isolates)** | **1^st^ Dose** | **24hr Post Dose** | **Change**  **(n = 13)** | | ***P* Value^*^** |
|  | **Descriptive Statistics** | **Descriptive Statistics** |  |  |  |
|  | N, Mean (SD), Median (25^th^, 75^th^) | N, Mean (SD), Median (25^th^, 75^th^) | Diff (SE) | |  |
| **Staphylococcus coagulase-negative** |  |  |  | |  |
| RF: 1^st^ Dose vs. 24hr Post Dose | 12, 1.2 (1.1), 1.0 (0, 2) | 12, 1.2 (0.7), 1.0 (1, 2) | 0.08 (0.38) | | 0.8379 |
| RGVF: 1^st^ Dose vs. 24hr Post Dose | 13, 0.6 (0.9), 0.0 (0, 1) | 13, 0.8 (1.0), 1.0 (0, 1) | 0.15 (0.29) | | 0.5993 |
| HEC/VF: 1^st^ Dose vs. 24hr Post Dose | 12, 0.7 (0.8), 0.5 (0, 1) | 12, 0.6 (0.7), 0.5 (0, 1) | -0.07 (0.31) | | 0.8183 |
|  |  |  |  | |  |
| Change at 24hr Post Dose (RF v RGVF) |  |  | -0.08 (0.48) | | 0.8756 |
| Change at 24hr Post Dose (RF v HEC/VF) |  |  | 0.15 (0.37) | | 0.6903 |
| Change at 24hr Post Dose (RGVF v HEC/VF) |  |  | 0.22 (0.38) | | 0.6674 |
|  |  |  |  | |  |
| **Micrococcus** |  |  |  | |  |
| RF: 1^st^ Dose vs. 24hr Post Dose | 12, 0.1 (0.3), 0.0 (0, 0) | 12, 0.0 (0.0), 0.0 (0, 0) | -0.07 (0.07) | | 0.3082 |
| RGVF: 1^st^ Dose vs. 24hr Post Dose | 13, 0.0 (0.0), 0.0 (0, 0) | 13, 0.1 (0.3), 0.0 (0, 0) | 0.06 (0.06) | | 0.3428 |
| HEC/VF: 1^st^ Dose vs. 24hr Post Dose | 12, 0.1 (0.3), 0.0 (0, 0) | 12, 0.1 (0.3), 0.0 (0, 0) | -0.00 (0.02) | | 0.9551 |
|  |  |  |  | |  |
| Change at 24hr Post Dose (RF v RGVF) |  |  | -0.13 (0.08) | | 0.0978 |
| Change at 24hr Post Dose (RF v HEC/VF) |  |  | -0.07 (0.07) | | 0.2979 |
| Change at 24hr Post Dose (RGVF v HEC/VF) |  |  | 0.06 (0.07) | | 0.3641 |
|  |  |  |  | |  |
| **Viridans Streptococcus H2O2-positive** |  |  |  | |  |
| RF: 1^st^ Dose vs. 24hr Post Dose | 12, 1.4 (1.2), 1.5 (0, 2.5) | 12, 0.6 (1.1), 0.0 (0, 1) | -0.75 (0.54) | | 0.1635 |
| RGVF: 1^st^ Dose vs. 24hr Post Dose | 13, 1.4 (1.3), 2.0 (0, 2) | 13, 1.5 (1.3), 0.0 (0, 2) | -0.02 (0.41) | | 0.9679 |
| HEC/VF: 1^st^ Dose vs. 24hr Post Dose | 12, 1.3 (1.4), 1.5 (0, 2) | 12, 0.8 (1.2), 0.0 (0, 1.5) | -0.50 (0.20) | | 0.0110 |
|  |  |  |  | |  |
| Change at 24hr Post Dose (RF v RGVF) |  |  | -0.73 (0.66) | | 0.2700 |
| Change at 24hr Post Dose (RF v HEC/VF) |  |  | -0.25 (0.59) | | 0.6761 |
| Change at 24hr Post Dose (RGVF v HEC/VF) |  |  | 0.49 (0.50) | | 0.3270 |
|  |  |  |  | |  |
| **Rectal Microflora Cultures (Facultative Isolates)** | **1^st^ Dose** | **24hr Post Dose** | **Change**  **(n = 13)** | | **P Value^*^** |
|  | **Descriptive Statistics** | **Descriptive Statistics** |  |  |  |
|  | N, Mean (SD), Median (25^th^, 75^th^) | N, Mean (SD), Median (25^th^, 75^th^) | Diff (SE) | |  |
| **Viridans Streptococcus H2O2-negative** |  |  |  | |  |
| RF: 1^st^ Dose vs. 24hr Post Dose | 12, 0.6 (1.2), 0.0 (0, 0.5) | 12, 0.9 (1.4), 0.0 (0, 2) | 0.25 (0.63) | | 0.6916 |
| RGVF: 1^st^ Dose vs. 24hr Post Dose | 13, 1.5 (1.3), 2.0 (0, 3) | 13, 1.4 (1.3), 1.0 (0, 2) | 0.05 (0.47) | | 0.9167 |
| HEC/VF: 1^st^ Dose vs. 24hr Post Dose | 12, 1.3 (1.4), 1.5 (0, 2) | 12, 1.7 (1.4), 2.0 (0, 2.5) | 0.33 (0.48) | | 0.4842 |
|  |  |  |  | |  |
| Change at 24hr Post Dose (RF v RGVF) |  |  | 0.20 (0.73) | | 0.7849 |
| Change at 24hr Post Dose (RF v HEC/VF) |  |  | -0.08 (0.79) | | 0.9164 |
| Change at 24hr Post Dose (RGVF v HEC/VF) |  |  | -0.28 (0.79) | | 0.7211 |
|  |  |  |  | |  |
| **Gram positive cocci, other** |  |  |  | |  |
| RF: 1^st^ Dose vs. 24hr Post Dose | 12, 1.3 (1.6), 0.0 (0, 3) | 12, 0.6 (0.9), 0.0 (0, 1.5) | -0.69 (0.58) | | 0.2303 |
| RGVF: 1^st^ Dose vs. 24hr Post Dose | 13, 1.8 (1.6), 2.0 (0, 3) | 13, 1.0 (1.2), 1.0 (0, 2) | -0.70 (0.45) | | 0.1182 |
| HEC/VF: 1^st^ Dose vs. 24hr Post Dose | 12, 0.8 (1.1), 0.0 (0, 2) | 12, 0.9 (1.6), 0.0 (0, 1.5) | 0.10 (0.30) | | 0.7329 |
|  |  |  |  | |  |
| Change at 24hr Post Dose (RF v RGVF) |  |  | 0.01 (0.75) | | 0.9905 |
| Change at 24hr Post Dose (RF v HEC/VF) |  |  | -0.79 (0.71) | | 0.2634 |
| Change at 24hr Post Dose (RGVF v HEC/VF) |  |  | -0.80 (0.60) | | 0.1840 |
|  |  |  |  | |  |
| **Escherichia Coli** |  |  |  | |  |
| RF: 1^st^ Dose vs. 24hr Post Dose | 12, 1.8 (1.2), 2.0 (1, 2.5) | 12, 1.5 (1.6), 1.0 (0, 3) | -0.33 (0.29) | | 0.2554 |
| RGVF: 1^st^ Dose vs. 24hr Post Dose | 13, 2.2 (1.1), 2.0 (2, 3) | 13, 2.6 (1.0), 2.0 (2, 3) | 0.37 (0.29) | | 0.2018 |
| HEC/VF: 1^st^ Dose vs. 24hr Post Dose | 12, 1.3, (1.3), 1.0 (0, 2.5) | 12, 1.9 (1.6), 2.0 (0.5, 3.5) | 0.66 (0.39) | | 0.0880 |
|  |  |  |  | |  |
| Change at 24hr Post Dose (RF v RGVF) |  |  | -0.69 (0.39) | | 0.0748 |
| Change at 24hr Post Dose (RF v HEC/VF) |  |  | -0.98 (0.39) | | 0.0117 |
| Change at 24hr Post Dose (RGVF v HEC/VF) |  |  | -0.29 (0.54) | | 0.5881 |
|  |  |  |  | |  |
| **Rectal Microflora Cultures (Facultative Isolates)** | **1^st^ Dose** | **24hr Post Dose** | **Change**  **(n = 13)** | | ***P* Value^*^** |
|  | **Descriptive Statistics** | **Descriptive Statistics** |  |  |  |
|  | N, Mean (SD), Median (25^th^, 75^th^) | N, Mean (SD), Median (25^th^, 75^th^) | Diff (SE) | |  |
| **Proteus** |  |  |  | |  |
| RF: 1^st^ Dose vs. 24hr Post Dose | 12, 0.0 (0.0), 0.0 (0, 0) | 12, 0.0 (0.0), 0.0 (0, 0) |  | |  |
| RGVF: 1^st^ Dose vs. 24hr Post Dose | 13, 0.0 (0.0), 0.0 (0, 0) | 13, 0.0 (0.0), 0.0 (0, 0) |  | |  |
| HEC/VF: 1^st^ Dose vs. 24hr Post Dose | 12, 0.0 (0.0), 0.0 (0, 0) | 12, 0.0 (0.0), 0.0 (0, 0) |  | |  |
|  |  |  |  | |  |
| Change at 24hr Post Dose (RF v RGVF) |  |  |  | |  |
| Change at 24hr Post Dose (RF v HEC/VF) |  |  |  | |  |
| Change at 24hr Post Dose (RGVF v HEC/VF) |  |  |  | |  |
|  |  |  |  | |  |
| **Gram negative rods, other** |  |  |  | |  |
| RF: 1^st^ Dose vs. 24hr Post Dose | 12, 0.1 (0.3), 0.0 (0, 0) | 12, 0.5 (1.2), 0.0 (0, 0) | 0.40 (0.31) | | 0.1872 |
| RGVF: 1^st^ Dose vs. 24hr Post Dose | 13, 0.0 (0.0), 0.0 (0, 0) | 13, 0.2 (0.6), 0.0 (0, 0) | 0.25 (0.18) | | 0.1693 |
| HEC/VF: 1^st^ Dose vs. 24hr Post Dose | 12, 0.4 (0.7), 0.0 (0, 1) | 12, 0.2 (0.4), 0.0 (0, 0) | -0.25 (0.20) | | 0.2225 |
|  |  |  |  | |  |
| Change at 24hr Post Dose (RF v RGVF) |  |  | 0.16 (0.41) | | 0.7023 |
| Change at 24hr Post Dose (RF v HEC/VF) |  |  | 0.65 (0.34) | | 0.0556 |
| Change at 24hr Post Dose (RGVF v HEC/VF) |  |  | 0.50 (0.30) | | 0.0997 |
|  |  |  |  | |  |
| **Candida yeast** |  |  |  | |  |
| RF: 1^st^ Dose vs. 24hr Post Dose | 12, 0.0 (0.0), 0.0 (0, 0) | 12, 0.0 (0.0), 0.0 (0, 0) |  | |  |
| RGVF: 1^st^ Dose vs. 24hr Post Dose | 13, 0.0 (0.0), 0.0 (0, 0) | 13, 0.0 (0.0), 0.0 (0, 0) |  | |  |
| HEC/VF: 1^st^ Dose vs. 24hr Post Dose | 12, 0.0 (0.0), 0.0 (0, 0) | 12, 0.0 (0.0), 0.0 (0, 0) |  | |  |
|  |  |  |  | |  |
| Change at 24hr Post Dose (RF v RGVF) |  |  |  | |  |
| Change at 24hr Post Dose (RF v HEC/VF) |  |  |  | |  |
| Change at 24hr Post Dose (RGVF v HEC/VF) |  |  |  | |  |

1. * *P*-value from significance test of relevant contrast from GEE model [↑](#endnote-ref-1)
